# Supplementary material for: Spatially resolved gene expression profiling of tumor microenvironment reveals key steps of lung adenocarcinoma development
Source: Nat Commun. 2024 Dec 6;15:10637. doi: 10.1038/s41467-024-54671-7 (PMC11621540; doi:10.1038/s41467-024-54671-7)
Supplement: Supplementary file 1 — Supplementary Information [file 41467_2024_54671_MOESM1_ESM.pdf]

SUPPLEMENTARY INFORMATION

# Spatially resolved gene expression profiling of tumor microenvironment reveals key steps of lung adenocarcinoma development

Yuma Takano, Jun Suzuki, Kotaro Nomura, Gento Fujii, Junko Zenkoh, Hitomi Kawai, Yuta Kuze, Yukie Kashima, Satoi Nagasawa, Yuka Nakamura, Motohiro Kojima, Katsuya Tsuchihara, Masahide Seki, Akinori Kanai, Daisuke Matsubara, Takashi Kohno, Masayuki Noguchi, Akihiro Nakaya, Masahiro Tsuboi, Genichiro Ishii, Yutaka Suzuki*, Ayako Suzuki*

*To whom correspondence should be addressed;
Yutaka Suzuki; Tel.: +81 4 7136 4076; Email: ysuzuki@edu.k.u-tokyo.ac.jp
Ayako Suzuki; Tel.: +81 4 7136 4076; Email: asuzuki@edu.k.u-tokyo.ac.jp

Contents:
Supplementary Figures S1–S17 (p. 2–91)
Supplementary Tables S1–S8 (p. 92–103)
Supplementary References (p. 104)

# Supplementary Figures

*Supplementary Figure S1*

**a** LUAD No.1

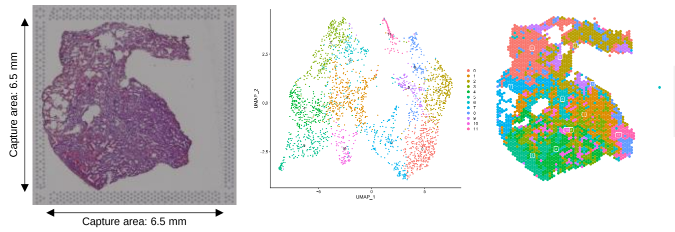


| Cluster | Annotation |
|---|---|
| 0 | Tumor cell |
| 1 | Tumor cell (*AGR3*, *CLDN6*, *KRT8*) |
| 2 | Epithelial cell, endothelial cell (*AGER*, *VWF*) |
| 3 | Fibroblast (*COL3A1*, *LUM*) |
| 4 | Tumor cell (*SCGB3A1*) |
| 5 | Plasma cell (*IGLC2*, *IGHG4*, *IGKC*) |
| 6 | No tissue, others |
| 7 | No tissue, epithelial cell (*SFTPC*) |
| 8 | Macrophage (FABP4, MARCO) |
| 9 | SMC, endothelial cell (*TAGLN*, *ACTA2*) |
| 10 | Macrophage (*APOE*, *SPP1*) |
| 11 | Tumor cell (*CEACAM6*, *CRABP2*) |

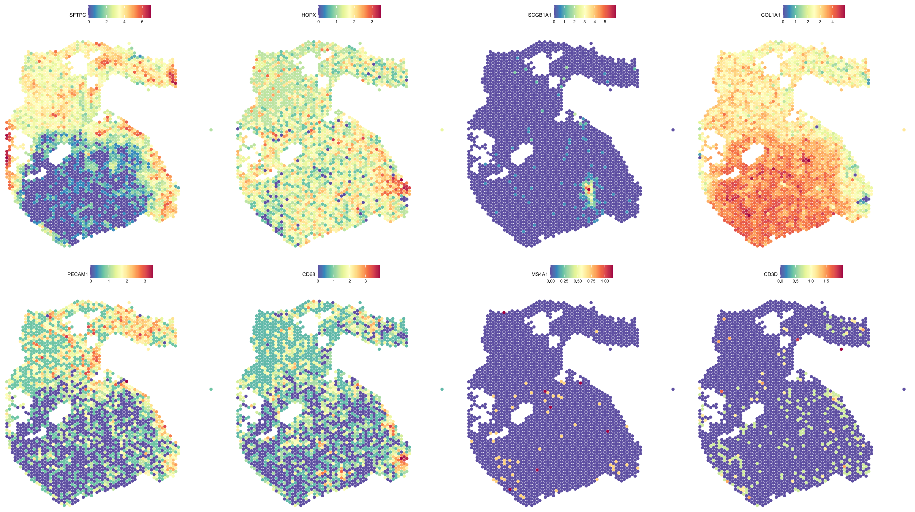


**b** LUAD No.2

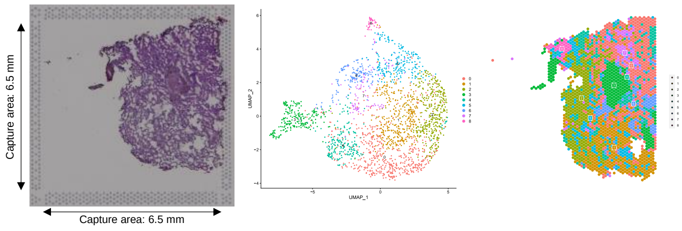


| Cluster | Annotation |
|---|---|
| 0 | Tumor cell |
| 1 | Tumor cell |
| 2 | Tumor cell |
| 3 | Macrophage (*LYZ*, *APOC1*, *APOE*) |
| 4 | Immune cell (*IGHG3*, *IGKC*, *IGHG1*) |
| 5 | No tissue |
| 6 | No tissue |
| 7 | Others |
| 8 | Tumor cell with immune response (*DDIT4*, *WARS*, *IFI6*) |

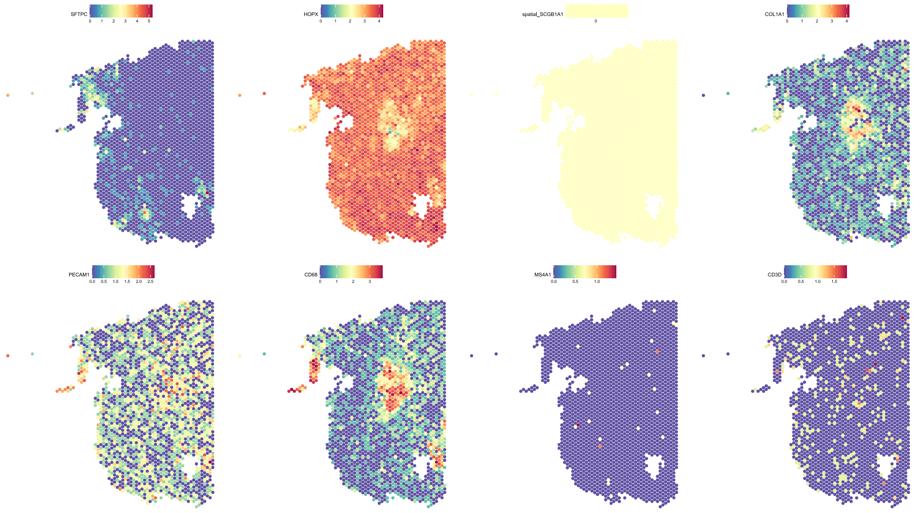

**c** <u>*LUAD No.3*</u>

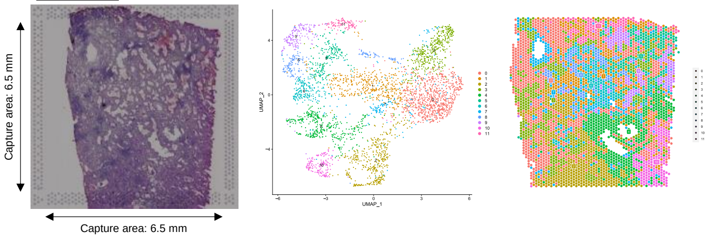


| Cluster | Annotation |
|---|---|
| 0 | Immune cell (*IGLC2*, *IGHG1*) |
| 1 | Fibroblast (*SPARC*, *COL4A1*) |
| 2 | Tumor cell (*CNMD*, *LINC00342*, *TNC*) |
| 3 | B cell, T cell (*CCL21*, *MS4A1*) |
| 4 | Tumor cell (*SCGB3A1*) |
| 5 | Tumor cell (*SFTPA2*, *SFTPA1*) |
| 6 | Tumor cell (*SCGB3A2*) |
| 7 | SMC, endothelial cell |
| 8 | Macrophage (*SPP1*) |
| 9 | No tissue |
| 10 | Tumor cell (*AZGP1*, *CNMD, TNC*) |
| 11 | Tumor cell (*AGR2*, *LPCAT1, CXCL14*) |

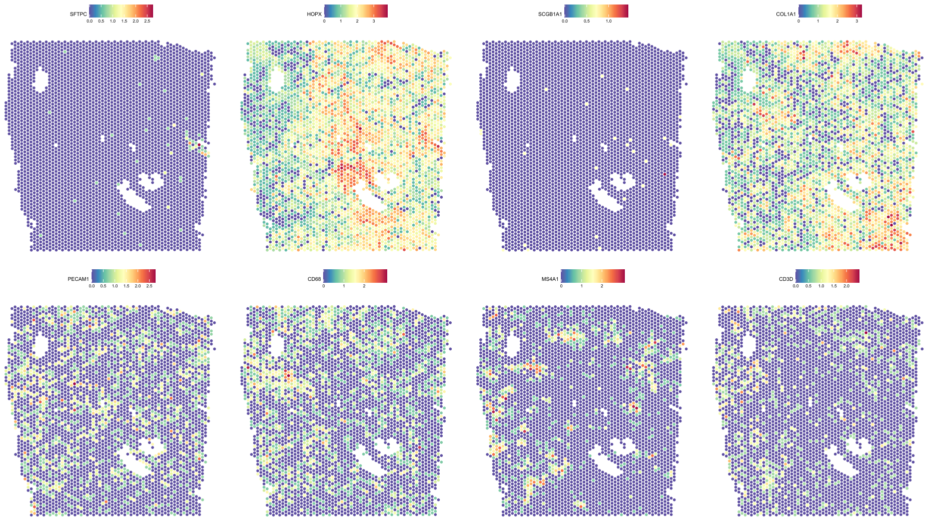


**d** <u>*LUAD No.4*</u>

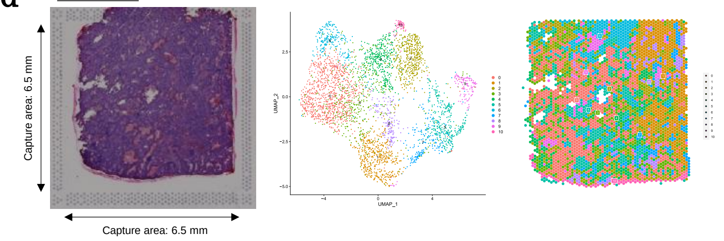


| Cluster | Annotation |
|---|---|
| 0 | Tumor cell |
| 1 | Tumor cell (*SCGB3A2*) |
| 2 | Immune cell (*IGHA1*, *IGHG4*, *IGHG1*) |
| 3 | Tumor cell |
| 4 | Fibroblast (*A2M*, *LUM*) |
| 5 | No tissue |
| 6 | Tumor cell (*SPINK1, MIA*) |
| 7 | No tissue |
| 8 | Tumor cell (*SFTPC*) |
| 9 | Macrophage (*SPP1*, *APOC1*) |
| 10 | Immune cell (*IGHG4*, *IGKC, IGHA1*) |

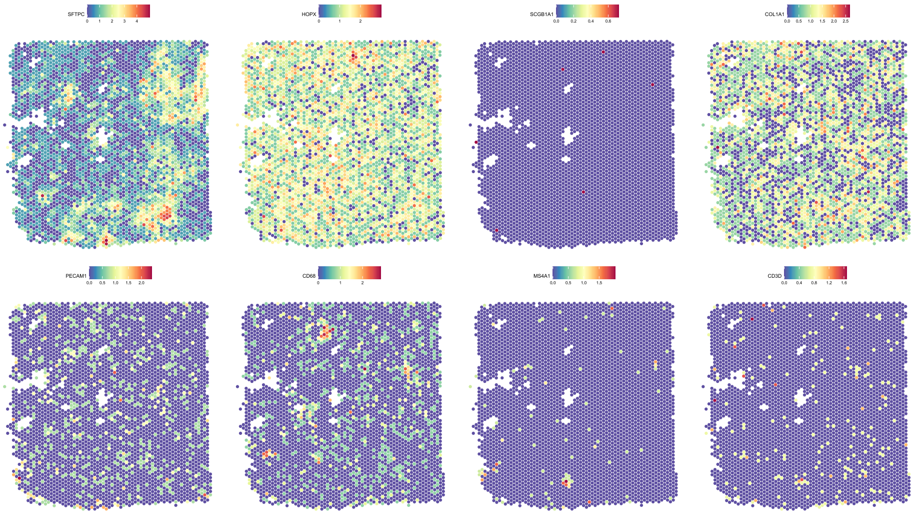

**e** LUAD No.5

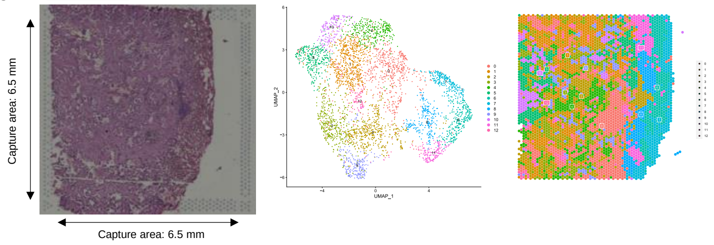


| Cluster | Annotation |
|---|---|
| 0 | Tumor cell |
| 1 | Tumor cell (*DMBT1, CXCL17*) |
| 2 | Immune cell (*IGHG4, IGHA1, IGLC2*) |
| 3 | Fibroblast (*COL1A2, COL1A1*) |
| 4 | Tumor cell (*ALB, DLK1*) |
| 5 | Tumor cell (*SFTPA1*) |
| 6 | Epithelial cell (*CAV1, AGER*) |
| 7 | No tissue |
| 8 | Tumor cell (*SCGB3A1*) |
| 9 | Macrophage (*SPP1, FN1, MSR1*) |
| 10 | Tumor cell (*VSIG1, TM4SF4*) |
| 11 | Macrophage (*APOC1, APOE, CCL18*) |
| 12 | Macrophage (SPP1, MMP12, IL1RN) |

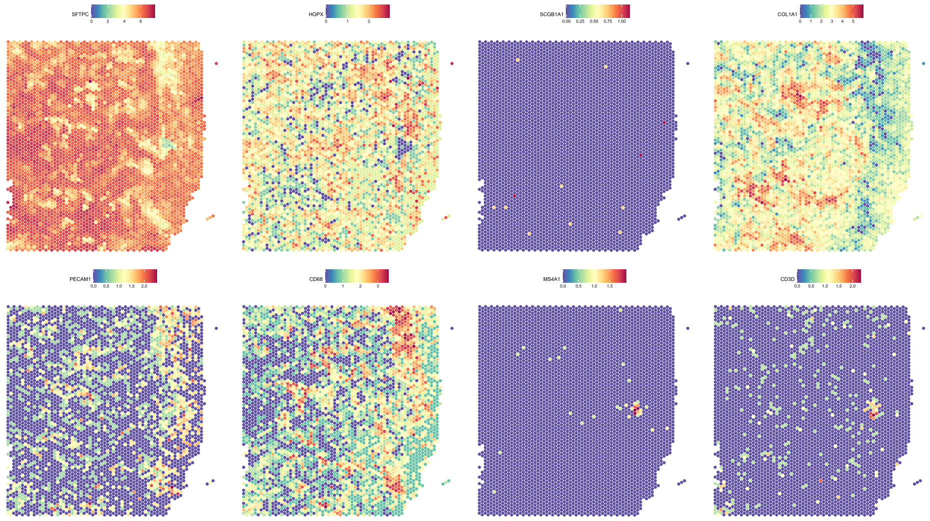


**f** LUAD No.14

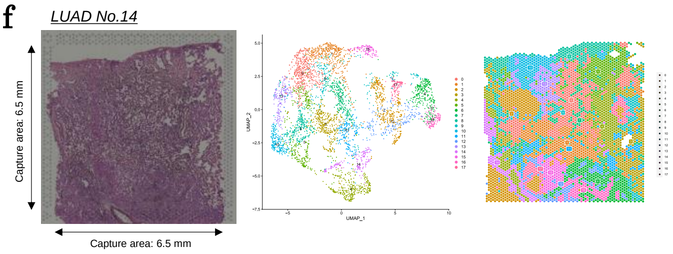


| Cluster | Annotation |
|---|---|
| 0 | Tumor cell (*SCGB3A2, SFTPA1*) |
| 1 | Tumor cell |
| 2 | Tumor cell, fibroblast (*SPP1, MMP7, COL3A1*) |
| 3 | Tumor cell |
| 4 | Bronchiolar epithelial cell (*SCGB1A1*) |
| 5 | Tumor cell, SMC (*SFTPC, TAGLN*) |
| 6 | Tumor cell (*FAM3C, ANXA1, HMGA1*) |
| 7 | Macrophage (*APOC1, CCL18, APOE*) |
| 8 | No tissue, tumor cell |
| 9 | Tumor cell |
| 10 | Immune cell (*IGHA1, IGLC2, IGHG3*) |
| 11 | Tumor cell (*SFTPC*) |
| 12 | Immune cell, fibroblast (*IGHM, COL3A1*) |
| 13 | Tumor cell (*SCGB3A2, NR4A1*) |
| 14 | Bronchiolar epithelial cell (*CLU, BPIFB1*) |
| 15 | Tumor cell with anti-inflammation (*TGFA, IL37*) |
| 16 | Tumor cell with hypoxia (*NDRG1, SLC2A1*) |
| 17 | Tumor cell (*S100A2*) |

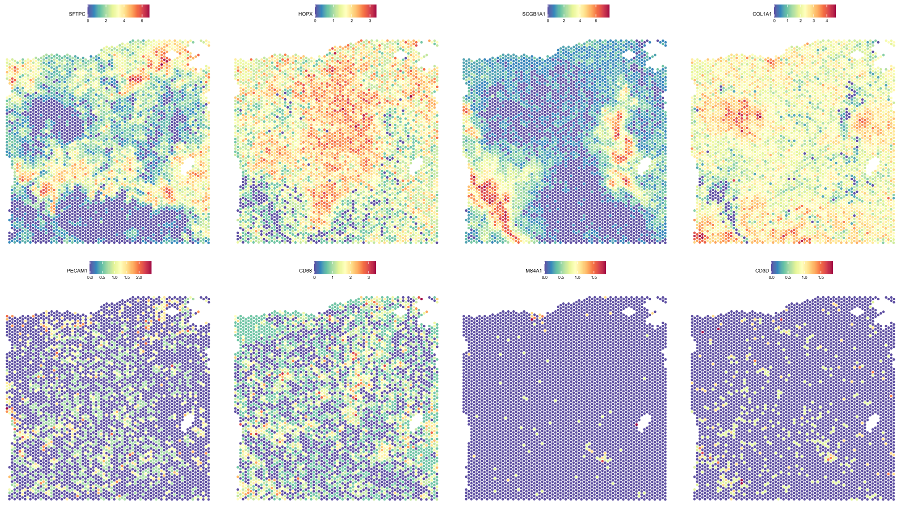

**g**

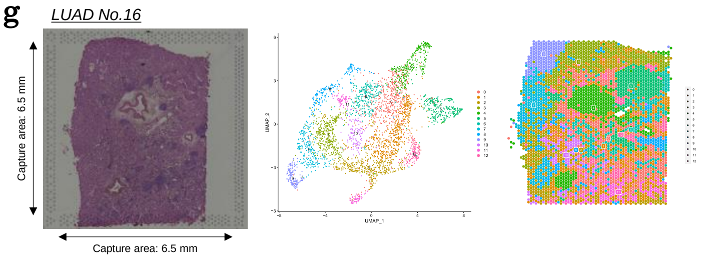


| Cluster | Annotation |
|---|---|
| 0 | Tumor cell |
| 1 | Immune cell (*IGHG1, IGKC, IGHG4*) |
| 2 | Fibroblast (*COL3A1, LUM, SPARC*) |
| 3 | Tumor cell |
| 4 | SMC (*MALAT1, MYL9, ACTA2*) |
| 5 | Bronchiolar epithelial cell (*SCGB1A1*) |
| 6 | No tissue |
| 7 | Tumor cell (*MUC21, TFPI2, S100A6*) |
| 8 | Macrophage (*APOC1, APOE, LYZ*) |
| 9 | Tumor cell (*SFTPB*) |
| 10 | Tumor cell, fibroblast (*SPP1*) |
| 11 | Tumor cell (*SFTPA1, SFTPB*) |
| 12 | T cell, B cell (*CCL19, TRAC, TRBC2*) |

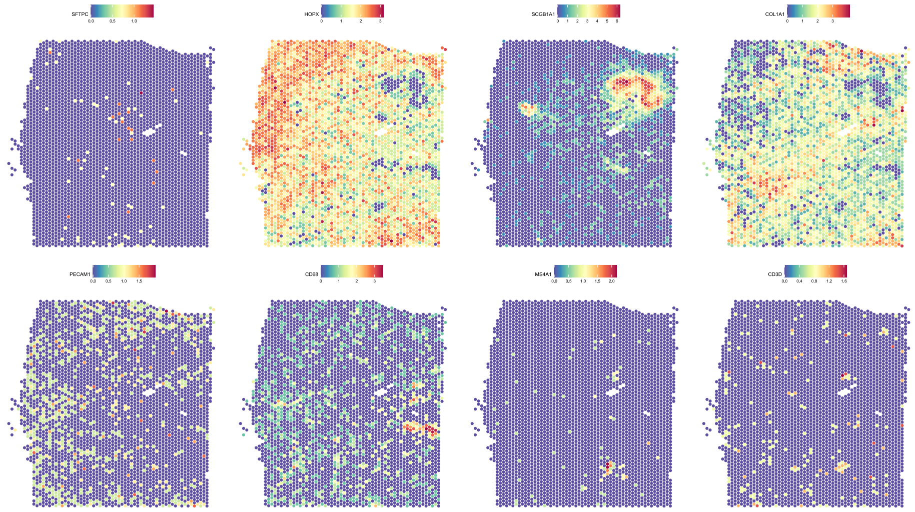


**h**

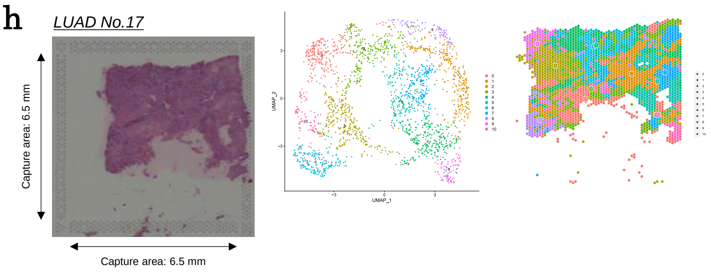


| Cluster | Annotation |
|---|---|
| 0 | Tumor cell, no tissue |
| 1 | Fibroblast (*SFRP4, LUM, THBS1*) |
| 2 | Tumor cell (*SFTPA2, UPK3BL1, LCN2*) |
| 3 | Macrophage (*SPP1, MMP12*) |
| 4 | Immune cell (*IGHG4, IGHG3*) |
| 5 | Fibroblast (*MGP, A2M*) |
| 6 | Tumor cell (*SCGB3A2, SLC34A2*) |
| 7 | Fibroblast (*COL4A1, COL4A2*) |
| 8 | Fibroblast (*MMP11, COL11A1*) |
| 9 | Macrophage (*APOC1, APOE*) |
| 10 | Tumor cell (*S100A9, S100A8*) |

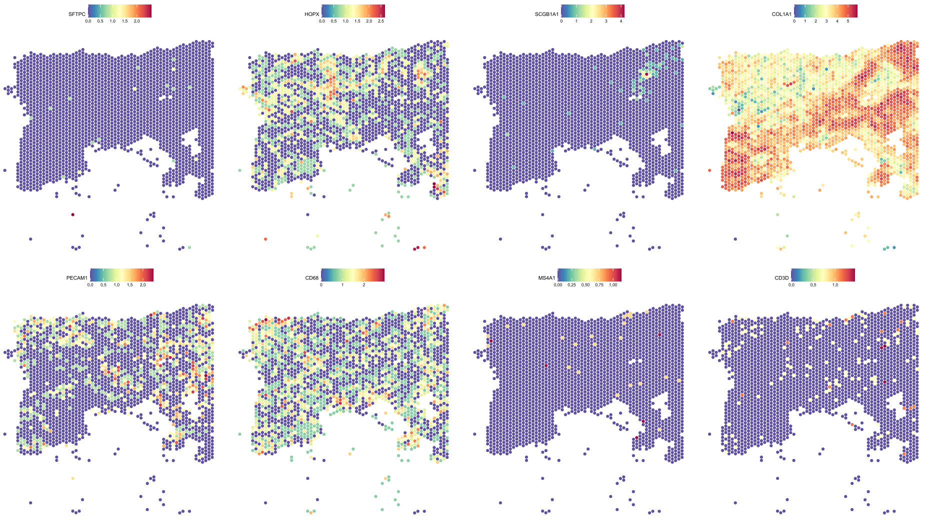

# *Supplementary Figure S1*

**i** *LUAD No.2 FFPE, A*

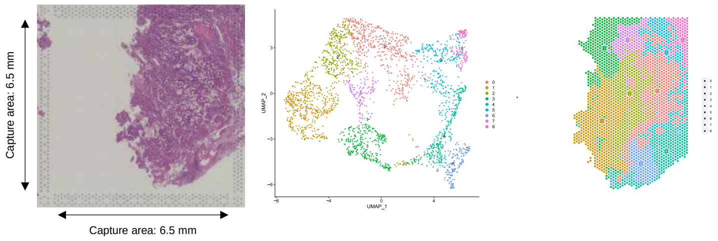


| Cluster | Annotation |
|---|---|
| 0 | Mucinous tumor cell (*MUC5AC*) |
| 1 | Tumor cell with hypoxia (*SLC2A1*) |
| 2 | Tumor cell (*NTS, CPS1*) |
| 3 | Tumor cell (*SPINK1*) |
| 4 | Immune cell (*IGHG2, JCHAIN, IGHA1*) |
| 5 | Tumor cell (*SCGB3A2, SFTA3*) |
| 6 | T cell, B cell (*LTF, CXCL13, MS4A1*) |
| 7 | Tumor cell (*MUCL3, SPINK1*) |
| 8 | Tumor cell with oxidative stress (*RNASE1, DUOXA1, DUOX1*) |

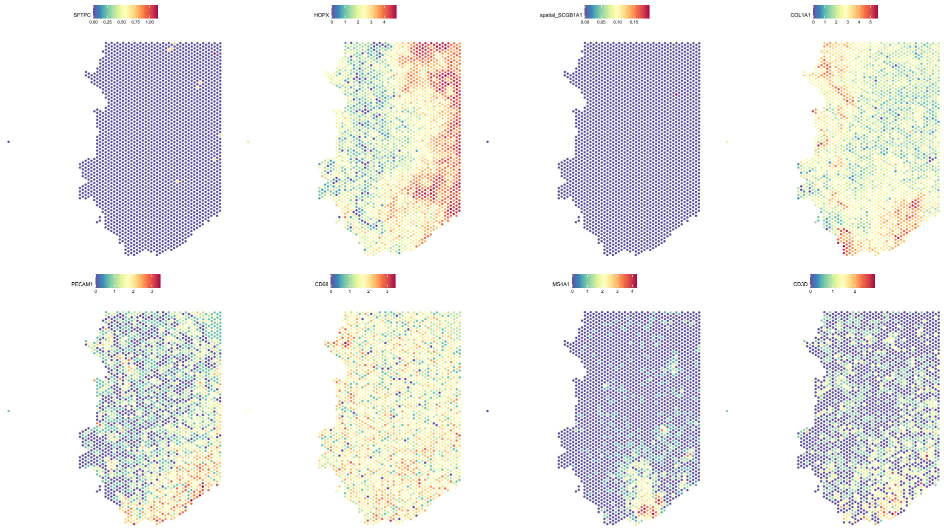


**j** *LUAD No.2 FFPE, B*

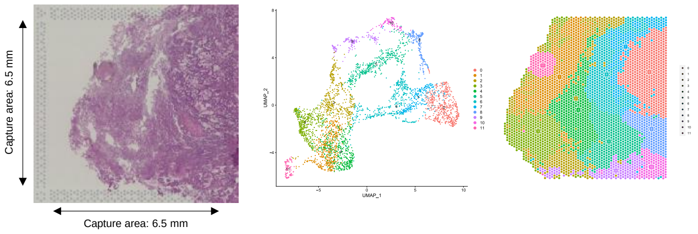


| Cluster | Annotation |
|---|---|
| 0 | Mucinous tumor cell (*NTS, MUC5AC*) |
| 1 | Tumor cell (*FGG, NAPSA*) |
| 2 | Immune cell (*IGHG2, JCHAIN*) |
| 3 | Tumor cell |
| 4 | Tumor cell |
| 5 | Tumor cell (*GKN2, WFDC5*) |
| 6 | Tumor cell with immune response |
| 7 | Tumor cell (*CACNB2, LCN2*) |
| 8 | Tumor cell (*SPINK1*) |
| 9 | Tumor cell (*FABP1, ALDOB*) |
| 10 | Tumor cell (*MMP7, CXCL14, SPINK1*) |
| 11 | Macrophage (*SPP1, ACP5, MARCO*) |

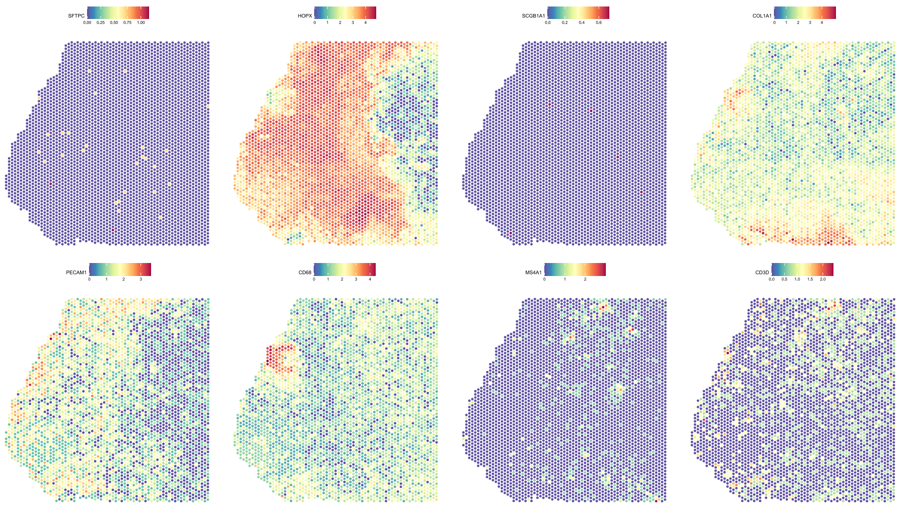

**k** LUAD No.2 FFPE, C

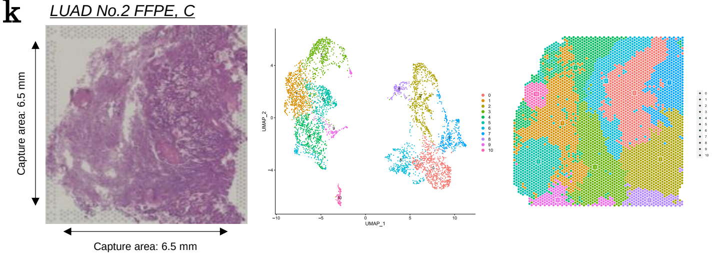


| Cluster | Annotation |
|---|---|
| 0 | Mucinous tumor cell (*NTS*, *MUC5AC*) |
| 1 | Tumor cell (*FGG*, *ABCA3*) |
| 2 | Tumor cell (*SPINK1*) |
| 3 | Tumor cell (GKN2, *RNASE1*, *WFDC5*) |
| 4 | Immune cell (*IGHA1*, *JCHAIN*) |
| 5 | Tumor cell |
| 6 | Tumor cell with immune response |
| 7 | Tumor cell with hypoxia (*SLC2A1*, TNS4) |
| 8 | Tumor cell (*F3, MUCL3, CXCL14*) |
| 9 | Immune cell, tumor cell (*FABP1*, *IGHM*) |
| 10 | Macrophage (*SPP1*, *ACP5*) |

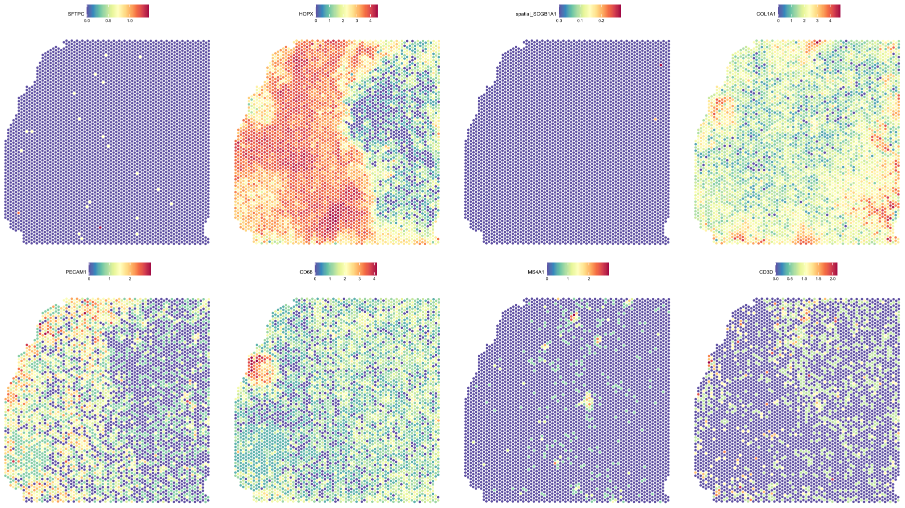


**l** LUAD No.2 FFPE, D

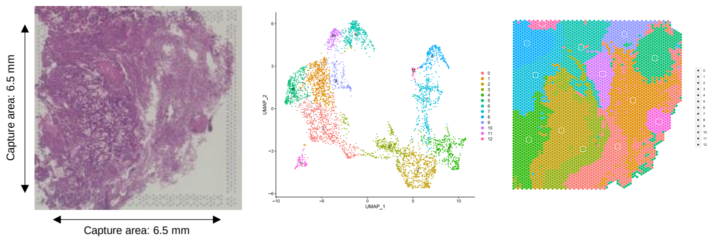


| Cluster | Annotation |
|---|---|
| 0 | Immune cell (*IGHG3*, *JCHAIN*) |
| 1 | Tumor cell (*FGG*, *ABCA3*) |
| 2 | Mucinous tumor cell (*MUC5AC*, *NTS*) |
| 3 | Tumor cell with immune response (*WARS*) |
| 4 | Tumor cell with hypoxia (*SLC2A1, TNS4*) |
| 5 | Tumor cell |
| 6 | Tumor cell (*GKN2*, *WFDC5*) |
| 7 | Tumor cell (*SPINK1*, *MMP7*) |
| 8 | Tumor cell (*MAGEA10, C15orf48, SPINK1*) |
| 9 | Tumor cell, immune cell (*FABP1, ALDOB*) |
| 10 | Tumor cell with oxidative stress (*RNASE1, DUOXA1, DUOX1*) |
| 11 | Macrophage (*SPP1, ACP5*) |
| 12 | Tumor cell (*CXCL14*, *MMP7*) |

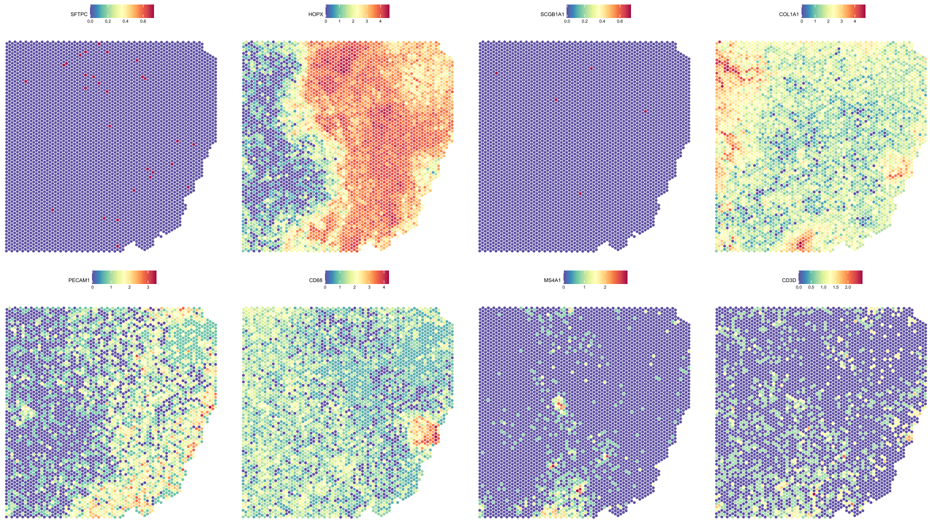

**m** LUAD No.3 FFPE, A

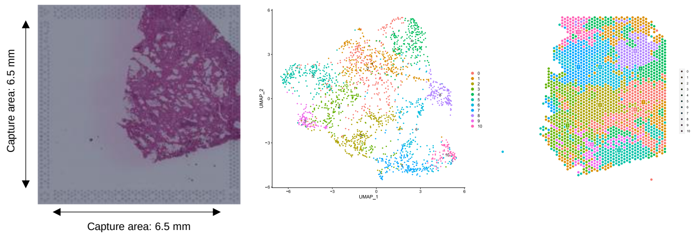


| Cluster | Annotation |
|---|---|
| 0 | Tumor cell |
| 1 | Immune cell (*IGLC1, IGKC*) |
| 2 | Tumor cell (*SCGB3A2*) |
| 3 | Tumor cell, others |
| 4 | T cell, B cell (*CXCL13, MS4A1*) |
| 5 | Tumor cell, fibroblast (*IGFBP3, FN1, CXCL14*) |
| 6 | Tumor cell |
| 7 | Tumor cell (*SCGB3A1*) |
| 8 | Tumor cell (*CNMD, ANKRD36C, TNC*) |
| 9 | Tumor cell (*LCN2, NAPSA*) |
| 10 | Tumor cell (*CNMD, ANKRD36C, FOS*) |

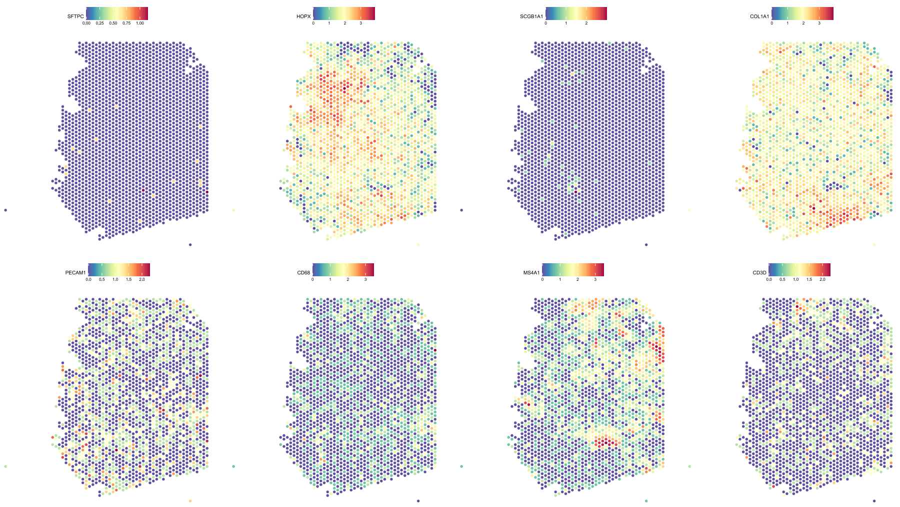


**n** LUAD No.3 FFPE, B

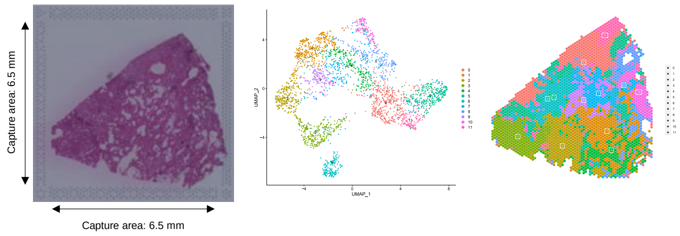


| Cluster | Annotation |
|---|---|
| 0 | Immune cell (*IGHA1, IGKC*) |
| 1 | Tumor cell (*CTSH, SLC34A2*) |
| 2 | Tumor cell (*SCGB3A1, SLPI*) |
| 3 | Tumor cell (*ANKRD36C, CNMD*) |
| 4 | Endothelial cell |
| 5 | B cell, T cell (*BANK1, MS4A1*) |
| 6 | Tumor cell (*CNMD, ANKRD36C*) |
| 7 | Tumor cell |
| 8 | Fibroblast, macrophage (*FTL, IGFBP3*) |
| 9 | Tumor cell (*SCGB3A2, SERPINA1*) |
| 10 | Tumor cell (*CXCL14, GDF15, MMP7*) |
| 11 | T cell (*IL7R, CCL19, TRBC2*) |

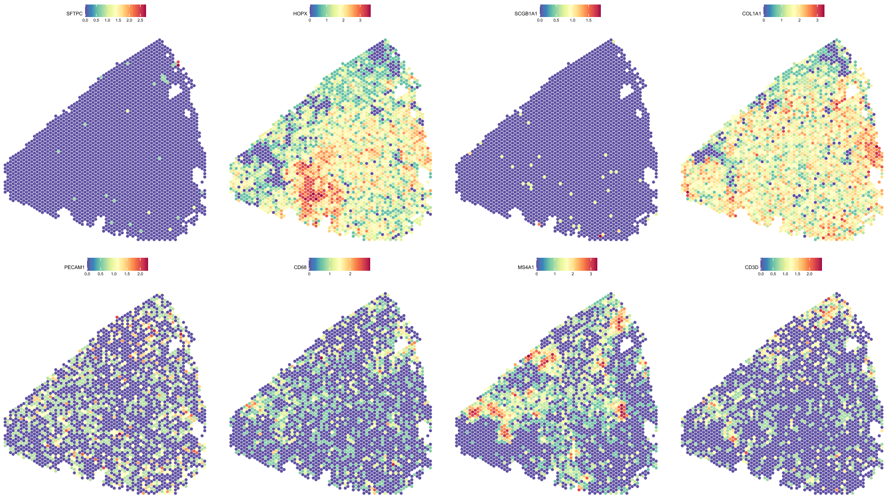

**o** LUAD No.4 FFPE, C

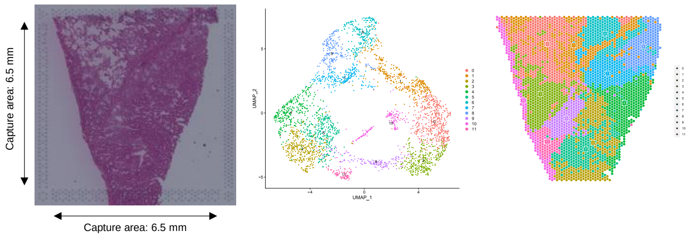


| Cluster | Annotation |
|---|---|
| 0 | Tumor cell |
| 1 | Immune cell, fibroblast (*IGHM, IGHG2*) |
| 2 | Tumor cell (*FGG, MLPH*) |
| 3 | Tumor cell (*SCGB3A2, GPAM*) |
| 4 | Tumor cell (*DHRS2, ANKRD36C*) |
| 5 | T cell (*CXCL9, CCL19*) |
| 6 | Tumor cell (*SFTPC, OLFML3*) |
| 7 | Tumor cell (*SPINK1, AZGP1*) |
| 8 | Tumor cell (*SPP1, VEGFA*) |
| 9 | Tumor cell |
| 10 | Pleura, tumor cell |
| 11 | Tumor cell (*ZNF385B, PGC*) |

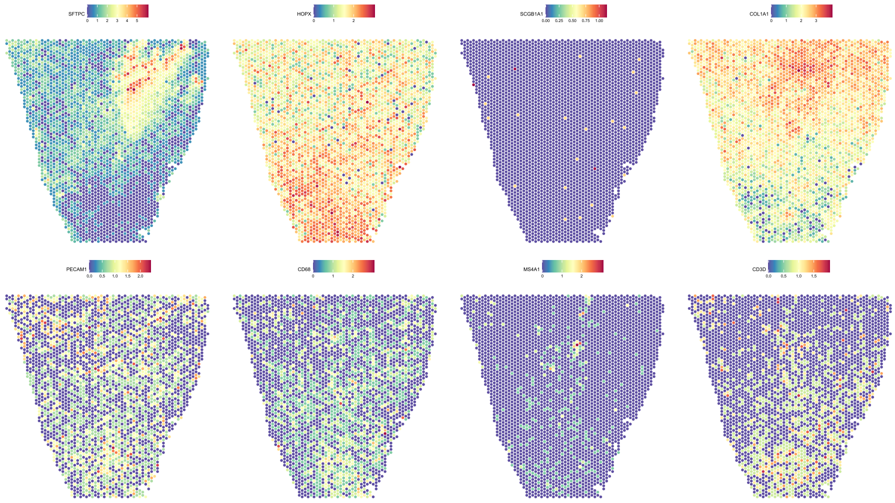


**p** LUAD No.4 FFPE, D

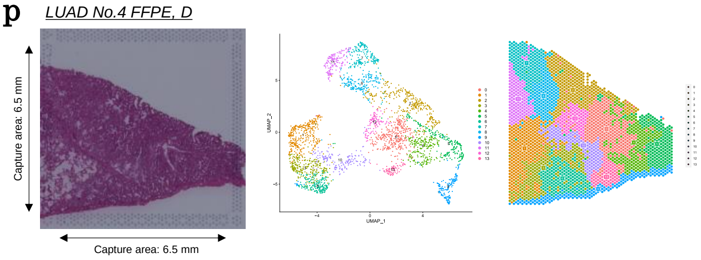


| Cluster | Annotation |
|---|---|
| 0 | T cell (*CCL19, CXCL9*) |
| 1 | Fibroblast (*COL3A1*) |
| 2 | Tumor cell (*DHRS2*) |
| 3 | Tumor cell (*GPAM*) |
| 4 | Tumor cell (*FGG, SDR16C5*) |
| 5 | Tumor cell (*MLPH, PGC*) |
| 6 | Tumor cell (*SCGB3A2, GPAM*) |
| 7 | Tumor cell (*SPP1, VEGFA*) |
| 8 | Tumor cell (*SPINK1, AZGP1*) |
| 9 | Tumor cell, pleura |
| 10 | Immune cell, fibroblast, tumor cell |
| 11 | Tumor cell (*SFTPC, OLFML3*) |
| 12 | Tumor cell (*FGB, FGG*) |
| 13 | Tumor cell (*ZNF385B*) |

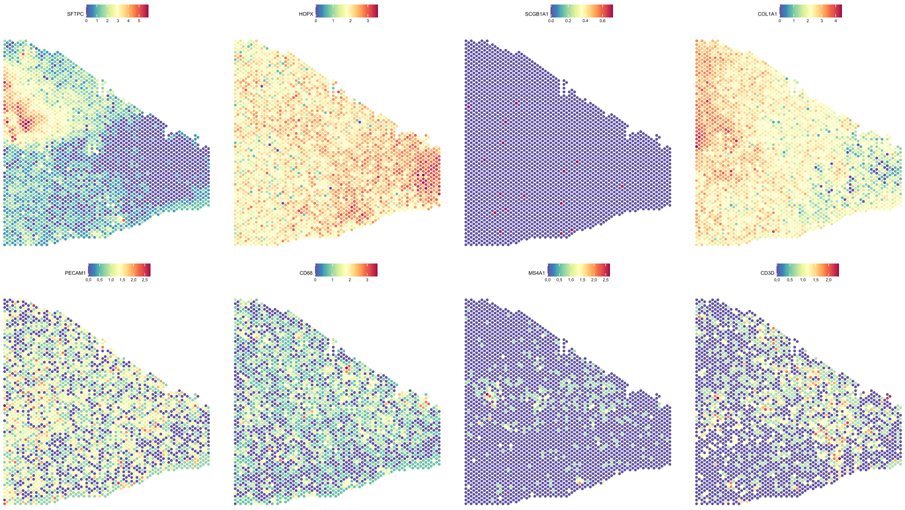

**Supplementary Figure S1 H&E images and results of clustering analysis of Visium data in eight invasive adenocarcinoma (IA) cases**

**(a–h)** Visium analysis of fresh frozen (FF) samples from LUAD cases (No. 1–5, 14, 16, and 17). Upper panel: H&E image, clustering analysis results (UMAP and spatial plots), and annotation of each cluster are presented in the right, middle, and left panels, respectively. The capture area that is surrounded by the fiducial frame in the H&E image is 6.5 mm × 6.5 mm. Lower panel: expression patterns of representative cell type marker genes are shown in the spatial plot. **(i–l)** Visium analysis of formalin-fixed paraffin-embedded (FFPE) samples from LUAD No. 2; sections A–D are provided. The result of clustering analysis in LUAD No.2 FFPE section C is shown in **Figure 2**. **(m–p)** Visium analysis of FFPE samples from LUAD No. 3 and 4; sections A–D are presented. The result of clustering analysis from LUAD No. 3 FFPE section B is also shown in **Figure 3**.

**a**

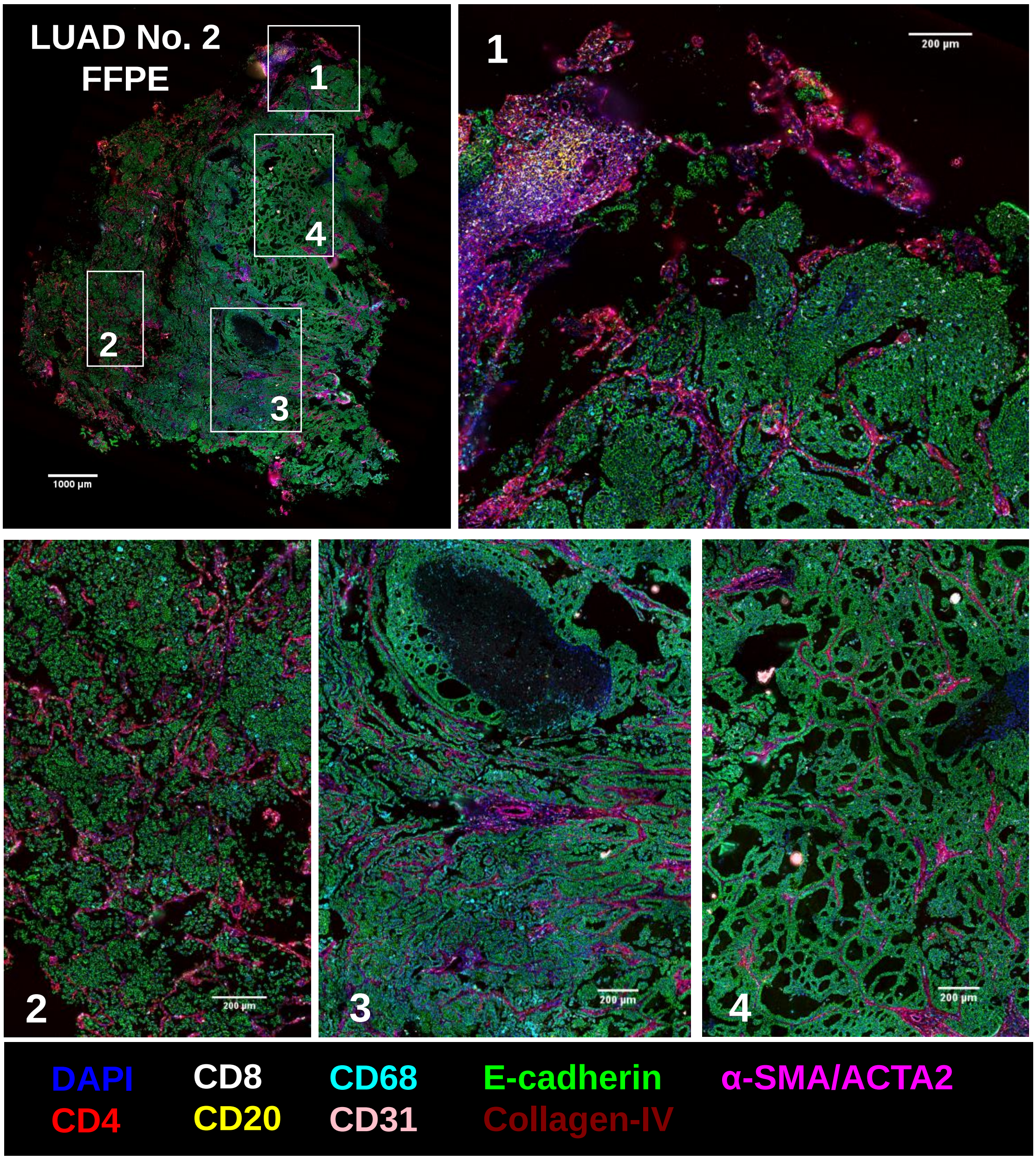
LUAD No. 2
FFPE
1
2
3
4
1000 µm
200 µm
DAPI
CD8
CD68
E-cadherin
α-SMA/ACTA2
CD4
CD20
CD31
Collagen-IV

**b**

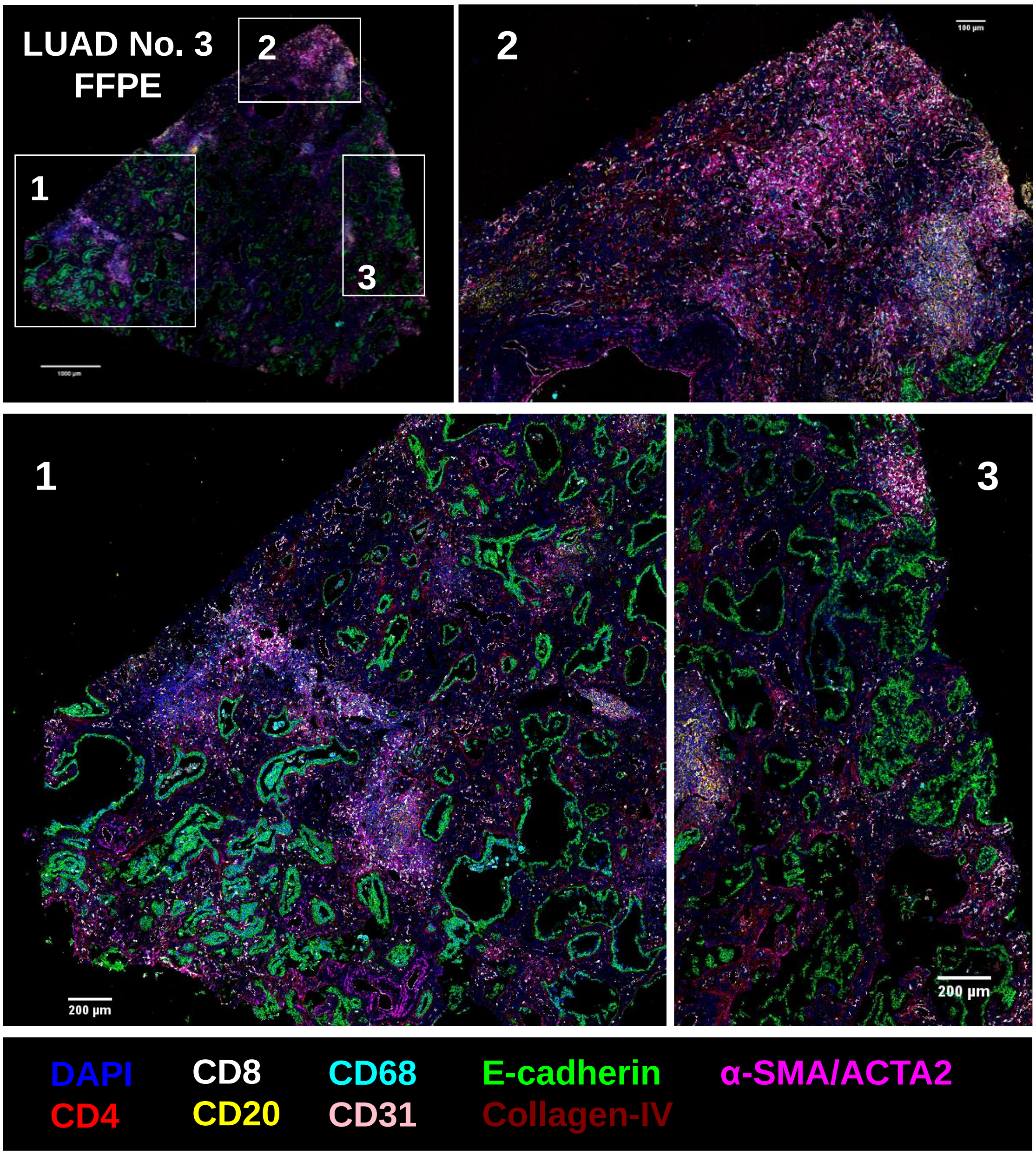

LUAD No. 3
FFPE
1
2
3
1000 µm
100 µm
200 µm
DAPI
CD8
CD68
E-cadherin
α-SMA/ACTA2
CD4
CD20
CD31
Collagen-IV

**c**

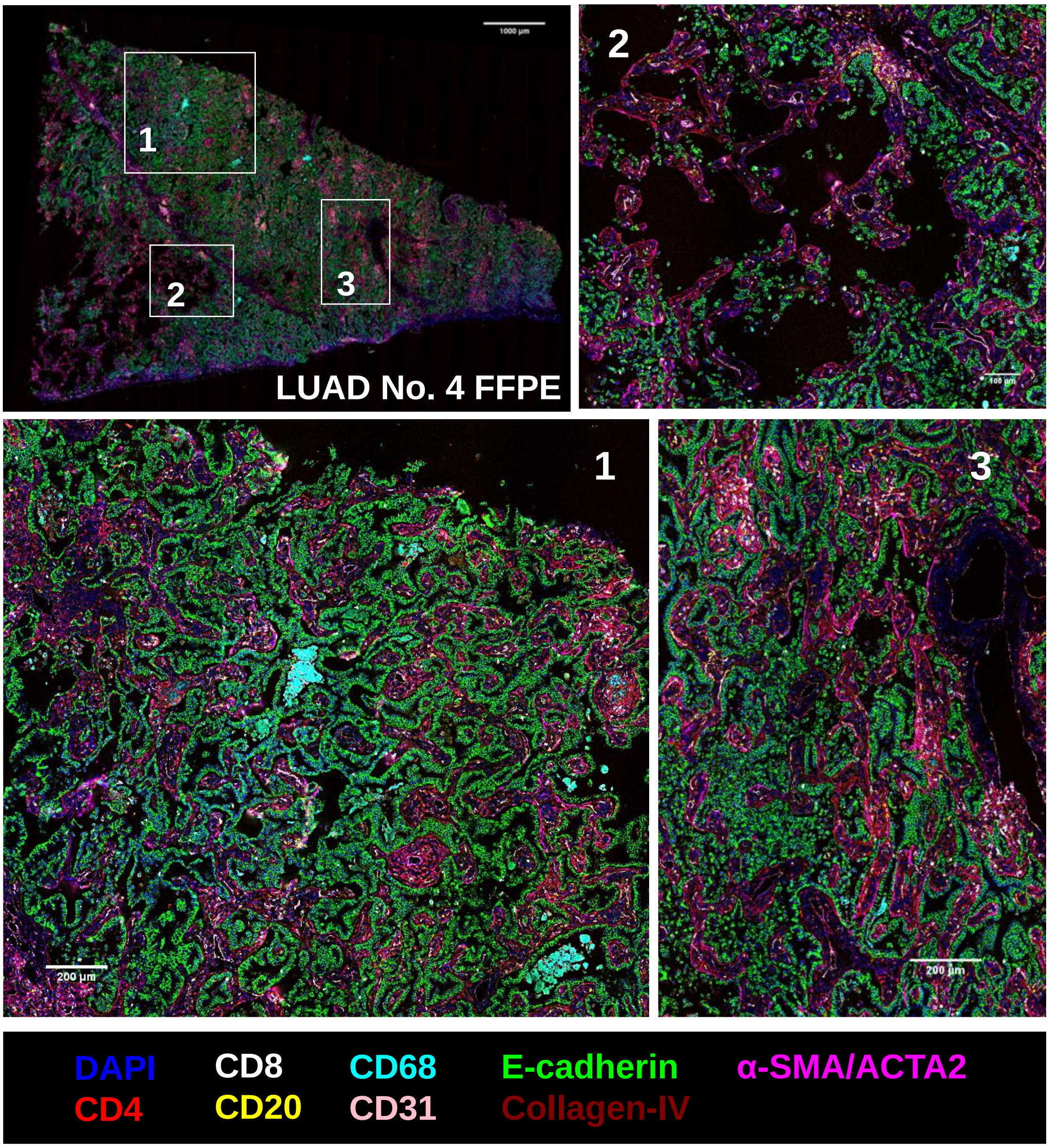
1000 μm
1
2
3
LUAD No. 4 FFPE
2
100 μm
1
200 μm
3
200 μm
DAPI
CD4
CD8
CD20
CD68
CD31
E-cadherin
Collagen-IV
α-SMA/ACTA2

**d**

LUAD No. 2 FFPE (IA)

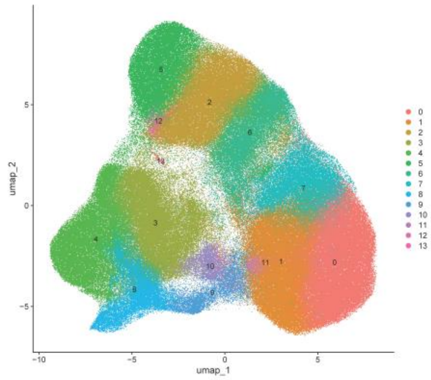


*Top 3 DEGs; log2FC > 0.5; log2FC > 2 in bold

| Cluster | Annotation | Feature* |
|---|---|---|
| 0 | Proliferating tumor cell | HLA-A, Ki67, LIF |
| 1 | Proliferating tumor cell | Ki67, HLA-A |
| 2 | Tumor cell | NKX2-1, Pan-Cytokeratin |
| 3 | Stromal cell | **α-SMA**, **CD31**, CD44 |
| 4 | Immune cell/Endothelial cell | **DC-LAMP**, **CXCL13**, **Podoplanin** |
| 5 | Tumor cell/Others | **PD-1**, Pan-Cytokeratin, NKX2-1 |
| 6 | Tumor cell | NKX2-1, Pan-Cytokeratin |
| 7 | High IDO1 cell | IDO1, HLA-A |
| 8 | T cell/B cell | **CD45RO**, **CD8**, **CD4** |
| 9 | CD8+ T cell | **CD8**, CD45RO, CD3e |
| 10 | Immune cell | FOXP3, CD56 |
| 11 | Macrophage/DC | CD11c, HLA-A, LIF |
| 12 | Tumor cell/Immune cell | **DC-LAMP**, NKX2-1, HLA-DPB1 |
| 13 | Stromal cell/Immune cell | **Podoplanin**, **DC-LAMP**, **CD8** |

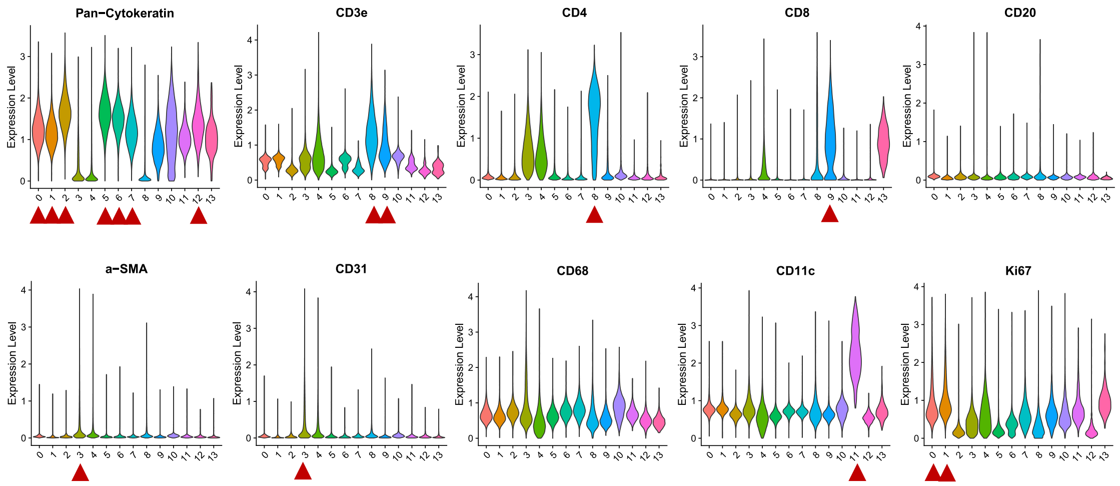


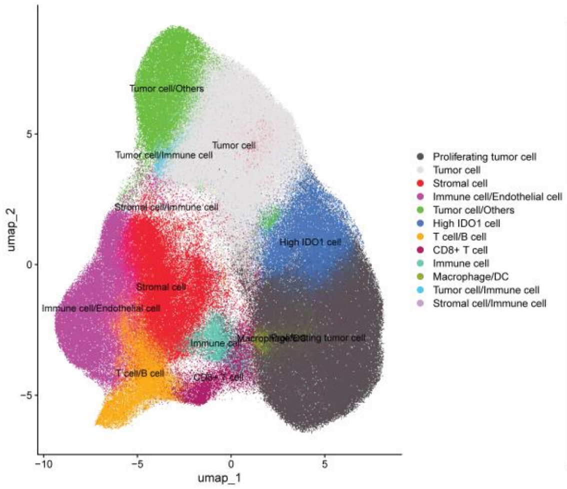


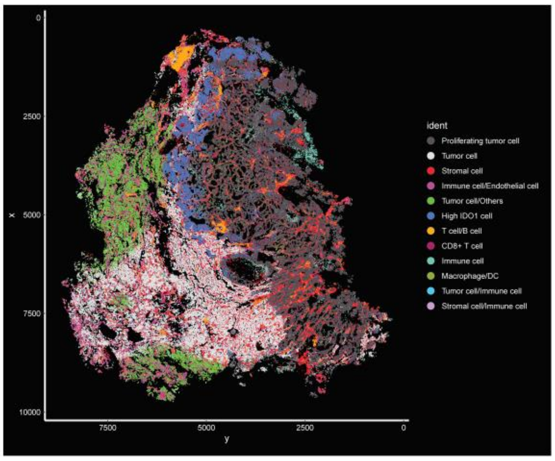

**e**

LUAD No. 3 FFPE (IA)

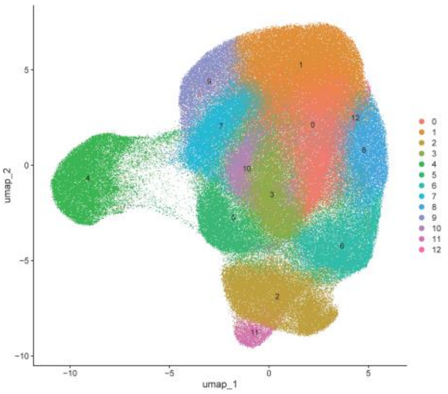


*Top 3 DEGs; log2FC > 0.5; log2FC > 2 in bold

| Cluster | Annotation | Feature* |
|---|---|---|
| 0 | Stromal cell/Immune cell | DC-LAMP, PD-L1, Ki67 |
| 1 | Stromal cell/Immune cell | --- |
| 2 | T cell | **CD8**, **CD45RO**, CD4 |
| 3 | Stromal cell/Immune cell | CD56, LIF, Podoplanin |
| 4 | Tumor cell | **Pan-Cytokeratin**, **E-Cadherin**, **NKX2-1** |
| 5 | Macrophage/DC | CD68, HLA-DPB1, CD11c |
| 6 | B cell | **CD20**, CXCL13, HLA-A |
| 7 | Fibroblast/SMC | **α-SMA**, Collagen-IV |
| 8 | B cell | **CXCL13**, CD20 |
| 9 | High Galectin-3 cell | Galectin3, LAG3, DC-LAMP |
| 10 | Endothelial cell | **CD31**, HLA-A, Podoplanin |
| 11 | Treg | **FOXP3**, **CD45RO**, **Ki67** |
| 12 | Stromal cell/Immune cell | Podoplanin, PD-L1, DC-LAMP |

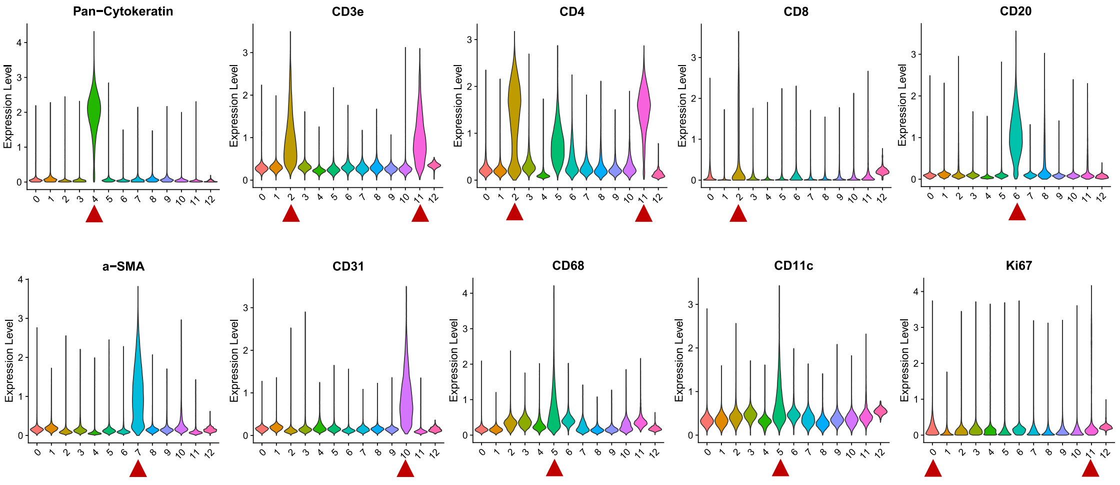


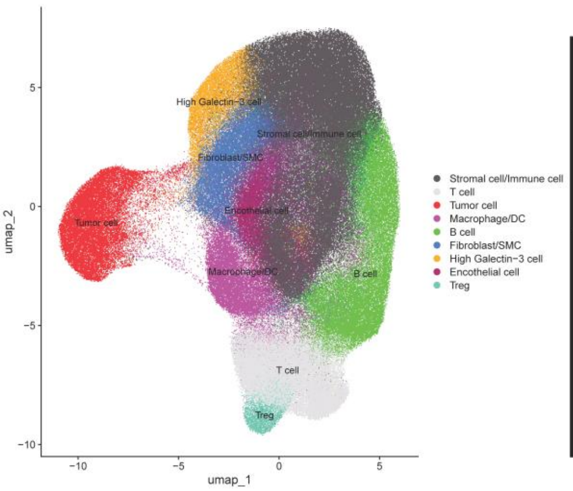


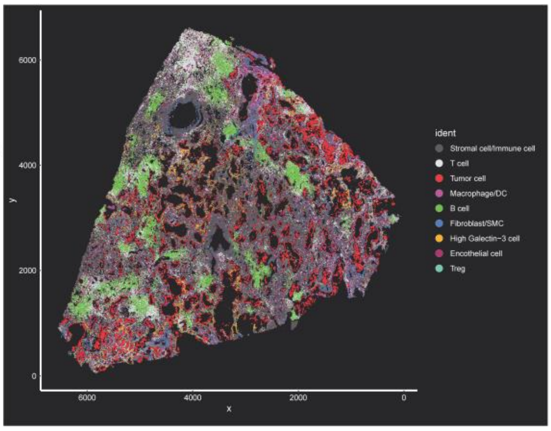

**f**

LUAD No. 4 FFPE (IA)

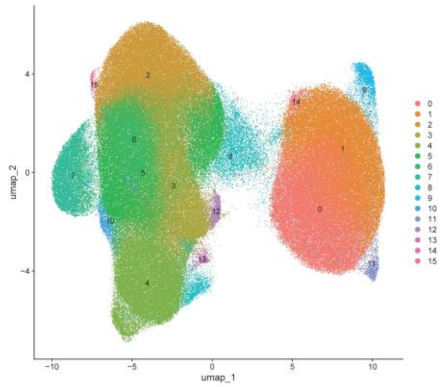


*Top 3 DEGs; log2FC > 0.5; log2FC > 2 in bold

| Cluster | Annotation | Feature* |
|---|---|---|
| 0 | Tumor cell | Pan-Cytokeratin, NKX2-1, HLA-DPB1 |
| 1 | Tumor cell | **NKX2-1**, Pan-Cytokeratin, E-Cadherin |
| 2 | Fibroblast/SMC | α-SMA |
| 3 | Immune cell | CD4, CD68, CD11c |
| 4 | T cell | **CD45RO**, **CD4**, CD3e |
| 5 | Fibroblast/SMC | **α-SMA** |
| 6 | Stromal cell/Immune cell | Podoplanin, CD8, Ki67 |
| 7 | Endothelial cell | **CD31**, α-SMA, HLA-A |
| 8 | Tumor cell/Stromal cell | α-SMA, Pan-Cytokeratin, E-Cadherin |
| 9 | Proliferating tumor cell | **Ki67**, NKX2-1, Pan-Cytokeratin |
| 10 | B cell | **CD20**, HLA-A |
| 11 | Others | **CD8**, **Podoplanin**, **DC-LAMP** |
| 12 | Macrophage/DC | **CD68**, CD31, CD11c |
| 13 | DC/Immune cell | **DC-LAMP**, HLA-A, CD4 |
| 14 | Tumor cell | TP53, NKX2-1, Pan-Cytokeratin |
| 15 | Stromal cell/Immune cell | Podoplanin, PD-L1, DC-LAMP |

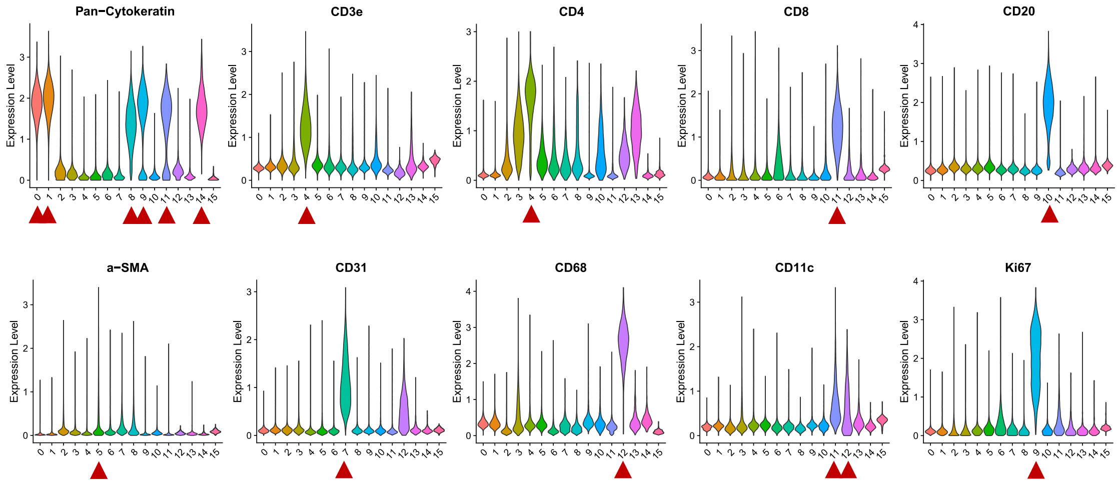


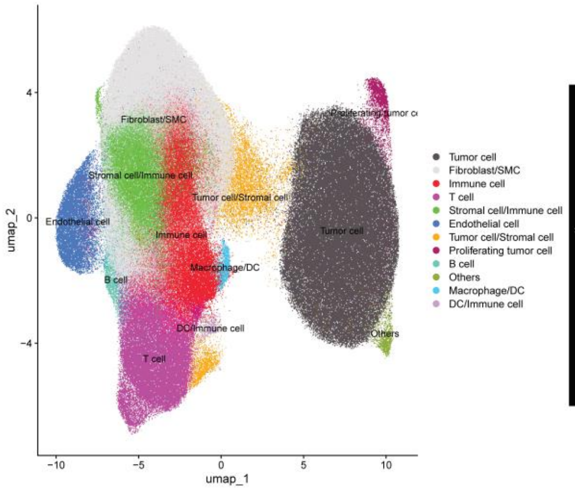


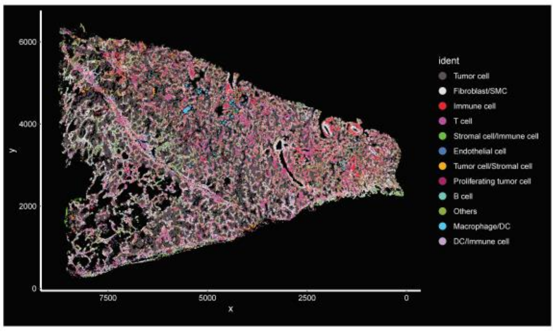

**Supplementary Figure S2 Representative images and cell phenotyping results of PhenoCycler data in IA cases**

(**a–c**) Representative images of PhenoCycler results in FFPE sections from LUAD No. 2–4. PhenoCycler signals of DAPI and eight proteins are shown. (**a**) The entire image and four ROIs are presented for LUAD No. 2 FFPE. ROI-1, a region containing lymphoid follicle and surrounding tumor cells. ROI-2, well- or moderately-differentiated tumor region. ROI-3, region with necrosis. ROI-4, mucinous tumor region. (**b**) The entire image and three ROIs are shown for LUAD No. 3 FFPE. ROI-1, a region with immune cell infiltration and surrounding tumor cells (around clusters 3, 5, and 6). ROI-2, T-cell-enriched region. ROI-3, tumor region with high expression levels of invasive genes (around cluster 10). (**c**) The entire image and three ROIs are presented for LUAD No. 4 FFPE. ROI-1, tumor region with high expression levels of invasive or hypoxic genes. ROI-2, normal-like differentiated tumor cells with lymphoid follicles and stroma. ROI-3, tumor region with stroma. (**d–f**) Clustering results and annotation of PhenoCycler data for cell phenotyping in LUAD No. 2–4 FFPE. The top panel displays a UMAP plot of the clustering results as well as a table with cell phenotype annotation for each cluster. The table includes the differentially expressed proteins of each cluster (top 3 with log2FC > 0.5; proteins with log2FC > 2 are bold). Violin plots of representative cell type markers are shown in middle panel. The UMAP and spatial plot with cell phenotyping data are shown in the bottom panel. All images and plots were flipped to match the orientation of Visium.

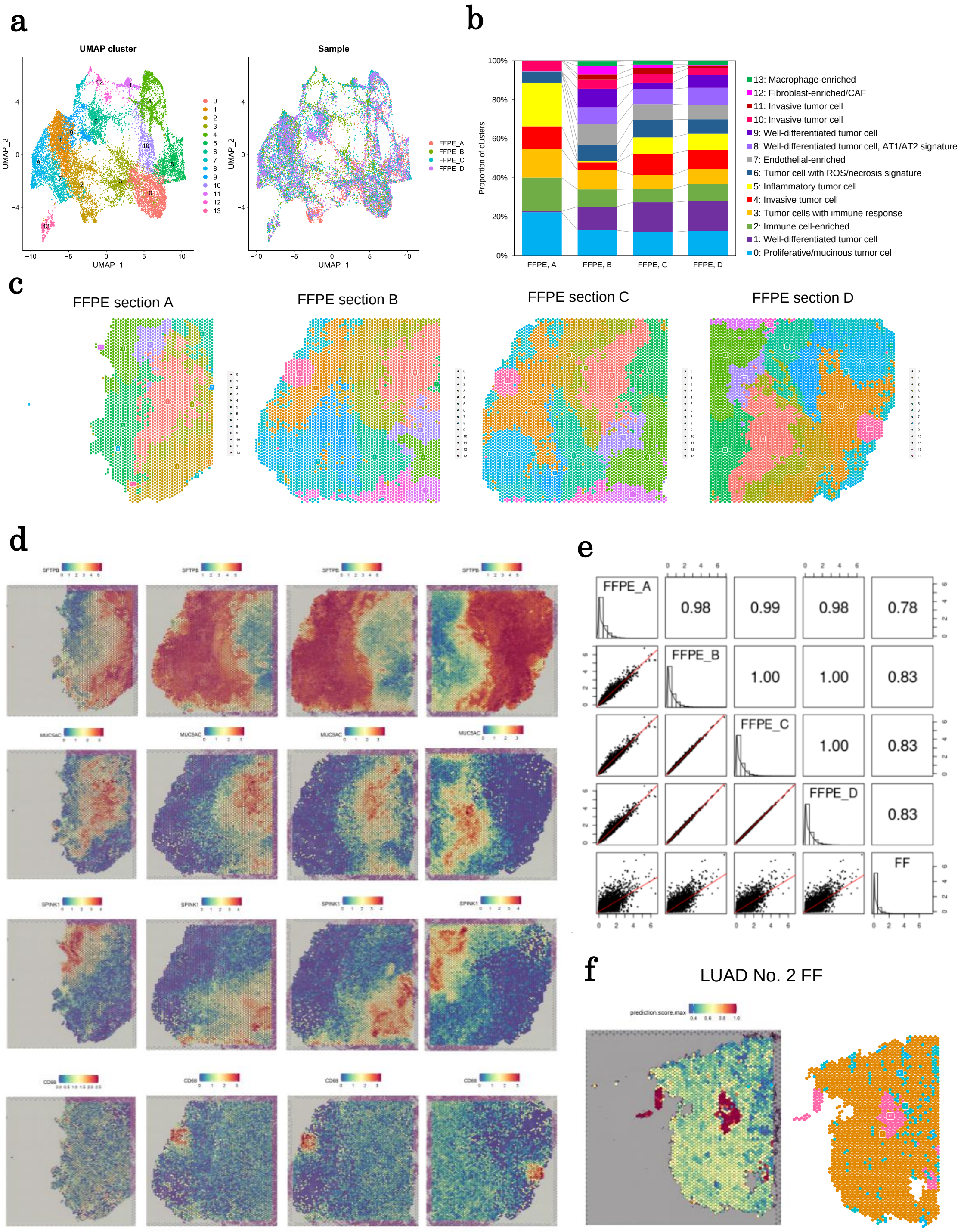
a
UMAP cluster
Sample
UMAP_1
UMAP_2
FFPE_A
FFPE_B
FFPE_C
FFPE_D
b
Proportion of clusters
FFPE, A
FFPE, B
FFPE, C
FFPE, D
13: Macrophage-enriched
12: Fibroblast-enriched/CAF
11: Invasive tumor cell
10: Invasive tumor cell
9: Well-differentiated tumor cell
8: Well-differentiated tumor cell, AT1/AT2 signature
7: Endothelial-enriched
6: Tumor cell with ROS/necrosis signature
5: Inflammatory tumor cell
4: Invasive tumor cell
3: Tumor cells with immune response
2: Immune cell-enriched
1: Well-differentiated tumor cell
0: Proliferative/mucinous tumor cel
c
FFPE section A
FFPE section B
FFPE section C
FFPE section D
d
e
FFPE_A
FFPE_B
FFPE_C
FFPE_D
FF
0.98
0.99
0.98
0.78
1.00
1.00
0.83
1.00
0.83
0.83
f
LUAD No. 2 FF
prediction.score.max

*Supplementary Figure S3*

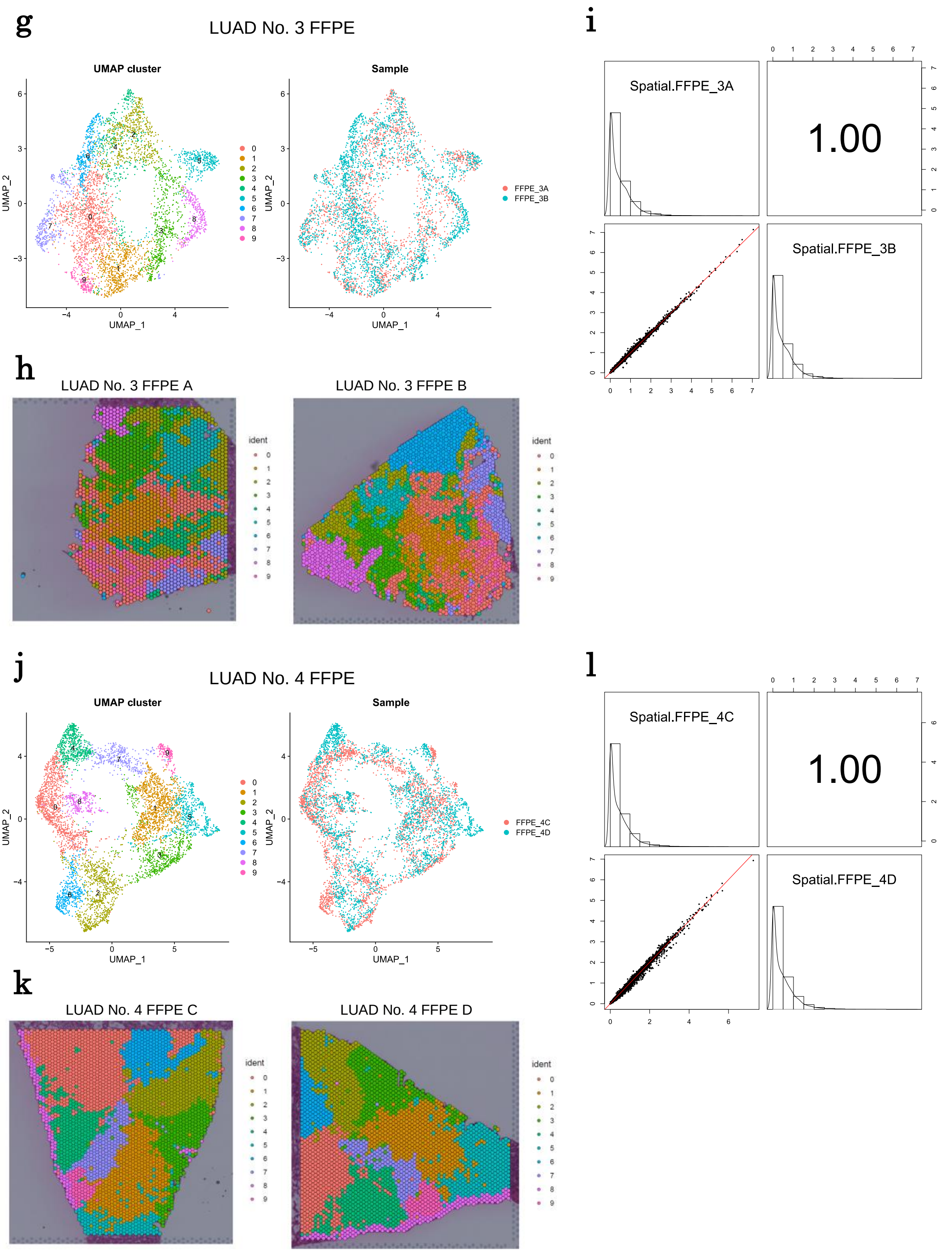

**Supplementary Figure S3 Integration and comparison of multiple sections in LUAD No. 2–4**

(**a**) The integration of Visium data from four FFPE sections of LUAD No. 2. The clusters and sections are represented by UMAP plots in the left and right panels, respectively. Four FFPE sections are serial sections. (**b**) The percentage of clusters in each FFPE section of LUAD No. 2. (**c**) Spatial plots of clusters in each FFPE section of LUAD No. 2. (**d**) Expression patterns of representative cell type markers in the spatial plots of the four FFPE sections of LUAD No. 2. *SFTPB*, well-differentiated tumor cells; *MUC5AC*, mucinous tumor cells; *SPINK1*, invasive tumor cells; and *CD68*, macrophages. (**e**) Comparison of gene expression levels between FF and FFPE specimens from LUAD No. 2. (**f**) Projecting cluster annotation onto Visium FF results from LUAD No. 2. The scores and results of the projection are presented in the upper and lower panels, respectively. (**g**) Integration of Visium data from two FFPE sections of LUAD No. 3. The clusters and sections are represented by UMAP plots in the left and right panels, respectively. (**h**) Spatial plots of clusters in each FFPE section of LUAD No. 3. (**i**) Comparison of gene expression levels between sections of LUAD No. 3. (**j**) Integration of Visium data from two FFPE sections of LUAD No. 4. The clusters and sections are represented by UMAP plots in the left and right panels, respectively. (**k**) Spatial plots of clusters in each FFPE section of LUAD No. 4. (**l**) Comparison of gene expression levels between sections of LUAD No. 4. Source data are provided as a Source Data file for **b**.

Note:

Before proceeding with the analysis, we determined the extent to which the obtained data was compatible with the FF and FFPE Visium platforms. Each platform has unique advantages. For example, the pathological images on the FFPE platform are clearer than those on the FF platform. The FF platform can identify new transcripts or isoforms, including noncoding RNAs. The FF platform is better suited for many experimental validation analyses, including extensive transcriptome, epigenome, and genome analyses on locally dissected samples.

For comparison, we chose four consecutive FFPE sections and one FF section from LUAD No. 2. The histopathological images and H&E staining patterns were clearer in FFPE than in FF sections, allowing for more precise pathological inspection. In **Supplementary Figure S4a**, several pathological features, including well- or moderately-differentiated tumor regions, necrotic regions, mucinous-rich acinar or cribriform regions, and papillary regions with CAFs, are clearly represented in FFPE sections. For

this LUAD No. 2 specimen, we evaluated the gene expression profiles. Data from each FFPE section were highly reproducible (Pearson's correlation: 0.98–1.00). A comparison of FF and FFPE results from different parts of the same specimen revealed a reasonably high correlation between the platforms (Pearson's correlation: 0.78–0.83). In addition, we investigated whether FFPE and FF data could be combined. We found that FFPE and FF data could be combined using the label transfer method. In fact, when clustering was performed using mixed data, the FF and FFPE clusters were reasonably merged. Specifically, the number of detected genes in a given sequencing depth was higher in FFPE spatial transcriptome sequencing, partly owing to the procedure's inability to capture targets other than preset mRNAs, such as noncoding RNAs and mRNA-like technical noise products. Finally, all spatial expression pattern data from representative protein-coding genes obtained through FF and FFPE Visium analyses could be treated similarly.

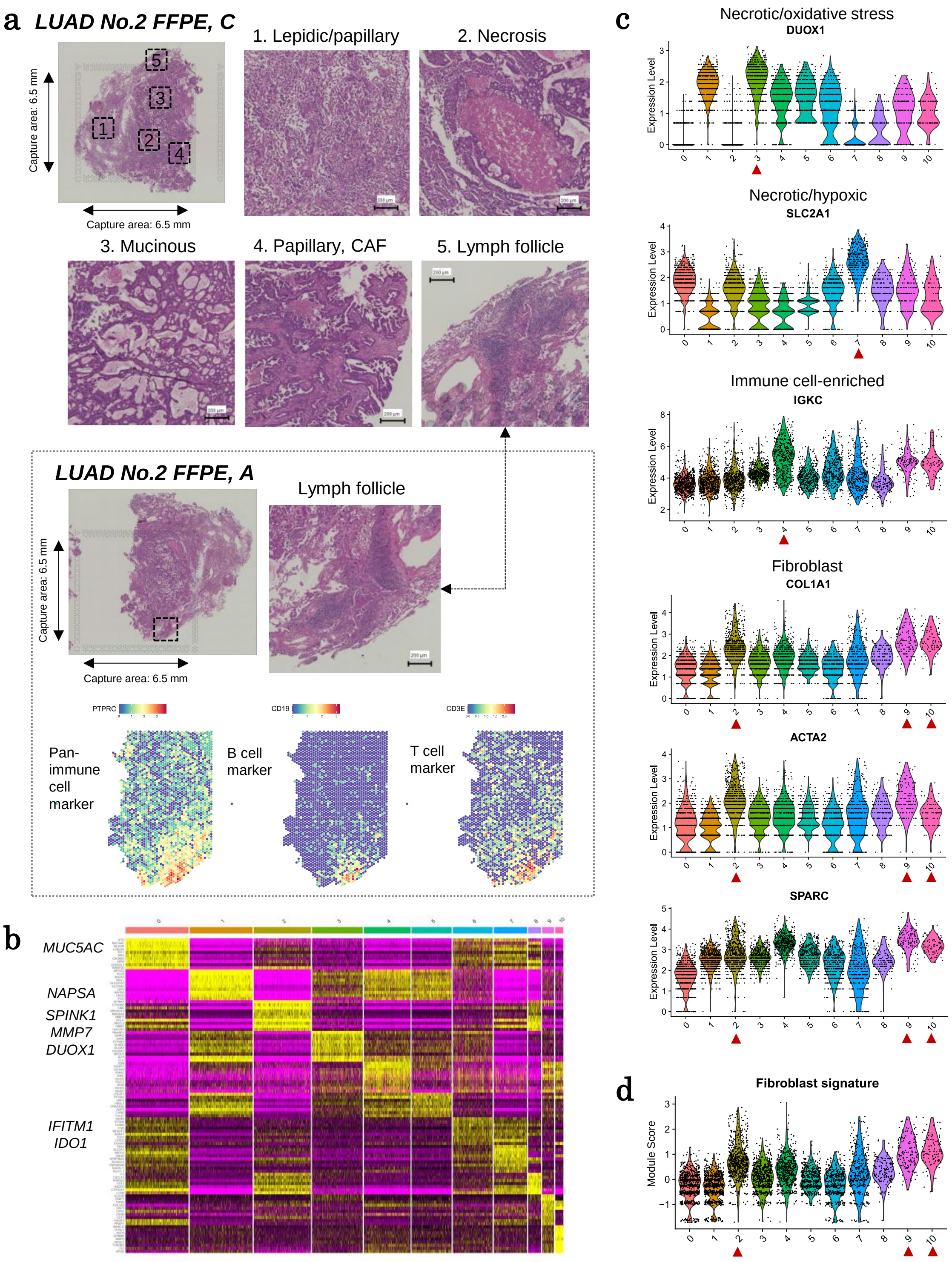
a
LUAD No.2 FFPE, C
Capture area: 6.5 mm
Capture area: 6.5 mm
1. Lepidic/papillary
2. Necrosis
3. Mucinous
4. Papillary, CAF
5. Lymph follicle
LUAD No.2 FFPE, A
Lymph follicle
Capture area: 6.5 mm
Capture area: 6.5 mm
PTPRC
CD19
CD3E
Pan-immune cell marker
B cell marker
T cell marker
b
MUC5AC
NAPSA
SPINK1
MMP7
DUOX1
IFITM1
IDO1
c
Necrotic/oxidative stress
DUOX1
Necrotic/hypoxic
SLC2A1
Immune cell-enriched
IGKC
Fibroblast
COL1A1
ACTA2
SPARC
Expression Level
d
Fibroblast signature
Module Score

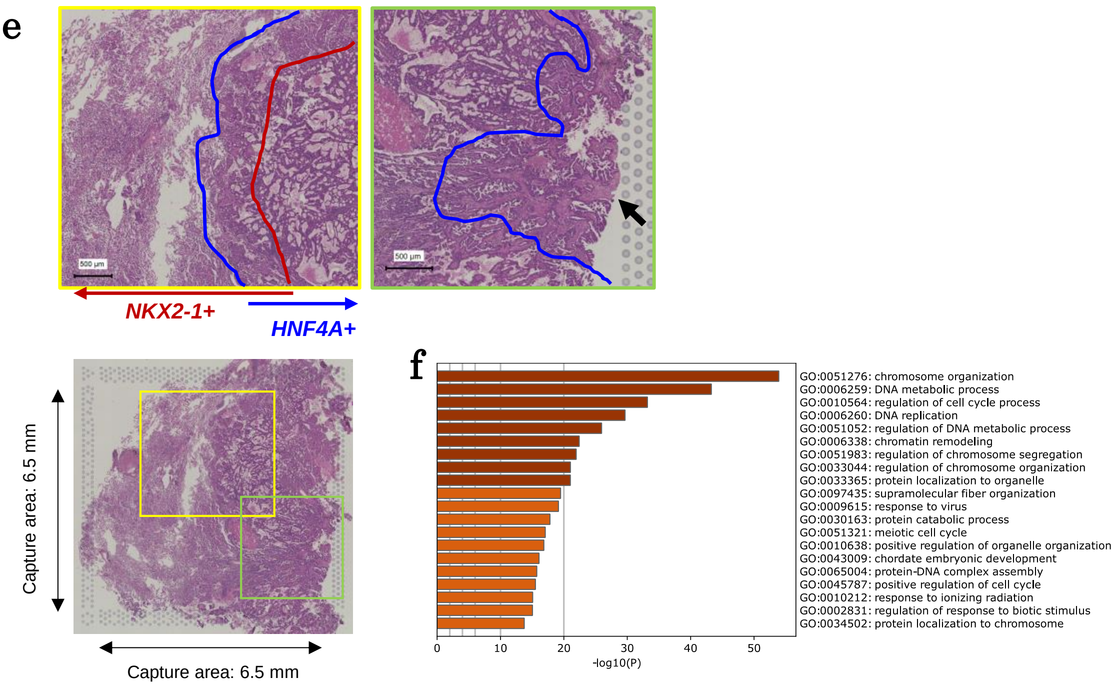


**Supplementary Figure S4 Characterization of LUAD No. 2 FFPE**

(**a**) Histological images of representative regions from LUAD No. 2 FFPE section C. The immune cell-rich region (5. Lymph Follicle) is partially out of frame in section C, so the H&E image of the adjacent section A was used, and expressions of representative immune cell markers were evaluated in this region. (**b**) Heatmap showing the expression patterns of differentially expressed genes (DEGs) in each cluster. The names of representative genes are highlighted in the margin. (**c**) Violin plots of expression levels in each cluster for representative genes. The cluster(s) with higher expressions of these genes are highlighted in red triangles in the margin. (**d**) Violin plots showing the expression signatures of three fibroblast markers. Module scores were calculated using the Seurat AddModuleScore function to show their average expression signature. (**e**) Upper left, H&E image of the boundary region between *NKX2-1*-positive well-differentiated tumor cells and *HNF4A*-positive mucinous tumor cells (around cluster 6), as defined in **Figure 2c**. Upper right, H&E image of the invasive tumor region (around cluster 3), as defined in **Figure 2g**. CAFs were enriched in this region (black arrow). Lower, the regions of the upper panels are indicated by yellow and yellow-green frames, respectively. (**f**) Gene Ontology enrichment analysis of DEGs in cluster 2. Source data are provided as a Source Data file for **f**.

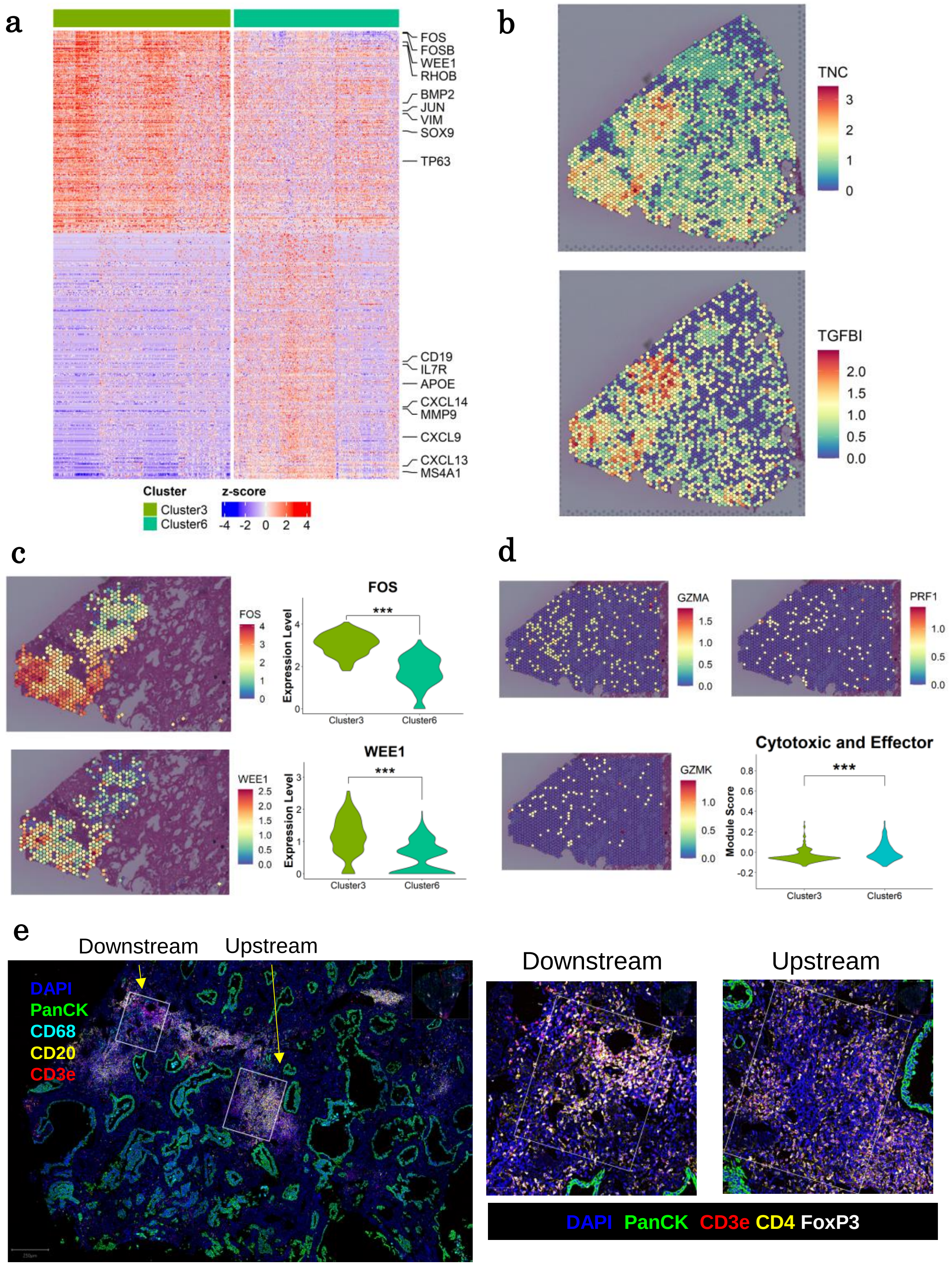
a
FOS
FOSB
WEE1
RHOB
BMP2
JUN
VIM
SOX9
TP63
CD19
IL7R
APOE
CXCL14
MMP9
CXCL9
CXCL13
MS4A1
Cluster
Cluster3
Cluster6
z-score
-4 -2 0 2 4
b
TNC
TGFBI
c
FOS
WEE1
Expression Level
Cluster3
Cluster6
***
d
GZMA
PRF1
GZMK
Cytotoxic and Effector
Module Score
e
Downstream
Upstream
DAPI
PanCK
CD68
CD20
CD3e
DAPI PanCK CD3e CD4 FoxP3

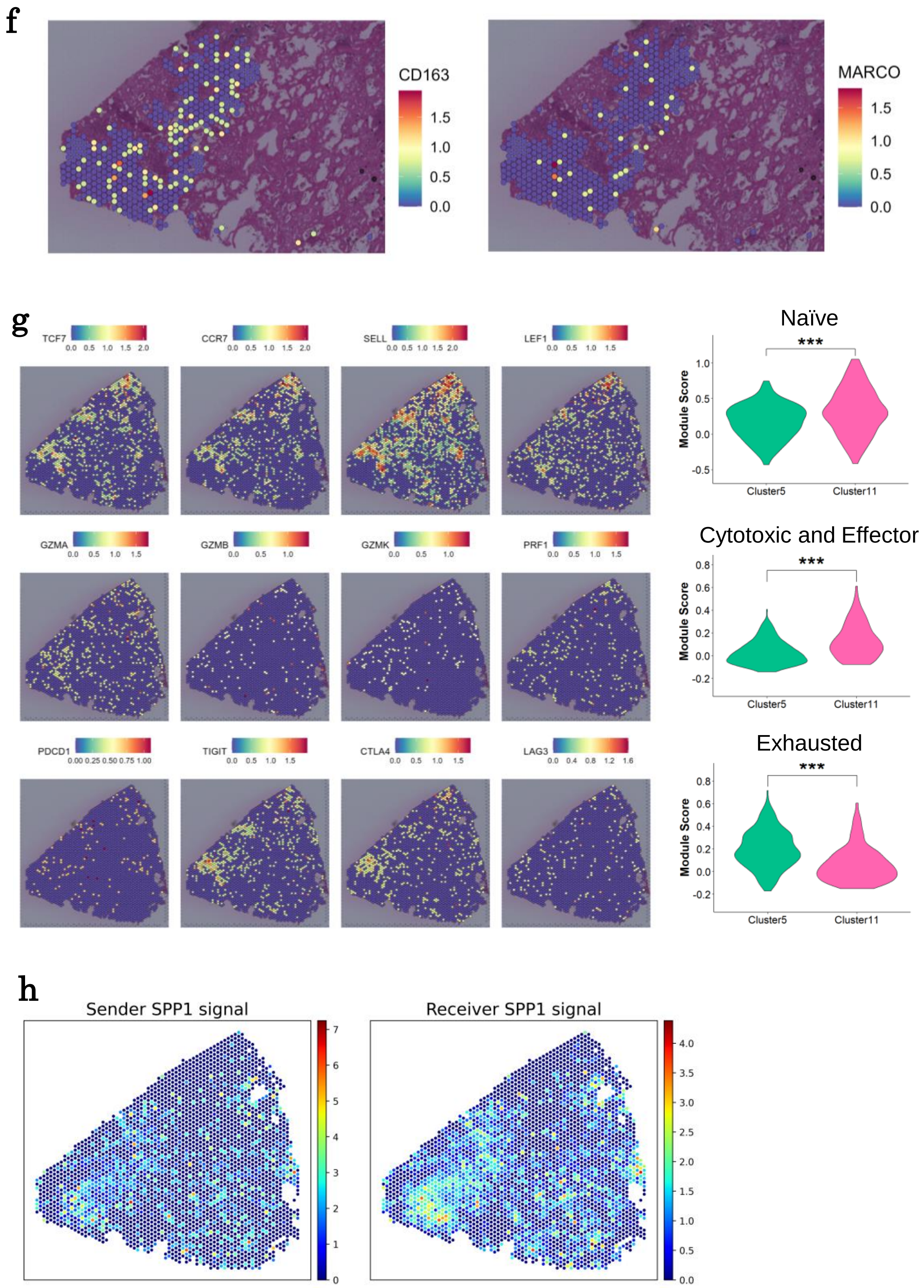
f
CD163
MARCO
g
TCF7
CCR7
SELL
LEF1
GZMA
GZMB
GZMK
PRF1
PDCD1
TIGIT
CTLA4
LAG3
Naïve
Cytotoxic and Effector
Exhausted
Module Score
Cluster5
Cluster11
***
h
Sender SPP1 signal
Receiver SPP1 signal

**Supplementary Figure S5 Characterization of LUAD No. 3 FFPE**

(**a**) DEGs between clusters 3 and 6 of LUAD No. 3 FFPE section B. Representative DEGs associated with EMT and immune cell markers are shown in the margin. The heatmap was visualized by ComplexHeatmap[1]. (**b**) The expression patterns of *TNC* and *TGFBI* are shown in the left and right panels, respectively, for LUAD No. 3 FFPE section B. (**c**) A comparison of the expression patterns of proliferation markers *FOS* and *WEE1* in clusters 3 and 6. Spatial and violin plots are shown on the left and right, respectively. *P*-values were determined using the Wilcoxon rank sum test. ***$P <$ 1e−4. (**d**) Comparison of the expression patterns of the cytotoxic and effector immune cell markers *GZMA*, *GZMK*, and *PRF1* between clusters 3 and 6. Spatial and violin plots are shown on the left and right, respectively. *P*-values were determined using the Wilcoxon rank sum test. ***$P <$ 1e−4. (**e**) Result of multiplexed immunostaining PhenoCycler with DAPI and representative cell markers. The region surrounding clusters 3, 5, and 6 is shown in the left panel. For cluster 5 with flow of immune cell infiltration, the right panels zoom in on the two subregions (downstream and upstream). (**f**) Expression of *CD163* (M2 macrophages) and *MARCO* (alveolar macrophages) in clusters 3 and 6. (**g**) Spatial expression patterns of naïve, cytotoxic or effector, and exhausted immune cell markers. The left and right panels show spatial and violin plots for comparing clusters 5 and 11. (**h**) An example of the results of ligand–receptor interaction analysis. The sender and receiver signals for SPP1 signaling (database: CellChat[2]) are shown.

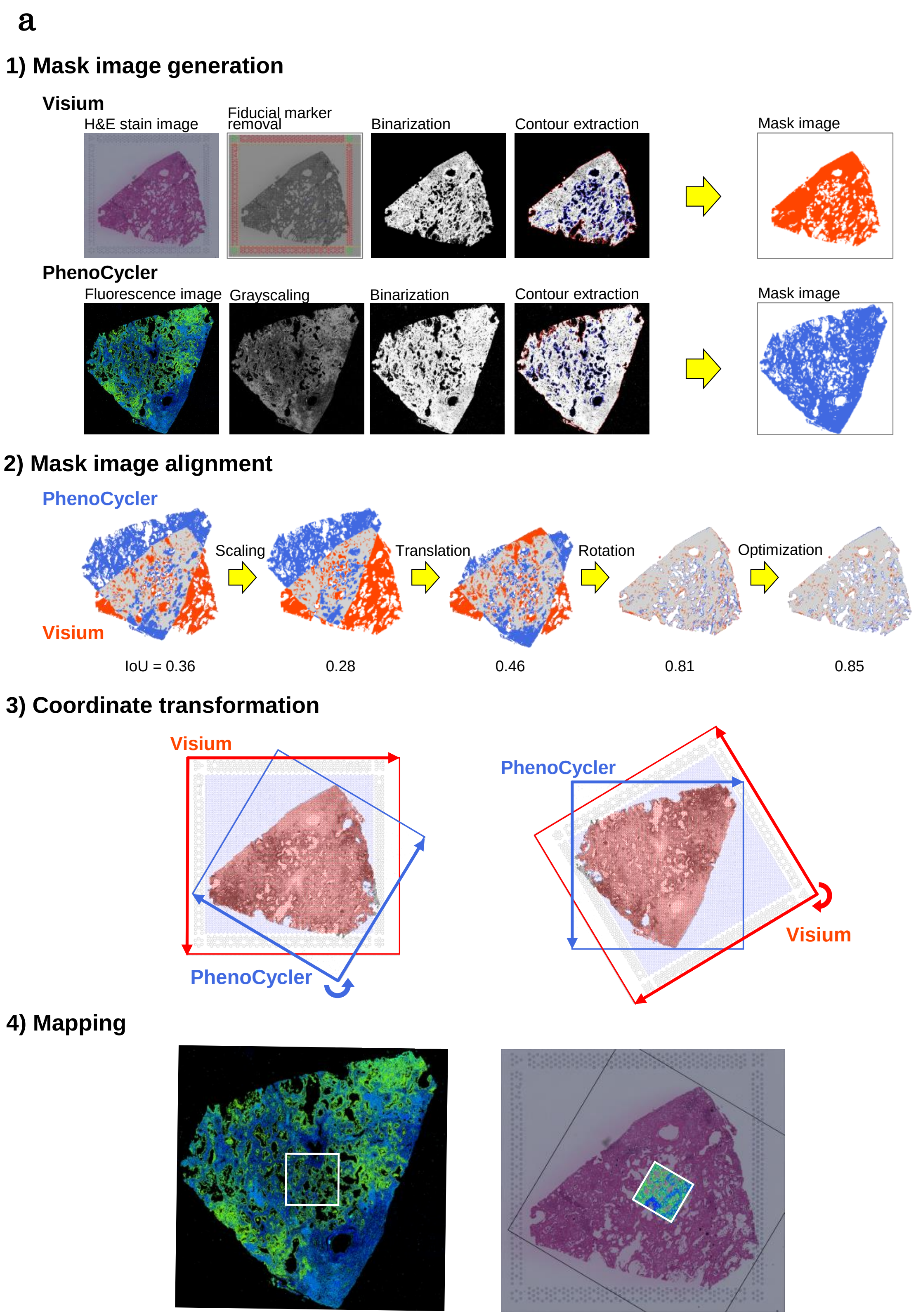
a
1) Mask image generation
Visium
H&E stain image
Fiducial marker removal
Binarization
Contour extraction
Mask image
PhenoCycler
Fluorescence image
Grayscaling
Binarization
Contour extraction
Mask image
2) Mask image alignment
PhenoCycler
Visium
Scaling
Translation
Rotation
Optimization
IoU = 0.36
0.28
0.46
0.81
0.85
3) Coordinate transformation
Visium
PhenoCycler
PhenoCycler
Visium
4) Mapping

**b**

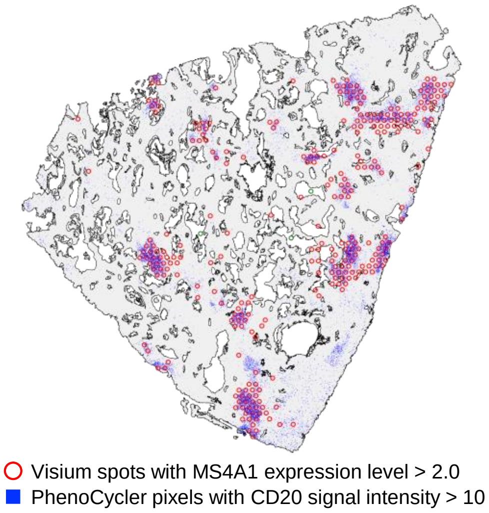
Visium spots with MS4A1 expression level > 2.0
PhenoCycler pixels with CD20 signal intensity > 10

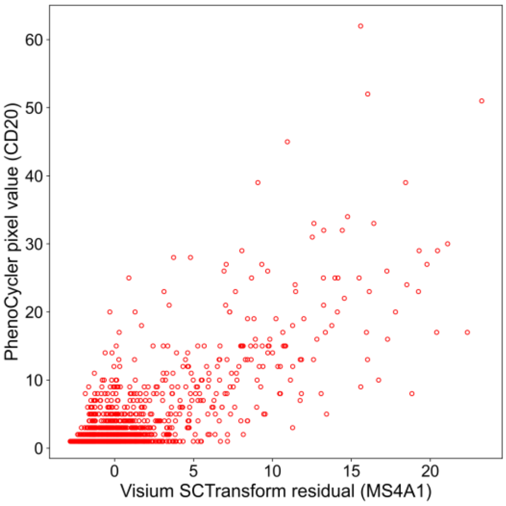
PhenoCycler pixel value (CD20)
Visium SCTransform residual (MS4A1)
0
10
20
30
40
50
60
0
5
10
15
20

**c**

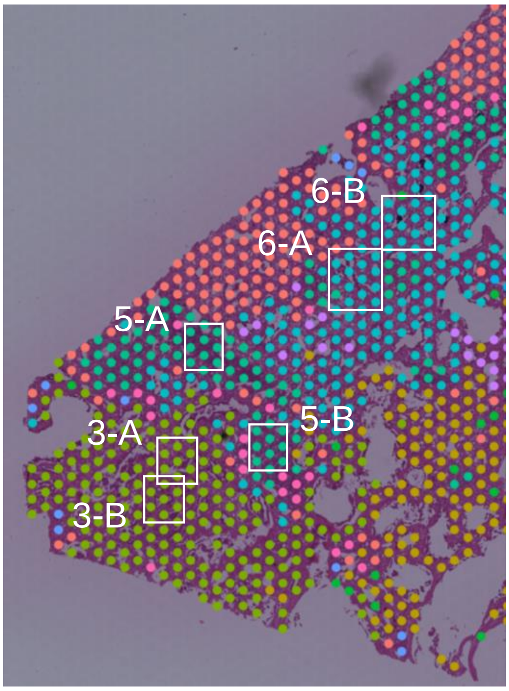
6-B
6-A
5-A
5-B
3-A
3-B

*Supplementary Figure S6*

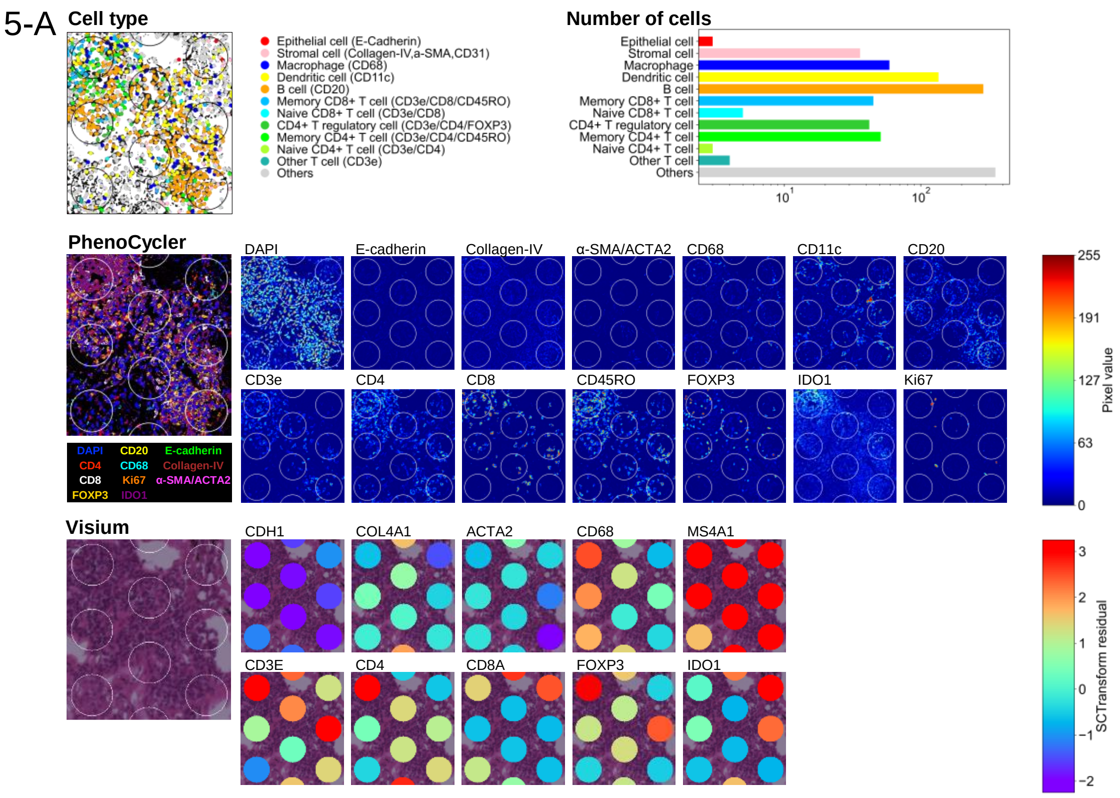


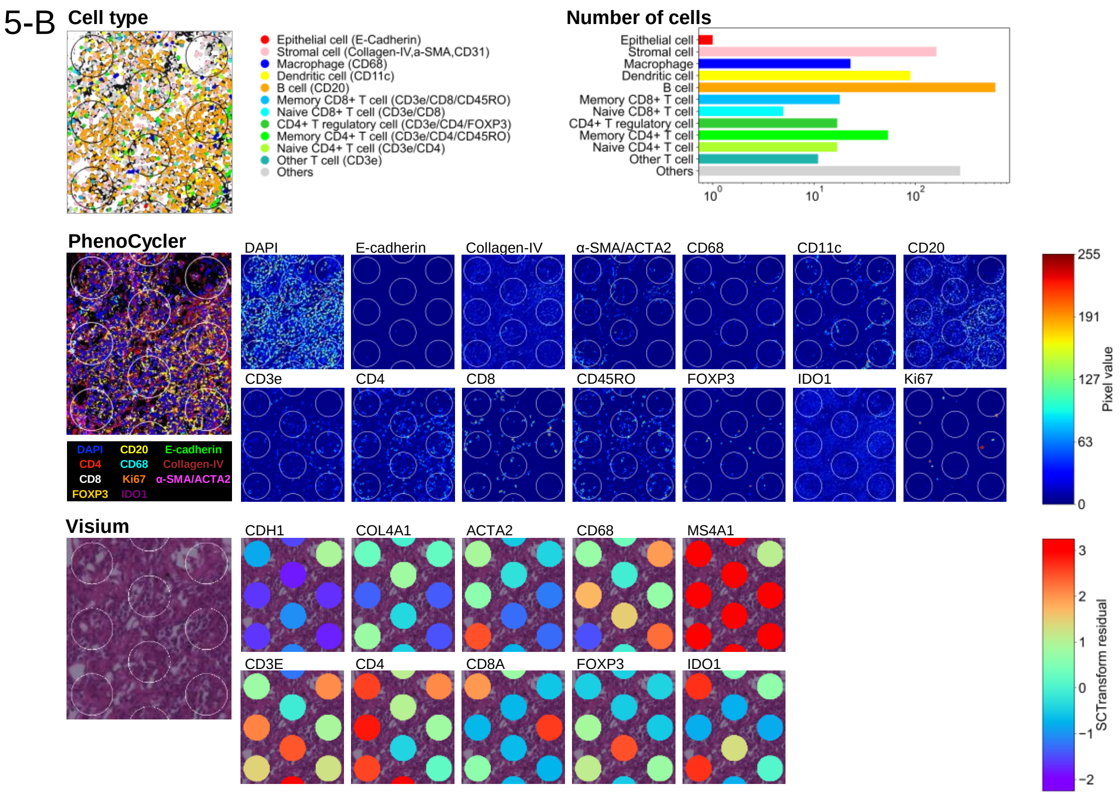

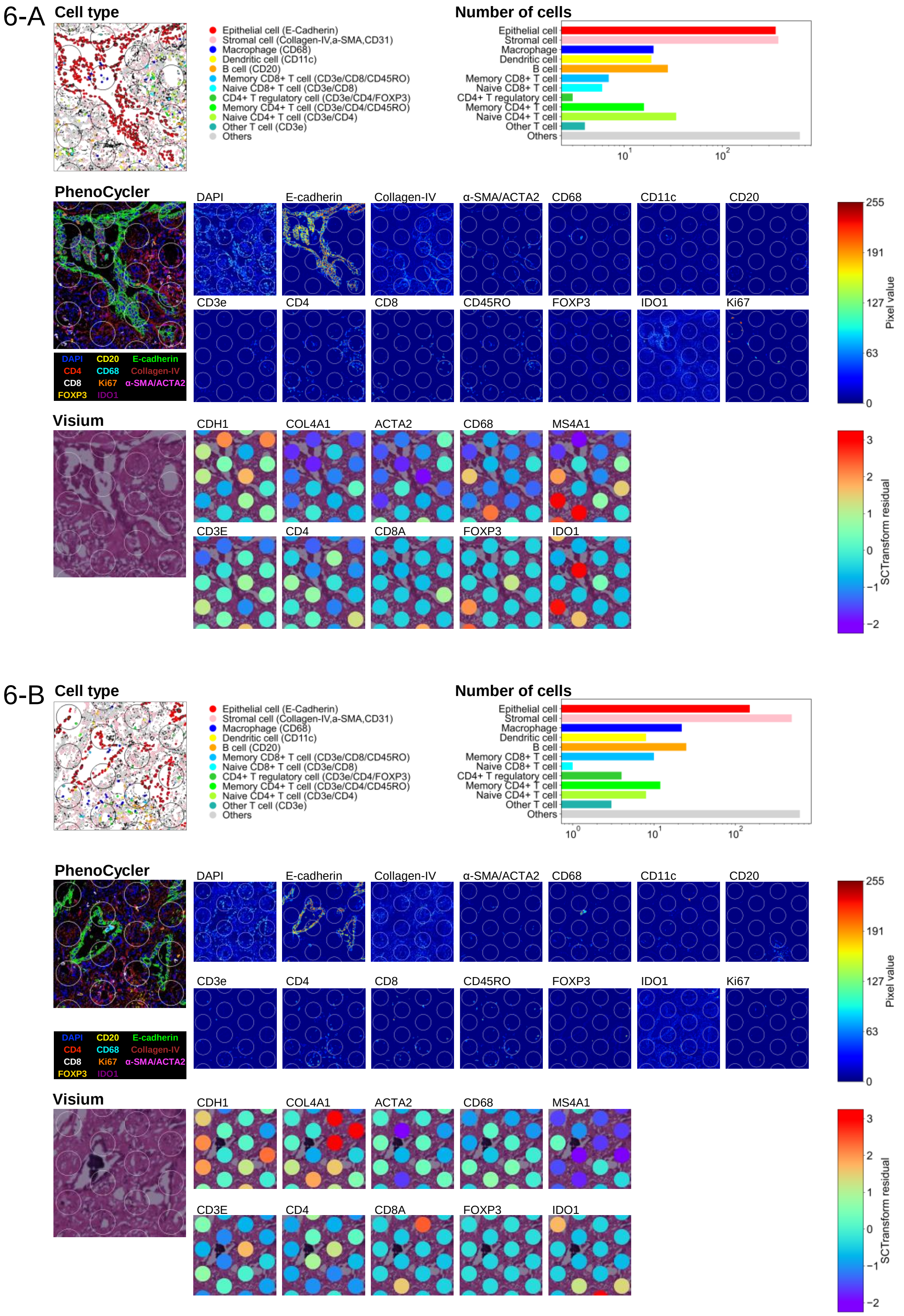
6-A
Cell type
Epithelial cell (E-Cadherin)
Stromal cell (Collagen-IV,a-SMA,CD31)
Macrophage (CD68)
Dendritic cell (CD11c)
B cell (CD20)
Memory CD8+ T cell (CD3e/CD8/CD45RO)
Naive CD8+ T cell (CD3e/CD8)
CD4+ T regulatory cell (CD3e/CD4/FOXP3)
Memory CD4+ T cell (CD3e/CD4/CD45RO)
Naive CD4+ T cell (CD3e/CD4)
Other T cell (CD3e)
Others
Number of cells
Epithelial cell
Stromal cell
Macrophage
Dendritic cell
B cell
Memory CD8+ T cell
Naive CD8+ T cell
CD4+ T regulatory cell
Memory CD4+ T cell
Naive CD4+ T cell
Other T cell
Others
$10^1$
$10^2$
PhenoCycler
DAPI
E-cadherin
Collagen-IV
α-SMA/ACTA2
CD68
CD11c
CD20
CD3e
CD4
CD8
CD45RO
FOXP3
IDO1
Ki67
DAPI CD20 E-cadherin
CD4 CD68 Collagen-IV
CD8 Ki67 α-SMA/ACTA2
FOXP3 IDO1
255
191
127
63
0
Pixel value
Visium
CDH1
COL4A1
ACTA2
CD68
MS4A1
CD3E
CD4
CD8A
FOXP3
IDO1
3
2
1
0
−1
−2
SCTransform residual
6-B
Cell type
Epithelial cell (E-Cadherin)
Stromal cell (Collagen-IV,a-SMA,CD31)
Macrophage (CD68)
Dendritic cell (CD11c)
B cell (CD20)
Memory CD8+ T cell (CD3e/CD8/CD45RO)
Naive CD8+ T cell (CD3e/CD8)
CD4+ T regulatory cell (CD3e/CD4/FOXP3)
Memory CD4+ T cell (CD3e/CD4/CD45RO)
Naive CD4+ T cell (CD3e/CD4)
Other T cell (CD3e)
Others
Number of cells
Epithelial cell
Stromal cell
Macrophage
Dendritic cell
B cell
Memory CD8+ T cell
Naive CD8+ T cell
CD4+ T regulatory cell
Memory CD4+ T cell
Naive CD4+ T cell
Other T cell
Others
$10^0$
$10^1$
$10^2$
PhenoCycler
DAPI
E-cadherin
Collagen-IV
α-SMA/ACTA2
CD68
CD11c
CD20
CD3e
CD4
CD8
CD45RO
FOXP3
IDO1
Ki67
DAPI CD20 E-cadherin
CD4 CD68 Collagen-IV
CD8 Ki67 α-SMA/ACTA2
FOXP3 IDO1
255
191
127
63
0
Pixel value
Visium
CDH1
COL4A1
ACTA2
CD68
MS4A1
CD3E
CD4
CD8A
FOXP3
IDO1
3
2
1
0
−1
−2
SCTransform residual

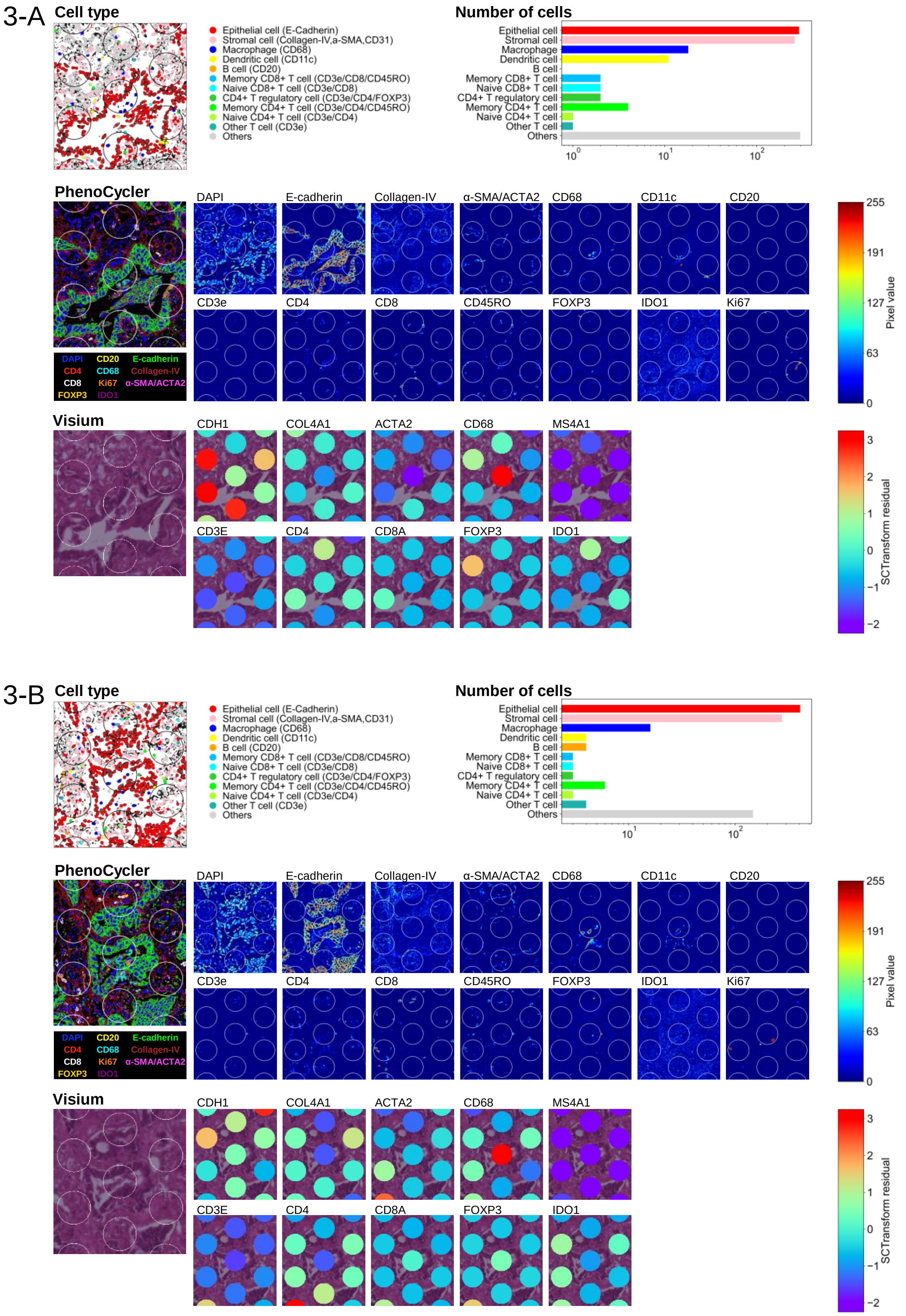
3-A
Cell type
Epithelial cell (E-Cadherin)
Stromal cell (Collagen-IV,a-SMA,CD31)
Macrophage (CD68)
Dendritic cell (CD11c)
B cell (CD20)
Memory CD8+ T cell (CD3e/CD8/CD45RO)
Naive CD8+ T cell (CD3e/CD8)
CD4+ T regulatory cell (CD3e/CD4/FOXP3)
Memory CD4+ T cell (CD3e/CD4/CD45RO)
Naive CD4+ T cell (CD3e/CD4)
Other T cell (CD3e)
Others
Number of cells
Epithelial cell
Stromal cell
Macrophage
Dendritic cell
B cell
Memory CD8+ T cell
Naive CD8+ T cell
CD4+ T regulatory cell
Memory CD4+ T cell
Naive CD4+ T cell
Other T cell
Others
10^0
10^1
10^2
PhenoCycler
DAPI CD20 E-cadherin
CD4 CD68 Collagen-IV
CD8 Ki67 α-SMA/ACTA2
FOXP3 IDO1
DAPI
E-cadherin
Collagen-IV
α-SMA/ACTA2
CD68
CD11c
CD20
CD3e
CD4
CD8
CD45RO
FOXP3
IDO1
Ki67
255
191
127
63
0
Pixel value
Visium
CDH1
COL4A1
ACTA2
CD68
MS4A1
CD3E
CD4
CD8A
FOXP3
IDO1
3
2
1
0
−1
−2
SCTransform residual
3-B
Cell type
Epithelial cell (E-Cadherin)
Stromal cell (Collagen-IV,a-SMA,CD31)
Macrophage (CD68)
Dendritic cell (CD11c)
B cell (CD20)
Memory CD8+ T cell (CD3e/CD8/CD45RO)
Naive CD8+ T cell (CD3e/CD8)
CD4+ T regulatory cell (CD3e/CD4/FOXP3)
Memory CD4+ T cell (CD3e/CD4/CD45RO)
Naive CD4+ T cell (CD3e/CD4)
Other T cell (CD3e)
Others
Number of cells
Epithelial cell
Stromal cell
Macrophage
Dendritic cell
B cell
Memory CD8+ T cell
Naive CD8+ T cell
CD4+ T regulatory cell
Memory CD4+ T cell
Naive CD4+ T cell
Other T cell
Others
10^1
10^2
PhenoCycler
DAPI CD20 E-cadherin
CD4 CD68 Collagen-IV
CD8 Ki67 α-SMA/ACTA2
FOXP3 IDO1
DAPI
E-cadherin
Collagen-IV
α-SMA/ACTA2
CD68
CD11c
CD20
CD3e
CD4
CD8
CD45RO
FOXP3
IDO1
Ki67
255
191
127
63
0
Pixel value
Visium
CDH1
COL4A1
ACTA2
CD68
MS4A1
CD3E
CD4
CD8A
FOXP3
IDO1
3
2
1
0
−1
−2
SCTransform residual

**Supplementary Figure S6 Workflow and results for the integration of Visium and PhenoCycler image data**

(**a**) Procedure for integrating Visium and PhenoCycler data. 1) The Visium H&E stained image and PhenoCycler fluorescence image were binarized to generate mask images using contour extraction. 2) Mask images of Visium (red) and PhenoCycler (blue) were scaled, translated, and rotated to maximize overlap based on their intersection over union (IoU), which is defined as (Visium ∩ PhenoCycler)/(Visium ∪ PhenoCycler). Using an exhaustive grid search in the vicinity of the above-stated solution, IoU was optimized to generate the final transformations. In the case of LUAD No. 3 FFPE (section B), IoU improved from 0.36 to 0.85, indicating adequate alignment quality for integrating the spatial datasets of various resolutions and orientations. 3) A mathematical function representing the transformations used in image alignment was developed to reciprocally associate pixel positions in Visium and PhenoCycler images. 4) Signals in Visium and PhenoCycler images were positionally integrated together using the mathematical function and its inverse. The pixels in a region of interest (ROI), represented by a white square in the PhenoCycler image (left panel), were mapped into the corresponding square in the Visium image (right panel) using the mathematical function generated by image alignment. (**b**) Expression patterns of MS4A1/CD20 (B cell marker) in Visium and PhenoCycler in LUAD No. 3 FFPE (section B). Left panel shows the positional relationship between Visium spots and PhenoCycler pixels with high signal levels. Spots with high *MS4A1* expression (>2.0) are shown as red circles, while pixels with high fluorescence level of the CD20 antibody (>10) are shown as blue dots. Right panel shows a comparison of MS4A1/CD20 expression levels in Visium and PhenoCycler. Each marker denotes a Visium spot. The horizontal axis shows the gene expression level of *MS4A1* determined by the residual of the SCTransform normalization of Visium count data. The vertical axis shows the expression level of the CD20 antibody, as measured by the average of non-zero fluorescence signals in the circular region of the PhenoCycler data to which the Visium spot corresponds. (**c**) ROIs for LUAD No. 3 FFPE (section B). For ROIs in clusters 3 and 6 (3-A, 3-B, 6-A, and 6-B) and cluster 5 (5-A and 5-B) (**Fig. 3e**), the types of cells and their frequencies detected using PhenoCycler fluorescence data are schematically presented. The fluorescence intensities of individual antibodies with PhenoCycler were compared to the positions of the circular regions of Visium spots. Superimposed images of the selected antibodies' fluorescence intensities were generated. The expression levels of the genes analyzed by Visium spots were plotted against the H&E staining image to investigate the relationships between Visium and PhenoCycler

expression levels.

Note:

We demonstrated that the integration of Visium and PhenoCycler data could be used to identify and validate cell types, particularly immune cells, which are sometimes difficult to decipher using Visium data due to low spatial resolution and differences in RNA and protein expression. For immune cell cluster 5 in LUAD No. 3 FFPE section B, we confirmed that B cells dominated while CD4+ Tregs were enriched in the downstream ROI (5-A) (also discussed in **Supplementary Figure S5**). For cluster 6, we confirmed infiltration of immune cells, including T and B cells. For cluster 3, where TAM-like alveolar macrophages were detected in Visium, we also found macrophages next to tumor cells in PhenoCycler data.

**a**

Tumor cell: Well-differentiated

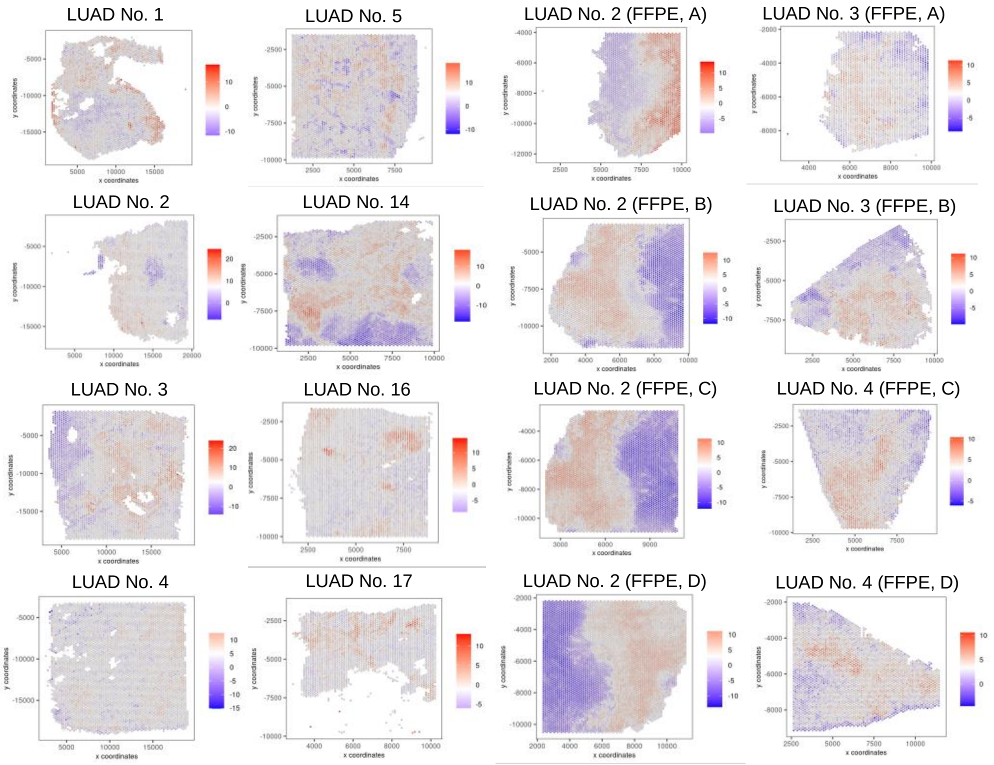


**b**

Tumor cell: Proliferative

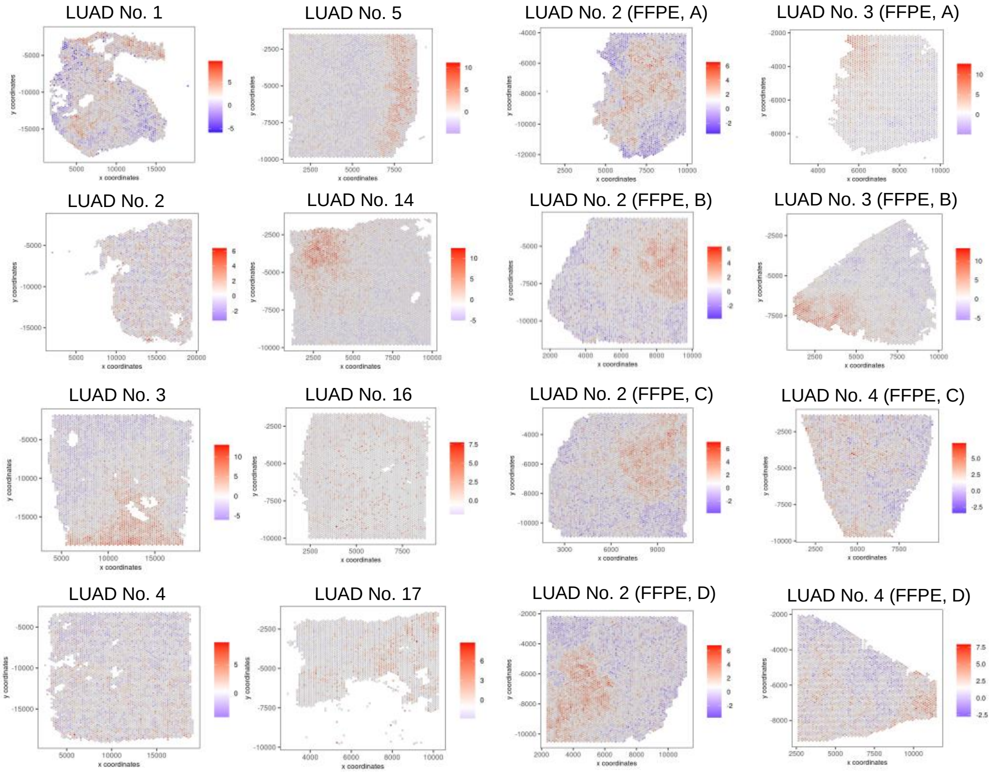

**c**

Tumor cell: Invasive

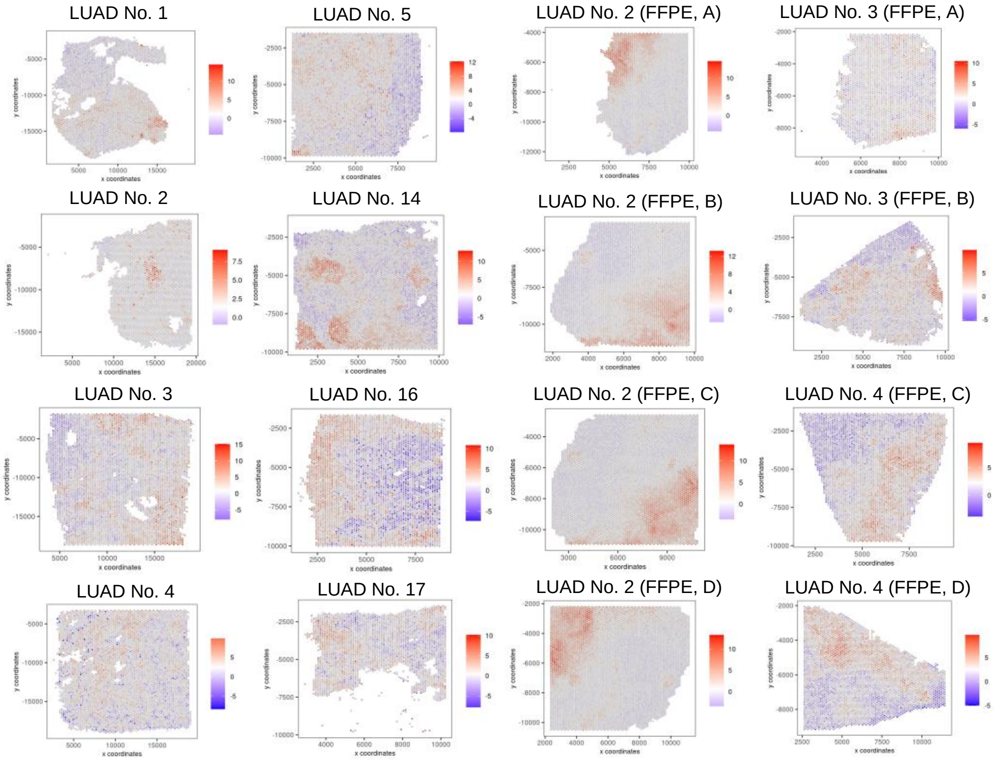


**d**

Microenvironment: Fibroblast/CAF

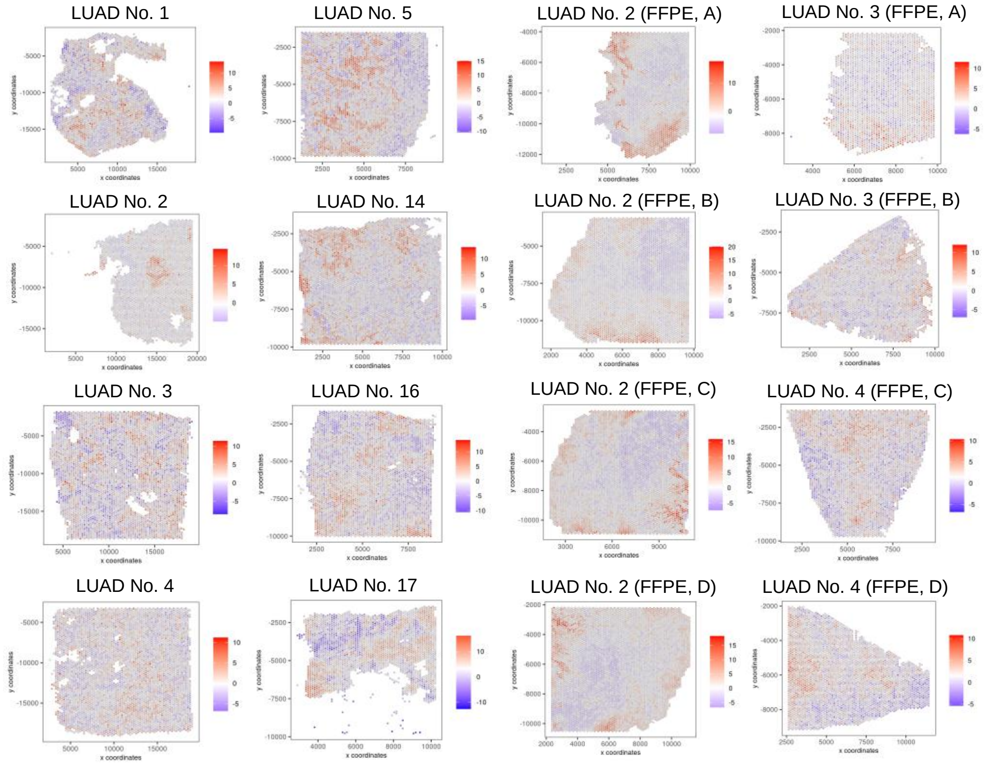

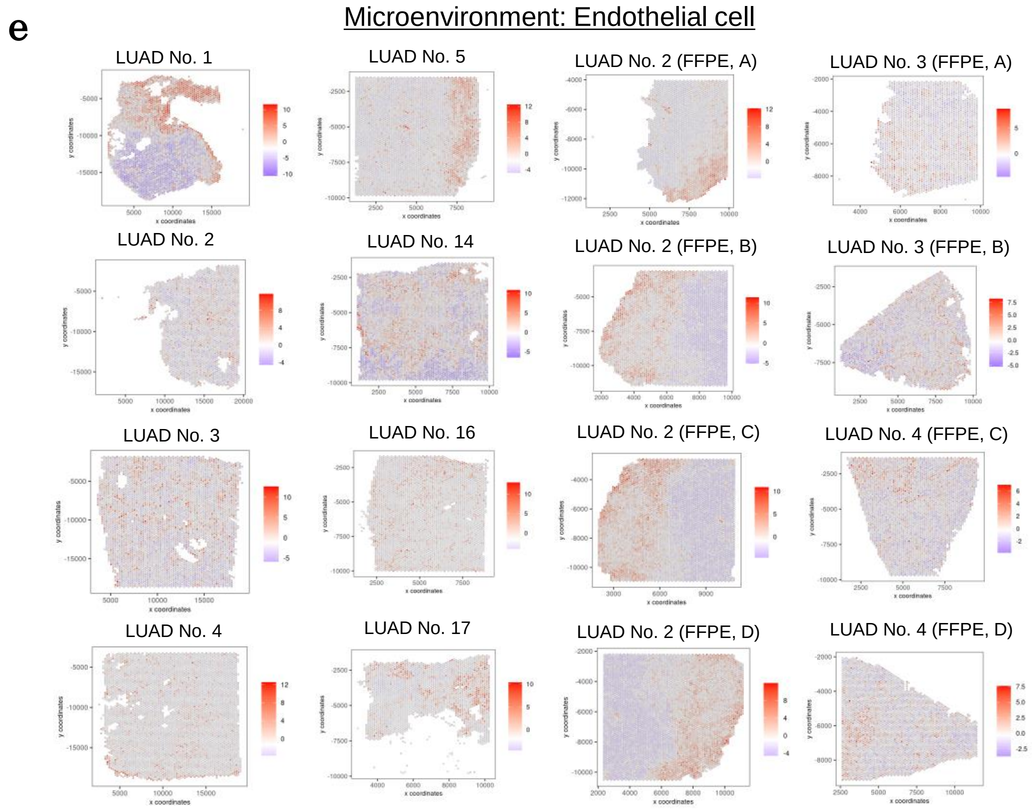
e
Microenvironment: Endothelial cell
LUAD No. 1
LUAD No. 5
LUAD No. 2 (FFPE, A)
LUAD No. 3 (FFPE, A)
LUAD No. 2
LUAD No. 14
LUAD No. 2 (FFPE, B)
LUAD No. 3 (FFPE, B)
LUAD No. 3
LUAD No. 16
LUAD No. 2 (FFPE, C)
LUAD No. 4 (FFPE, C)
LUAD No. 4
LUAD No. 17
LUAD No. 2 (FFPE, D)
LUAD No. 4 (FFPE, D)
x coordinates
y coordinates

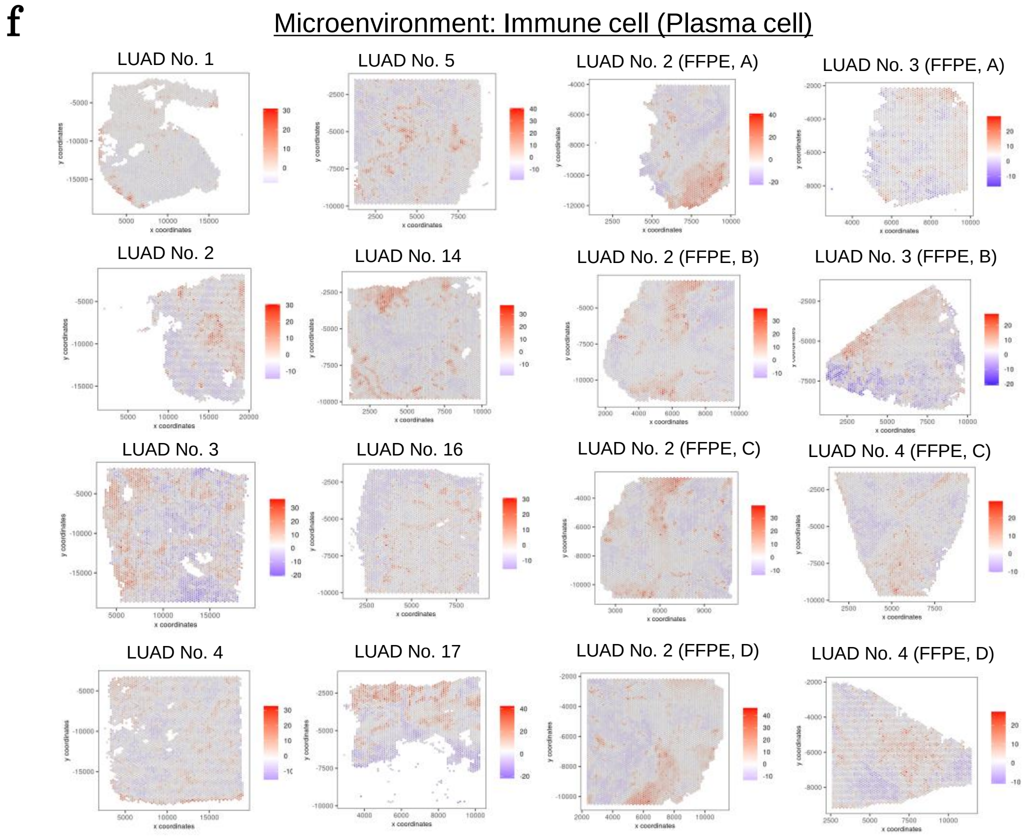
f
Microenvironment: Immune cell (Plasma cell)
LUAD No. 1
LUAD No. 5
LUAD No. 2 (FFPE, A)
LUAD No. 3 (FFPE, A)
LUAD No. 2
LUAD No. 14
LUAD No. 2 (FFPE, B)
LUAD No. 3 (FFPE, B)
LUAD No. 3
LUAD No. 16
LUAD No. 2 (FFPE, C)
LUAD No. 4 (FFPE, C)
LUAD No. 4
LUAD No. 17
LUAD No. 2 (FFPE, D)
LUAD No. 4 (FFPE, D)
x coordinates
y coordinates

**g**

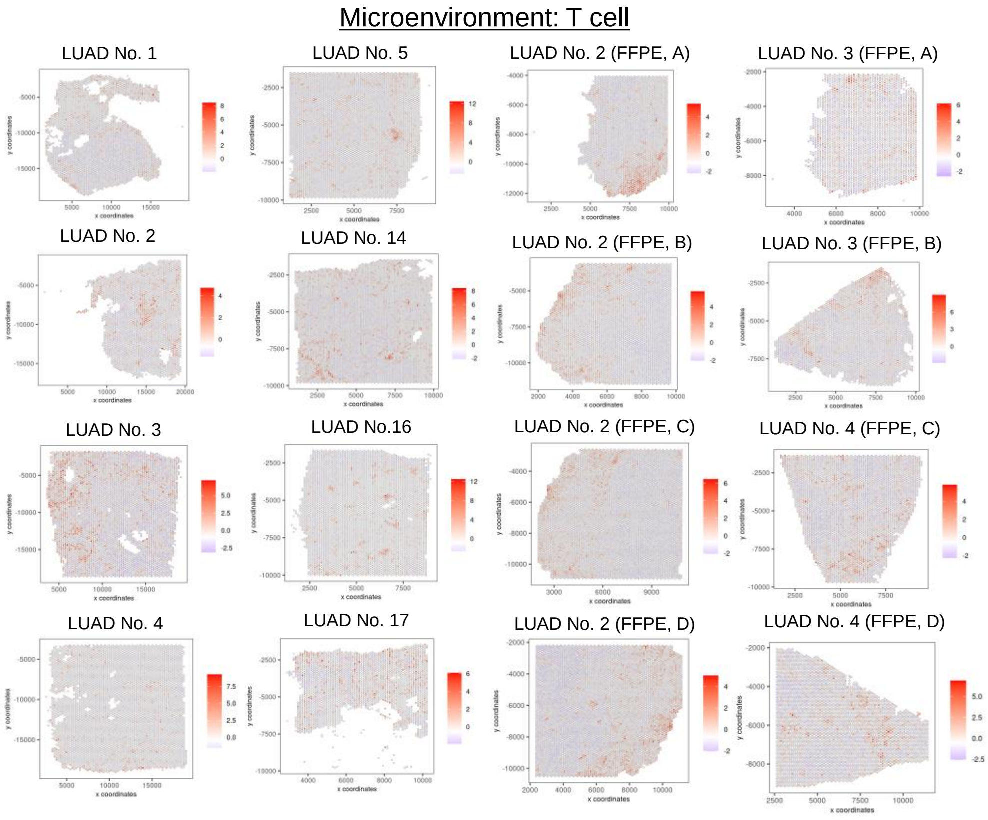
Microenvironment: T cell
LUAD No. 1
LUAD No. 5
LUAD No. 2 (FFPE, A)
LUAD No. 3 (FFPE, A)
LUAD No. 2
LUAD No. 14
LUAD No. 2 (FFPE, B)
LUAD No. 3 (FFPE, B)
LUAD No. 3
LUAD No.16
LUAD No. 2 (FFPE, C)
LUAD No. 4 (FFPE, C)
LUAD No. 4
LUAD No. 17
LUAD No. 2 (FFPE, D)
LUAD No. 4 (FFPE, D)
x coordinates
y coordinates

**h**

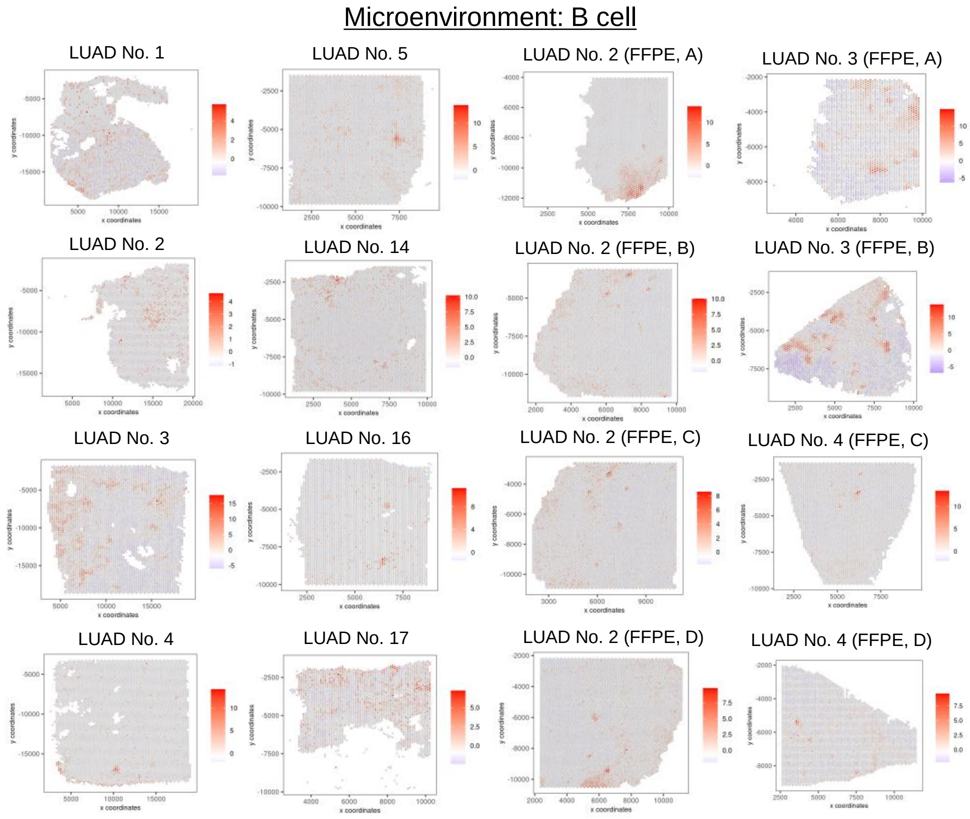
Microenvironment: B cell
LUAD No. 1
LUAD No. 5
LUAD No. 2 (FFPE, A)
LUAD No. 3 (FFPE, A)
LUAD No. 2
LUAD No. 14
LUAD No. 2 (FFPE, B)
LUAD No. 3 (FFPE, B)
LUAD No. 3
LUAD No. 16
LUAD No. 2 (FFPE, C)
LUAD No. 4 (FFPE, C)
LUAD No. 4
LUAD No. 17
LUAD No. 2 (FFPE, D)
LUAD No. 4 (FFPE, D)
x coordinates
y coordinates

**i**

## Microenvironment: M1 macrophage

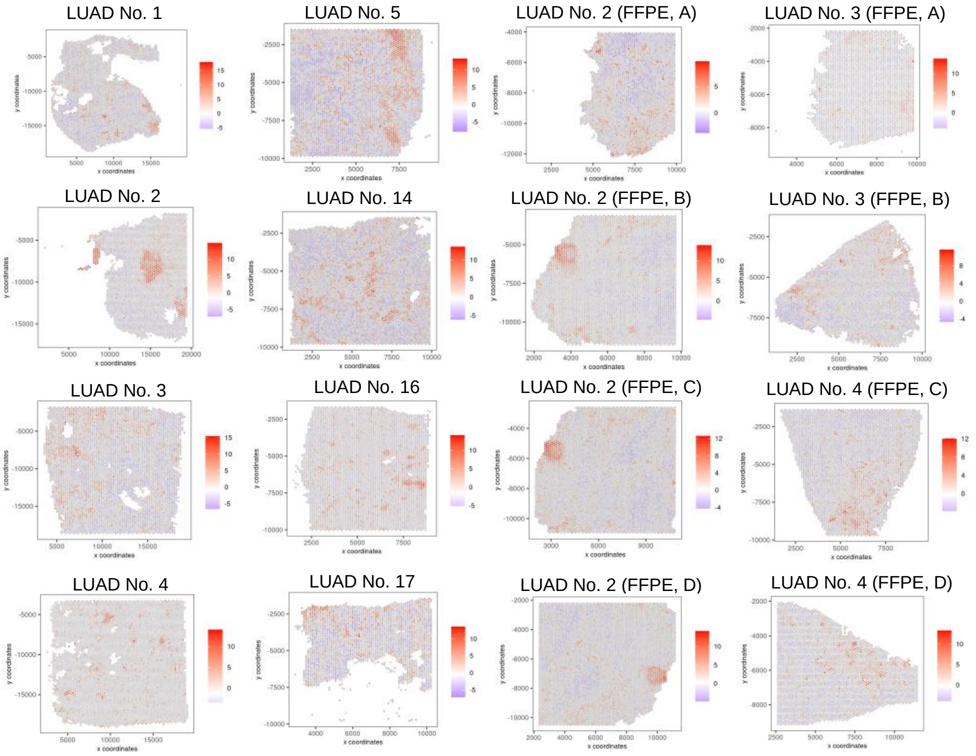


**j**

## Microenvironment: M2 macrophage

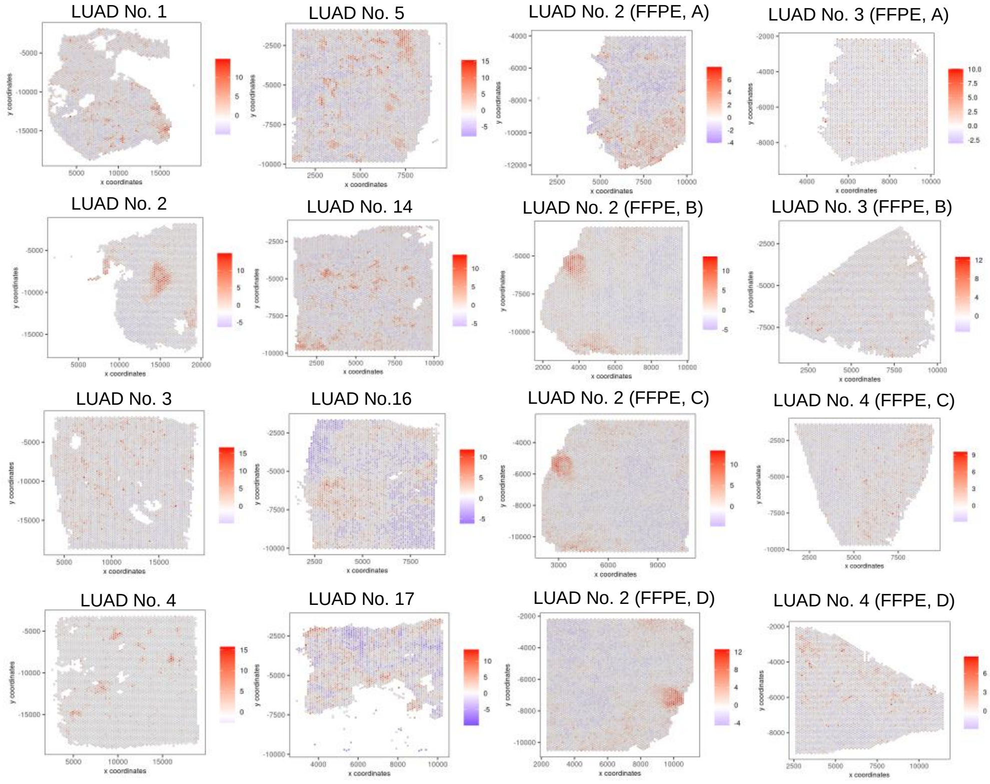

**Supplementary Figure S7 TME scoring in all IA sections or cases**

Results of tumor microenvironment (TME) scoring using PAGE analysis by Giotto[3]. The spatial distributions of scores for well-differentiated, proliferative, and invasive tumor cells are shown in panels **a**, **b**, and **c**, respectively. For non-tumor cells, the spatial distributions of the scores are represented by **d–j**.

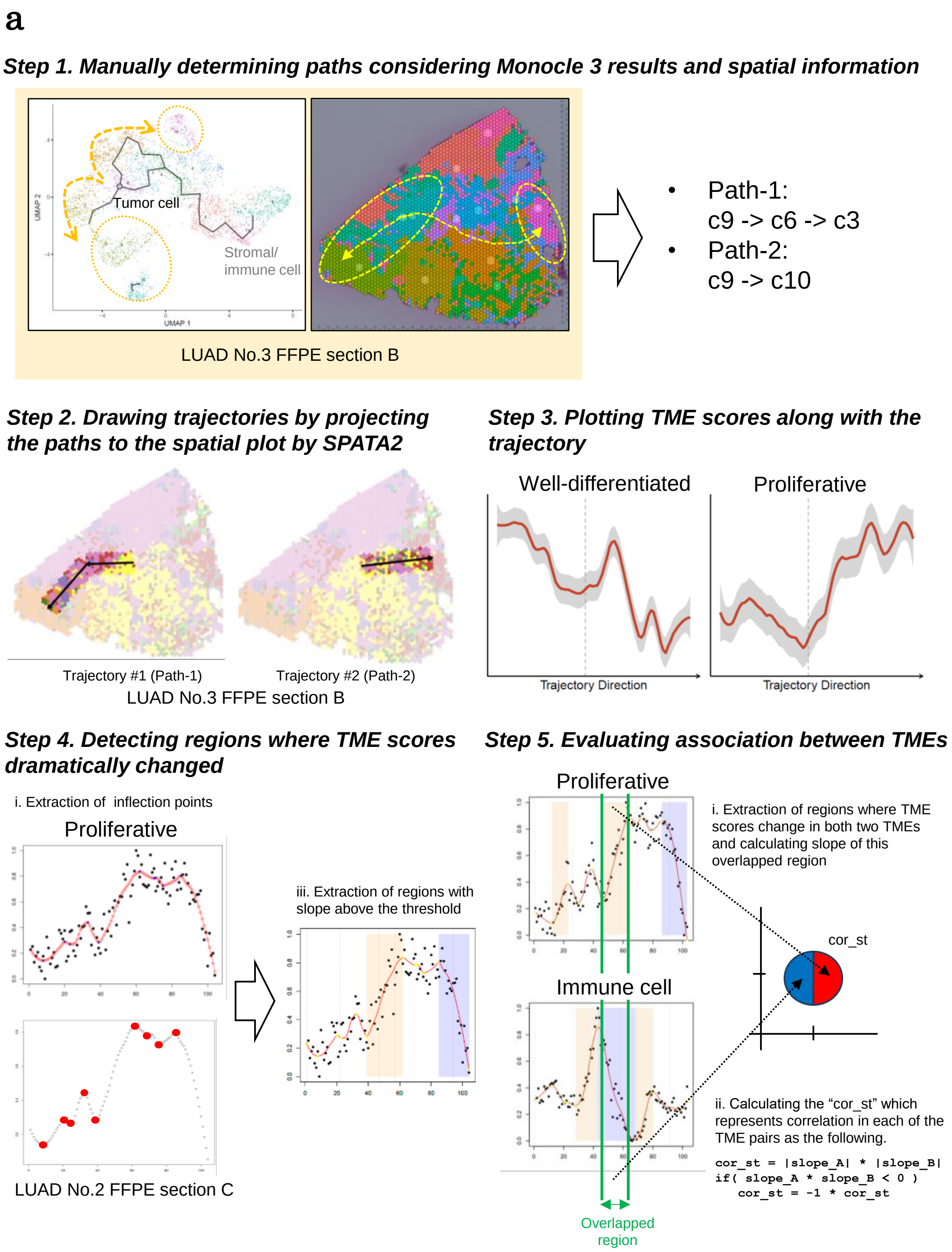
a
Step 1. Manually determining paths considering Monocle 3 results and spatial information
Tumor cell
Stromal/
immune cell
UMAP 1
UMAP 2
LUAD No.3 FFPE section B
Path-1:
c9 -> c6 -> c3
Path-2:
c9 -> c10
Step 2. Drawing trajectories by projecting the paths to the spatial plot by SPATA2
Trajectory #1 (Path-1)
Trajectory #2 (Path-2)
LUAD No.3 FFPE section B
Step 3. Plotting TME scores along with the trajectory
Well-differentiated
Proliferative
Trajectory Direction
Trajectory Direction
Step 4. Detecting regions where TME scores dramatically changed
i. Extraction of inflection points
Proliferative
iii. Extraction of regions with slope above the threshold
LUAD No.2 FFPE section C
Step 5. Evaluating association between TMEs
Proliferative
i. Extraction of regions where TME scores change in both two TMEs and calculating slope of this overlapped region
cor_st
Immune cell
ii. Calculating the "cor_st" which represents correlation in each of the TME pairs as the following.
cor_st = |slope_A| * |slope_B|
if( slope_A * slope_B < 0 )
cor_st = -1 * cor_st
Overlapped
region

**b**

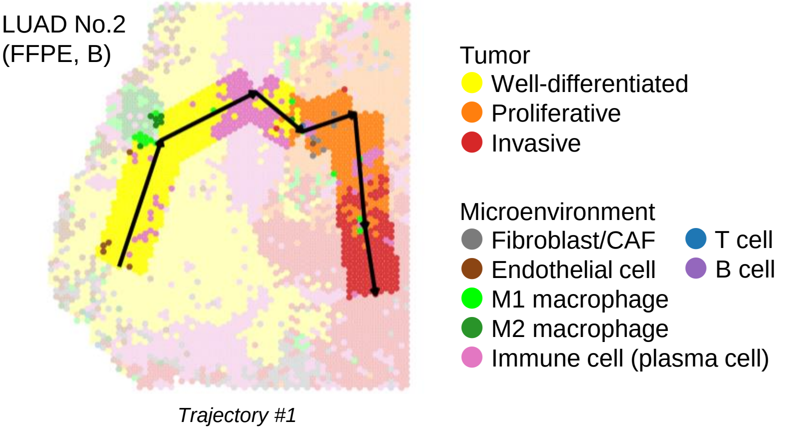

LUAD No.2
(FFPE, B)
Tumor
Well-differentiated
Proliferative
Invasive
Microenvironment
Fibroblast/CAF
Endothelial cell
M1 macrophage
M2 macrophage
Immune cell (plasma cell)
T cell
B cell
Trajectory #1


**c**

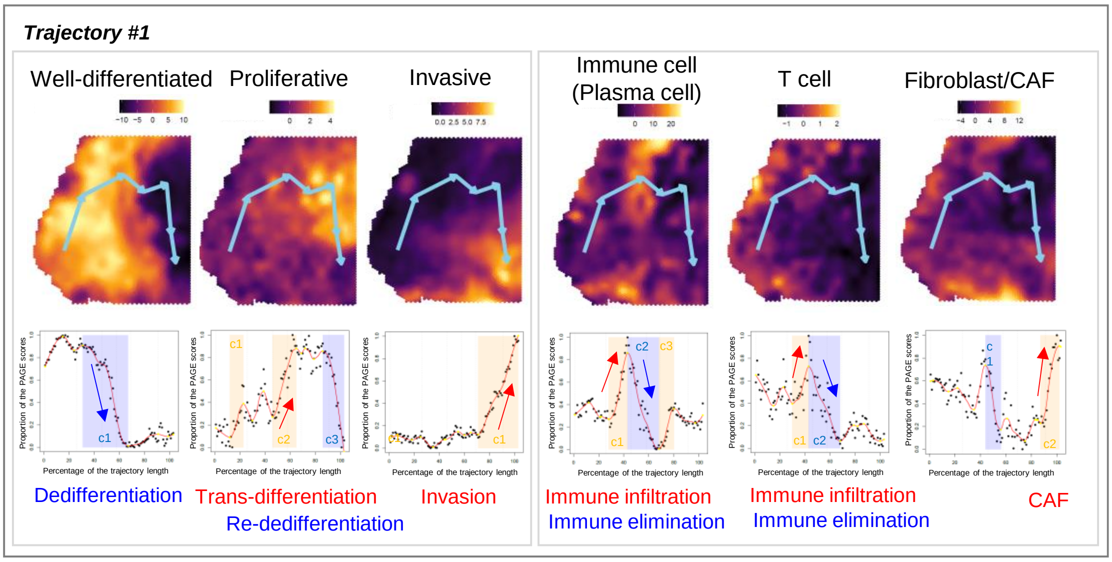

Trajectory #1
Well-differentiated
Proliferative
Invasive
Immune cell
(Plasma cell)
T cell
Fibroblast/CAF
Proportion of the PAGE scores
Percentage of the trajectory length
Dedifferentiation
Trans-differentiation
Re-dedifferentiation
Invasion
Immune infiltration
Immune elimination
Immune infiltration
Immune elimination
CAF

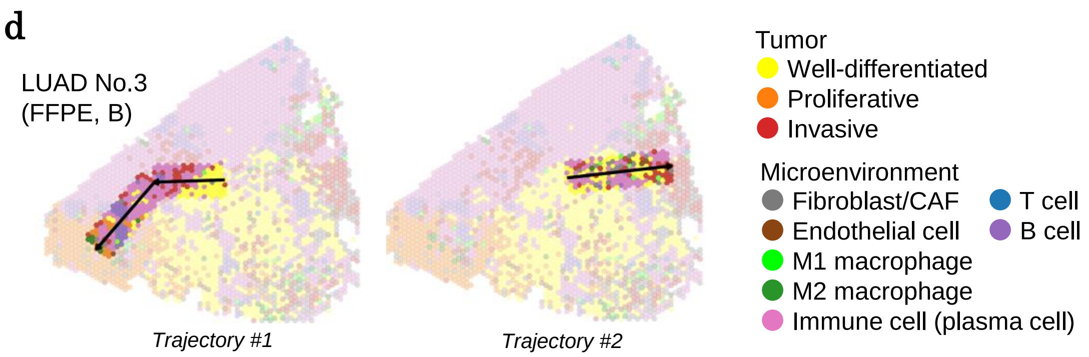
d
LUAD No.3
(FFPE, B)
Trajectory #1
Trajectory #2
Tumor
Well-differentiated
Proliferative
Invasive
Microenvironment
Fibroblast/CAF
Endothelial cell
M1 macrophage
M2 macrophage
Immune cell (plasma cell)
T cell
B cell

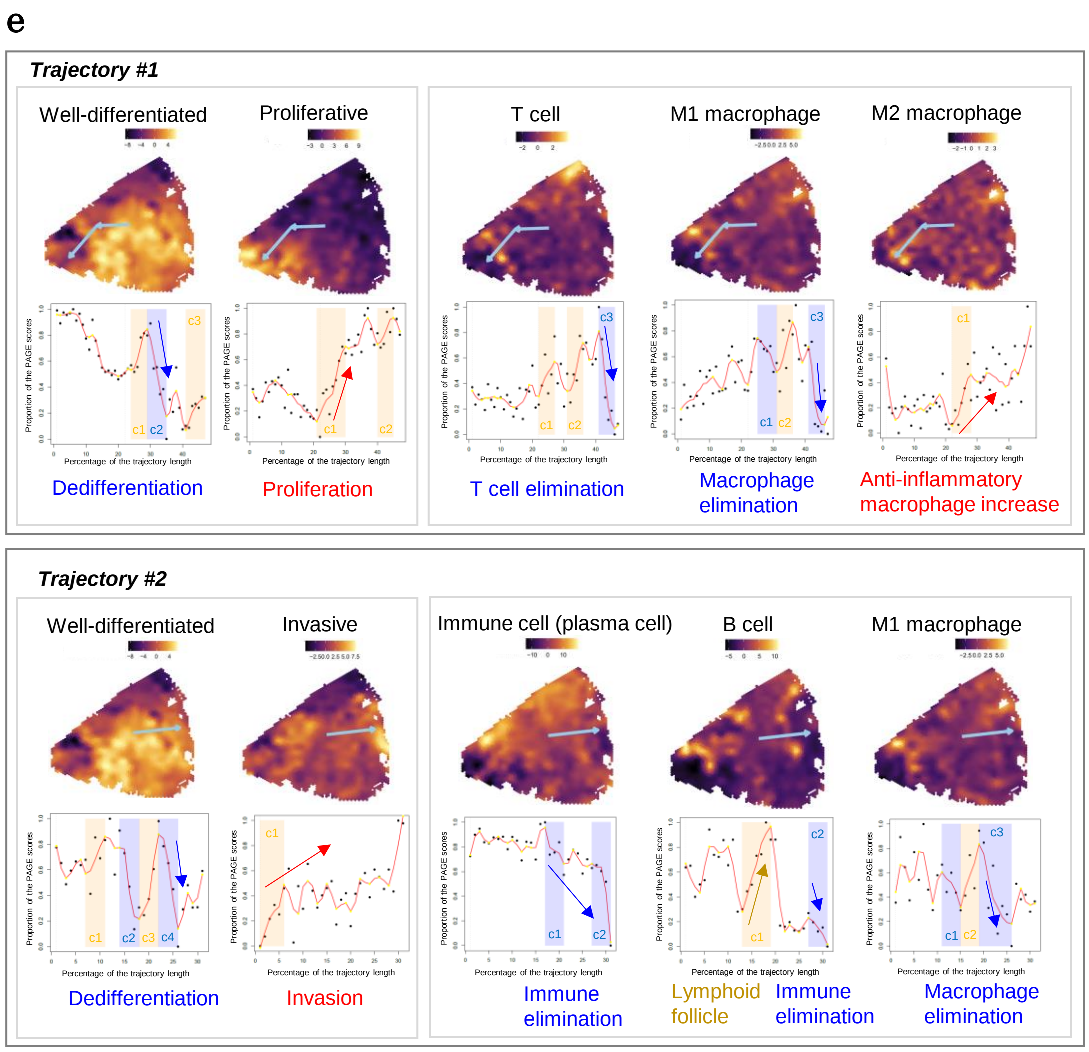
e
Trajectory #1
Well-differentiated
Proliferative
T cell
M1 macrophage
M2 macrophage
Dedifferentiation
Proliferation
T cell elimination
Macrophage elimination
Anti-inflammatory macrophage increase
Trajectory #2
Well-differentiated
Invasive
Immune cell (plasma cell)
B cell
M1 macrophage
Dedifferentiation
Invasion
Immune elimination
Lymphoid follicle
Immune elimination
Macrophage elimination

**f**

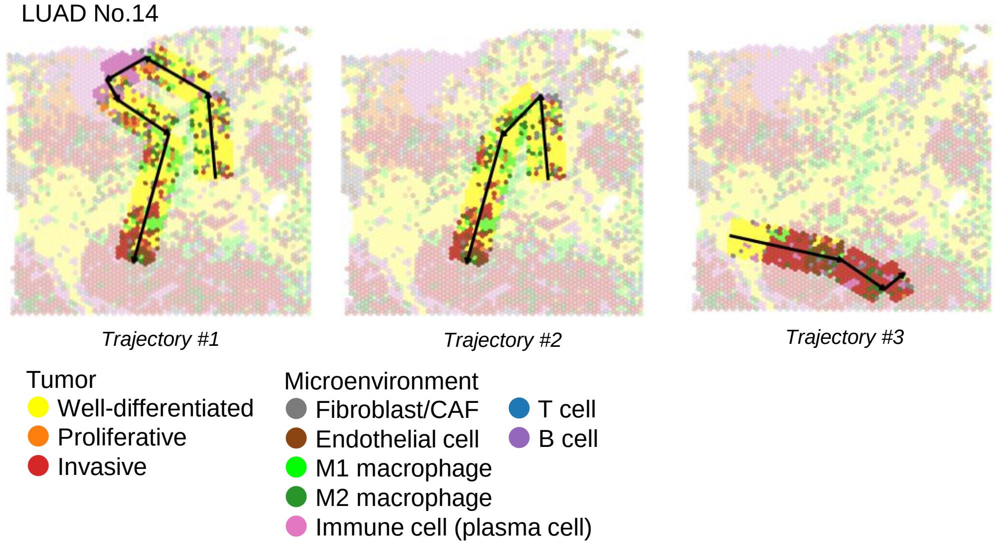
LUAD No.14
Trajectory #1
Trajectory #2
Trajectory #3
Tumor
Well-differentiated
Proliferative
Invasive
Microenvironment
Fibroblast/CAF
Endothelial cell
M1 macrophage
M2 macrophage
Immune cell (plasma cell)
T cell
B cell

**g**

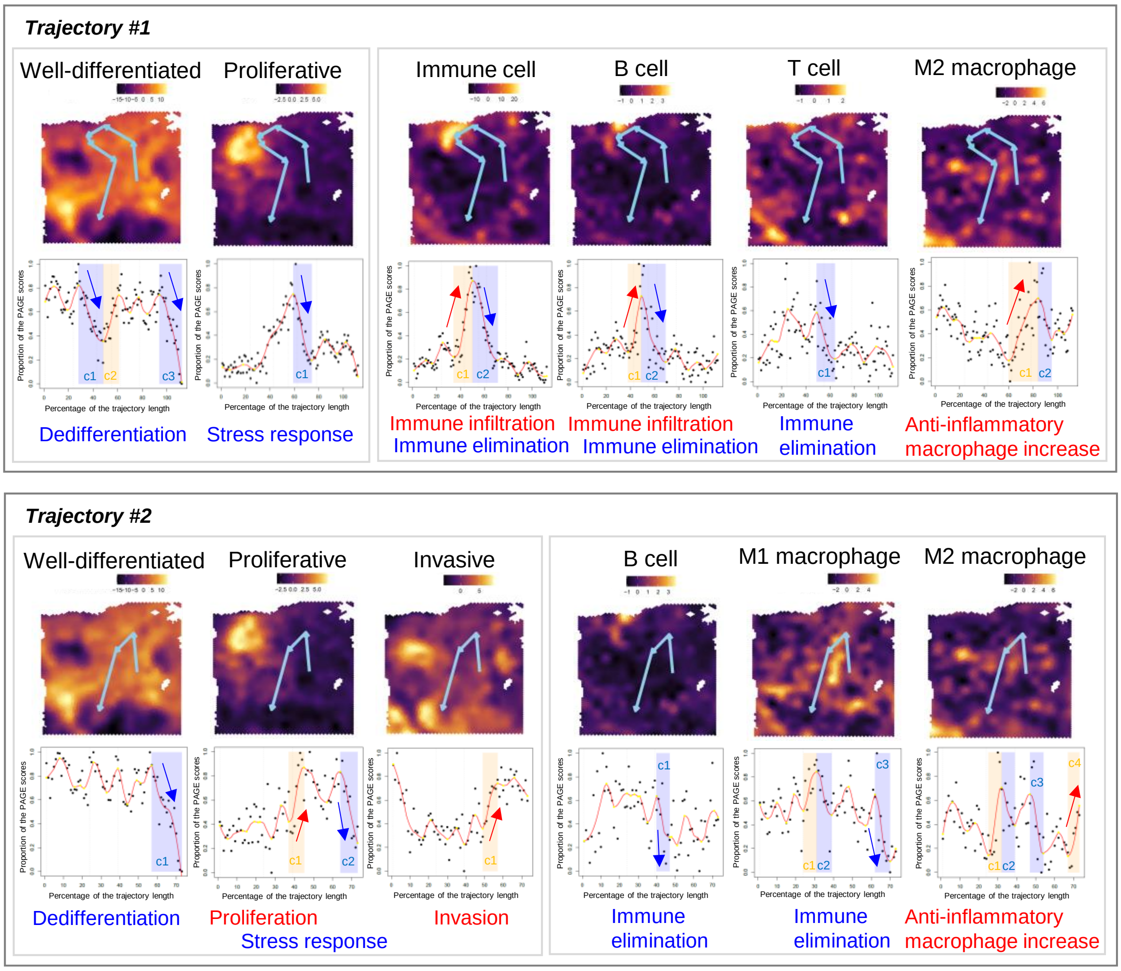
Trajectory #1
Well-differentiated
Proliferative
Immune cell
B cell
T cell
M2 macrophage
Percentage of the trajectory length
Proportion of the PAGE scores
Dedifferentiation
Stress response
Immune infiltration
Immune elimination
Immune infiltration
Immune elimination
Immune elimination
Anti-inflammatory macrophage increase
Trajectory #2
Well-differentiated
Proliferative
Invasive
B cell
M1 macrophage
M2 macrophage
Dedifferentiation
Proliferation
Stress response
Invasion
Immune elimination
Immune elimination
Anti-inflammatory macrophage increase

*Supplementary Figure S8*

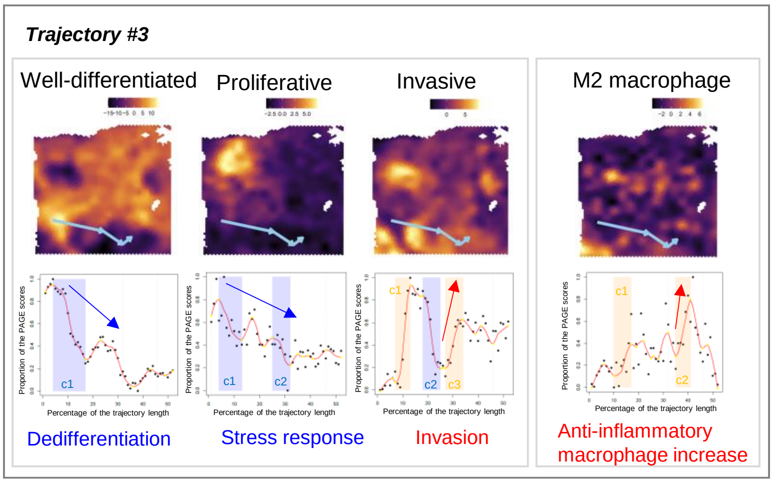


**h**

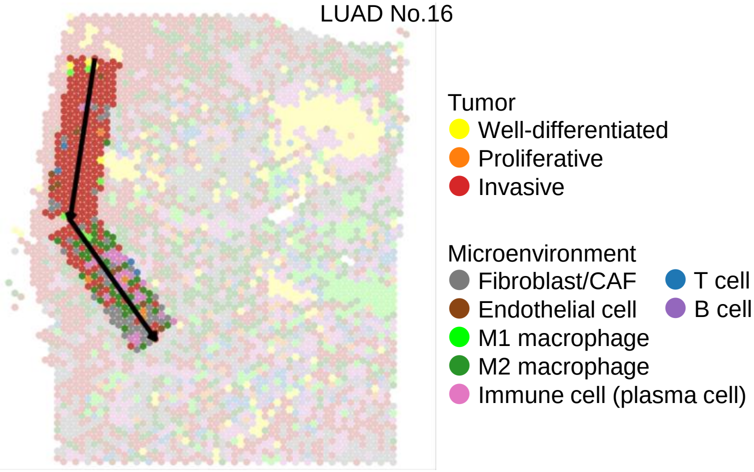


*Trajectory #1*

**i**

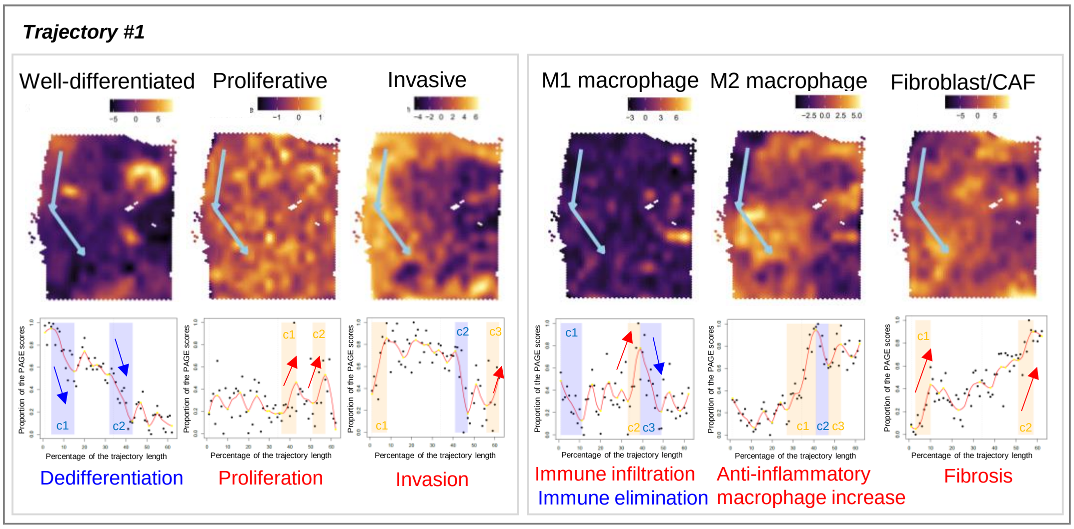

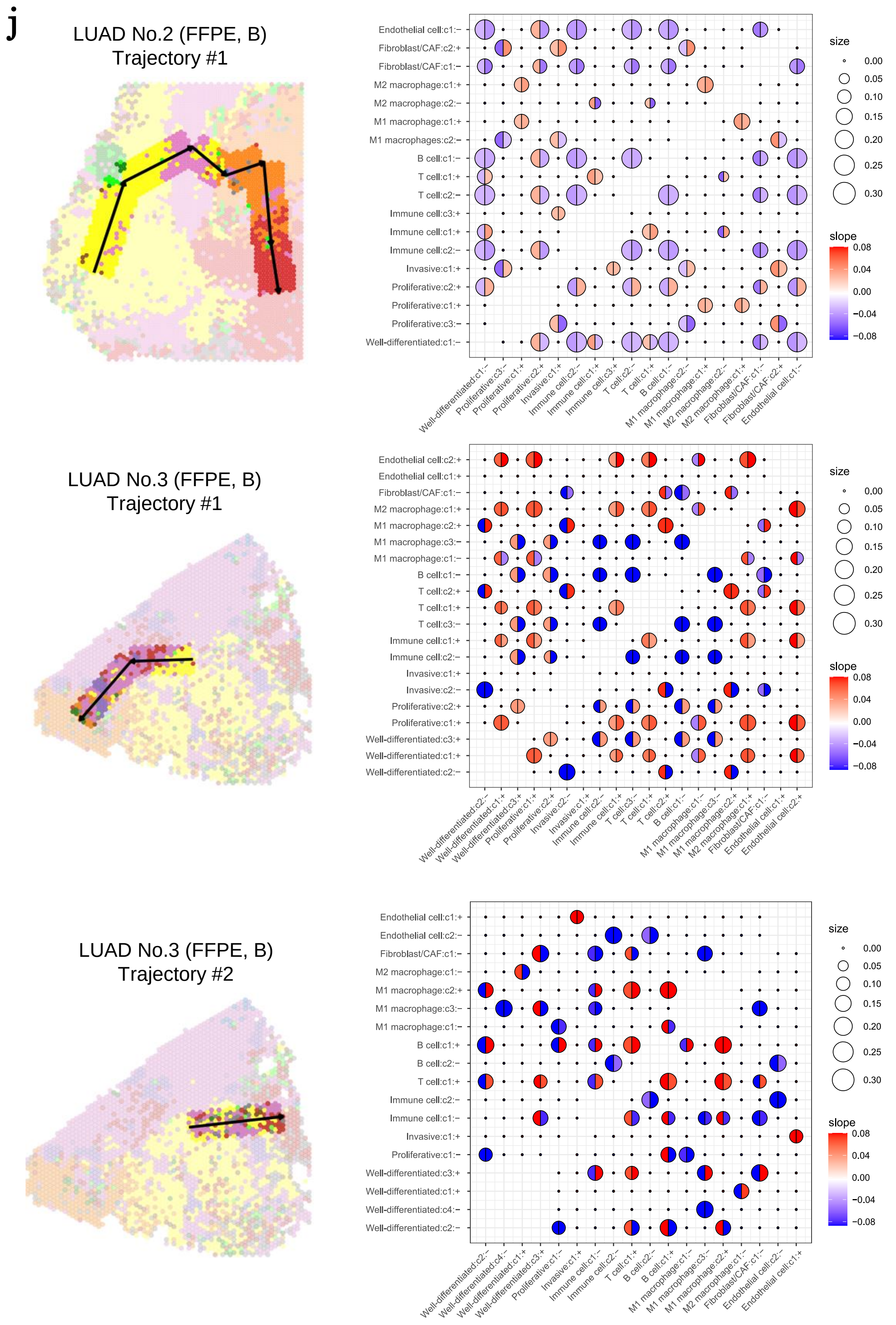
j
LUAD No.2 (FFPE, B)
Trajectory #1
Endothelial cell:c1:−
Fibroblast/CAF:c2:+
Fibroblast/CAF:c1:−
M2 macrophage:c1:+
M2 macrophage:c2:−
M1 macrophage:c1:+
M1 macrophages:c2:−
B cell:c1:−
T cell:c1:+
T cell:c2:−
Immune cell:c3:+
Immune cell:c1:+
Immune cell:c2:−
Invasive:c1:+
Proliferative:c2:+
Proliferative:c1:+
Proliferative:c3:−
Well-differentiated:c1:−
size
0.00
0.05
0.10
0.15
0.20
0.25
0.30
slope
0.08
0.04
0.00
−0.04
−0.08
LUAD No.3 (FFPE, B)
Trajectory #1
Endothelial cell:c2:+
Endothelial cell:c1:+
Fibroblast/CAF:c1:−
M2 macrophage:c1:+
M1 macrophage:c2:+
M1 macrophage:c3:−
M1 macrophage:c1:−
B cell:c1:−
T cell:c2:+
T cell:c1:+
T cell:c3:−
Immune cell:c1:+
Immune cell:c2:−
Invasive:c1:+
Invasive:c2:−
Proliferative:c2:+
Proliferative:c1:+
Well-differentiated:c3:+
Well-differentiated:c1:+
Well-differentiated:c2:−
size
0.00
0.05
0.10
0.15
0.20
0.25
0.30
slope
0.08
0.04
0.00
−0.04
−0.08
LUAD No.3 (FFPE, B)
Trajectory #2
Endothelial cell:c1:+
Endothelial cell:c2:−
Fibroblast/CAF:c1:−
M2 macrophage:c1:−
M1 macrophage:c2:+
M1 macrophage:c3:−
M1 macrophage:c1:−
B cell:c1:+
B cell:c2:−
T cell:c1:+
Immune cell:c2:−
Immune cell:c1:−
Invasive:c1:+
Proliferative:c1:−
Well-differentiated:c3:+
Well-differentiated:c1:+
Well-differentiated:c4:−
Well-differentiated:c2:−
size
0.00
0.05
0.10
0.15
0.20
0.25
0.30
slope
0.08
0.04
0.00
−0.04
−0.08

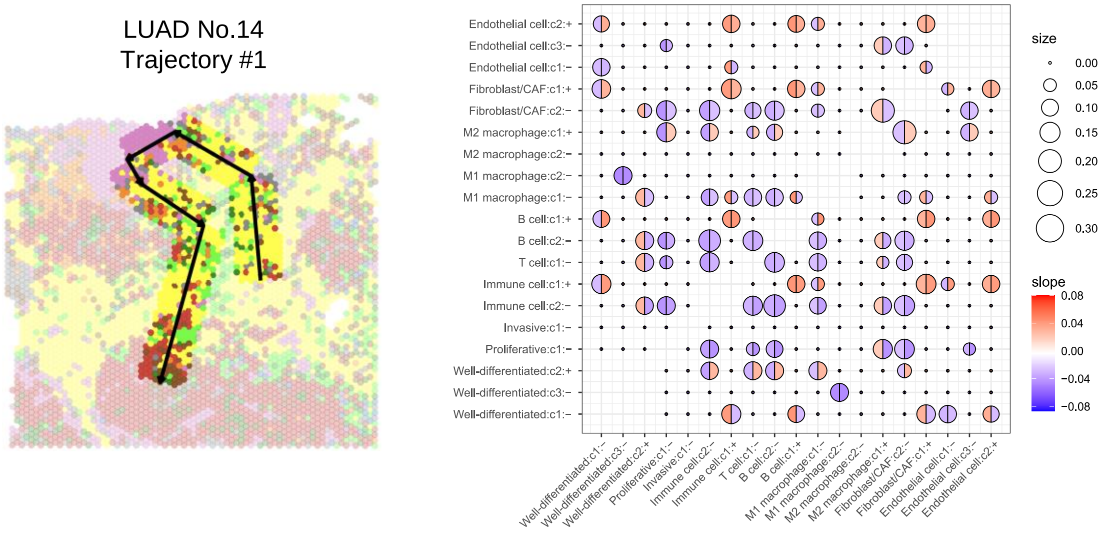
LUAD No.14
Trajectory #1
Endothelial cell:c2:+
Endothelial cell:c3:-
Endothelial cell:c1:-
Fibroblast/CAF:c1:+
Fibroblast/CAF:c2:-
M2 macrophage:c1:+
M2 macrophage:c2:-
M1 macrophage:c2:-
M1 macrophage:c1:-
B cell:c1:+
B cell:c2:-
T cell:c1:-
Immune cell:c1:+
Immune cell:c2:-
Invasive:c1:-
Proliferative:c1:-
Well-differentiated:c2:+
Well-differentiated:c3:-
Well-differentiated:c1:-
Well-differentiated:c1:-
Well-differentiated:c3:-
Well-differentiated:c2:+
Proliferative:c1:-
Invasive:c1:-
Immune cell:c2:-
Immune cell:c1:+
T cell:c1:-
B cell:c2:-
B cell:c1:+
M1 macrophage:c1:-
M1 macrophage:c2:-
M2 macrophage:c2:-
M2 macrophage:c1:+
Fibroblast/CAF:c2:-
Fibroblast/CAF:c1:+
Endothelial cell:c1:-
Endothelial cell:c3:-
Endothelial cell:c2:+
size
0.00
0.05
0.10
0.15
0.20
0.25
0.30
slope
0.08
0.04
0.00
−0.04
−0.08

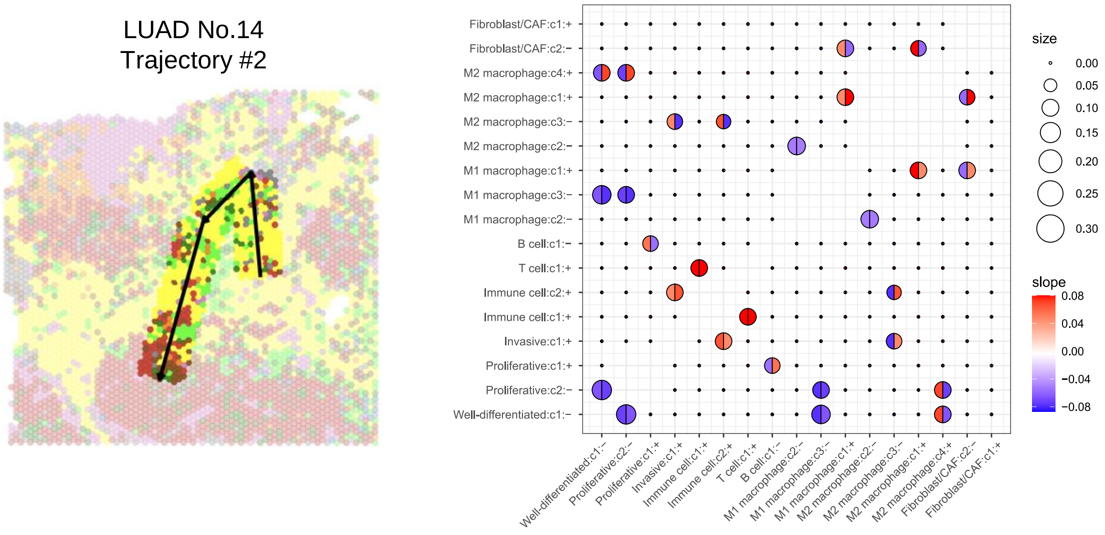
LUAD No.14
Trajectory #2
Fibroblast/CAF:c1:+
Fibroblast/CAF:c2:-
M2 macrophage:c4:+
M2 macrophage:c1:+
M2 macrophage:c3:-
M2 macrophage:c2:-
M1 macrophage:c1:+
M1 macrophage:c3:-
M1 macrophage:c2:-
B cell:c1:-
T cell:c1:+
Immune cell:c2:+
Immune cell:c1:+
Invasive:c1:+
Proliferative:c1:+
Proliferative:c2:-
Well-differentiated:c1:-
Well-differentiated:c1:-
Proliferative:c2:-
Proliferative:c1:+
Invasive:c1:+
Immune cell:c1:+
Immune cell:c2:+
T cell:c1:+
B cell:c1:-
M1 macrophage:c2:-
M1 macrophage:c3:-
M1 macrophage:c1:+
M2 macrophage:c2:-
M2 macrophage:c3:-
M2 macrophage:c1:+
M2 macrophage:c4:+
Fibroblast/CAF:c2:-
Fibroblast/CAF:c1:+
size
0.00
0.05
0.10
0.15
0.20
0.25
0.30
slope
0.08
0.04
0.00
−0.04
−0.08

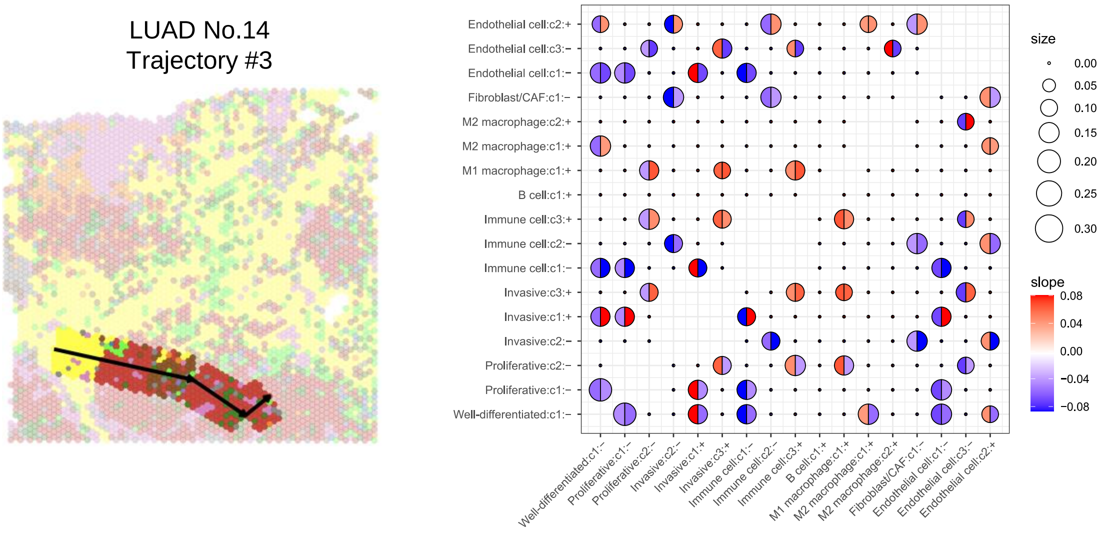
LUAD No.14
Trajectory #3
Endothelial cell:c2:+
Endothelial cell:c3:-
Endothelial cell:c1:-
Fibroblast/CAF:c1:-
M2 macrophage:c2:+
M2 macrophage:c1:+
M1 macrophage:c1:+
B cell:c1:+
Immune cell:c3:+
Immune cell:c2:-
Immune cell:c1:-
Invasive:c3:+
Invasive:c1:+
Invasive:c2:-
Proliferative:c2:-
Proliferative:c1:-
Well-differentiated:c1:-
Well-differentiated:c1:-
Proliferative:c1:-
Proliferative:c2:-
Invasive:c2:-
Invasive:c1:+
Invasive:c3:+
Immune cell:c1:-
Immune cell:c2:-
Immune cell:c3:+
B cell:c1:+
M1 macrophage:c1:+
M2 macrophage:c1:+
M2 macrophage:c2:+
Fibroblast/CAF:c1:-
Endothelial cell:c1:-
Endothelial cell:c3:-
Endothelial cell:c2:+
size
0.00
0.05
0.10
0.15
0.20
0.25
0.30
slope
0.08
0.04
0.00
−0.04
−0.08

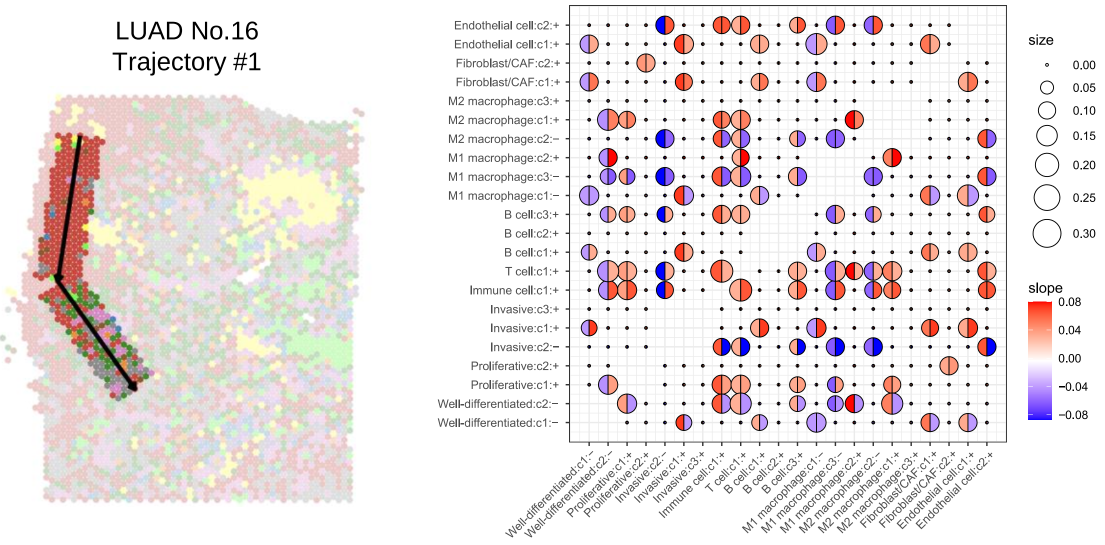


**Supplementary Figure S8 Evaluation of changes of TME scores in IA cases**

(**a**) A workflow for TME scoring to evaluate TME changes in IA cases. (**b**, **c**) TME scores and spatial transcriptome trajectory in LUAD No. 2 FFPE section B. (**b**) The most dominant TMEs in each spot were colored according to the color legend. (**c**) The spatial distribution of the scores is shown in each TME (top). The changes in the TME score and the trajectory are shown on dot plots (bottom). Smoothened lines are shown in red. (**d**, **e**) TME scores and two spatial transcriptome trajectories in LUAD No. 3 FFPE section B are presented as in **b** and **c**. (**f**, **g**) TME scores and three spatial transcriptome trajectories in LUAD No. 14 FF are shown as in **b** and **c**. (**h**, **i**) TME scores and spatial transcriptome trajectory in LUAD No. 16 FF as shown in **b** and **c**. (**j**) Relationships between each TME pair. Source data are provided as a Source Data file for **j**.

*Supplementary Figure S9*

**a**

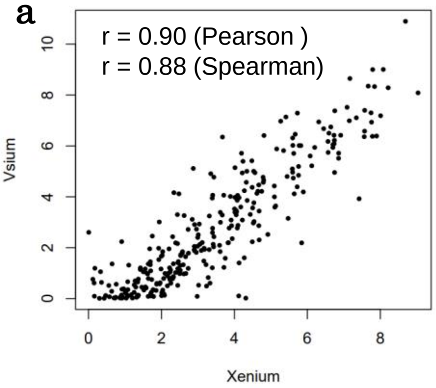


**b**

LUAD No.3 FFPE

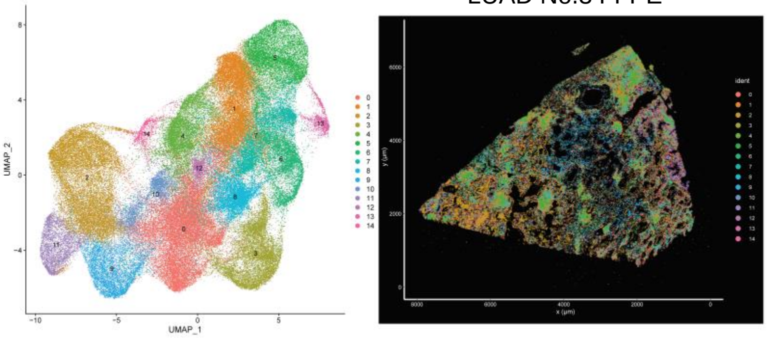


| Cluster | Annotation |
|---|---|
| 0 | Fibroblast |
| 1 | T cell, B cell |
| 2 | Proliferative tumor cell |
| 3 | Endothelial cell |
| 4 | Macrophage |
| 5 | CXCL13+ B cell |
| 6 | LEC |
| 7 | CXCL9+ T cell |
| 8 | Plasma cell |
| 9 | Differentiated tumor cell |
| 10 | VSMC, myofibroblast |
| 11 | Invasive tumor cell |
| 12 | Mastocyte |
| 13 | CCL22+ DC |
| 14 | SPP1+ macrophage |

LUAD No.14 FF

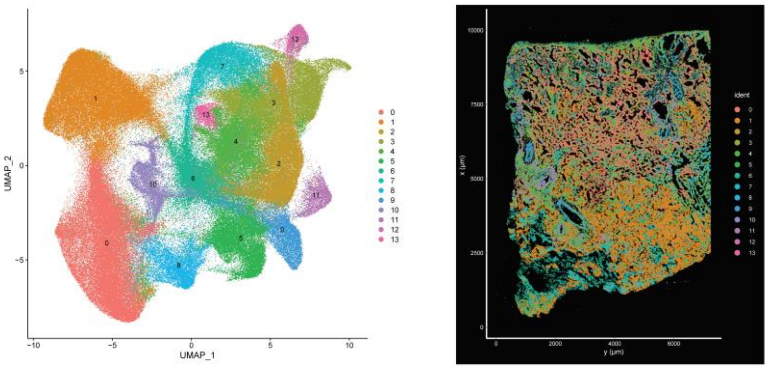


| Cluster | Annotation |
|---|---|
| 0 | Differentiated tumor cell |
| 1 | Invasive/hypoxic tumor cell |
| 2 | Fibroblast |
| 3 | Macrophage |
| 4 | T cell |
| 5 | Endothelial cell |
| 6 | Others |
| 7 | SPP1+ macrophage |
| 8 | Differentiated tumor cell |
| 9 | VSMC, myofibroblast |
| 10 | Bronchiolar epithelial cell |
| 11 | LEC |
| 12 | Plasma cell |
| 13 | CXCL13+ T cell |

LUAD No.16 FF

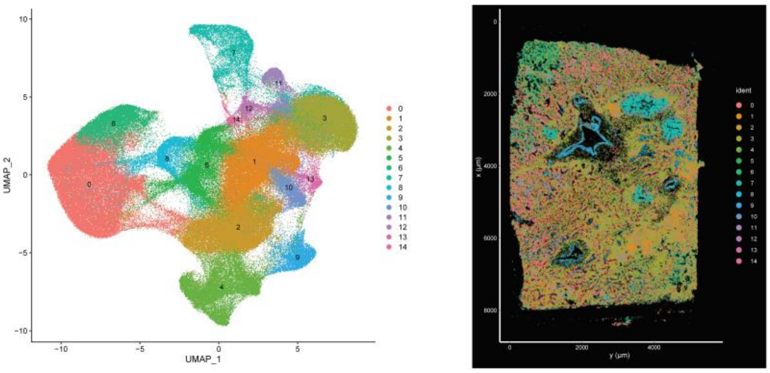


| Cluster | Annotation |
|---|---|
| 0 | Invasive tumor cell |
| 1 | T cell |
| 2 | Fibroblast |
| 3 | Others |
| 4 | Endothelial cell |
| 5 | DC |
| 6 | Differentiated tumor cell |
| 7 | Bronchiolar epithelial cell |
| 8 | Macrophage |
| 9 | VSMC, myofibroblast |
| 10 | CCL22+ immune cell |
| 11 | Mastocyte |
| 12 | Plasma cell |
| 13 | LEC |
| 14 | pDC |

LUAD No.17 FF

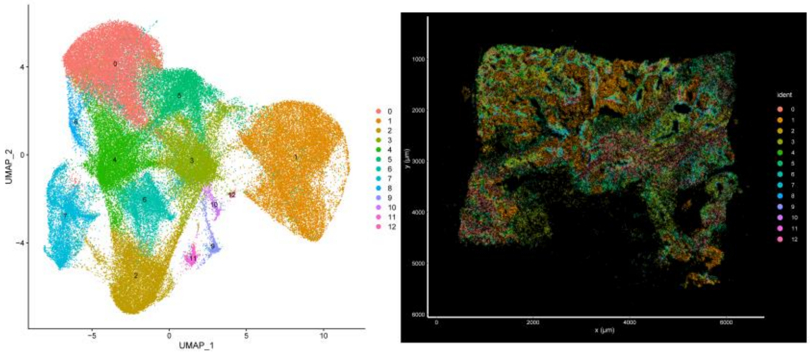


| Cluster | Annotation |
|---|---|
| 0 | Fibroblast |
| 1 | Tumor cell |
| 2 | Plasma cell |
| 3 | Others |
| 4 | DC, macrophage |
| 5 | SPP1+ macrophage |
| 6 | T cell |
| 7 | Endothelial cell |
| 8 | IDO1+ fibroblast |
| 9 | LEC |
| 10 | Mastocyte |
| 11 | CXCL13+ T cell |
| 12 | Invasive tumor cell |

**c**

LUAD No. 3 (FFPE, A) LUAD No. 3 (FFPE, B)

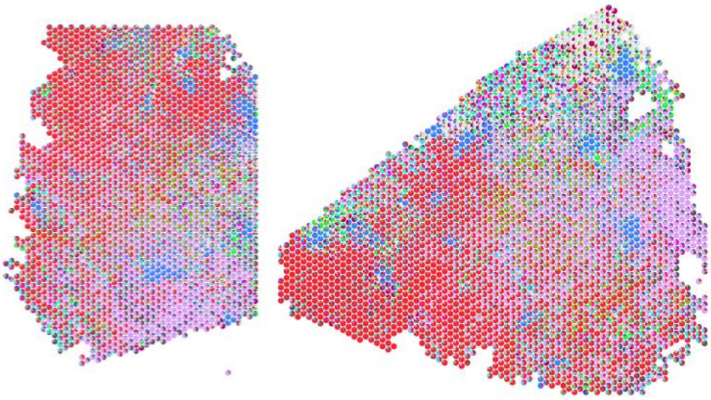

Cell types (clusters of Xenium) in LUAD No. 3 FFPE

| | |
|---|---|
| 0 | Fibroblast |
| 1 | T cell, B cell |
| 2 | Proliferative tumor cell |
| 3 | Endothelial cell |
| 4 | Macrophage |
| 5 | CXCL13+ B cell |
| 6 | LEC |
| 7 | CXCL9+ T cell |
| 8 | Plasma cell |
| 9 | Differentiated tumor cell |
| 10 | VSMC, myofibroblast |
| 11 | Invasive tumor cell |
| 12 | Mastocyte |
| 13 | CCL22+ DC |
| 14 | SPP1+ macrophage |

LUAD No. 14 (FF)

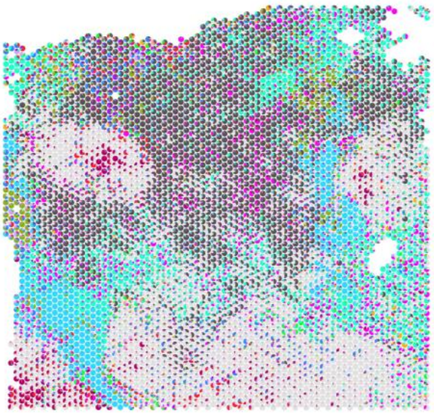

Cell types (clusters of Xenium) in LUAD No. 14 FF

| | |
|---|---|
| 0 | Differentiated tumor cell |
| 1 | Invasive/hypoxic tumor cell |
| 2 | Fibroblast |
| 3 | Macrophage |
| 4 | T cell |
| 5 | Endothelial cell |
| 6 | Others |
| 7 | SPP1+ macrophage |
| 8 | Differentiated tumor cell |
| 9 | VSMC, myofibroblast |
| 10 | Bronchiolar epithelial cell |
| 11 | LEC |
| 12 | Plasma cell |
| 13 | CXCL13+ T cell |

## LUAD No. 16 (FF)

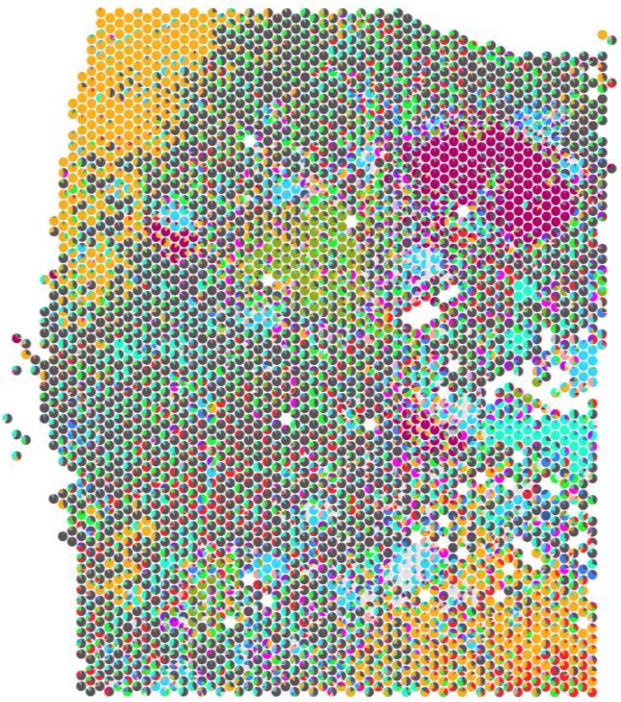

### Cell types (clusters of Xenium) in LUAD No. 16 FF

| | |
|---|---|
| 0 | Invasive tumor cell |
| 1 | T cell |
| 2 | Fibroblast |
| 3 | Others |
| 4 | Endothelial cell |
| 5 | DC |
| 6 | Differentiated tumor cell |
| 7 | Bronchiolar epithelial cell |
| 8 | Macrophage |
| 9 | VSMC, myofibroblast |
| 10 | CCL22+ immune cell |
| 11 | Mastocyte |
| 12 | Plasma cell |
| 13 | LEC |
| 14 | pDC |

## LUAD No. 17 (FF)

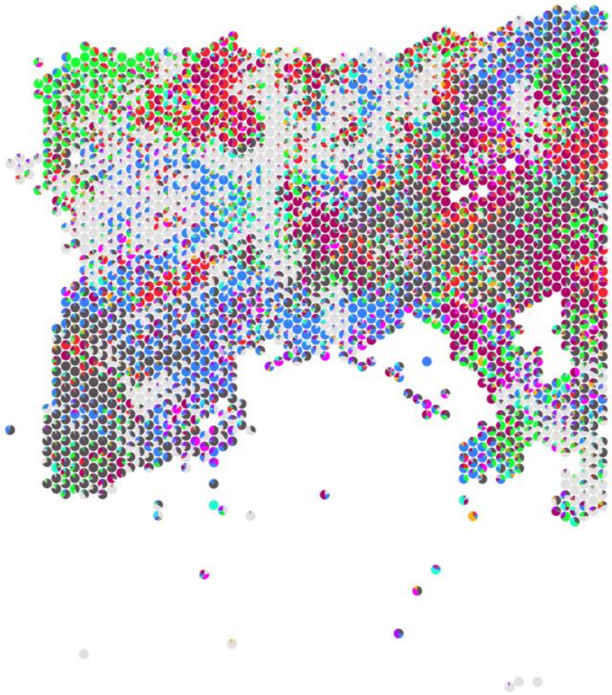

### Cell types (clusters of Xenium) in LUAD No. 17 FF

| | |
|---|---|
| 0 | Fibroblast |
| 1 | Tumor cell |
| 2 | Plasma cell |
| 3 | Others |
| 4 | DC, macrophage |
| 5 | SPP1+ macrophage |
| 6 | T cell |
| 7 | Endothelial cell |
| 8 | IDO1+ fibroblast |
| 9 | LEC |
| 10 | Mastocyte |
| 11 | CXCL13+ T cell |
| 12 | Invasive tumor cell |

**d**

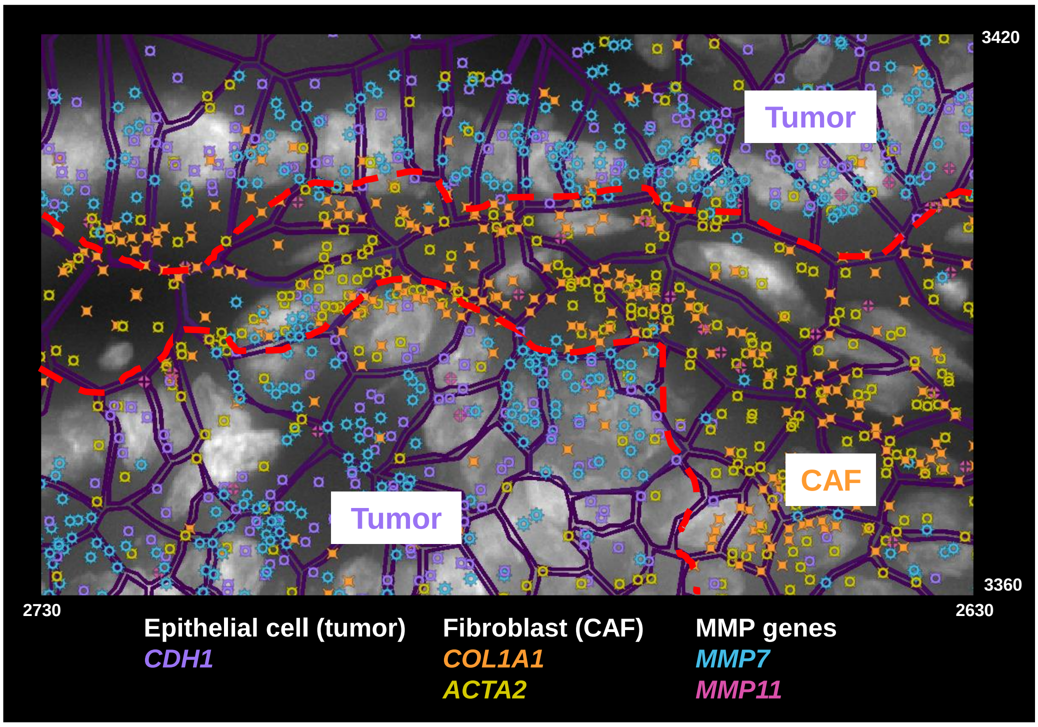

Tumor
Tumor
CAF
3420
3360
2730
2630
Epithelial cell (tumor)
CDH1
Fibroblast (CAF)
COL1A1
ACTA2
MMP genes
MMP7
MMP11


**e**

**(i)**

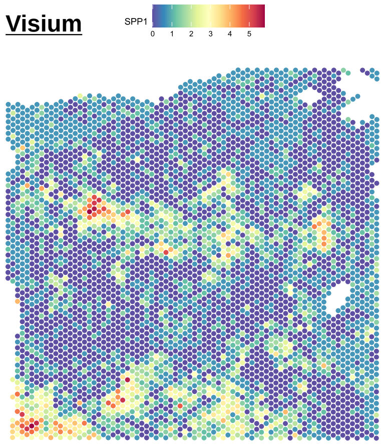

Visium
SPP1
0 1 2 3 4 5


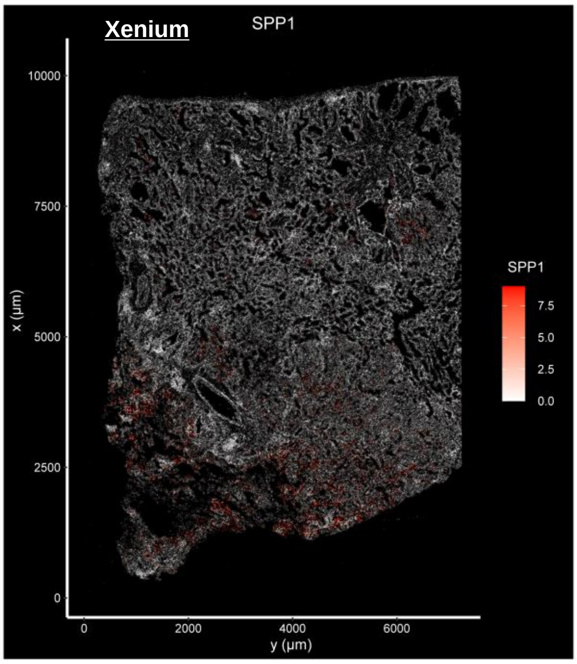

Xenium
SPP1
10000
7500
5000
2500
0
x (µm)
0
2000
4000
6000
y (µm)
SPP1
7.5
5.0
2.5
0.0

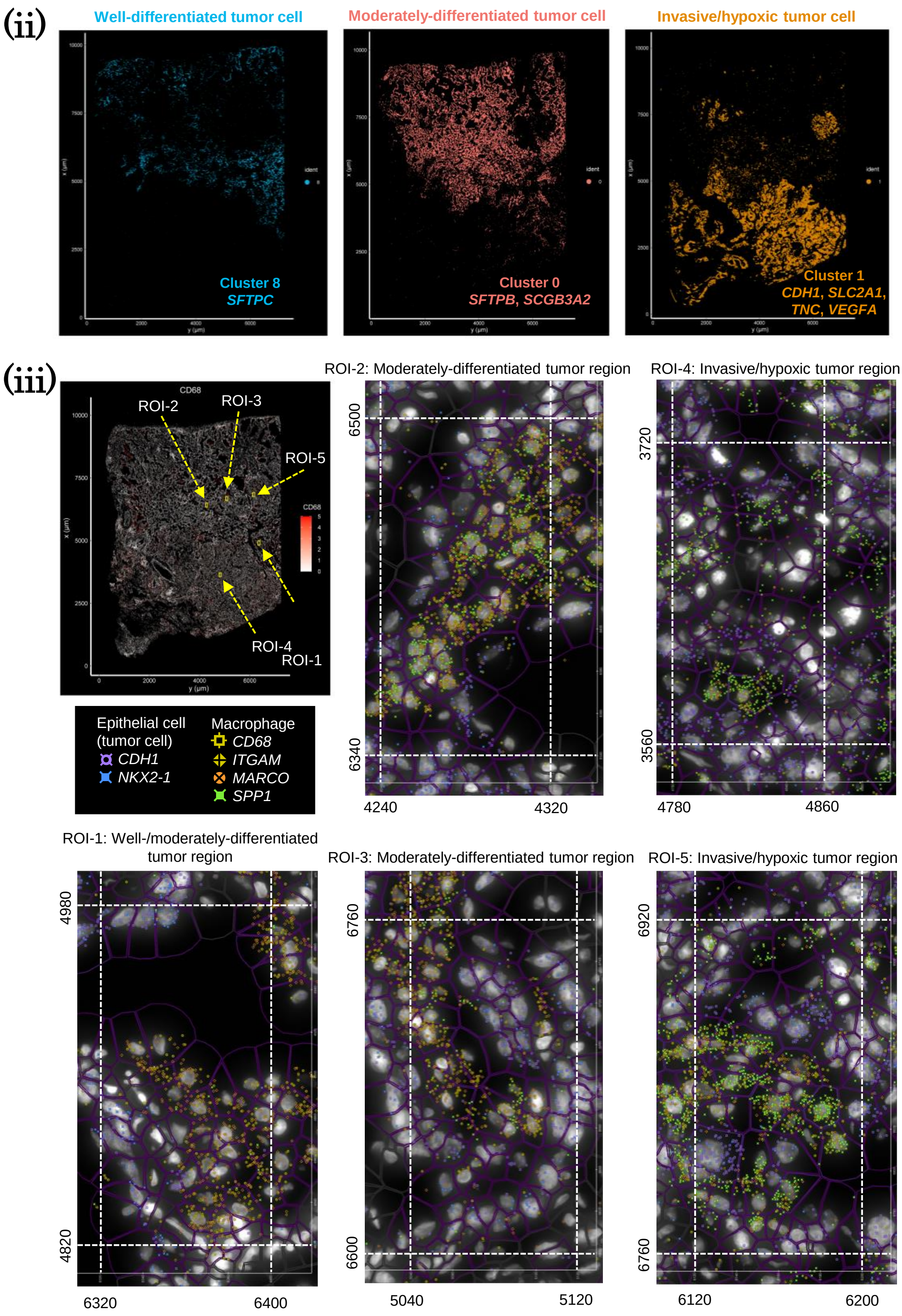
(ii)
Well-differentiated tumor cell
Cluster 8
SFTPC
Moderately-differentiated tumor cell
Cluster 0
SFTPB, SCGB3A2
Invasive/hypoxic tumor cell
Cluster 1
CDH1, SLC2A1, TNC, VEGFA
(iii)
CD68
ROI-2
ROI-3
ROI-5
ROI-4
ROI-1
Epithelial cell (tumor cell)
CDH1
NKX2-1
Macrophage
CD68
ITGAM
MARCO
SPP1
ROI-2: Moderately-differentiated tumor region
6500
6340
4240
4320
ROI-4: Invasive/hypoxic tumor region
3720
3560
4780
4860
ROI-1: Well-/moderately-differentiated tumor region
4980
4820
6320
6400
ROI-3: Moderately-differentiated tumor region
6760
6600
5040
5120
ROI-5: Invasive/hypoxic tumor region
6920
6760
6120
6200

**Supplementary Figure S9 Spatial and single-cell expression analysis using Xenium for five IA cases**

(**a**) A comparison of expression levels (aggregated bulk) in Visium and Xenium of LUAD No.2 FFPE. Each dot represents one gene. (**b**) Clustering analysis results for Xenium of LUAD No. 3 FFPE, No. 14, 16, and 17 (the result of LUAD No. 2 FFPE is shown in **Figure 6**). The left and middle panels show the UMAP plot with cluster annotations, as well as the spatial plot. The annotation for each cluster is shown in the table in the right panel, respectively. (**c**) Deconvolution of Visium using Xenium data. On the left, the spatial plot of Visium is accompanied by pie charts showing the proportion of cell types. On the right, the table presents information of each cell type (Xenium cluster). (**d**) Association between tumor cells and adjacent CAFs at the single-cell level in the invasive region (as shown in **Figure 6f**). MMP represents matrix metalloproteinase. (**e**) Characterization of macrophages in LUAD No. 14 FF. (i) The expression pattern of *SPP1* is shown in Visium and Xenium data in the left and right panels, respectively. (ii) The spatial distribution of clusters 8 (well-differentiated tumor cell), 0 (moderately-differentiated tumor region), and 1 (invasive/hypoxic tumor region). (iii) The distribution of macrophages is shown by Xenium Explorer. Macrophages expressing high levels of *SPP1* increased in invasive tumor regions.

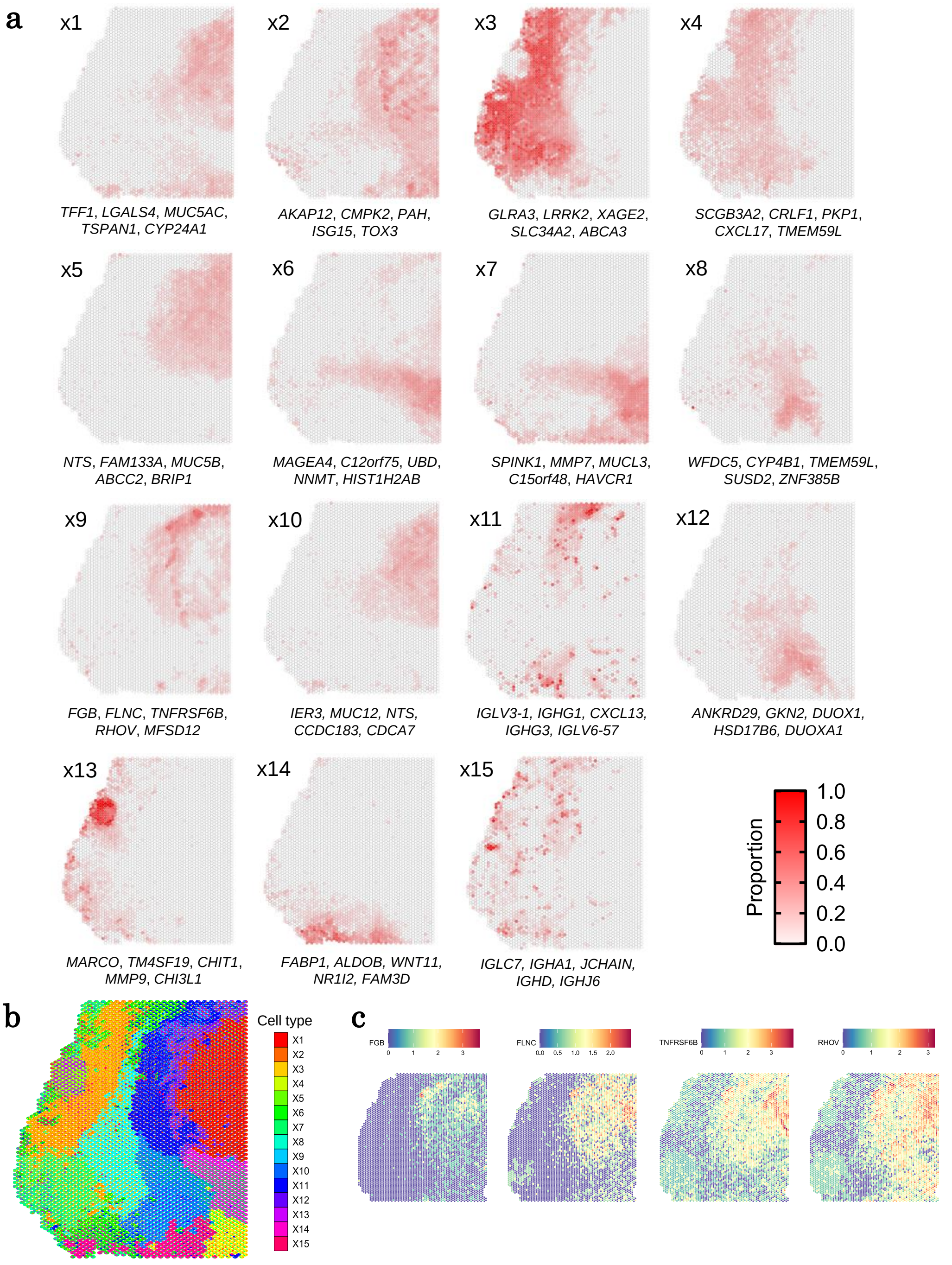


**Supplementary Figure S10 Reference-free cell-type deconvolution analysis of Visium**

(**a**) Spatial plots of cell proportions for 15 cell-types in LUAD No. 2 FFPE section C, obtained using reference-free cell-type deconvolution analysis[4]. (**b**) The spatial plot of Visium is presented using pie charts showing the proportion of the 15 cell-types. (**c**) Expression patterns of genes specific to the cell-type X9 in Visium data.

*Supplementary Figure S11*

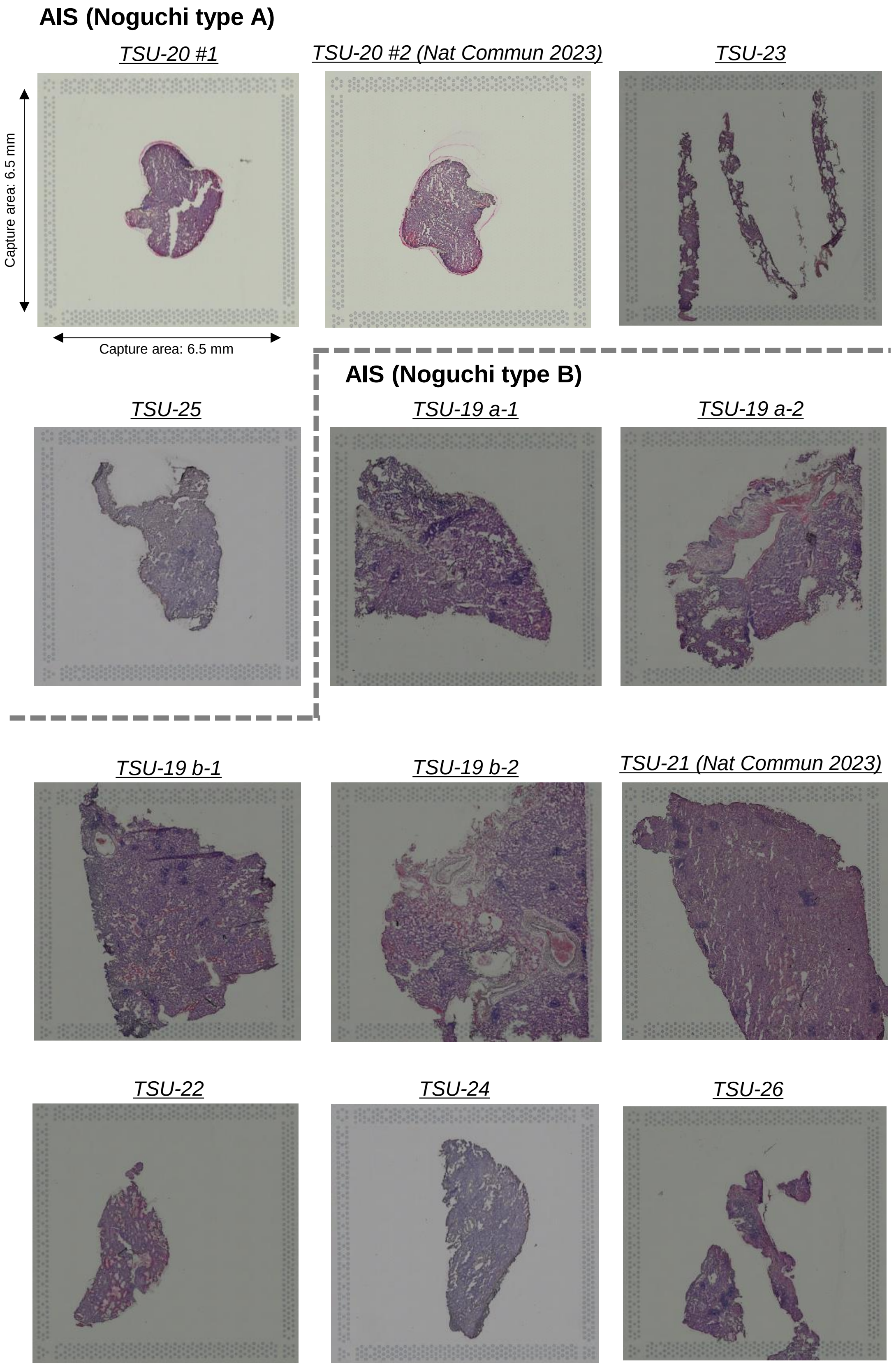

*Supplementary Figure S11*

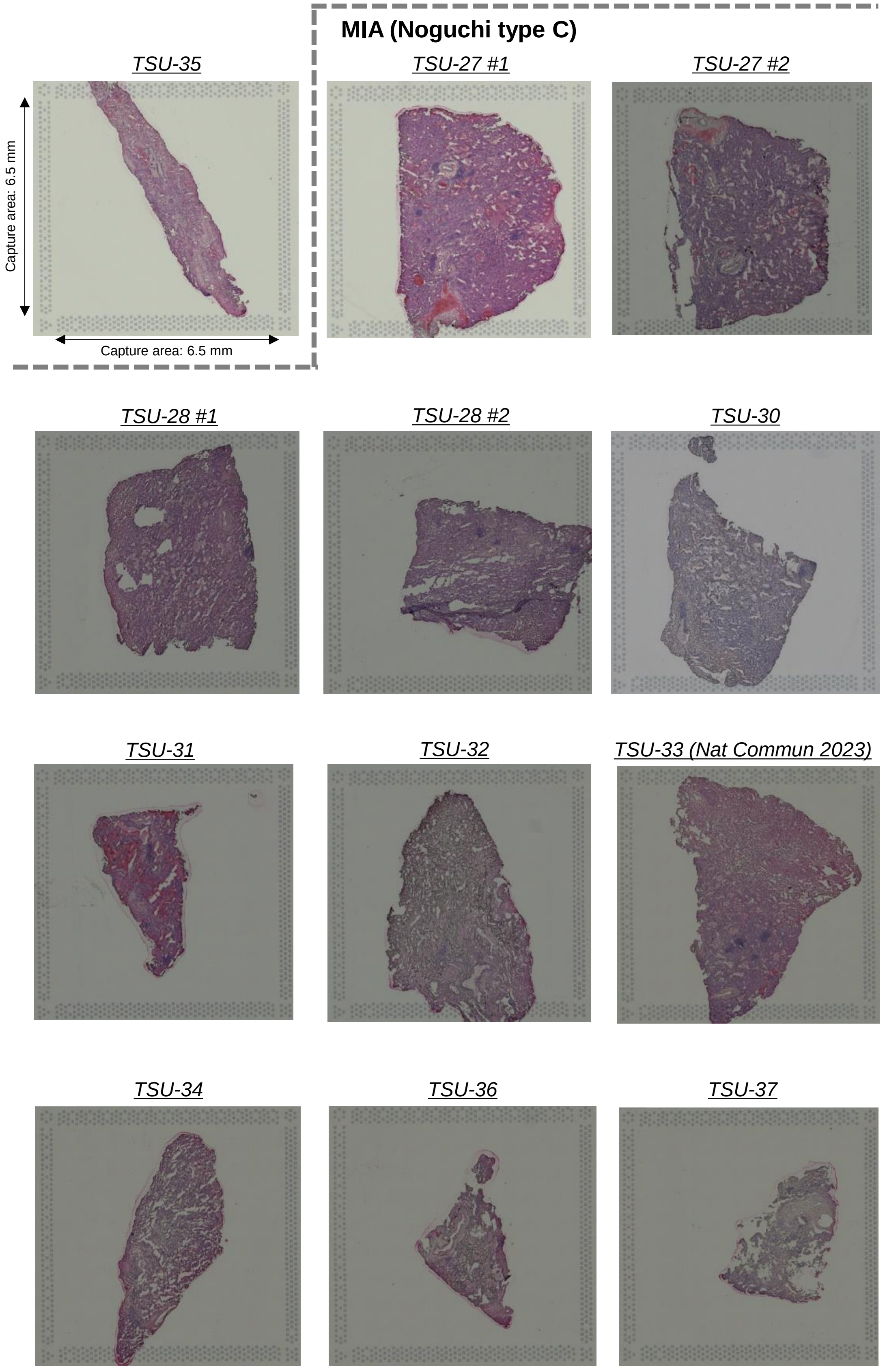

*Supplementary Figure S11*

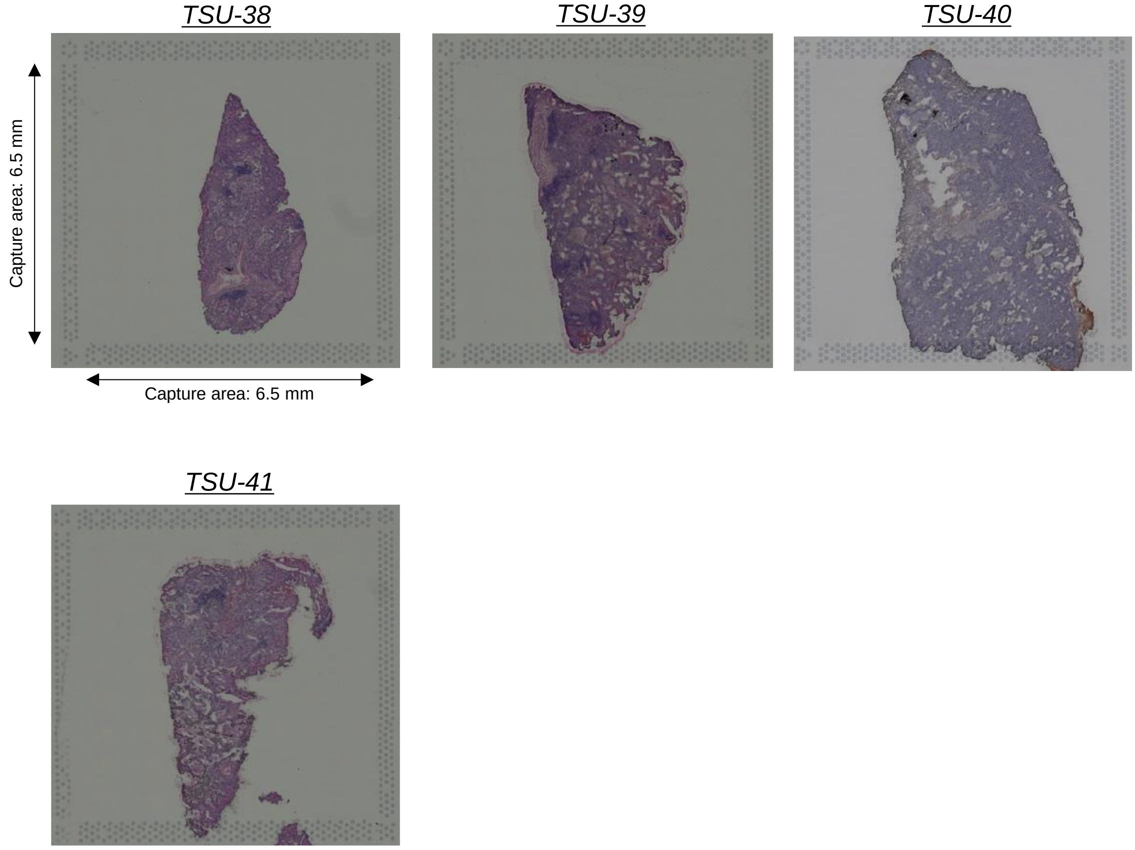


**Supplementary Figure S11 H&E images of Visium FF data in 22 early cases**

H&E images for Visium analysis. The study presents data from 28 sections taken from patients with adenocarcinoma *in situ* (AIS) and minimally invasive adenocarcinoma (MIA). The capture area that is surrounded by the fiducial frame in the H&E is 6.5 mm × 6.5 mm.

## AIS (Noguchi type A)

*TSU-20 #1*

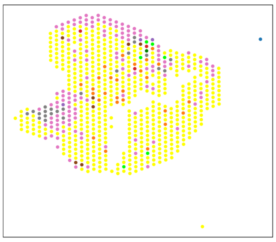

*TSU-20 #2 (Nat Commun 2023)*

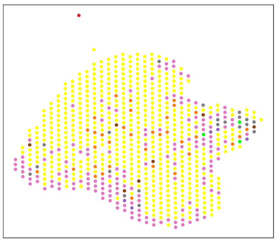

*TSU-23*

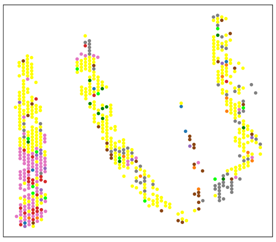

*TSU-25*

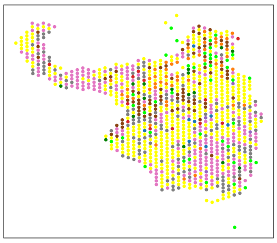

## AIS (Noguchi type B)

*TSU-19 a-1*

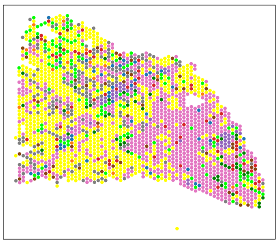

*TSU-19 a-2*

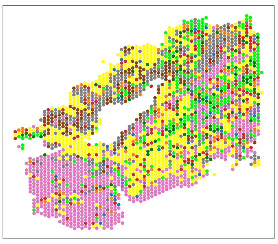

*TSU-19 b-1*

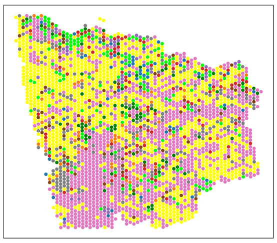

*TSU-19 b-2*

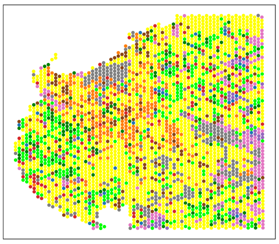

*TSU-21 (Nat Commun 2023)*

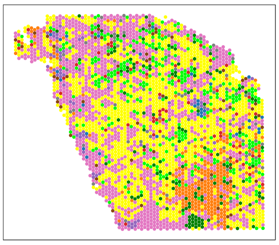

*TSU-22*

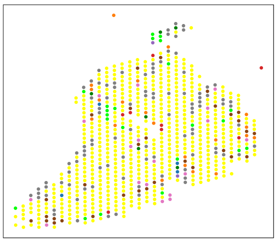

*TSU-24*

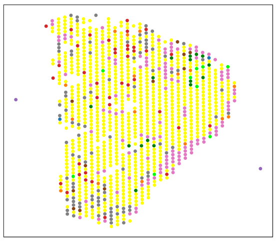

*TSU-26*

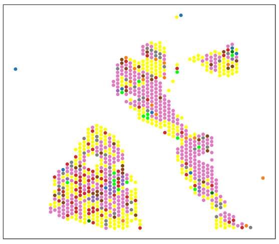

*TSU-35*

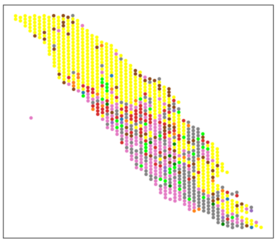

## Early-Ad; MIA (Noguchi type C)

*TSU-27 #1*

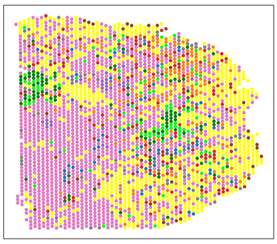

*TSU-27 #2*

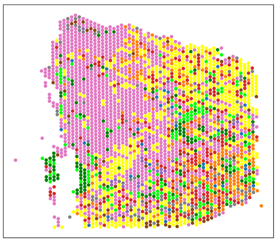

*TSU-28 #1*

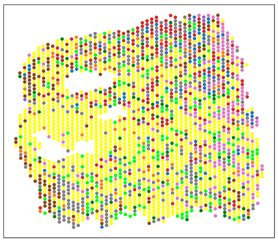

*TSU-28 #2*

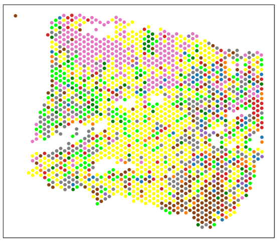

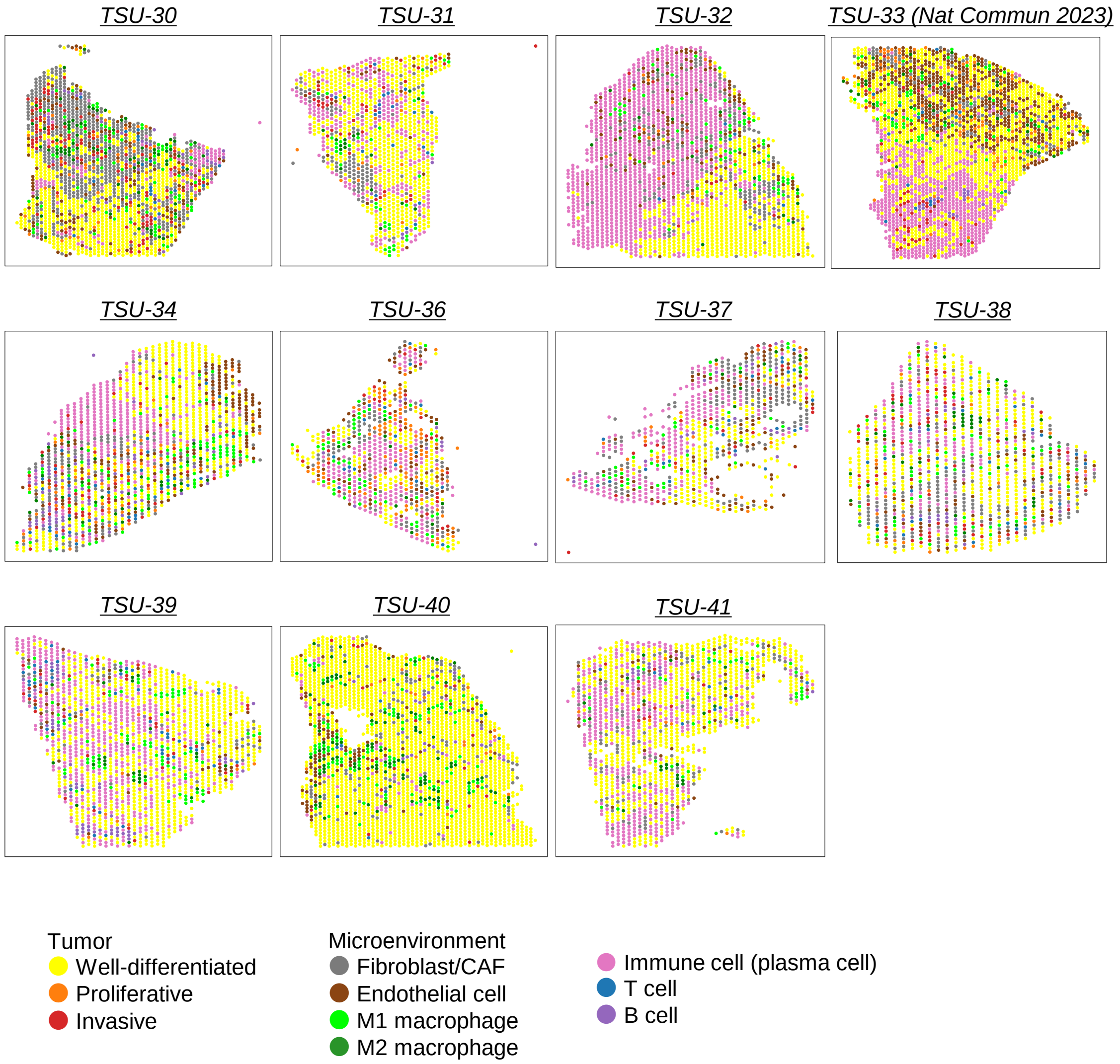


**Supplementary Figure S12 TME scoring in AIS/MIA cases**

Features are arranged according to TME scoring in AIS and MIA cases. The color legend appears in the margin.

**a**

***Step 1. Weighting proliferative/invasive spots by distance for each spot***

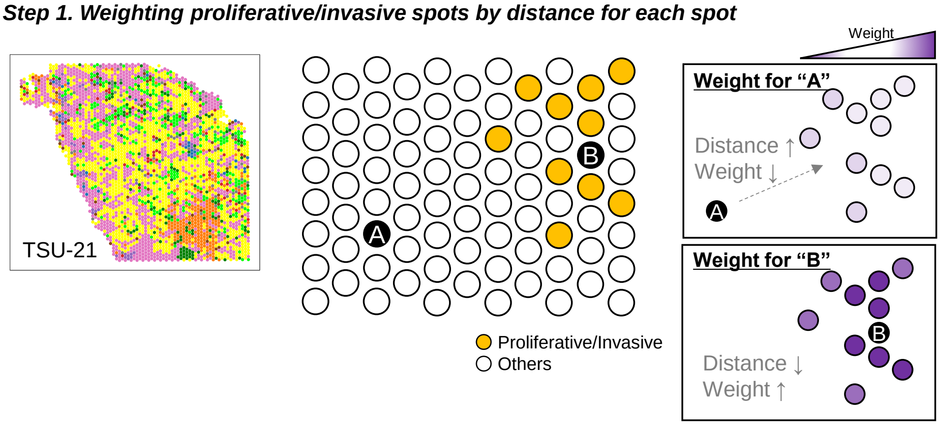


***Step 2. Calculating local enrichment score by summing the weighted counts in all spots***

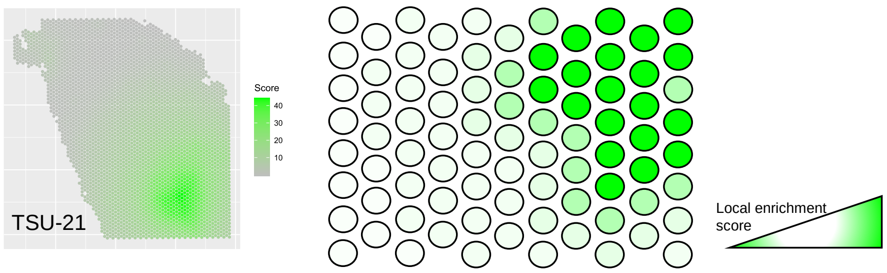


***Step 3. Defining "possibly malignant" regions by gathering spots with high local enrichment scores***

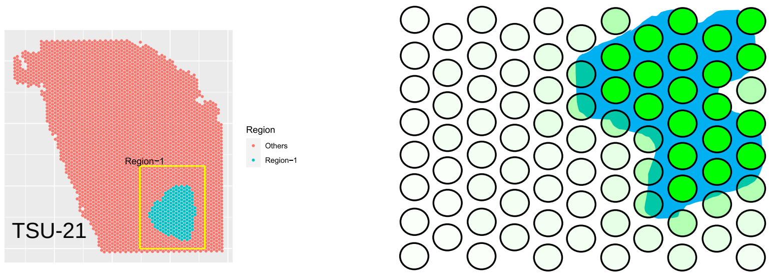


***Step 4. Comparing gene expression patterns between spots in the possibly malignant region and others***

# b

## AIS (Noguchi type A)

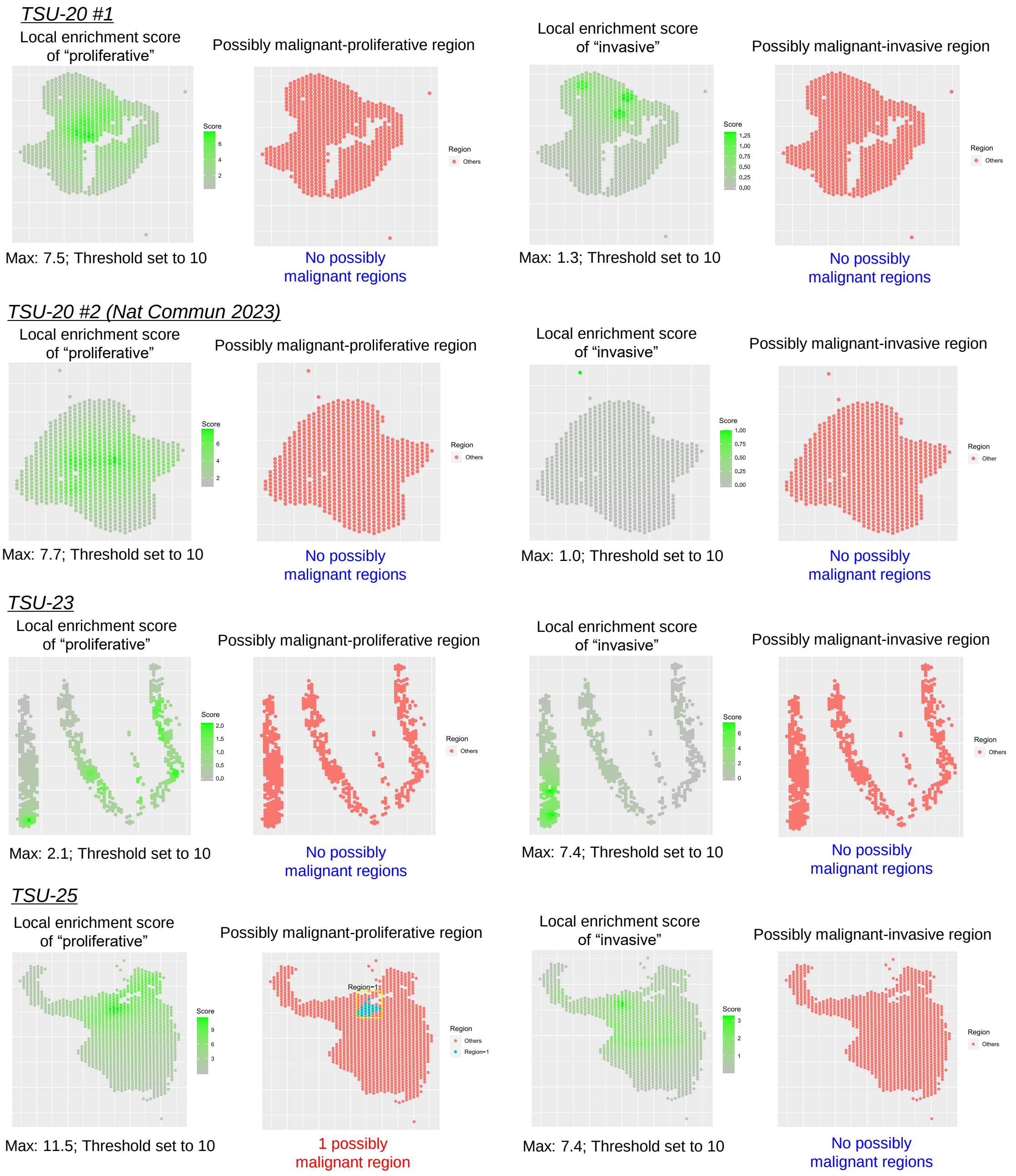

**c**

## AIS (Noguchi type B)

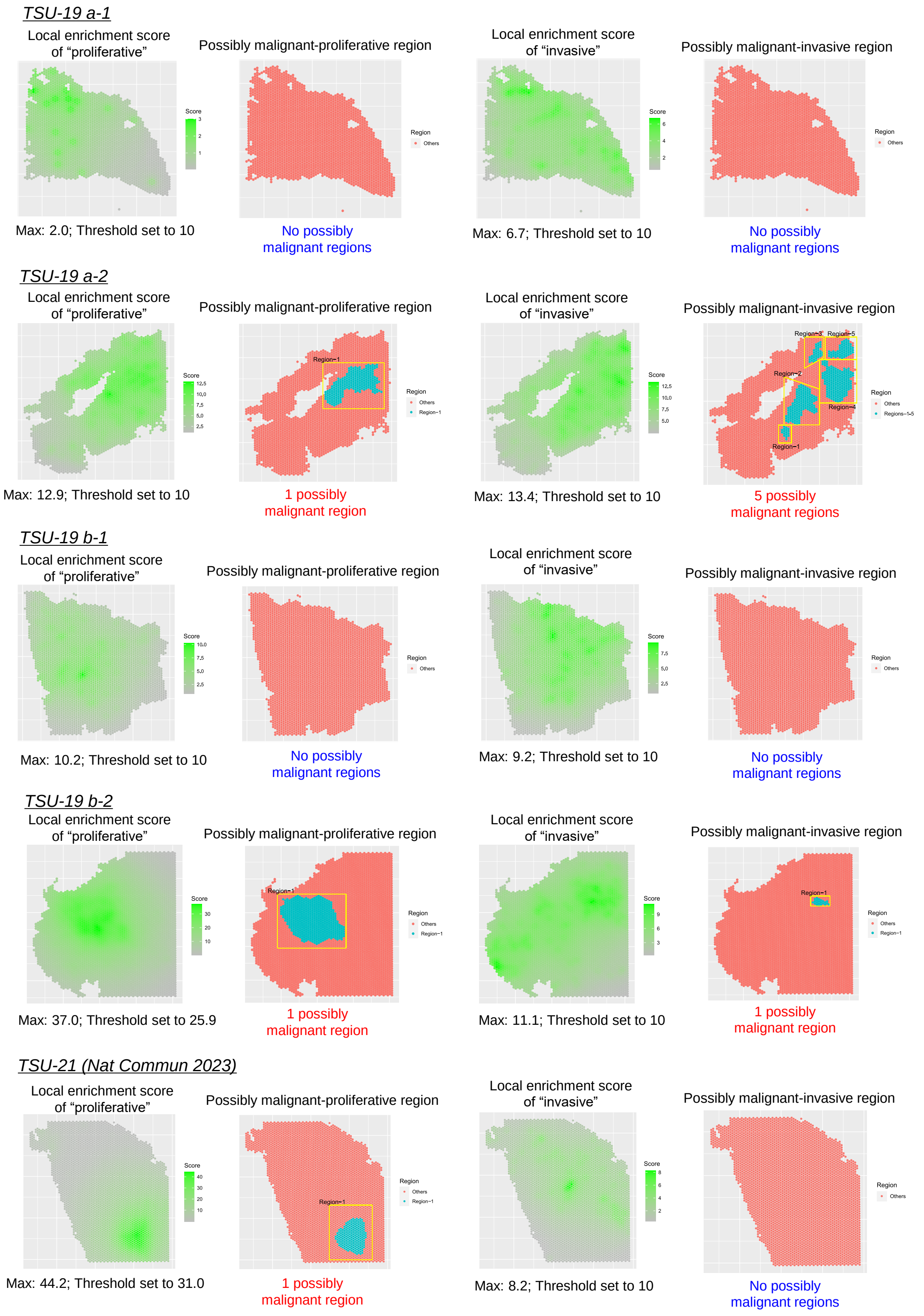

## *Supplementary Figure S13*

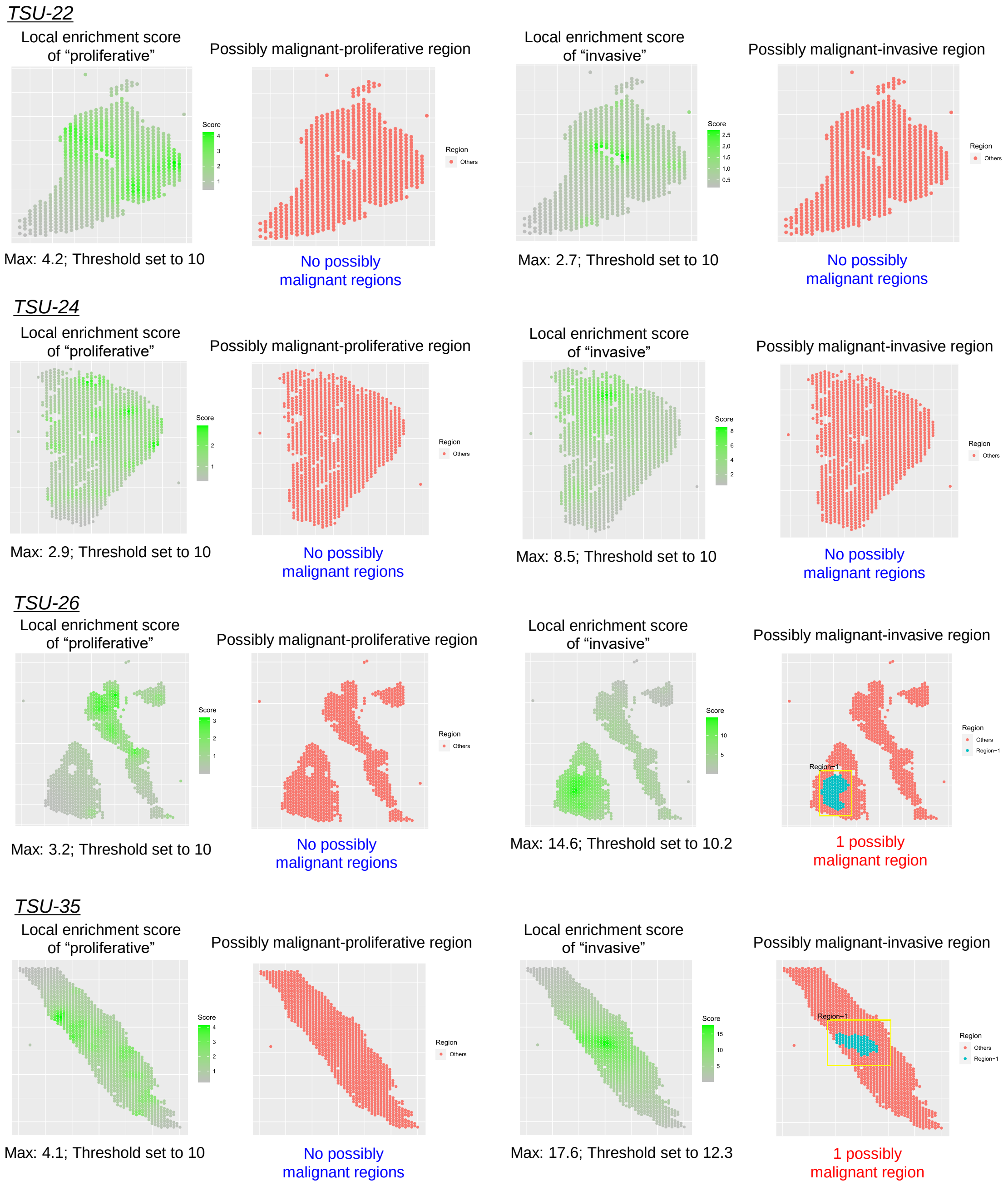

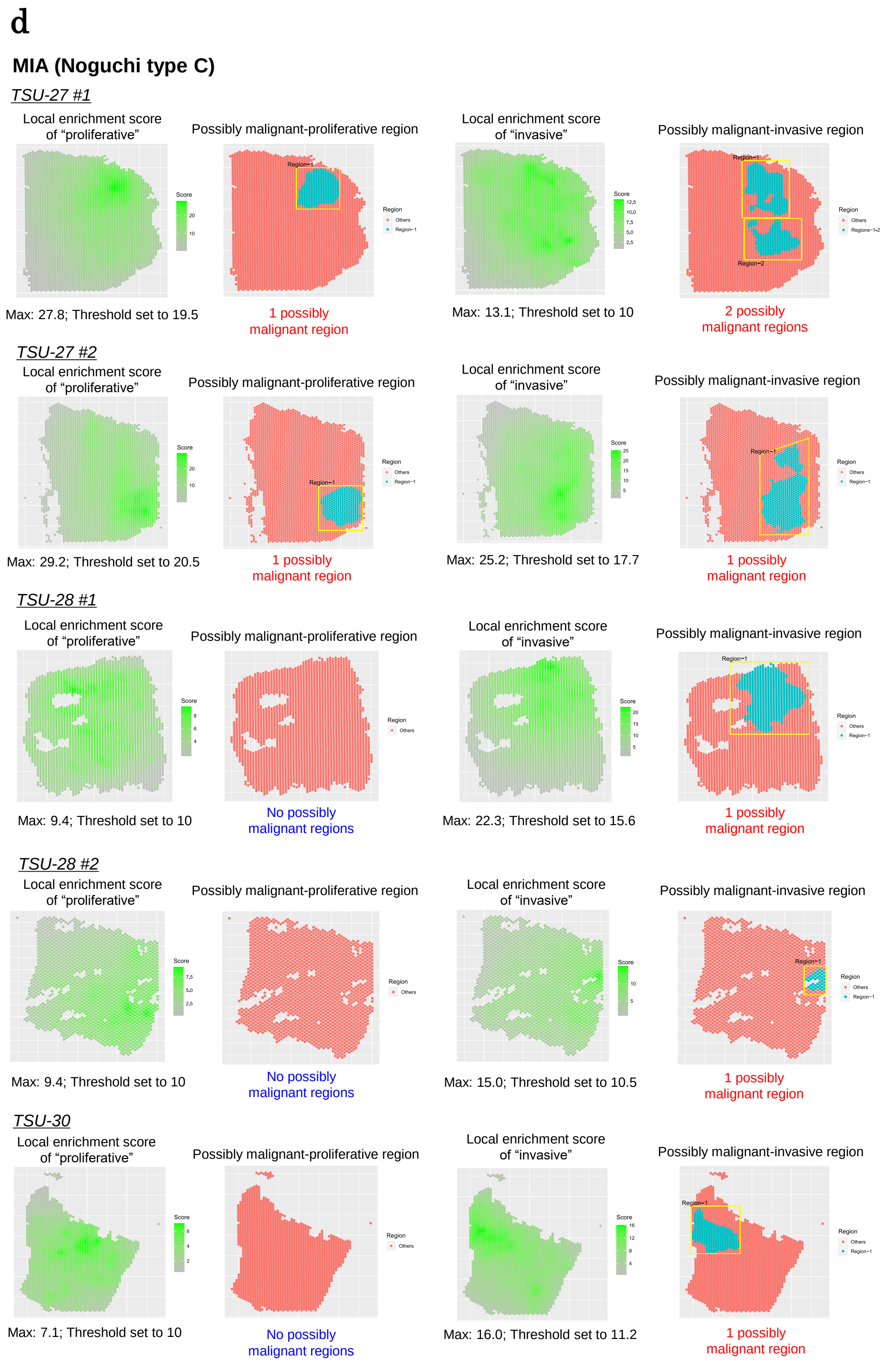
d
MIA (Noguchi type C)
TSU-27 #1
Local enrichment score of “proliferative”
Max: 27.8; Threshold set to 19.5
Possibly malignant-proliferative region
1 possibly malignant region
Local enrichment score of “invasive”
Max: 13.1; Threshold set to 10
Possibly malignant-invasive region
2 possibly malignant regions
TSU-27 #2
Local enrichment score of “proliferative”
Max: 29.2; Threshold set to 20.5
Possibly malignant-proliferative region
1 possibly malignant region
Local enrichment score of “invasive”
Max: 25.2; Threshold set to 17.7
Possibly malignant-invasive region
1 possibly malignant region
TSU-28 #1
Local enrichment score of “proliferative”
Max: 9.4; Threshold set to 10
Possibly malignant-proliferative region
No possibly malignant regions
Local enrichment score of “invasive”
Max: 22.3; Threshold set to 15.6
Possibly malignant-invasive region
1 possibly malignant region
TSU-28 #2
Local enrichment score of “proliferative”
Max: 9.4; Threshold set to 10
Possibly malignant-proliferative region
No possibly malignant regions
Local enrichment score of “invasive”
Max: 15.0; Threshold set to 10.5
Possibly malignant-invasive region
1 possibly malignant region
TSU-30
Local enrichment score of “proliferative”
Max: 7.1; Threshold set to 10
Possibly malignant-proliferative region
No possibly malignant regions
Local enrichment score of “invasive”
Max: 16.0; Threshold set to 11.2
Possibly malignant-invasive region
1 possibly malignant region

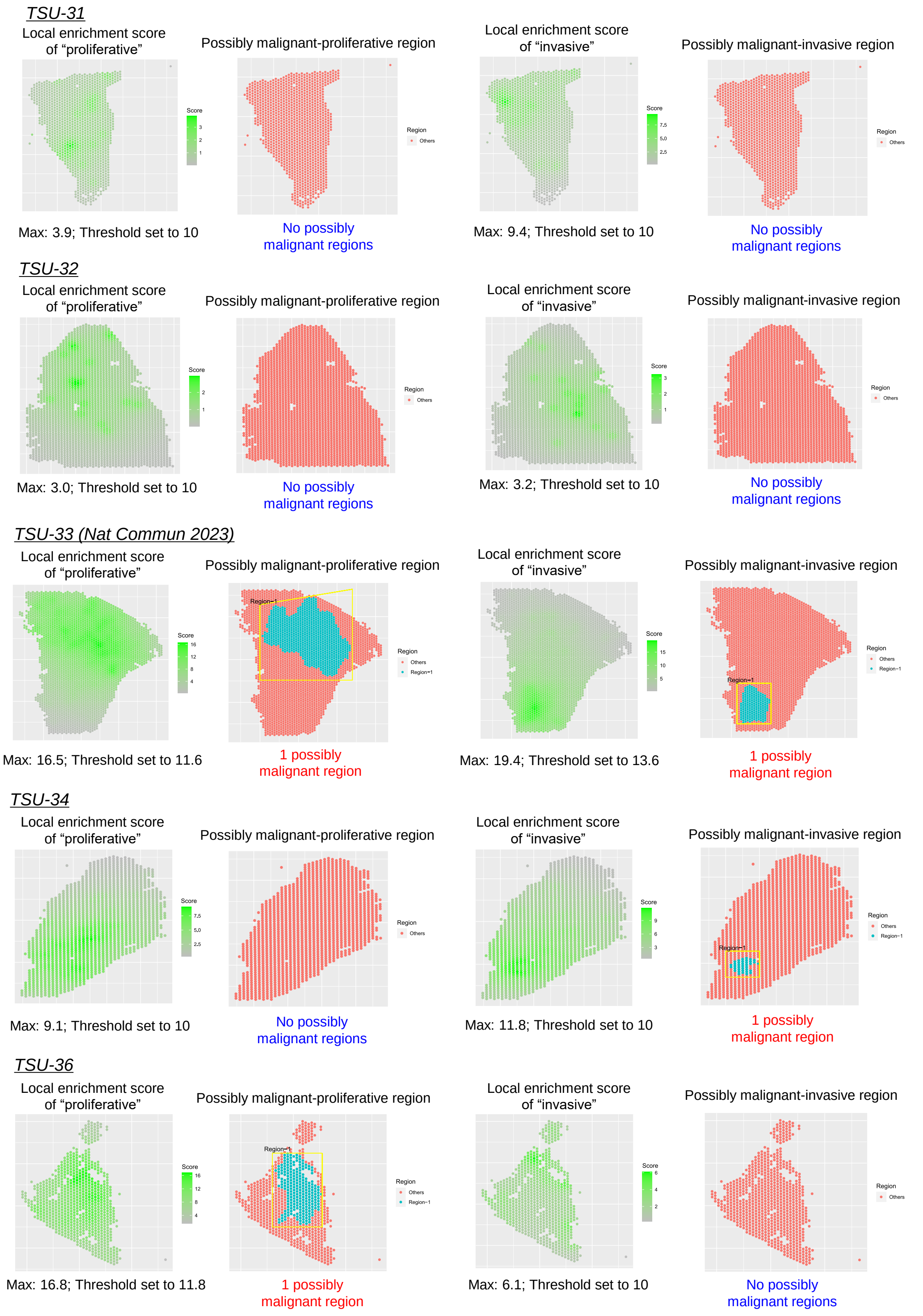
TSU-31
Local enrichment score of "proliferative"
Possibly malignant-proliferative region
Local enrichment score of "invasive"
Possibly malignant-invasive region
Max: 3.9; Threshold set to 10
No possibly malignant regions
Max: 9.4; Threshold set to 10
No possibly malignant regions
TSU-32
Local enrichment score of "proliferative"
Possibly malignant-proliferative region
Local enrichment score of "invasive"
Possibly malignant-invasive region
Max: 3.0; Threshold set to 10
No possibly malignant regions
Max: 3.2; Threshold set to 10
No possibly malignant regions
TSU-33 (Nat Commun 2023)
Local enrichment score of "proliferative"
Possibly malignant-proliferative region
Local enrichment score of "invasive"
Possibly malignant-invasive region
Max: 16.5; Threshold set to 11.6
1 possibly malignant region
Max: 19.4; Threshold set to 13.6
1 possibly malignant region
TSU-34
Local enrichment score of "proliferative"
Possibly malignant-proliferative region
Local enrichment score of "invasive"
Possibly malignant-invasive region
Max: 9.1; Threshold set to 10
No possibly malignant regions
Max: 11.8; Threshold set to 10
1 possibly malignant region
TSU-36
Local enrichment score of "proliferative"
Possibly malignant-proliferative region
Local enrichment score of "invasive"
Possibly malignant-invasive region
Max: 16.8; Threshold set to 11.8
1 possibly malignant region
Max: 6.1; Threshold set to 10
No possibly malignant regions

*Supplementary Figure S13*

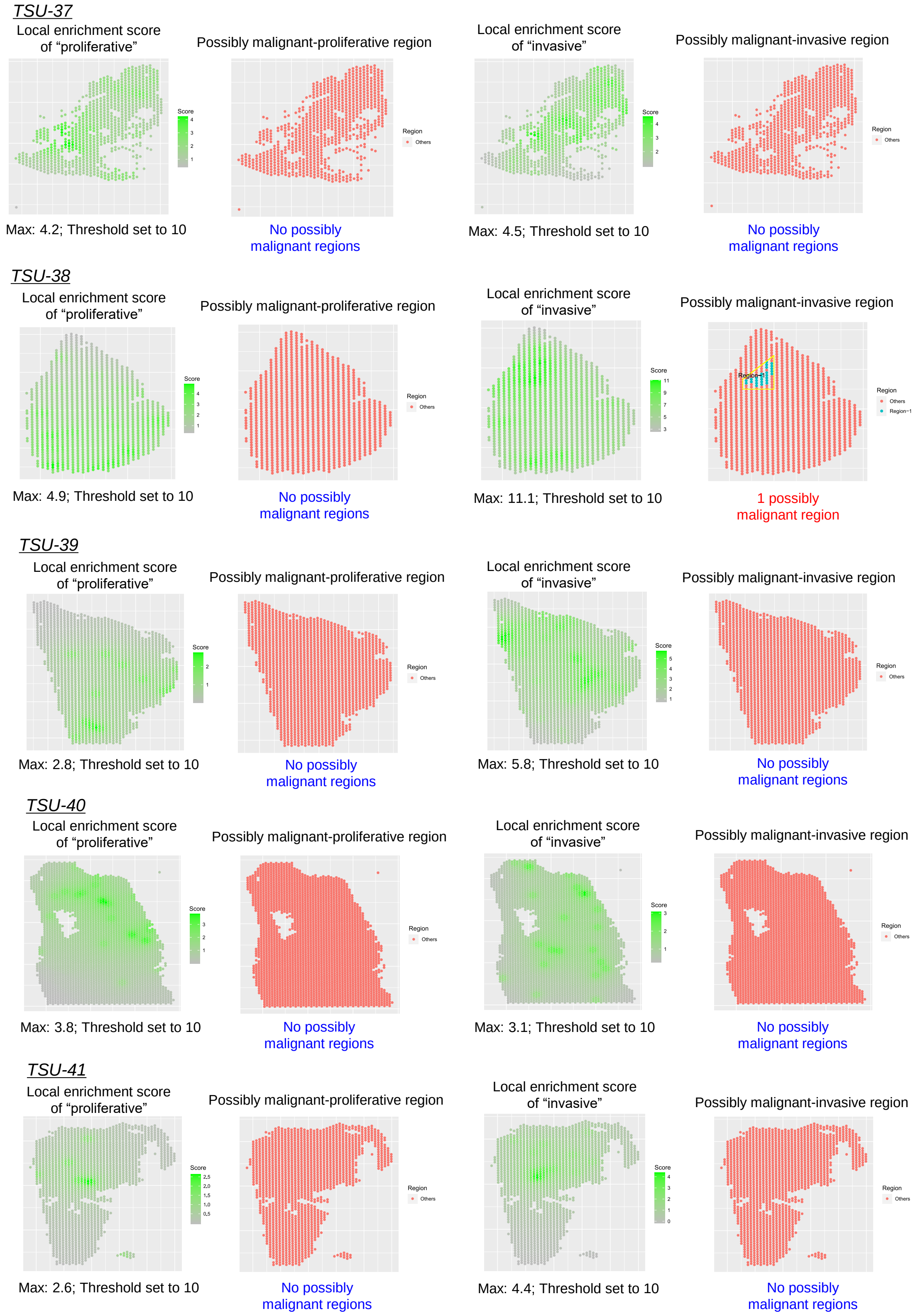

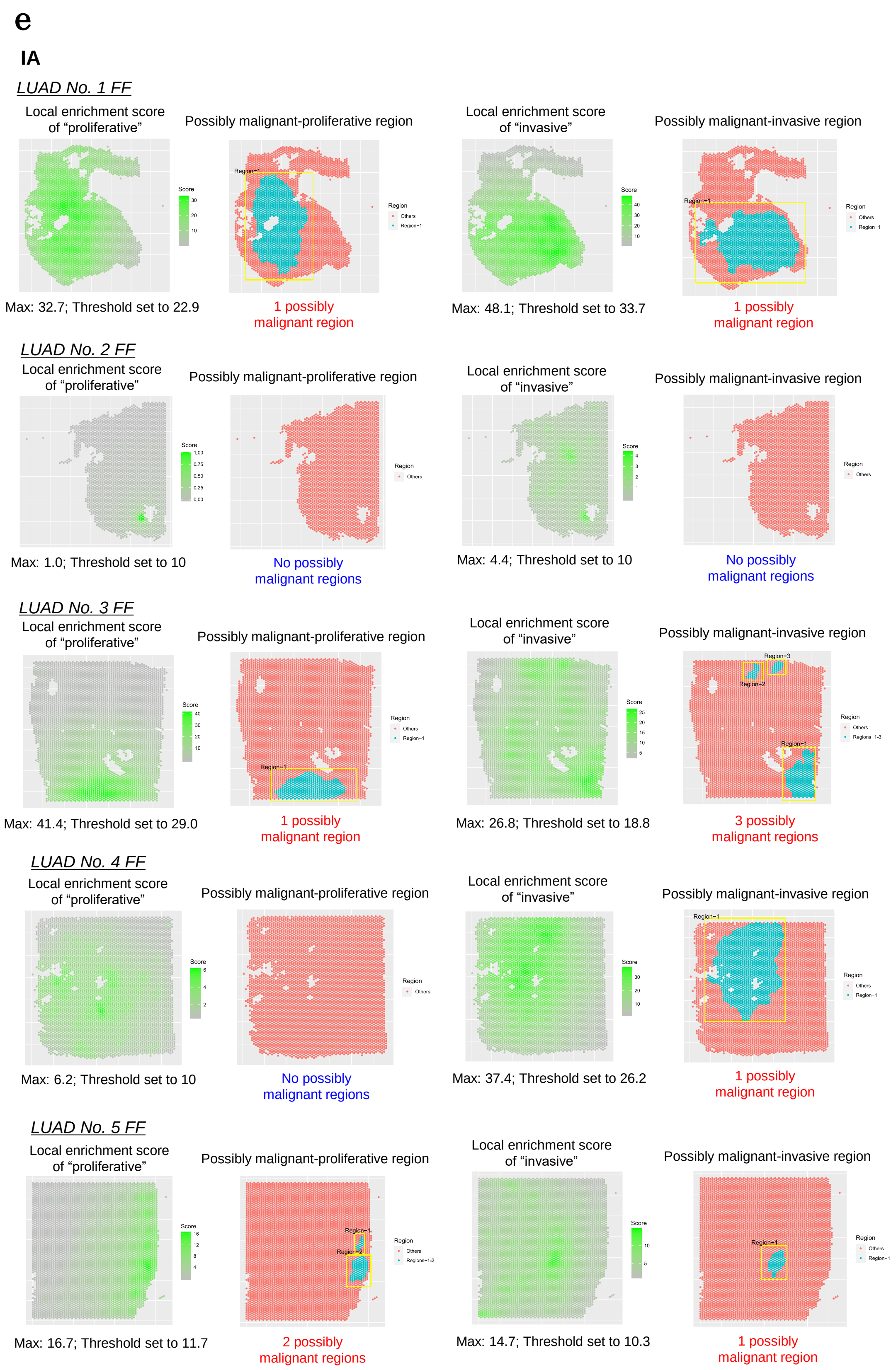
e
IA
LUAD No. 1 FF
Local enrichment score of "proliferative"
Possibly malignant-proliferative region
Local enrichment score of "invasive"
Possibly malignant-invasive region
Max: 32.7; Threshold set to 22.9
1 possibly malignant region
Max: 48.1; Threshold set to 33.7
1 possibly malignant region
LUAD No. 2 FF
Local enrichment score of "proliferative"
Possibly malignant-proliferative region
Local enrichment score of "invasive"
Possibly malignant-invasive region
Max: 1.0; Threshold set to 10
No possibly malignant regions
Max: 4.4; Threshold set to 10
No possibly malignant regions
LUAD No. 3 FF
Local enrichment score of "proliferative"
Possibly malignant-proliferative region
Local enrichment score of "invasive"
Possibly malignant-invasive region
Max: 41.4; Threshold set to 29.0
1 possibly malignant region
Max: 26.8; Threshold set to 18.8
3 possibly malignant regions
LUAD No. 4 FF
Local enrichment score of "proliferative"
Possibly malignant-proliferative region
Local enrichment score of "invasive"
Possibly malignant-invasive region
Max: 6.2; Threshold set to 10
No possibly malignant regions
Max: 37.4; Threshold set to 26.2
1 possibly malignant region
LUAD No. 5 FF
Local enrichment score of "proliferative"
Possibly malignant-proliferative region
Local enrichment score of "invasive"
Possibly malignant-invasive region
Max: 16.7; Threshold set to 11.7
2 possibly malignant regions
Max: 14.7; Threshold set to 10.3
1 possibly malignant region

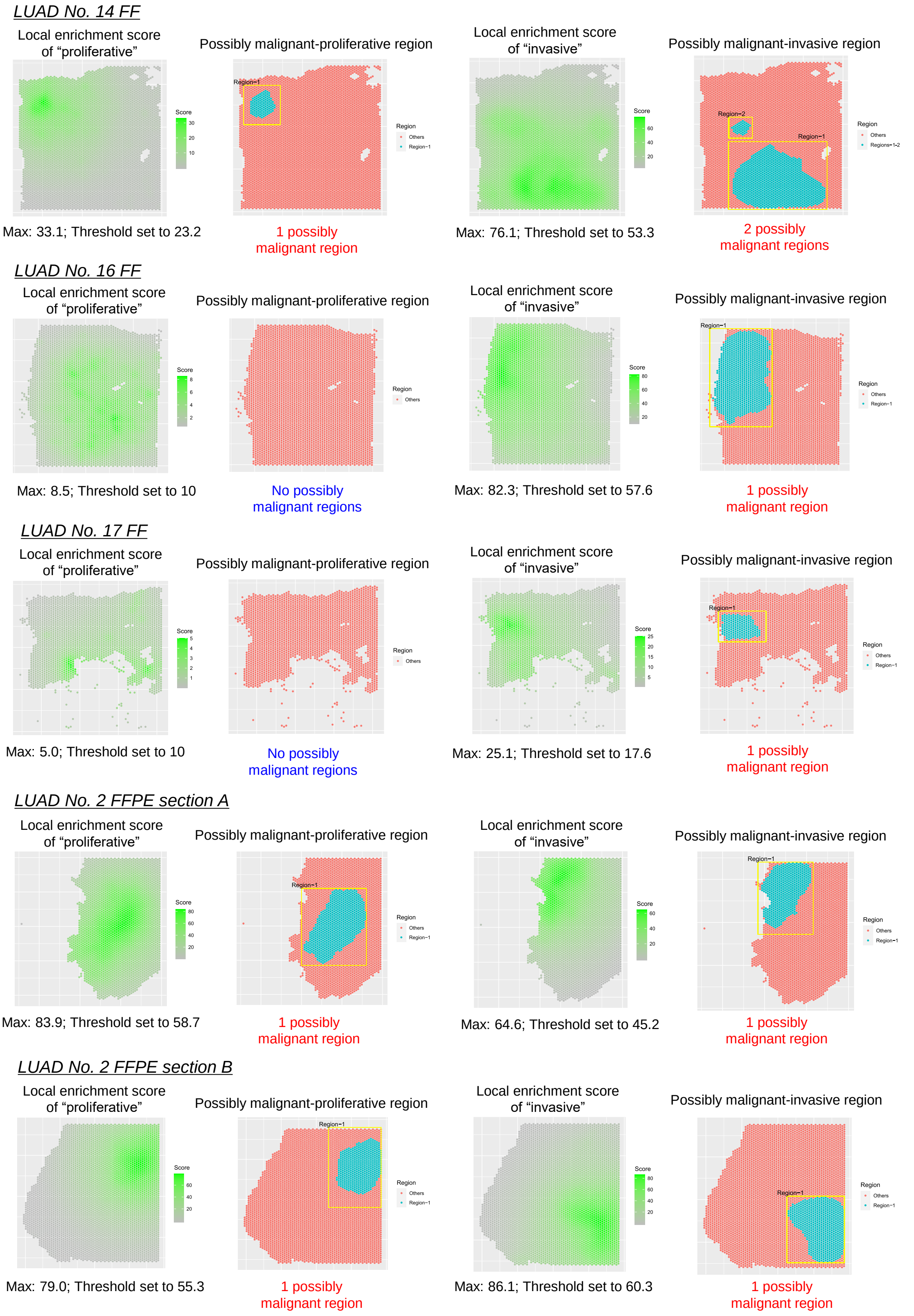

LUAD No. 14 FF
Local enrichment score of "proliferative"
Possibly malignant-proliferative region
Local enrichment score of "invasive"
Possibly malignant-invasive region
Max: 33.1; Threshold set to 23.2
1 possibly malignant region
Max: 76.1; Threshold set to 53.3
2 possibly malignant regions
LUAD No. 16 FF
Local enrichment score of "proliferative"
Possibly malignant-proliferative region
Local enrichment score of "invasive"
Possibly malignant-invasive region
Max: 8.5; Threshold set to 10
No possibly malignant regions
Max: 82.3; Threshold set to 57.6
1 possibly malignant region
LUAD No. 17 FF
Local enrichment score of "proliferative"
Possibly malignant-proliferative region
Local enrichment score of "invasive"
Possibly malignant-invasive region
Max: 5.0; Threshold set to 10
No possibly malignant regions
Max: 25.1; Threshold set to 17.6
1 possibly malignant region
LUAD No. 2 FFPE section A
Local enrichment score of "proliferative"
Possibly malignant-proliferative region
Local enrichment score of "invasive"
Possibly malignant-invasive region
Max: 83.9; Threshold set to 58.7
1 possibly malignant region
Max: 64.6; Threshold set to 45.2
1 possibly malignant region
LUAD No. 2 FFPE section B
Local enrichment score of "proliferative"
Possibly malignant-proliferative region
Local enrichment score of "invasive"
Possibly malignant-invasive region
Max: 79.0; Threshold set to 55.3
1 possibly malignant region
Max: 86.1; Threshold set to 60.3
1 possibly malignant region

*Supplementary Figure S13*

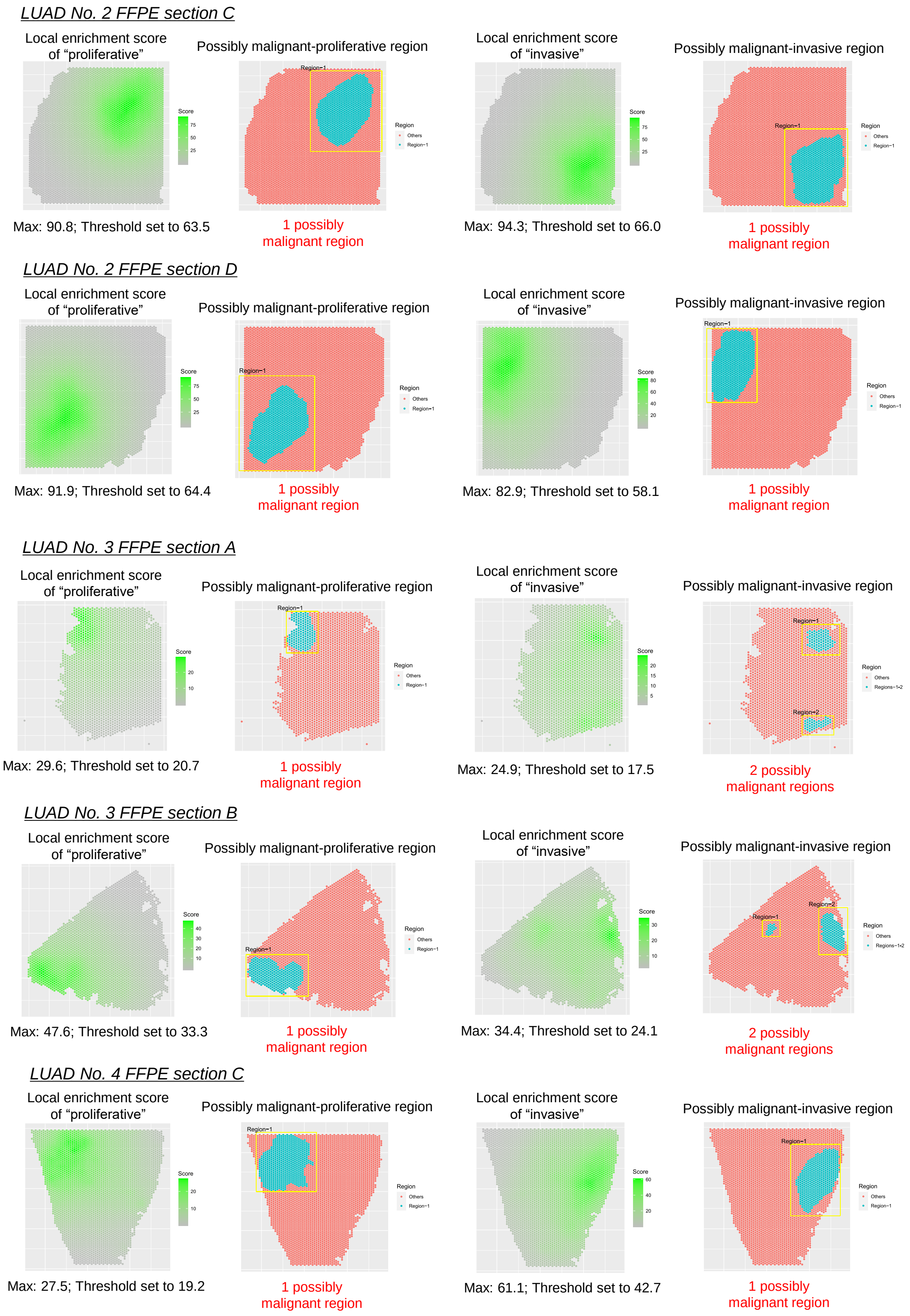

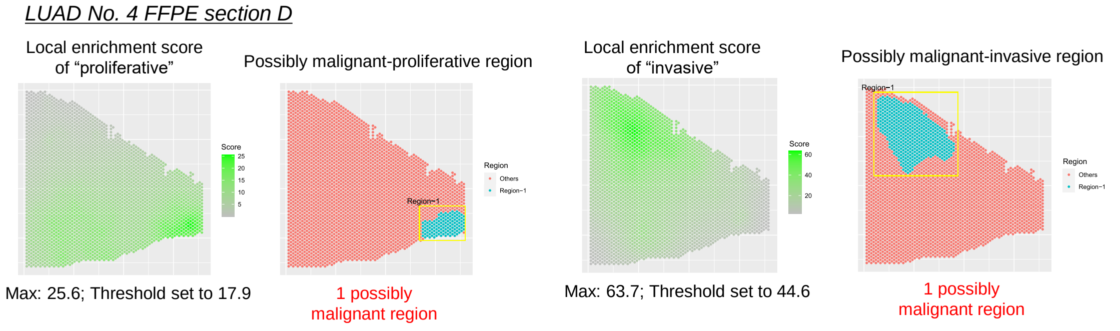


**Supplementary Figure S13 Evaluation of changes of TME scores for AIS/MIA cases**

(**a**) A workflow for evaluating changes to TME scores. Neighbor scores for "proliferative" and "invasive" spots were calculated, and "possibly malignant" regions ("possibly malignant-proliferative" and "possibly malignant-invasive" regions) were identified. (**b–e**) The spatial distribution of neighbor scores and "possibly malignant" regions is shown for early cases, which include AIS (Noguchi type A), AIS (Noguchi type B), and MIA (Noguchi type C), as well as IA cases. The results for proliferative and invasive spots are shown on the left and right, respectively. The maximum neighbor score, threshold of neighbor scores, and number of "possibly malignant regions" are shown at the bottom of the graphs.

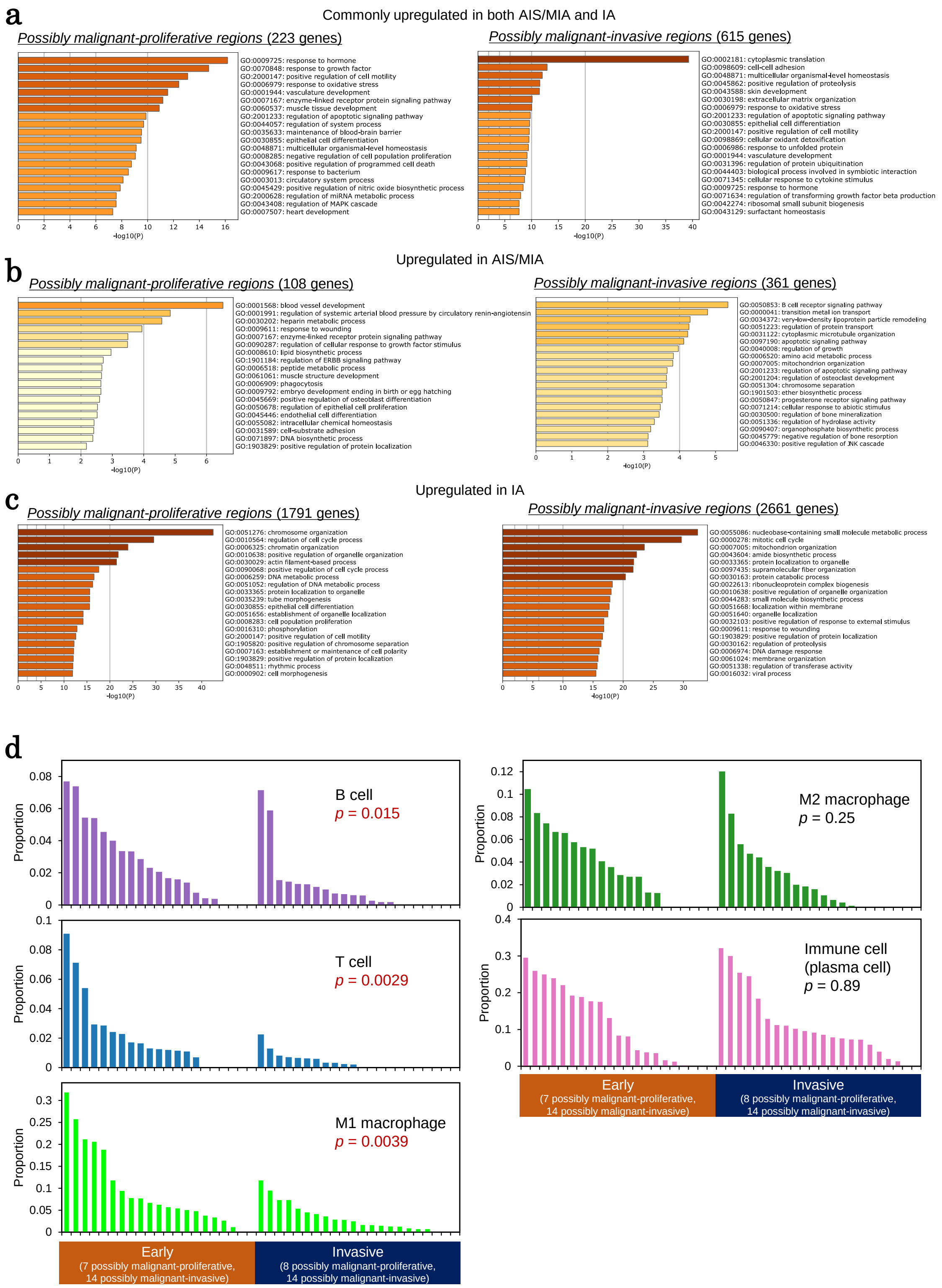
a
Commonly upregulated in both AIS/MIA and IA
Possibly malignant-proliferative regions (223 genes)
GO:0009725: response to hormone
GO:0070848: response to growth factor
GO:2000147: positive regulation of cell motility
GO:0006979: response to oxidative stress
GO:0001944: vasculature development
GO:0007167: enzyme-linked receptor protein signaling pathway
GO:0060537: muscle tissue development
GO:2001233: regulation of apoptotic signaling pathway
GO:0044057: regulation of system process
GO:0035633: maintenance of blood-brain barrier
GO:0030855: epithelial cell differentiation
GO:0048871: multicellular organismal-level homeostasis
GO:0008285: negative regulation of cell population proliferation
GO:0043068: positive regulation of programmed cell death
GO:0009617: response to bacterium
GO:0003013: circulatory system process
GO:0045429: positive regulation of nitric oxide biosynthetic process
GO:2000628: regulation of miRNA metabolic process
GO:0043408: regulation of MAPK cascade
GO:0007507: heart development
-log10(P)
Possibly malignant-invasive regions (615 genes)
GO:0002181: cytoplasmic translation
GO:0098609: cell-cell adhesion
GO:0048871: multicellular organismal-level homeostasis
GO:0045862: positive regulation of proteolysis
GO:0043588: skin development
GO:0030198: extracellular matrix organization
GO:0006979: response to oxidative stress
GO:2001233: regulation of apoptotic signaling pathway
GO:0030855: epithelial cell differentiation
GO:2000147: positive regulation of cell motility
GO:0098869: cellular oxidant detoxification
GO:0006986: response to unfolded protein
GO:0001944: vasculature development
GO:0031396: regulation of protein ubiquitination
GO:0044403: biological process involved in symbiotic interaction
GO:0071345: cellular response to cytokine stimulus
GO:0009725: response to hormone
GO:0071634: regulation of transforming growth factor beta production
GO:0042274: ribosomal small subunit biogenesis
GO:0043129: surfactant homeostasis
-log10(P)
b
Upregulated in AIS/MIA
Possibly malignant-proliferative regions (108 genes)
GO:0001568: blood vessel development
GO:0001991: regulation of systemic arterial blood pressure by circulatory renin-angiotensin
GO:0030202: heparin metabolic process
GO:0009611: response to wounding
GO:0007167: enzyme-linked receptor protein signaling pathway
GO:0090287: regulation of cellular response to growth factor stimulus
GO:0008610: lipid biosynthetic process
GO:1901184: regulation of ERBB signaling pathway
GO:0006518: peptide metabolic process
GO:0061061: muscle structure development
GO:0006909: phagocytosis
GO:0009792: embryo development ending in birth or egg hatching
GO:0045669: positive regulation of osteoblast differentiation
GO:0050678: regulation of epithelial cell proliferation
GO:0045446: endothelial cell differentiation
GO:0055082: intracellular chemical homeostasis
GO:0031589: cell-substrate adhesion
GO:0071897: DNA biosynthetic process
GO:1903829: positive regulation of protein localization
-log10(P)
Possibly malignant-invasive regions (361 genes)
GO:0050853: B cell receptor signaling pathway
GO:0000041: transition metal ion transport
GO:0034372: very-low-density lipoprotein particle remodeling
GO:0051223: regulation of protein transport
GO:0031122: cytoplasmic microtubule organization
GO:0097190: apoptotic signaling pathway
GO:0040008: regulation of growth
GO:0006520: amino acid metabolic process
GO:0007005: mitochondrion organization
GO:2001233: regulation of apoptotic signaling pathway
GO:2001204: regulation of osteoclast development
GO:0051304: chromosome separation
GO:1901503: ether biosynthetic process
GO:0050847: progesterone receptor signaling pathway
GO:0071214: cellular response to abiotic stimulus
GO:0030500: regulation of bone mineralization
GO:0051336: regulation of hydrolase activity
GO:0090407: organophosphate biosynthetic process
GO:0045779: negative regulation of bone resorption
GO:0046330: positive regulation of JNK cascade
-log10(P)
c
Upregulated in IA
Possibly malignant-proliferative regions (1791 genes)
GO:0051276: chromosome organization
GO:0010564: regulation of cell cycle process
GO:0006325: chromatin organization
GO:0010638: positive regulation of organelle organization
GO:0030029: actin filament-based process
GO:0090068: positive regulation of cell cycle process
GO:0006259: DNA metabolic process
GO:0051052: regulation of DNA metabolic process
GO:0033365: protein localization to organelle
GO:0035239: tube morphogenesis
GO:0030855: epithelial cell differentiation
GO:0051656: establishment of organelle localization
GO:0008283: cell population proliferation
GO:0016310: phosphorylation
GO:2000147: positive regulation of cell motility
GO:1905820: positive regulation of chromosome separation
GO:0007163: establishment or maintenance of cell polarity
GO:1903829: positive regulation of protein localization
GO:0048511: rhythmic process
GO:0000902: cell morphogenesis
-log10(P)
Possibly malignant-invasive regions (2661 genes)
GO:0055086: nucleobase-containing small molecule metabolic process
GO:0000278: mitotic cell cycle
GO:0007005: mitochondrion organization
GO:0043604: amide biosynthetic process
GO:0033365: protein localization to organelle
GO:0097435: supramolecular fiber organization
GO:0030163: protein catabolic process
GO:0022613: ribonucleoprotein complex biogenesis
GO:0010638: positive regulation of organelle organization
GO:0044283: small molecule biosynthetic process
GO:0051668: localization within membrane
GO:0051640: organelle localization
GO:0032103: positive regulation of response to external stimulus
GO:0009611: response to wounding
GO:1903829: positive regulation of protein localization
GO:0030162: regulation of proteolysis
GO:0006974: DNA damage response
GO:0061024: membrane organization
GO:0051338: regulation of transferase activity
GO:0016032: viral process
-log10(P)
d
Proportion
B cell
p = 0.015
T cell
p = 0.0029
M1 macrophage
p = 0.0039
M2 macrophage
p = 0.25
Immune cell
(plasma cell)
p = 0.89
Early
(7 possibly malignant-proliferative,
14 possibly malignant-invasive)
Invasive
(8 possibly malignant-proliferative,
14 possibly malignant-invasive)

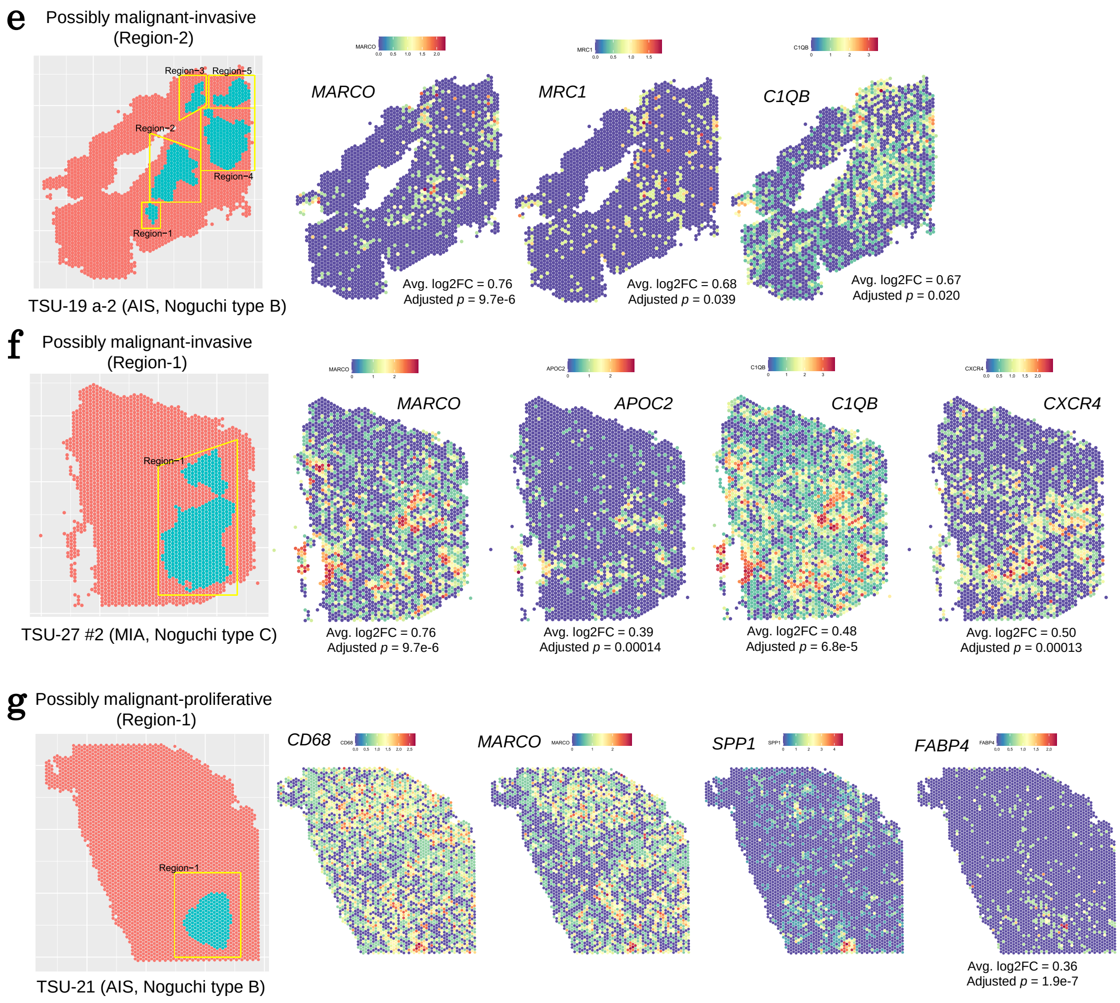


**Supplementary Figure S14 Characterization in "possibly malignant" regions**

(**a–c**) Results of Gene Ontology (GO) enrichment analysis with Metascape[5] which calculates *p*-values using the hypergeometric test. Genes with significantly higher expression in "possibly malignant-proliferative" or "possibly malignant-invasive" regions were selected for analysis. GO terms associated with highly expressed genes in both AIS/MIA and IA cases are shown in **a**. GO terms associated with only AIS/MIA or IA cases are shown in **b** and **c**, respectively. (**d**) The proportions of spots annotated with immune cells (TME scoring) in "possibly malignant-proliferative" and "possibly malignant-invasive" regions. When "possibly malignant" regions were detected in multiple sequential sections of the same tissue block, we used data from the following sections as a representative: TSU-27 #2, TSU-28 #1, LUAD No. 2 FFPE C, LUAD No. 3

FFPE B, and LUAD No. 4 FFPE C. *P*-value is shown in the inset (Wilcoxon rank sum test). (**e–g**) Representative “possibly malignant” regions. Genes with differential expression from other regions are shown. *P*-values and average log2FC were calculated using the Seurat FindMarkers function based on Wilcoxon rank sum test. (**e**) The “possibly malignant-invasive” region in TSU-19 (left; Region-2 in green); macrophage markers are significantly highly expressed (right). (**f**) The “possibly malignant-invasive” region in TSU-27 (left; Region-1 in blue). Macrophage and immune cell markers represent significantly higher expression (right). (**g**) The “possibly malignant-proliferative” region in TSU-21 (left; Region-1 in blue). Macrophage markers are shown (right). *FABP4* expression is significantly elevated in the “possibly malignant” region.

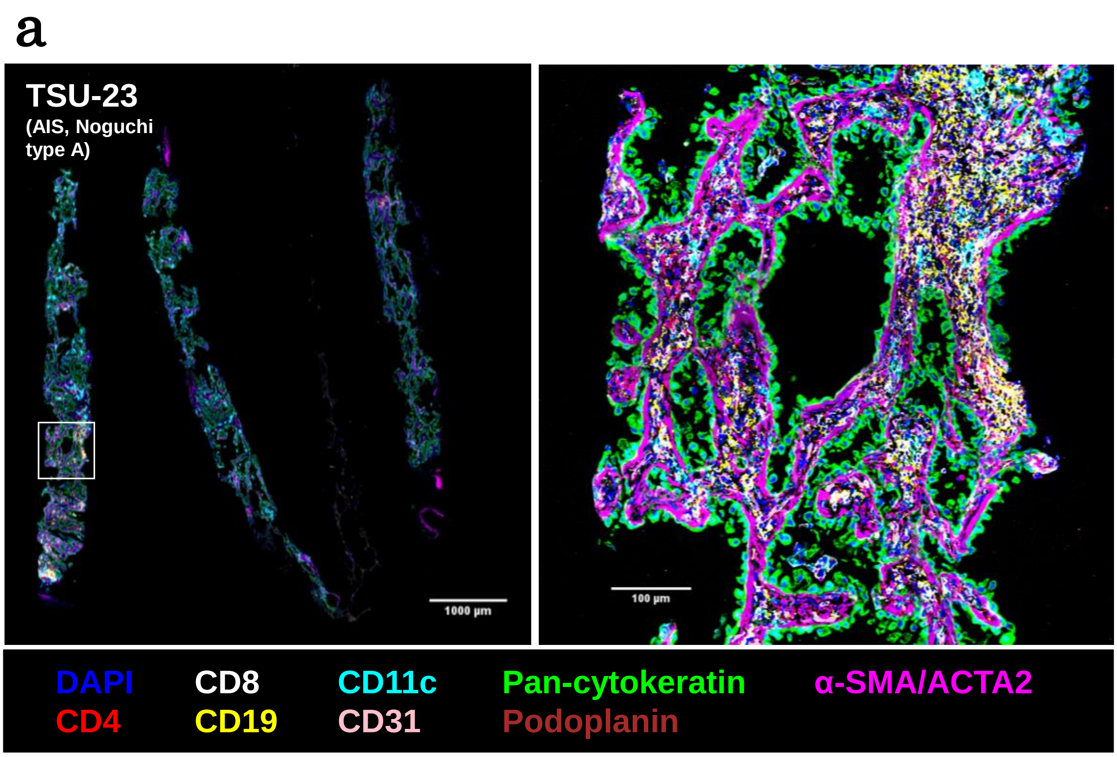
a
TSU-23
(AIS, Noguchi type A)
1000 μm
100 μm
DAPI
CD8
CD11c
Pan-cytokeratin
α-SMA/ACTA2
CD4
CD19
CD31
Podoplanin

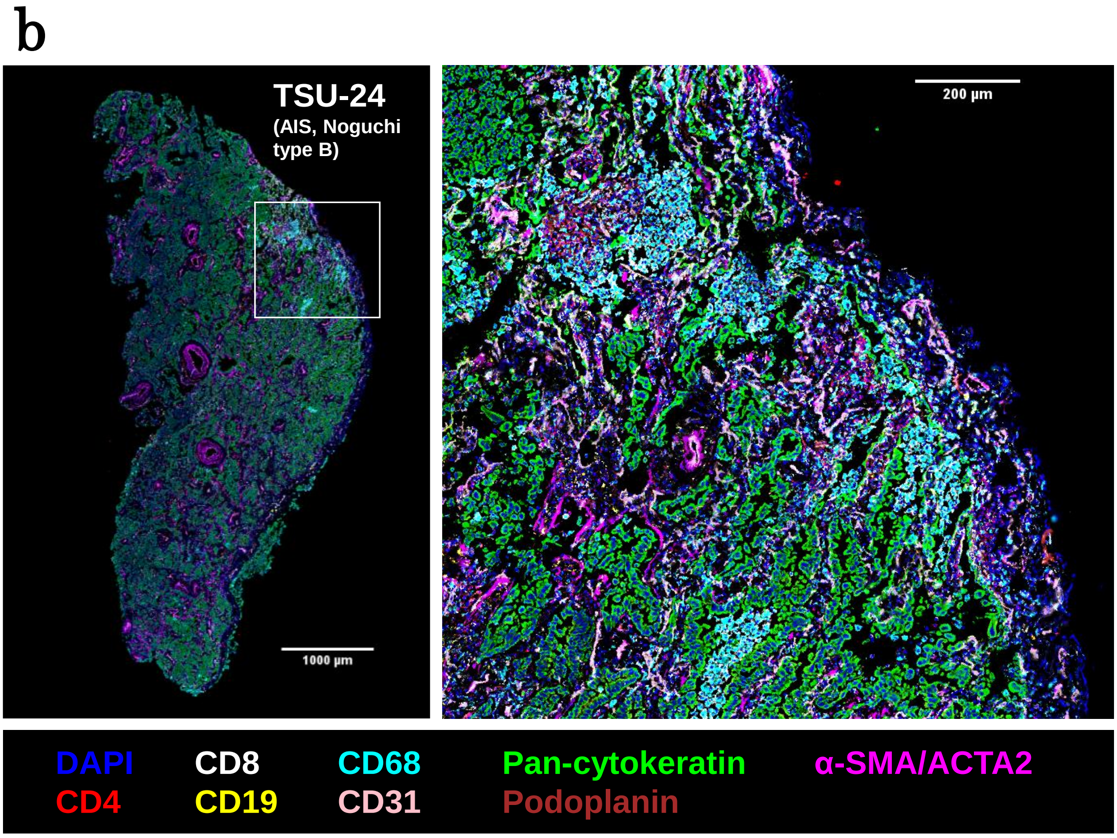
b
TSU-24
(AIS, Noguchi type B)
1000 μm
200 μm
DAPI
CD8
CD68
Pan-cytokeratin
α-SMA/ACTA2
CD4
CD19
CD31
Podoplanin

**c**

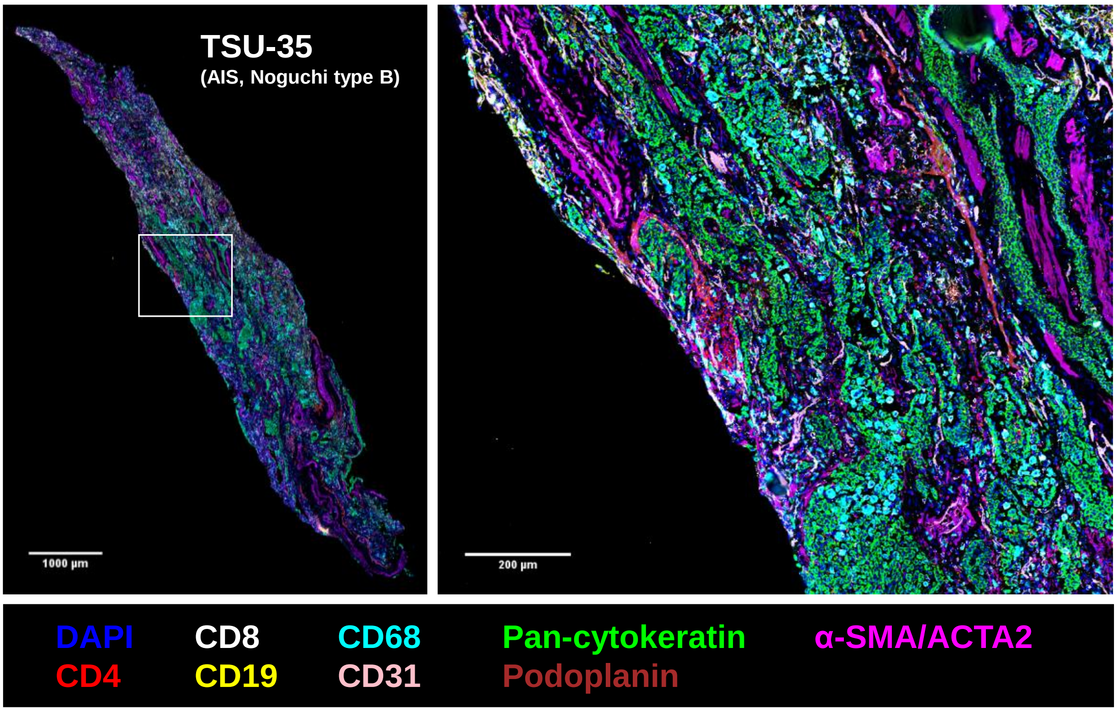
TSU-35
(AIS, Noguchi type B)
1000 µm
200 µm
DAPI
CD4
CD8
CD19
CD68
CD31
Pan-cytokeratin
Podoplanin
α-SMA/ACTA2

**d**

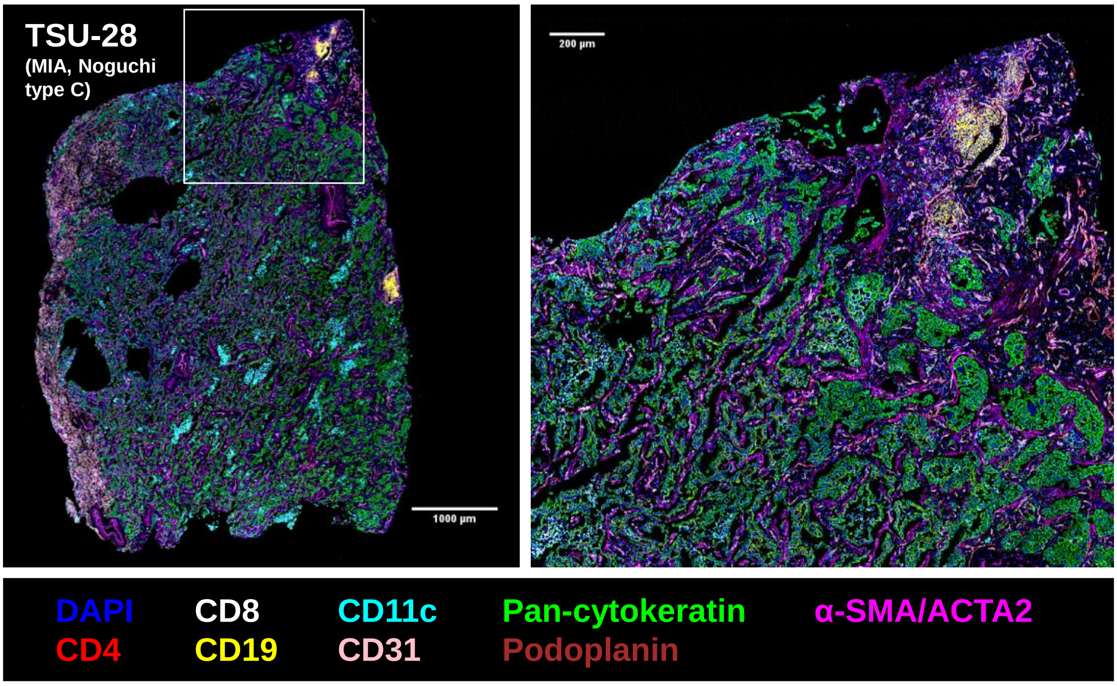
TSU-28
(MIA, Noguchi type C)
1000 µm
200 µm
DAPI
CD4
CD8
CD19
CD11c
CD31
Pan-cytokeratin
Podoplanin
α-SMA/ACTA2

e

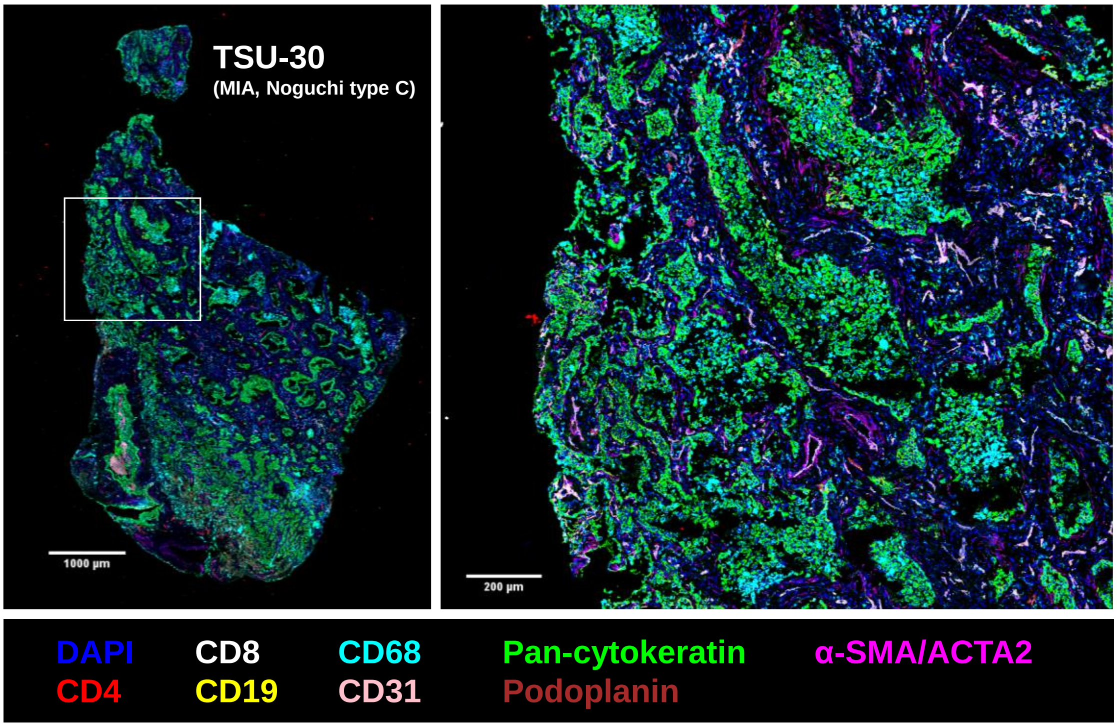
TSU-30
(MIA, Noguchi type C)
1000 μm
200 μm
DAPI
CD4
CD8
CD19
CD68
CD31
Pan-cytokeratin
Podoplanin
α-SMA/ACTA2

f

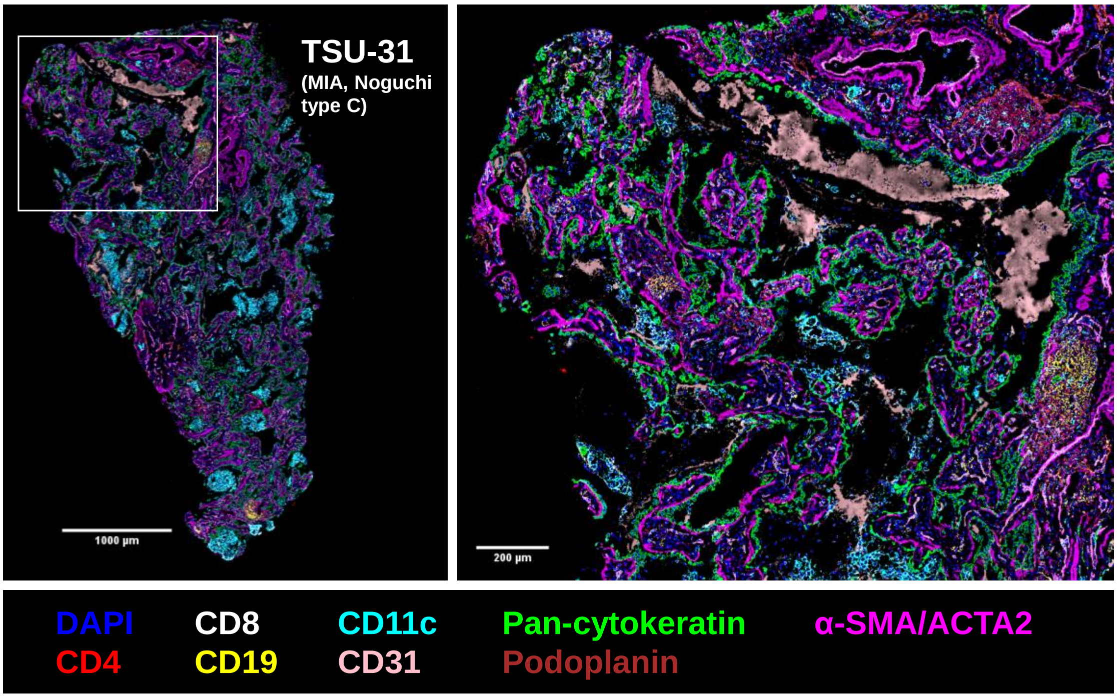
TSU-31
(MIA, Noguchi
type C)
1000 μm
200 μm
DAPI
CD4
CD8
CD19
CD11c
CD31
Pan-cytokeratin
Podoplanin
α-SMA/ACTA2

**g** TSU-20 (AIS, Noguchi type A; *Nat Commun* 2023)

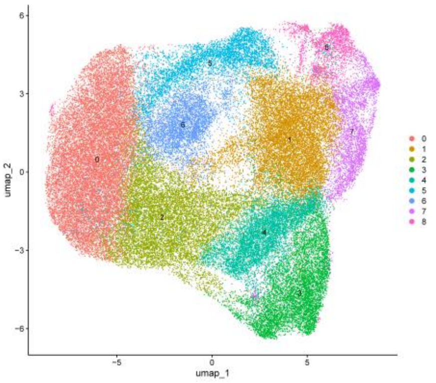


| Cluster | Annotation | Feature* |
|---|---|---|
| 0 | High podoplanin tumor cell | NKX2-1, Pan-Cytokeratin, Podoplanin |
| 1 | Immune cell | **CD45RO**, **CD4**, CD11c |
| 2 | Tumor cell/Stromal cell | α-SMA, Pan-Cytokeratin, β-Catenin1 |
| 3 | Endothelial cell | CD31, Caveolin, PD-L1 |
| 4 | Stromal cell/Immune cell | α-SMA, Caveolin, CD163 |
| 5 | Macrophage/DC | CD11c, CD68 |
| 6 | High PD-L1 tumor cell | **PD-L1**, NKX2-1, Pan-Cytokeratin |
| 7 | B cell | **CD19**, **CXCL13**, CD45RO |
| 8 | Proliferating immune cell | **Ki67**, CXCL13, CTLA4 |

*Top 3 DEGs; log2FC > 0.5; log2FC > 2 in bold

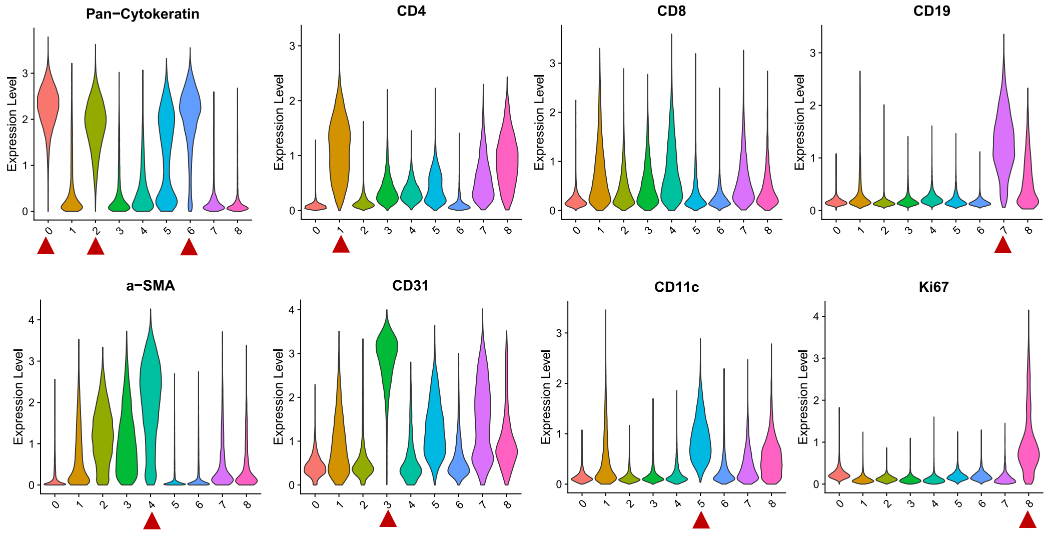


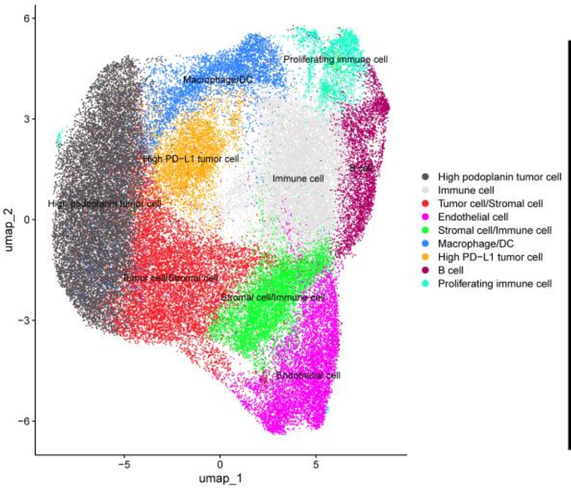


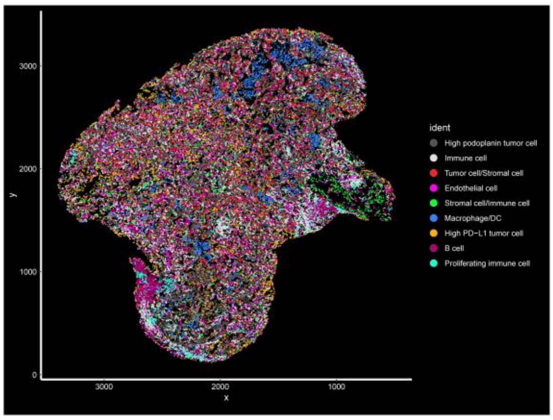

**h** TSU-23 (AIS, Noguchi type A)

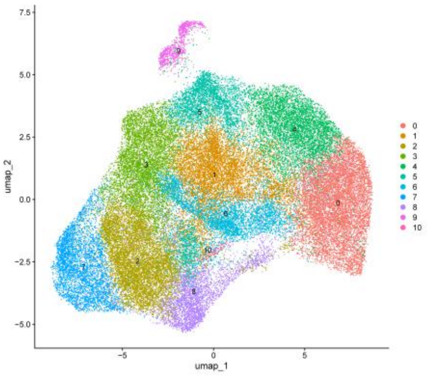


| Cluster | Annotation | Feature* |
|---|---|---|
| 0 | Tumor cell | NKX2-1, Pan-Cytokeratin, TP53 |
| 1 | Stromal cell | CD31, a-SMA |
| 2 | T cell | **CD45RO**, CD4, CD8 |
| 3 | Immune cell | CD163, TP53, CD8 |
| 4 | Tumor cell/Stromal cell | NKX2-1, Pan-Cytokeratin, α-SMA |
| 5 | Proliferating fibroblast/SMC | α-SMA, Ki67 |
| 6 | Endothelial cell | Podoplanin, CD31 |
| 7 | B cell | CD19, CD45RO, CD11c |
| 8 | Macrophage/DC | **CD11c**, CD4, CD45RO |
| 9 | Proliferating cell | **Ki67**, CD11c |
| 10 | Stromal cell/Immune cell | **Podoplanin**, CD45RO, CD11c |

*Top 3 DEGs; log2FC > 0.5; log2FC > 2 in bold

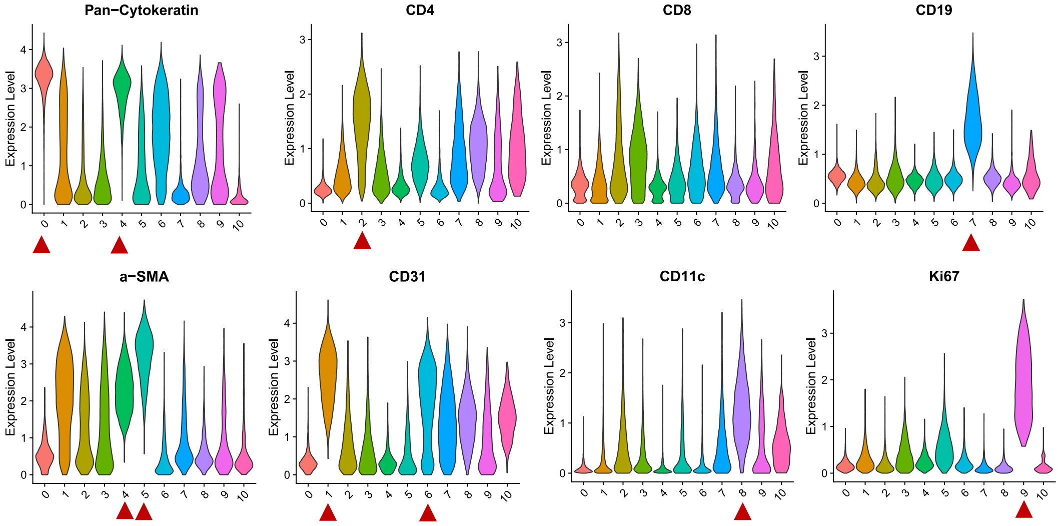


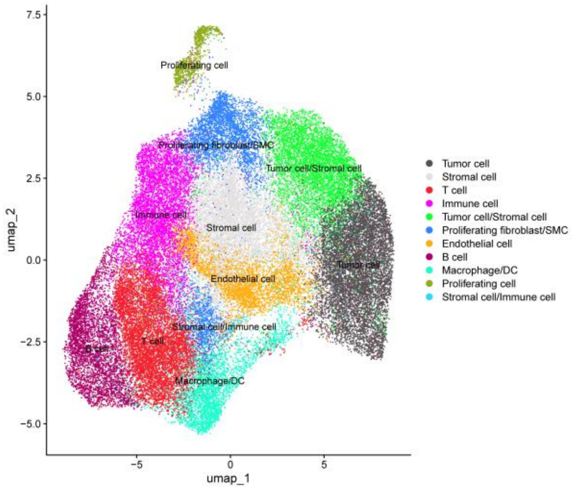


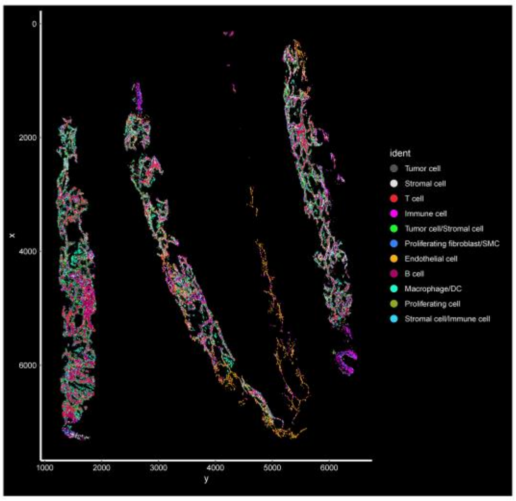

**i** TSU-21 (AIS, Noguchi type B; *Nat Commun* 2023)

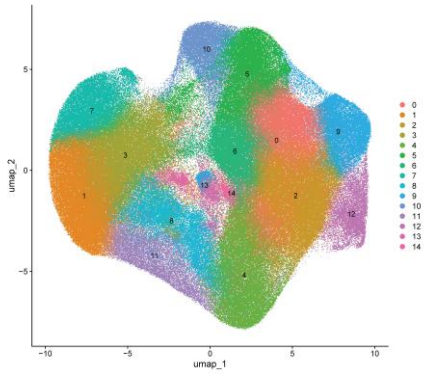


*Top 3 DEGs; log2FC > 0.5; log2FC > 2 in bold

| Cluster | Annotation | Feature* |
|---|---|---|
| 0 | Stromal cell/Immune cell | Podoplanin, CD19, Caveolin |
| 1 | Tumor cell | Pan-Cytokeratin, NKX2-1, β-Catenin1 |
| 2 | T cell | CD45RO, CD4, CD8 |
| 3 | Tumor cell | Pan-Cytokeratin, NKX2-1, β-Catenin1 |
| 4 | Macrophage/DC | CD11c, CD68, CD4 |
| 5 | Stromal cell | **α-SMA**, Caveolin, CD31 |
| 6 | Immune cell | CD163, CD8 |
| 7 | High LIF tumor cell | NKX2-1, LIF, Pan-Cytokeratin |
| 8 | High PD-L1 cell | **PD-L1**, Pan-Cytokeratin, CD11c |
| 9 | B cell | **CXCL13**, **CD19**, Podoplanin |
| 10 | High PD-L1 stromal cell | **PD-L1**, Caveolin, CD31 |
| 11 | Tumor cell/Immune cell | CD11c, Pan-Cytokeratin |
| 12 | Treg | **FOXP3**, CD45RO, CD4 |
| 13 | Proliferating cell | **Ki67** |
| 14 | Proliferating B cell | **CXCL13**, CD19, Ki67 |

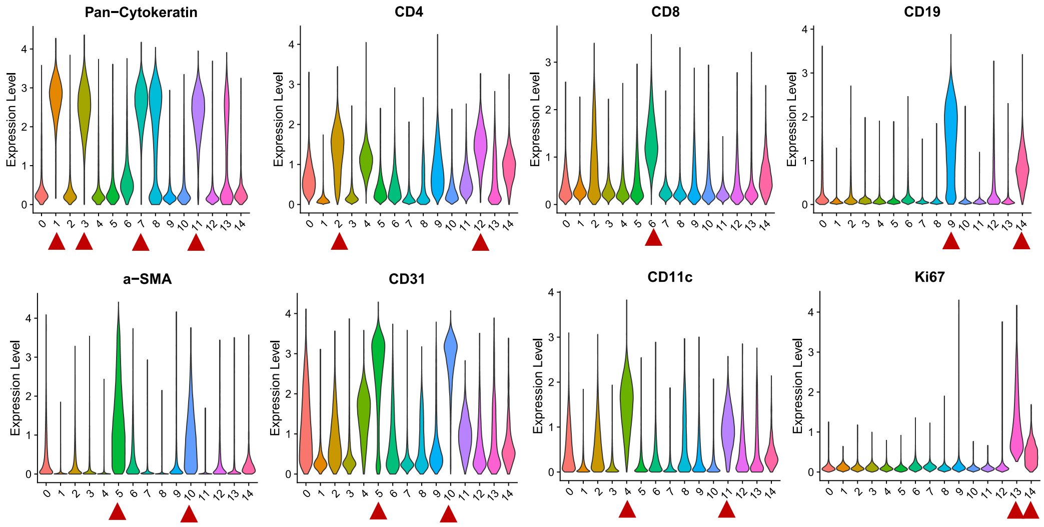


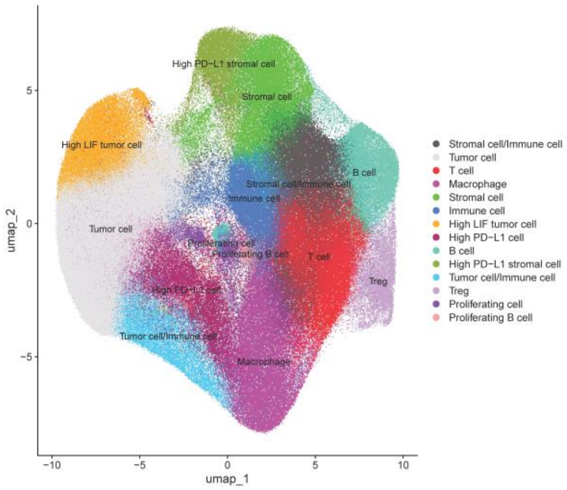


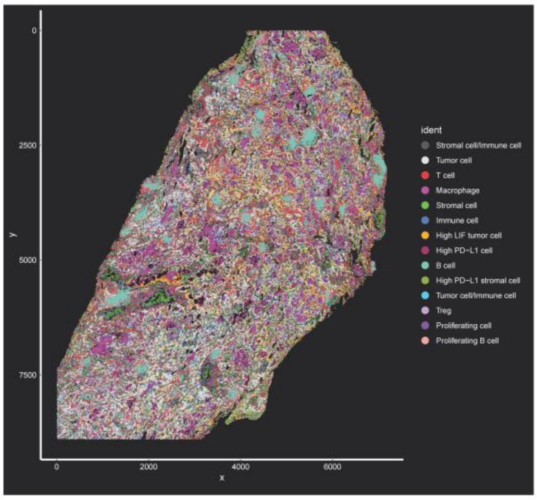

**j** TSU-24 (AIS, Noguchi type B)

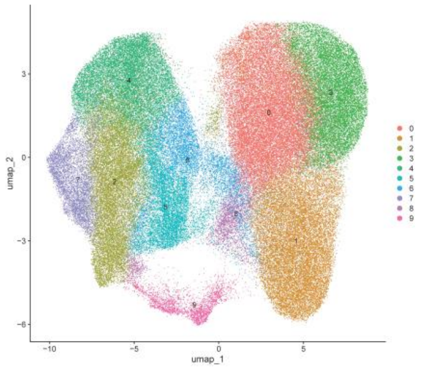


| Cluster | Annotation | Feature* |
|---|---|---|
| 0 | Tumor cell | NKX2-1, Pan-Cytokeratin |
| 1 | High podoplanin tumor cell | Podoplanin, Pan-Cytokeratin, NKX2-1 |
| 2 | Macrophage/DC | **CD11c**, CD68, CD45RO |
| 3 | Tumor cell | NKX2-1, PD-1, Pan-Cytokeratin |
| 4 | Immune cell | CD163, CD19, CD8 |
| 5 | Stromal cell | **CD31**, α-SMA |
| 6 | Fibroblast/SMC | **α-SMA** |
| 7 | CD4+ T cell | **CD45RO**, **CD4**, CTLA4 |
| 8 | Others | CD11c, Pan-Cytokeratin |
| 9 | Proliferating cell | **Ki67**, CD11c |

*Top 3 DEGs; log2FC > 0.5; log2FC > 2 in bold

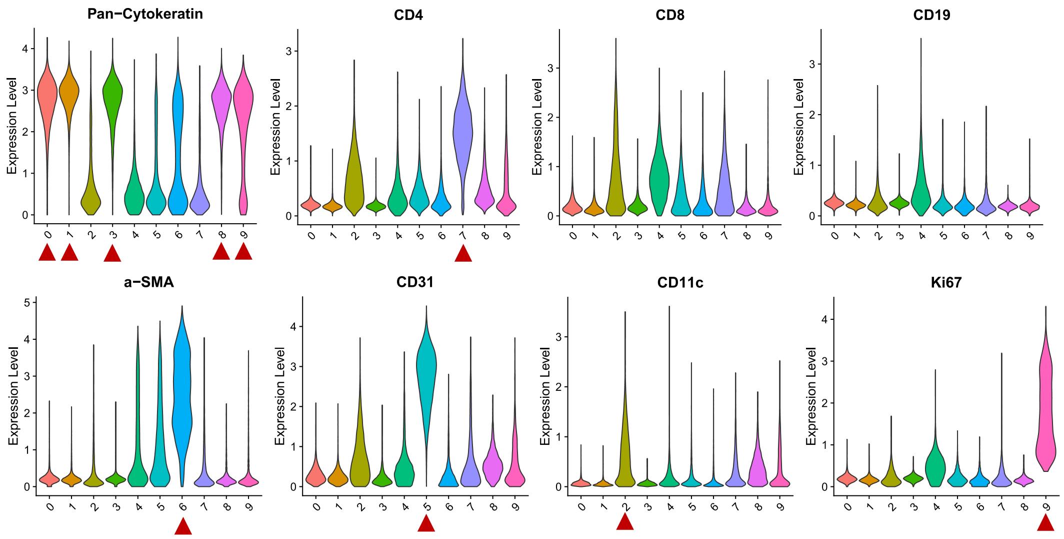


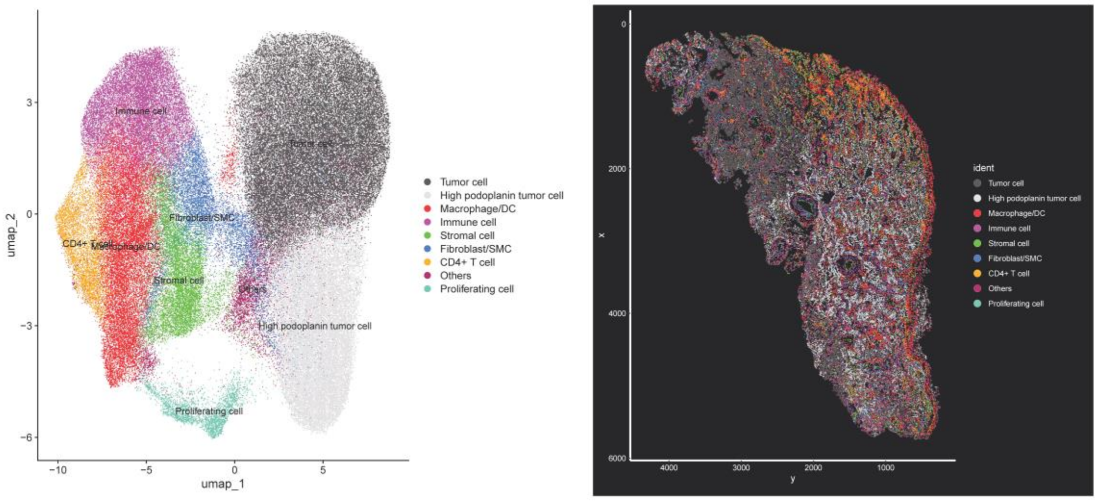

**k** TSU-35 (AIS, Noguchi type B)

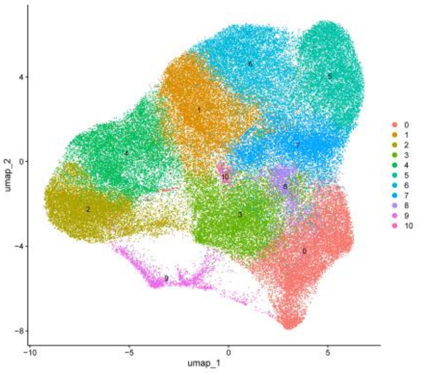


| Cluster | Annotation | Feature* |
|---|---|---|
| 0 | T cell | **CD45RO**, CD4, CD8 |
| 1 | Endothelial cell | Podoplanin, CD31 |
| 2 | Tumor cell | **NKX2-1**, Pan-Cytokeratin |
| 3 | Macrophage/DC | CD11c, CD68, CD4 |
| 4 | High podoplanin tumor cell | NKX2-1, Pan-Cytokeratin, Podoplanin |
| 5 | Fibroblast/SMC | α-SMA, FOXP3, CD56 |
| 6 | Endothelial cell | CD31, α-SMA |
| 7 | Immune cell | CD163, TP53, CD8 |
| 8 | B cell | **CD19**, CD45RO, CD4 |
| 9 | Proliferating tumor cell | **Ki67**, NKX2-1 |
| 10 | Proliferating immune cell | Ki67, PD-L1, CXCL13 |

*Top 3 DEGs; log2FC > 0.5; log2FC > 2 in bold

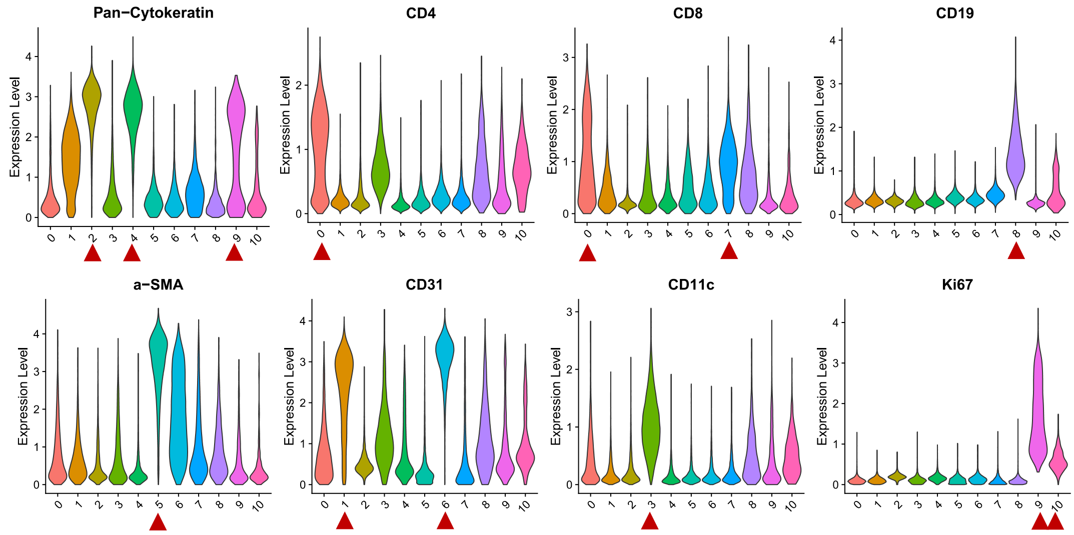


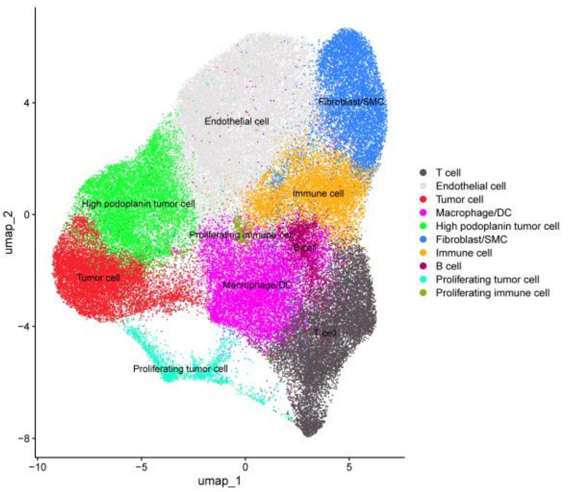


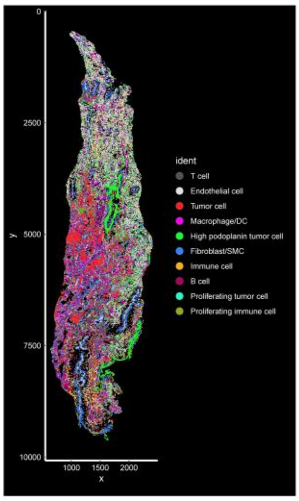

# 1 TSU-28 (MIA, Noguchi type C)

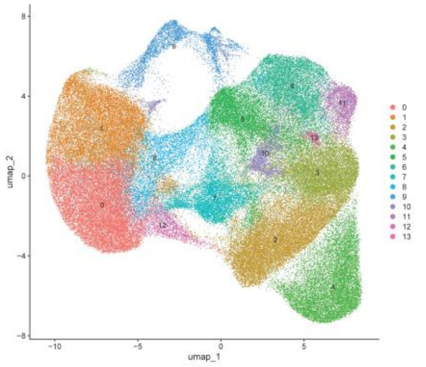


*Top 3 DEGs; log2FC > 0.5; log2FC > 2 in bold

| Cluster | Annotation | Feature* |
|---|---|---|
| 0 | High podoplanin tumor cell | NKX2-1, Pan-Cytokeratin, Podoplanin |
| 1 | Tumor cell | NKX2-1, Pan-Cytokeratin |
| 2 | Proliferating fibroblast/SMC | **α-SMA**, Ki67 |
| 3 | Immune cell | CD163, CD8, TP53 |
| 4 | Endothelial cell | **CD31**, α-SMA |
| 5 | Macrophage/DC | **CD11c**, CD45RO, CD4 |
| 6 | CD4+ T cell | **CD45RO**, **CD4** |
| 7 | Endothelial cell | CD31, Podoplanin |
| 8 | Tumor cell/Immune cell | CD11c, Pan-Cytokeratin, NKX2-1 |
| 9 | Proliferating tumor cell | **Ki67**, NKX2-1 |
| 10 | B cell | CD19, CD45RO, CD4 |
| 11 | CD8+ T cell | CD8, CD45RO, PD-1 |
| 12 | Tumor cell/Stromal cell | α-SMA, NKX2-1, Pan-Cytokeratin |
| 13 | Proliferating immune cell | **Ki67**, CD163, CD45RO |

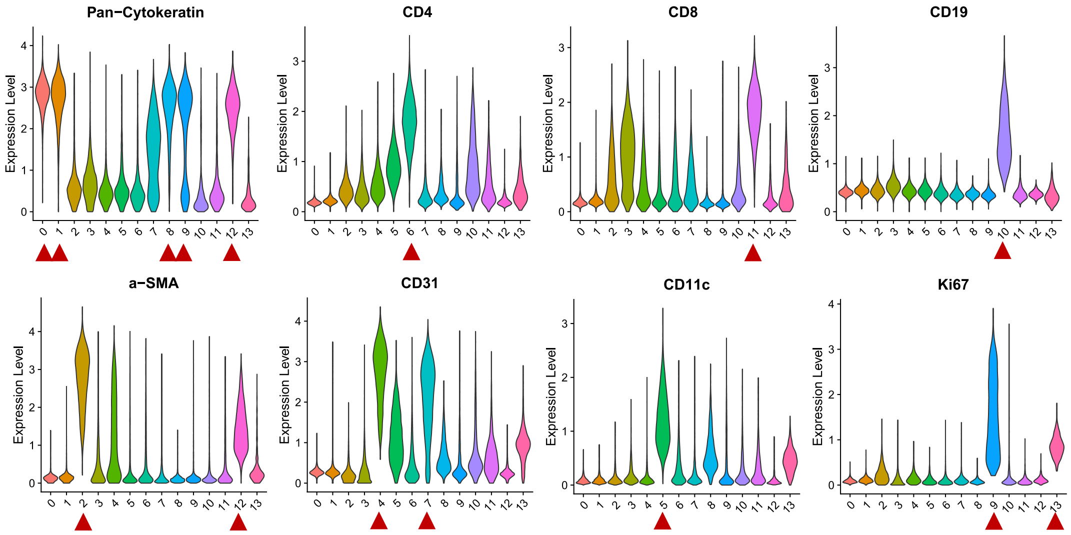


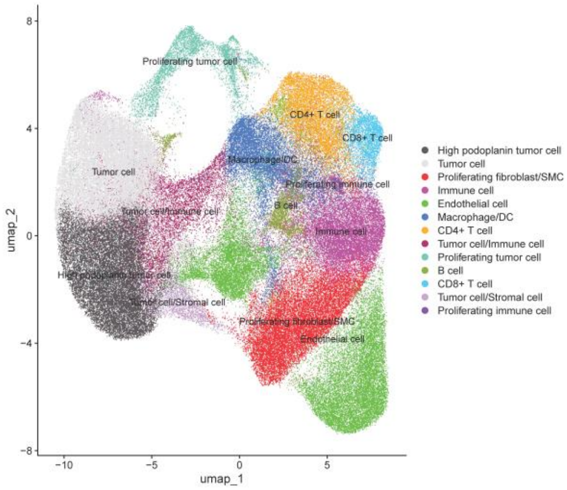


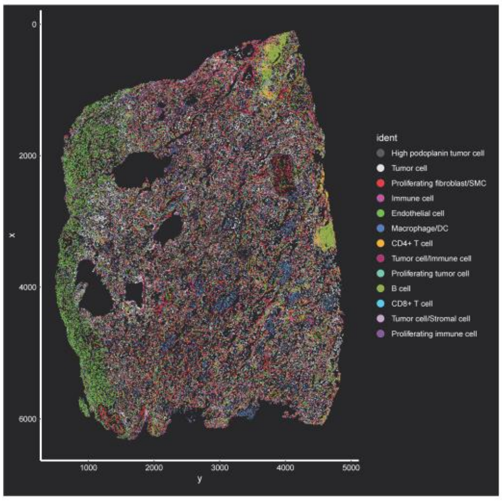

*Supplementary Figure S15*

**m** TSU-30 (MIA, Noguchi type C)

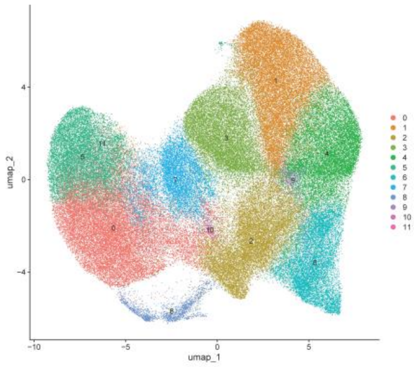


| Cluster | Annotation | Feature* |
|---|---|---|
| 0 | Tumor cell | NKX2-1, Pan-Cytokeratin |
| 1 | Fibroblast/SMC | α-SMA, CD56 |
| 2 | Macrophage/DC | **CD11c**, CD68, CD4 |
| 3 | Endothelial cell | **CD31**, α-SMA |
| 4 | Immune cell | CD163, CD8, TP53 |
| 5 | High podoplanin tumor cell | NKX2-1, Podoplanin, Pan-Cytokeratin |
| 6 | T cell | **CD45RO**, **CD4**, **CD8** |
| 7 | Endothelial cell | Podoplanin, CD31, PD-L1 |
| 8 | Proliferating cell | **Ki67**, CD11c, NKX2-1 |
| 9 | B cell | CD19, CD45RO, CD4 |
| 10 | Proliferating immune cell | **Ki67**, PD-L1, CXCL13 |
| 11 | Tumor cell/Immune cell | **CTLA4**, NKX2-1, Podoplanin |

*Top 3 DEGs; log2FC > 0.5; log2FC > 2 in bold

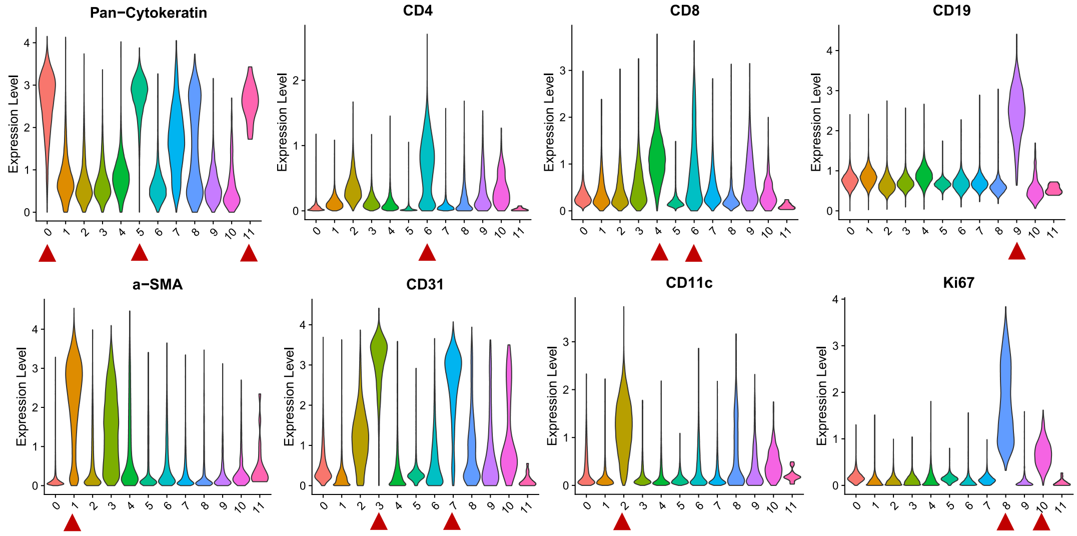


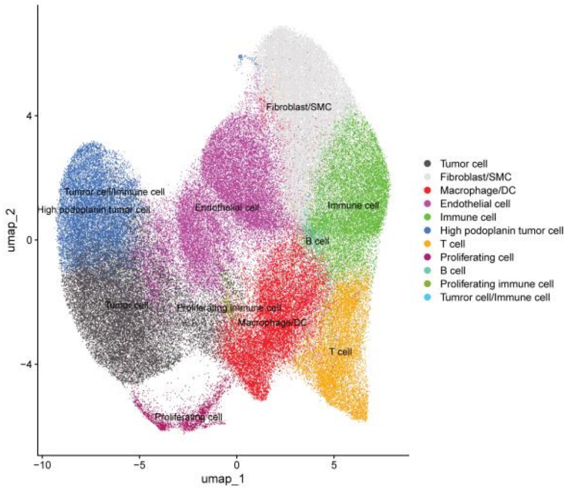


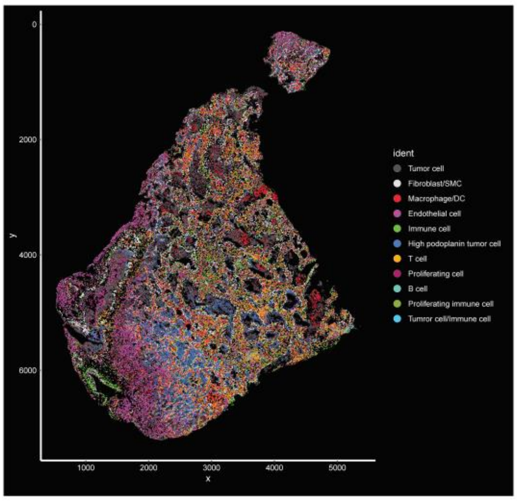

**n** TSU-31 (MIA, Noguchi type C)

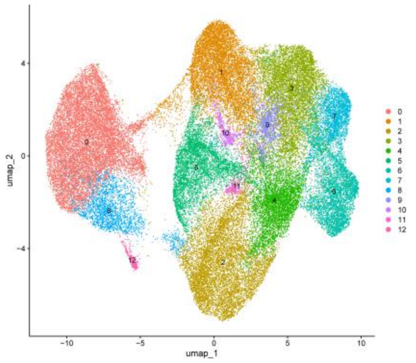


*Top 3 DEGs; log2FC > 0.5; log2FC > 2 in bold

| Cluster | Annotation | Feature* |
|---|---|---|
| 0 | Tumor cell | **NKX2-1**, **Pan-Cytokeratin** |
| 1 | Macrophage/DC | **CD11c**, CD31, CD4 |
| 2 | Proliferating fibroblast/SMC | **α-SMA**, Ki67, Podoplanin |
| 3 | CD4+ T cell | CD4, CD45RO, PD-1 |
| 4 | Immune cell | CD163, TP53, CD8 |
| 5 | Endothelial cell | CD31, α-SMA |
| 6 | B cell | **CD19**, Podoplanin |
| 7 | CD8+ T cell | **CD8**, CD45RO, PD-1 |
| 8 | Tumor cell/Stromal cell | Pan-Cytokeratin, NKX2-1, a-SMA |
| 9 | Treg | FOXP3, CD45RO, CD4 |
| 10 | Proliferating immune cell | **Ki67**, CD11c, CD4 |
| 11 | Endothelial cell | **Podoplanin**, CD31 |
| 12 | Proliferating tumor cell | **Ki67**, NKX2-1, Pan-Cytokeratin |

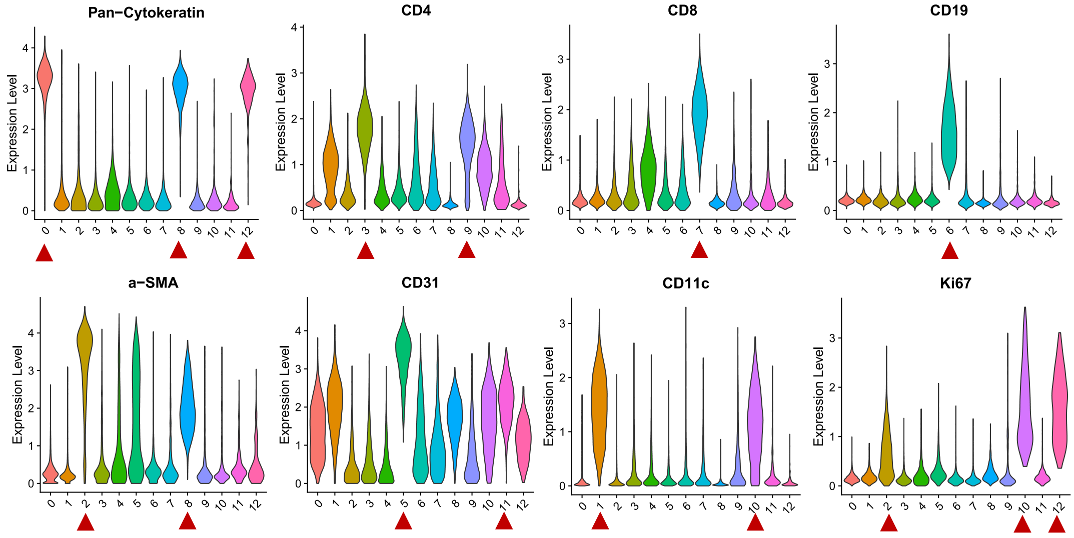


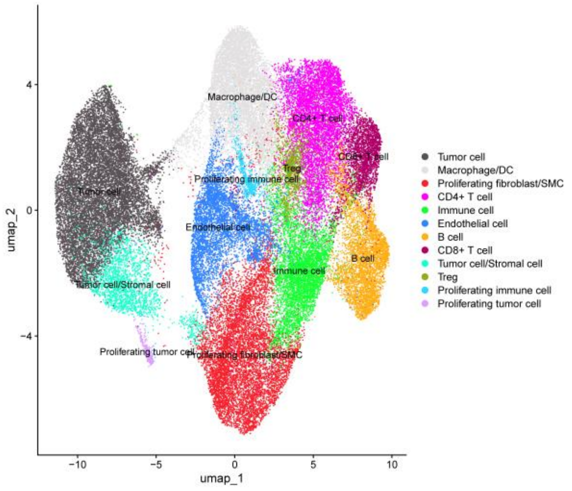


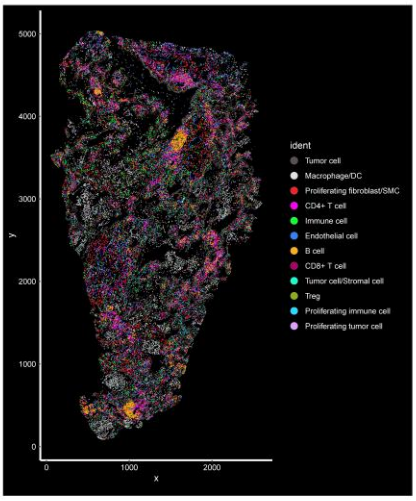

**o** TSU-33 (MIA, Noguchi type C; *Nat Commun* 2023)

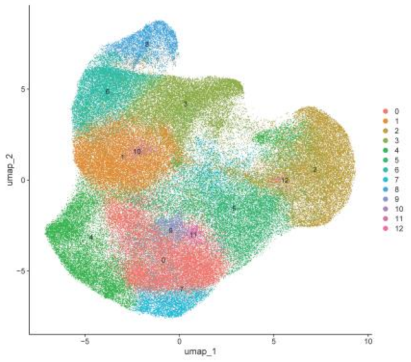


*Top 3 DEGs; log2FC > 0.5; log2FC > 2 in bold

| Cluster | Annotation | Feature* |
|---|---|---|
| 0 | Endothelial cell | Caveolin, CD31, Podoplanin |
| 1 | Proliferating immune cell | CD163, CD8, Ki67 |
| 2 | Tumor cell | NKX2-1, Pan-Cytokeratin, Galectin3 |
| 3 | Macrophage/DC | CD11c, CD68, CD4 |
| 4 | Fibroblast/SMC | **α-SMA**, Caveolin |
| 5 | High podoplanin tumor cell | NKX2-1, Podoplanin, Pan-Cytokeratin |
| 6 | T cell | CD45RO, CD4, CD8 |
| 7 | High PD-L1 endothelial cell | **PD-L1**, CD31, Caveolin |
| 8 | B cell | **CXCL13**, CD19, CD4 |
| 9 | Macrophage/DC/Endothelial cell | CD11c, CD31, CD68 |
| 10 | Proliferating immune cell | Ki67, PD-L1, CD45RO |
| 11 | Others | CD31, CD45RO, Podoplanin |
| 12 | Proliferating tumor cell | **Ki67**, NKX2-1, Pan-Cytokeratin |

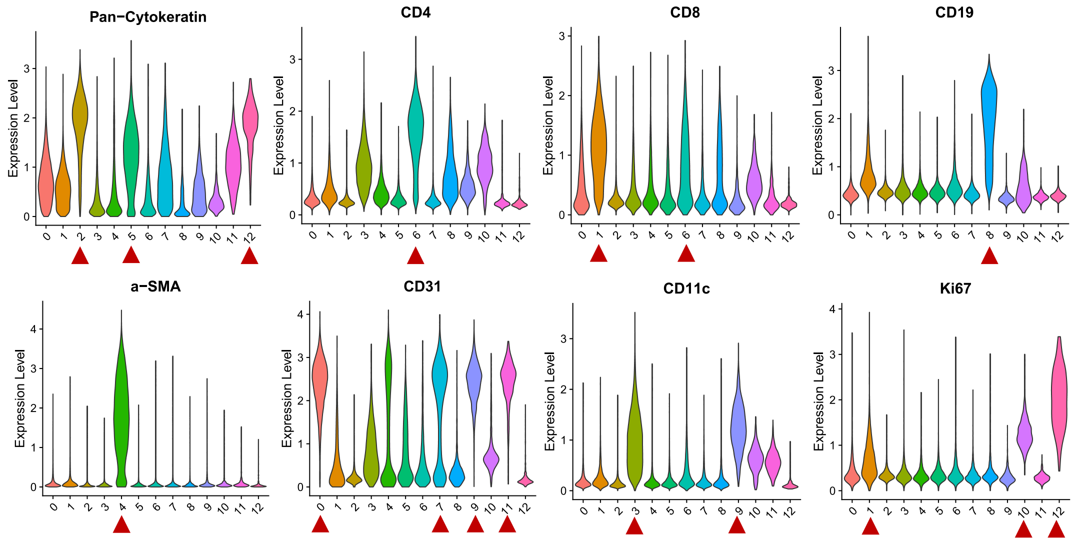


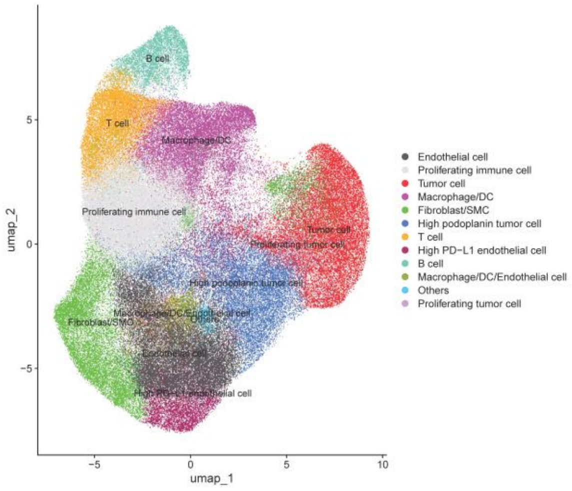


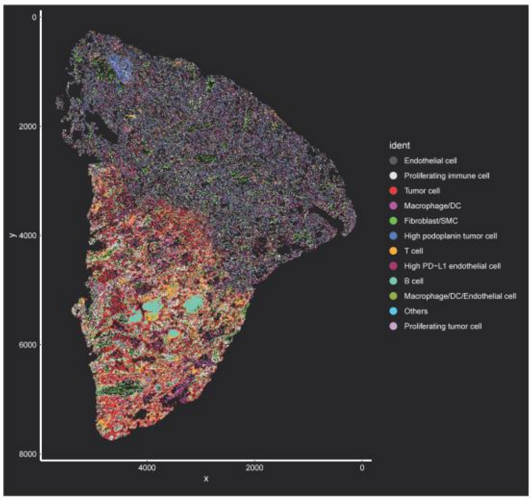

**Supplementary Figure S15 Representative images and cell phenotyping results of PhenoCycler data in AIS/MIA cases**

**(a–f)** Representative images of PhenoCycler results in the FF sections of AIS and MIA cases: TSU-23, TSU-24, TSU-35, TSU-28, TSU-30, and TSU-31. PhenoCycler signals of DAPI and eight proteins are shown. ROIs were chosen based on their proximity to the "possibly malignant" region or their abundance of proliferative or invasive spots. Each section includes the entire image as well as the ROIs. **(g–o)** Clustering and annotation of PhenoCycler data for cell phenotyping of AIS/MIA cases (TSU-20, TSU-23, TSU-21, TSU-24, TSU-35, TSU-28, TSU-30, TSU-31 and TSU-33). The data from the previous study[6] was used in three cases (TSU-20, TSU-21 and TSU-33). The top panel displays a UMAP plot of the clustering results as well as a table with cell phenotype annotations for each cluster. The table includes the differentially expressed proteins of each cluster (top 3 with log2FC > 0.5; proteins with log2FC > 2 are bold). The middle panel shows violin plots of representative cell type markers. The UMAP and spatial plot with cell phenotyping information are shown in the bottom panel.

## TSU-23 (AIS, Noguchi type A)

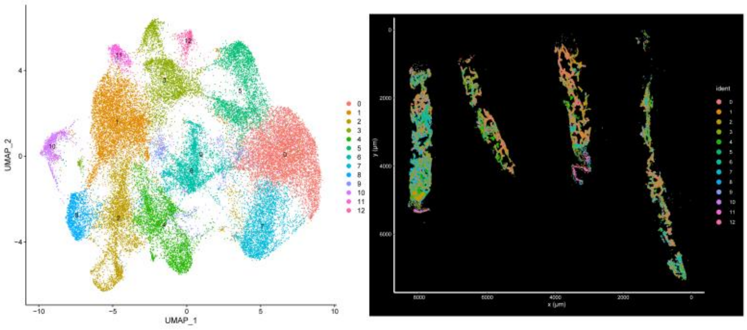


| Cluster | Annotation |
|---|---|
| 0 | Differentiated tumor cell |
| 1 | Fibroblast |
| 2 | T cell, B cell |
| 3 | Endothelial cell |
| 4 | Macrophage |
| 5 | Differentiated tumor cell |
| 6 | Others |
| 7 | Differentiated tumor cell |
| 8 | Plasma cell |
| 9 | Others |
| 10 | Mastocyte |
| 11 | VMSC, myofibroblast |
| 12 | Artery endothelial cell |

## TSU-25 (AIS, Noguchi type A)

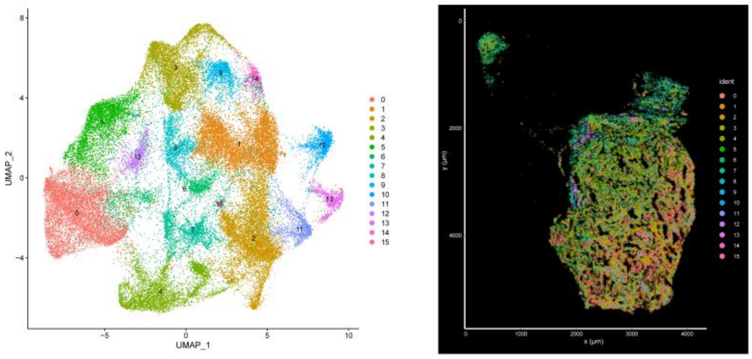


| Cluster | Annotation |
|---|---|
| 0 | Differentiated tumor cell |
| 1 | Fibroblast |
| 2 | T cell |
| 3 | Endothelial cell |
| 4 | Macrophage |
| 5 | Differentiated tumor cell |
| 6 | Others |
| 7 | DC |
| 8 | Others (IL6-high) |
| 9 | Pericyte |
| 10 | Mastocyte |
| 11 | Plasma cell |
| 12 | Others (AT1, VEGFA-high) |
| 13 | LEC |
| 14 | VMSC, myofibroblast |
| 15 | pDC |

## TSU-24 (AIS, Noguchi type B)

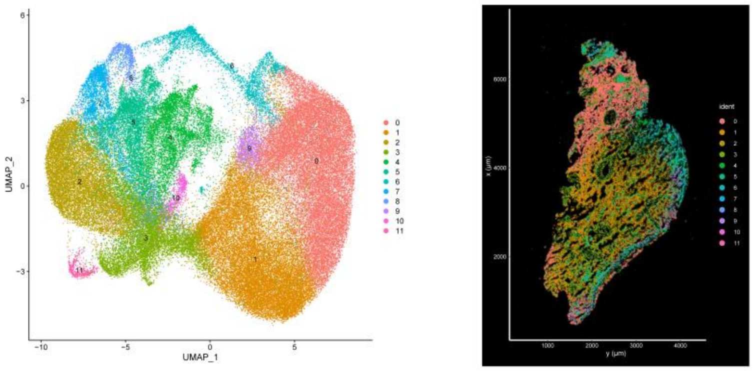


| Cluster | Annotation |
|---|---|
| 0 | Differentiated tumor cell |
| 1 | Differentiated tumor cell |
| 2 | Fibroblast |
| 3 | Others |
| 4 | Macrophage |
| 5 | T cell |
| 6 | Differentiated tumor cell |
| 7 | Endothelial cell |
| 8 | Others (IL6-high) |
| 9 | IDO1-expressing tumor cell |
| 10 | Mastocyte |
| 11 | LEC |

## TSU-35 (AIS, Noguchi type B)

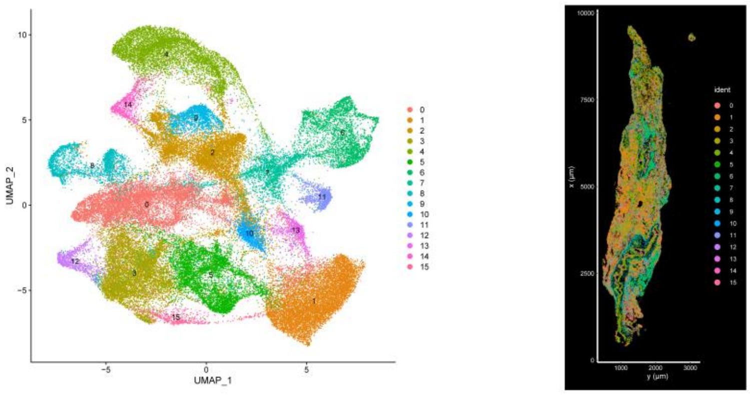


| Cluster | Annotation |
|---|---|
| 0 | Fibroblast |
| 1 | Differentiated tumor cell |
| 2 | Endothelial cell |
| 3 | T cell |
| 4 | VMSC, myofibroblast |
| 5 | Macrophage |
| 6 | Bronchiolar epithelial cell |
| 7 | Bronchiolar epithelial cell |
| 8 | LEC |
| 9 | Pericyte |
| 10 | Differentiated tumor cell |
| 11 | Mastocyte |
| 12 | Plasma cell |
| 13 | Others (AT1, VEGFA-high) |
| 14 | Artery endothelial cell |
| 15 | CCL22+ DC |

## TSU-27 (MIA, Noguchi type C)

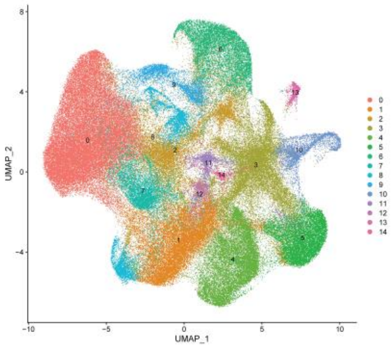


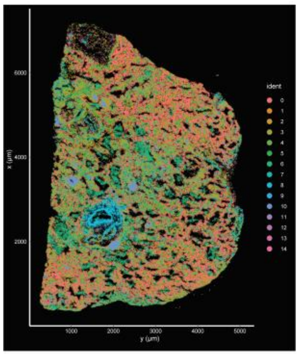


| Cluster | Annotation |
|---|---|
| 0 | IDO1 expressing tumor cell |
| 1 | Fibroblast |
| 2 | Others (tumor cell, macrophage) |
| 3 | T cell |
| 4 | Endothelial cell |
| 5 | Plasma cell |
| 6 | Macrophage |
| 7 | Differentiated tumor cell |
| 8 | Bronchiolar epithelial cell |
| 9 | CCL22+ DC |
| 10 | CXCL13+ B cell |
| 11 | Mastocyte |
| 12 | Others (IL6 high, iCAF) |
| 13 | Fibroblast |
| 14 | LEC |

## TSU-28 (MIA, Noguchi type C)

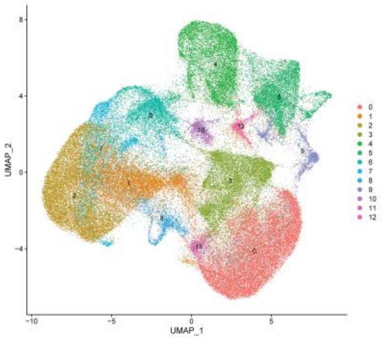


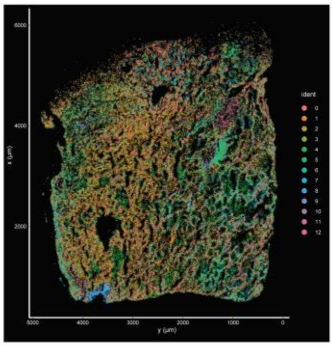


| Cluster | Annotation |
|---|---|
| 0 | Fibroblast |
| 1 | Differentiated tumor cell |
| 2 | Differentiated tumor cell |
| 3 | Endothelial cell |
| 4 | Macrophage |
| 5 | T cell |
| 6 | Differentiated tumor cell |
| 7 | Differentiated tumor cell |
| 8 | Bronchiolar epithelial cell |
| 9 | LEC |
| 10 | Mastocyte |
| 11 | VMSC, myofibroblast |
| 12 | Plasma cell |

## TSU-30 (MIA, Noguchi type C)

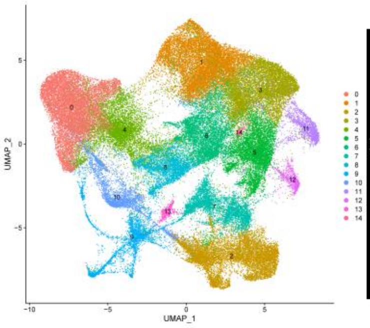


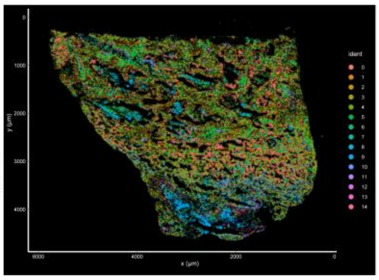


| Cluster | Annotation |
|---|---|
| 0 | Differentiated tumor cell |
| 1 | Fibroblast |
| 2 | Endothelial cell |
| 3 | Fibroblast |
| 4 | Differentiated tumor cell |
| 5 | T cell |
| 6 | DC, macrophage |
| 7 | VMSC, myofibroblast |
| 8 | SPP1+ macrophage |
| 9 | Bronchiolar epithelial cell |
| 10 | Differentiated tumor cell |
| 11 | Mastocyte |
| 12 | LEC |
| 13 | Fibroblast |
| 14 | Plasma cell |

## TSU-33 (MIA, Noguchi type C)

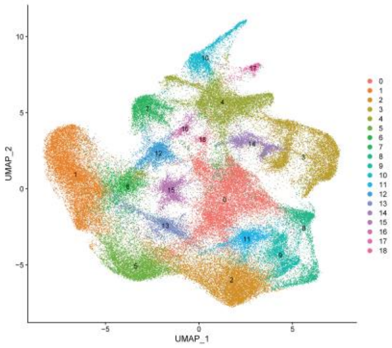


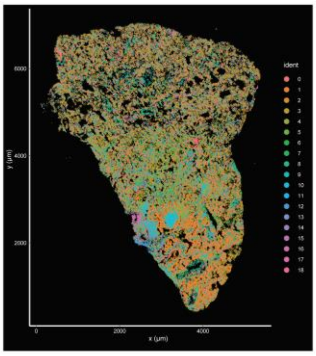


| Cluster | Annotation |
|---|---|
| 0 | Fibroblast |
| 1 | Differentiated tumor cell |
| 2 | Endothelial cell |
| 3 | Macrophage |
| 4 | T cell |
| 5 | Differentiated tumor cell |
| 6 | Differentiated tumor cell |
| 7 | Plasma cell |
| 8 | VMSC, myofibroblast |
| 9 | Endothelial cell |
| 10 | CXCL13+ B cell |
| 11 | Pericyte |
| 12 | Others |
| 13 | Others (AT1, VEGFA-high) |
| 14 | DC |
| 15 | Mastocyte |
| 16 | Bronchiolar epithelial cell |
| 17 | LEC |
| 18 | pDC |

TSU-36 (MIA, Noguchi type C)

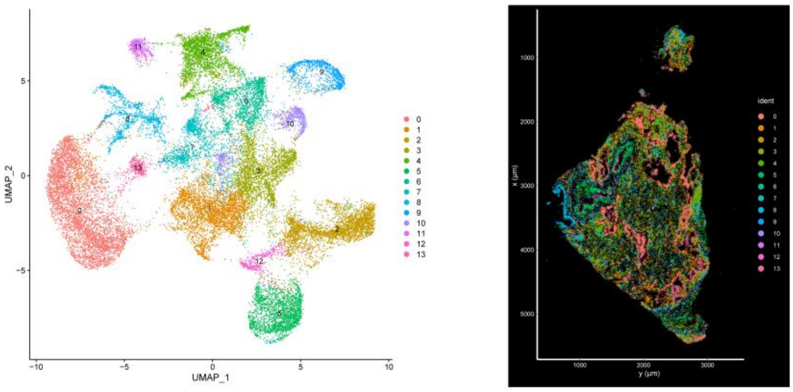


| Cluster | Annotation |
|---|---|
| 0 | Differentiated tumor cell |
| 1 | Fibroblast |
| 2 | Endothelial cell |
| 3 | Fibroblast |
| 4 | T cell |
| 5 | VMSC, myofibroblast |
| 6 | Macrophage |
| 7 | Plasma cell |
| 8 | Bronchiolar epithelial cell |
| 9 | LEC |
| 10 | Fibroblast |
| 11 | Mastocyte |
| 12 | Artery endothelial cell |
| 13 | Macrophage |

TSU-41 (MIA, Noguchi type C)

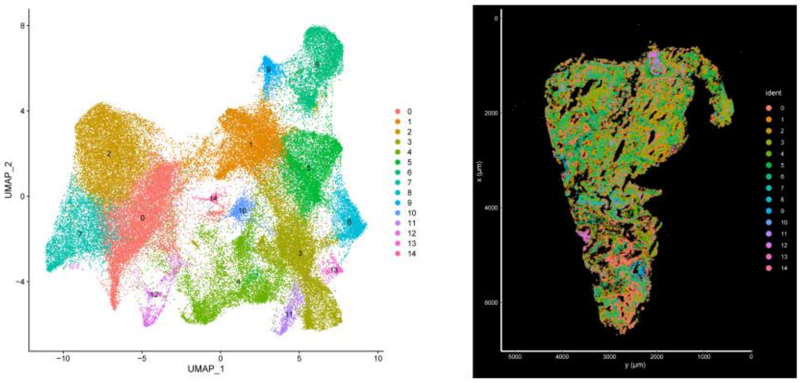


| Cluster | Annotation |
|---|---|
| 0 | Differentiated tumor cell |
| 1 | Fibroblast |
| 2 | Differentiated tumor cell |
| 3 | T cell |
| 4 | Macrophage |
| 5 | Fibroblast |
| 6 | Endothelial cell |
| 7 | Differentiated tumor cell |
| 8 | Plasma cell |
| 9 | VMSC, myofibroblast |
| 10 | Mastocyte |
| 11 | CCL22+ immune cell |
| 12 | Bronchiolar epithelial cell |
| 13 | LEC |
| 14 | Fibroblast |

**Supplementary Figure S16 Spatial and single-cell expression analysis using Xenium for 10 AIS/MIA cases**

Clustering results for Xenium of early adenocarcinoma cases. The left and middle panels show the UMAP plot with cluster annotations and the spatial plot, respectively. The annotation for each cluster is shown in the table to the right.

a

b

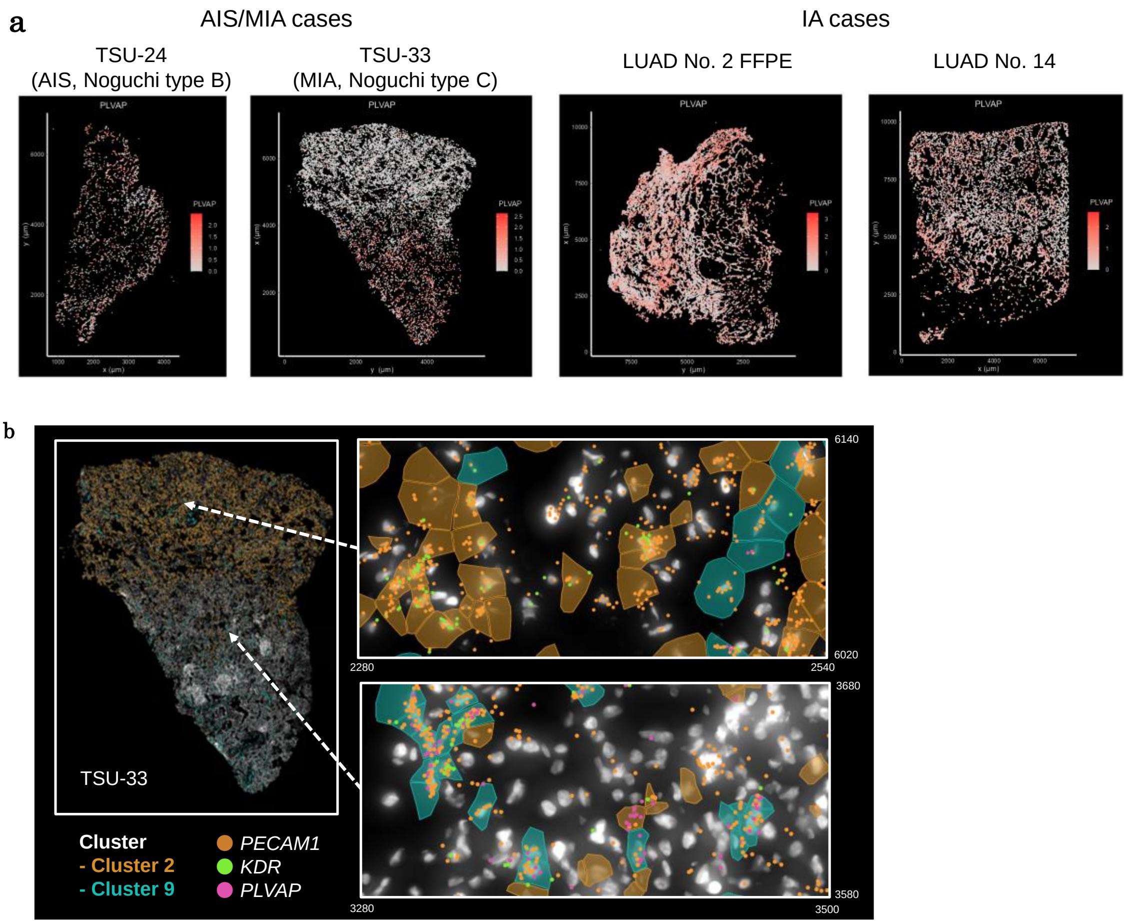


**Supplementary Figure S17 Characterization of endothelial cells in Xenium data**
**(a)** *PLVAP* expression profiles in Xenium data. Endothelial cell clusters are cluster 7 (TSU-24), clusters 2 and 9 (TSU-33), cluster 3 (LUAD No. 2 FFPE), and cluster 5 (LUAD No. 14), as shown in **Supplementary Figures S9** and **S16**. **(b)** The cluster distribution (left) and spatial expression patterns of endothelial cell markers (right) in Xenium are shown for TSU-33. Plots of RNA molecules of two representative regions are enlarged. The plots were rotated and horizontally flipped to match the orientation of Visium.

Note:

To characterize stromal cell features in AIS/MIA and IA cases in addition to immune cells, we focused on endothelial cell phenotypes. It is reported that endothelial cells would differentiate into three types, in response to angiogenic signals: migratory tip cells for the guide of vessel sprouts, proliferating stalk cells for extending sprouts, and quiescent phalanx cells which are need to line new vessels[7,8]. We focused on expression

patterns of *PLVAP*, which is a common marker of tumor endothelial cells and expressed in immature stalk cells[9]. In TSU-33 which harbors two endothelial cell clusters, cluster 9 showed differentially higher expression of *PLVAP* than cluster 2. Cluster 9 cells are located in more invasive regions (lower parts of the tissue section, including the "possibly malignant-invasive" region) while most of cluster 2 cells are in the top part of the tissue where tumors represented transcriptionally/histologically less malignant features, which indicate that cluster 9 cells might be tumor endothelial cells associated with angiogenesis. However, in other cases, endothelial cells widely expressed *PLVAP*. The results indicate that angiogenesis would occur with tumor endothelial cells and begin from early phases of lung cancer cases. Further evaluation will be needed to extend characterization of endothelial signaling in lung cancer progression.

## Supplementary Tables

### Supplementary Table S1 Number of spatial omics datasets used in this study

| Case | Pathological stage | Smoking history | Driver gene | FF/FFPE | Spatial omics analysis | | |
|---|---|---|---|---|---|---|---|
| | | | | | Visium | PhenoCycler | Xenium |
| IA | | | | | | | |
| LUAD No. 1 | IA3 | n | *EGFR* | FF | 1 | 0 | 0 |
| LUAD No. 2 | IIB | y | *KRAS* | FF | 1 | 0 | 0 |
| | | | | FFPE | 4 | 1 | 1 |
| LUAD No. 3 | IA3 | n | *EGFR* | FF | 1 | 0 | 0 |
| | | | | FFPE | 2 | 1 | 1 |
| LUAD No. 4 | IB | n | *EGFR* | FF | 1 | 0 | 0 |
| | | | | FFPE | 2 | 1 | 1 |
| LUAD No. 5 | IIB | n | *EGFR* | FF | 1 | 0 | 0 |
| LUAD No. 14 | IVA | y | *KRAS* | FF | 1 | 0 | 1 |
| LUAD No. 16 | IA3 | y | *EGFR* | FF | 1 | 0 | 1 |
| LUAD No. 17 | IIIA | n | *EGFR* | FF | 1 | 0 | 1 |
| AIS, Noguchi type A | | | | | | | |
| TSU-20 | 0 | n | *MAP2K1* | FF | 1 (1)* | (1)* | (1)* |
| TSU-23 | 0 | n | *EGFR* | FF | 1 | 1 | 1 |
| TSU-25 | 0 | y | *EGFR* | FF | 1 | 0 | 1 |
| AIS, Noguchi type B | | | | | | | |
| TSU-19 | 0 | y | *EGFR* | FF | 4 | 0 | 0 |
| TSU-21 | 0 | n | *EGFR* | FF | (1)* | (1)* | (1)* |
| TSU-22 | 0 | y | *EGFR* | FF | 1 | 0 | 0 |
| TSU-24 | 0 | y | *EGFR* | FF | 1 | 1 | 1 |
| TSU-26 | 0 | n | *EGFR* | FF | 1 | 0 | 0 |
| TSU-35 | 0 | n | *EGFR* | FF | 1 | 1 | 1 |
| MIA, Noguchi type C | | | | | | | |
| TSU-27 | IA1 | n | *EGFR* | FF | 2 | 0 | 1 |
| TSU-28 | IA1 | n | *EGFR* | FF | 2 | 1 | 1 |
| TSU-30 | IA1 | n | *EGFR* | FF | 1 | 1 | 1 |
| TSU-31 | IA1 | y | *EGFR* | FF | 1 | 1 | 0 |

| | | | | | | | |
|---|---|---|---|---|---|---|---|
| TSU-32 | IA1 | y | *EGFR* | FF | 1 | 0 | 0 |
| TSU-33 | IA1 | y | *EGFR* | FF | (1)* | (1)* | 1 |
| TSU-34 | IA1 | n | - | FF | 1 | 0 | 0 |
| TSU-36 | IA1 | n | *EGFR* | FF | 1 | 0 | 1 |
| TSU-37 | IA1 | n | *EGFR* | FF | 1 | 0 | 0 |
| TSU-38 | IA1 | n | *EGFR* | FF | 1 | 0 | 0 |
| TSU-39 | IA1 | y | *MET* | FF | 1 | 0 | 0 |
| TSU-40 | IA1 | n | *EGFR* | FF | 1 | 0 | 0 |
| TSU-41 | IA1 | n | *EGFR* | FF | 1 | 0 | 1 |

Visium data of LUAD No. 2 FFPE were obtained from four serial sections. Visium data of LUAD No. 3 FFPE, LUAD No. 4 FFPE, TSU-20, TSU-27 and TSU-28 were obtained from two serial sections. Four Visium data of TSU-19 were obtained from the different regions.

*These datasets were previously reported[6].

Note:

For LUAD No. 2, we planned to compare the results of FF and FFPE samples. In some cases, specimens were collected only when either preparation was available. In fact, in Visium analysis, the experimental protocols for FF and FFPE differ significantly. Both procedures have advantages and disadvantages. FFPE has better tissue morphology and histology than FF. However, when it comes to biochemical assays, such as full-length RNA sequencing and epigenome analyses, FF samples are far more suitable. In FFPE samples, RNA is generally degraded, so a probe-based approach is used to capture transcriptome profiles. For another example of the study design, for some specimens, several sections were prepared to test the reproducibility of data collection (using consecutive sections) and the horizontal spread of the profiles (using adjacent sections).

LUAD No. 2 and 3 were initially selected because they have typical driver mutations (*KRAS* and *EGFR*, respectively) and exhibit diverse local expression profiles. Those specimens were then chosen for the PhenoCycler and Xenium analyses because we believed that the interactions between the presumed cell types, based on the marker expression patterns in the Visium analysis, should be confirmed at the single-cell level. For other cases, we prioritized cases with both well-differentiated and malignant expression features that were more heterogeneous. It is also true that, in some cases, we could not conduct a thorough analysis because the remaining tissues had been exhausted (e.g., we had no remaining tissues of LUAD No. 4 FFPE for performing Xenium analysis).

## Supplementary Table S2 General statistics of Visium in IA cases

| Case | FF/FFPE | Number of spots | Number of reads | Mean reads per spot | Median genes per spot | Median UMI counts per spot | Sequencing saturation |
|---|---|---|---|---|---|---|---|
| LUAD No. 1 | FF | 2,649 | 474,827,182 | 179,248 | 4,394 | 11,803 | 0.87 |
| LUAD No. 2 | FF | 2,226 | 486,607,210 | 218,602 | 5,530 | 18,788 | 0.87 |
| LUAD No. 3 | FF | 3,804 | 472,542,245 | 124,222 | 2,579 | 6,597 | 0.90 |
| LUAD No. 4 | FF | 3,854 | 455,530,400 | 118,197 | 2,461 | 5,174 | 0.91 |
| LUAD No. 5 | FF | 4,143 | 382,522,043 | 92,330 | 3,144 | 7,728 | 0.84 |
| LUAD No. 14 | FF | 4,630 | 417,203,997 | 90,109 | 3,263 | 7,738 | 0.85 |
| LUAD No. 16 | FF | 3,640 | 426,493,578 | 117,169 | 2,031 | 3,937 | 0.91 |
| LUAD No. 17 | FF | 2,179 | 455,397,893 | 208,994 | 2,050 | 5,019 | 0.94 |
| LUAD No. 2, A | FFPE | 2,575 | 315,922,331 | 122,688 | 9,975 | 59,403 | 0.48 |
| LUAD No. 2, B | FFPE | 3,854 | 355,786,830 | 92,316 | 9,192 | 42,861 | 0.49 |
| LUAD No. 2, C | FFPE | 4,666 | 243,622,025 | 52,212 | 8,168 | 31,746 | 0.32 |
| LUAD No. 2, D | FFPE | 4,596 | 336,240,758 | 73,159 | 8,289 | 32,632 | 0.37 |
| LUAD No. 3, A | FFPE | 2,099 | 282,276,626 | 134,481 | 4,895 | 10,892 | 0.88 |
| LUAD No. 3, B | FFPE | 2,876 | 266,006,934 | 92,492 | 4,104 | 8,391 | 0.84 |
| LUAD No. 4, C | FFPE | 3,243 | 165,416,495 | 51,007 | 6,246 | 17,624 | 0.54 |
| LUAD No. 4, D | FFPE | 2,941 | 193,834,742 | 65,908 | 7,570 | 25,442 | 0.54 |

**Supplementary Table S3 General statistics of Visium in AIS/MIA cases**

| Case | Number of spots | Number of reads | Mean reads per spot | Median genes per spot | Median UMI counts per spot | Sequencing saturation |
|---|---|---|---|---|---|---|
| AIS, Noguchi type A | | | | | | |
| TSU-20 #1 | 737 | 467,436,361 | 634,242 | 3,833 | 10,021 | 0.96 |
| TSU-23 | 638 | 569,456,998 | 892,566 | 3,536 | 9,510 | 0.96 |
| TSU-25 | 1,069 | 531,049,007 | 496,772 | 2,018 | 3,457 | 0.98 |
| AIS, Noguchi type B | | | | | | |
| TSU-19 a-1 | 2,256 | 623,161,699 | 276,224 | 2,277 | 4,355 | 0.97 |
| TSU-19 a-2 | 2,526 | 768,602,464 | 304,277 | 1,547 | 2,597 | 0.98 |
| TSU-19 b-1 | 3,083 | 636,194,448 | 206,356 | 1,577 | 2,872 | 0.98 |
| TSU-19 b-2 | 3,519 | 651,275,192 | 185,074 | 1,654 | 2,852 | 0.97 |
| TSU-22 | 764 | 826,932,037 | 1,082,372 | 3,422 | 10,011 | 0.99 |
| TSU-24 | 1,161 | 599,708,658 | 516,545 | 3,587 | 8,324 | 0.97 |
| TSU-26 | 871 | 666,957,994 | 765,738 | 2,389 | 5,843 | 0.98 |
| TSU-35 | 1,024 | 403,958,649 | 394,491 | 2,520 | 4,687 | 0.97 |
| MIA, Noguchi type C | | | | | | |
| TSU-27 #1 | 3,046 | 271,416,110 | 89,106 | 2,182 | 3,977 | 0.87 |
| TSU-27 #2 | 2,839 | 225,147,862 | 79,305 | 2,514 | 5,268 | 0.86 |
| TSU-28 #1 | 2,441 | 405,565,409 | 166,147 | 2,627 | 4,541 | 0.91 |
| TSU-28 #2 | 1,834 | 188,769,395 | 102,928 | 2,446 | 4,315 | 0.88 |
| TSU-30 | 1,853 | 885,669,797 | 477,965 | 2,071 | 4,068 | 0.98 |
| TSU-31 | 1,140 | 553,622,646 | 485,634 | 2,886 | 6,593 | 0.97 |
| TSU-32 | 2,288 | 542,335,550 | 237,035 | 1,722 | 3,380 | 0.97 |
| TSU-34 | 1,449 | 295,738,028 | 204,098 | 1,042 | 1,519 | 0.97 |
| TSU-36 | 748 | 414,866,084 | 554,634 | 1,329 | 2,362 | 0.99 |
| TSU-37 | 813 | 533,601,580 | 656,337 | 1,777 | 3,123 | 0.99 |
| TSU-38 | 1,093 | 726,121,219 | 664,338 | 2,009 | 3,833 | 0.98 |
| TSU-39 | 1,662 | 223,231,157 | 134,315 | 2,199 | 4,086 | 0.93 |
| TSU-40 | 2,613 | 699,359,814 | 267,646 | 2,905 | 6,254 | 0.96 |
| TSU-41 | 1,458 | 753,321,845 | 516,682 | 2,358 | 5,201 | 0.98 |

All datasets were obtained from FF samples. Datasets #1 and #2 were derived from serial sections of the same block. Data a-1, a-2, b-1, and b-2 of TSU-19 were obtained from

different tissue regions. The quality of TSU-23 (needle biopsy) data apperars to be lower, as only 49.2% of reads were assigned to tissue-covered spots.
Data from three sections, TSU-20 (#2), TSU-21, and TSU-33, were previously published[6].

**Supplementary Table S4 General information of PhenoCycler for FFPE samples of IA cases**

## (a) List of antibodies, barcodes, and reporters

PhenoCycler inventories

| Molecule | Conjugated-antibody catalog number | Clone | Barcode | Fluorescence |
|---|---|---|---|---|
| CD31 | 4450017 | EP3095 | BX001 | AF750 |
| CD20 | 4450018 | L26 | BX007 | AF750 |
| beta-actin | 4450040 | W16197A | BX010 | AF750 |
| Pan-Cytokeratin | 4450020 | AE-1/AE-3 | BX019 | AF750 |
| CD44 | 4450041 | IM7 | BX005 | Atto 550 |
| E-cadherin | 4250021 | 4A2C7 | BX014 | Atto 550 |
| CD45RO | 4250023 | UCHL1 | BX017 | Atto 550 |
| ß-catenin1 | 4450036 | 12F7 | BX020 | Atto 550 |
| Podoplanin | 4250004 | NC-08 | BX023 | Atto 550 |
| CD8 | 4250012 | C8/144B | BX026 | Atto 550 |
| Mac2/Galectin-3 | 4450034 | M3/38 | BX035 | Atto 550 |
| Ki67 | 4250019 | B56 | BX047 | Atto 550 |
| CD4 | 4550112 | EPR6855 | BX003 | Cy5 / AF647 |
| LIF | 4550120 | M1506B09 | BX006 | Cy5 / AF647 |
| CD68 | 4550113 | KP1 | BX015 | Cy5 / AF647 |
| CD11c | 4550114 | 118/A5 | BX024 | Cy5 / AF647 |
| IDO1 | 4550123 | V1NC3IDO | BX027 | Cy5 / AF647 |
| CD3e | 4550119 | EP449E | BX045 | Cy5 / AF647 |

Custom conjugated

| Molecule | Antibody catalog number | Clone | Barcode | Fluorescence |
|---|---|---|---|---|
| α-SMA/ACTA2 | ab7817 (Abcam) | 1A4 | BX004 | AF750 |
| Collagen-IV | ab226485 (Abcam) | EPR20966 | BX013 | AF750 |
| HLA-A | ab216653 (Abcam) | EP1395Y | BX016 | AF750 |
| HLA-DPB1 | ab215973 (Abcam) | EPR11226 | BX022 | AF750 |
| TP53 | 554169 (BD Pharmingen) | Pab 1801 | BX025 | AF750 |
| CD163 | ab213612 (Abcam) | EPR19518 | BX028 | AF750 |
| CD278/ICOS | ab236226 (Abcam) | SP98 | BX031 | AF750 |
| TIGIT | ab243903 (Abcam) | BLR047F | BX037 | AF750 |
| DC-LAMP | DDX0191P-100 (Eurobio) | 1010E1.01 | BX029 | Atto 550 |

| | | | | |
|---|---|---|---|---|
| CD274/PD-L1 | ab228413 (Abcam) | 28-8 | BX041 | Atto 550 |
| FOXP3 | 14-4777-82 (Invitrogen) | 236A/E7 | BX052 | Atto 550 |
| CD56 | ab214435 (Abcam) | EP2567Y | BX055 | Atto 550 |
| CD223/LAG3 | #15372 (CST) | D2G4O | BX021 | Cy5 / AF647 |
| BCA1/CXCL13 | ab270408 (Abcam) | EPR23400-92 | BX030 | Cy5 / AF647 |
| CTLA4 | NB100-64849 (Novus) | BNI3 | BX033 | Cy5 / AF647 |
| TTF-1/NKX2-1 | ab212886 (Abcam) | 8G7G3/1 | BX042 | Cy5 / AF647 |
| CD279/PD-1 | ab186928 (Abcam) | EPR4877(2) | BX054 | Cy5 / AF647 |

## (b) Run design

| Cycle | DAPI | Atto 550 | | Cy5/AF647 | | AF750 | |
|---|---|---|---|---|---|---|---|
| | | Reporter No. | Molecule | Reporter No. | Molecule | Reporter No. | Molecule |
| 1 | DAPI | Blank | — | Blank | — | Blank | — |
| 2 | DAPI | RX005 | CD44 | RX003 | CD4 | RX001 | CD31 |
| 3 | DAPI | RX014 | E-cadherin | RX006 | LIF | RX004 | α-SMA/ACTA2 |
| 4 | DAPI | RX017 | CD45RO | RX015 | CD68 | RX007 | CD20 |
| 5 | DAPI | RX020 | β-Catenin1 | RX021 | CD223/LAG3 | RX010 | Beta-actin |
| 6 | DAPI | RX023 | Podoplanin | RX024 | CD11c | RX013 | Collagen-IV |
| 7 | DAPI | RX026 | CD8 | RX027 | IDO1 | RX016 | HLA-A |
| 8 | DAPI | RX029 | DC-LAMP | RX030 | BCA1/CXCL13 | RX019 | Pan-Cytokeratin |
| 9 | DAPI | RX035 | Mac2/Galectin-3 | RX033 | CTLA4 | RX022 | HLA-DPB1 |
| 10 | DAPI | RX041 | CD274/PD-L1 | RX042 | TTF-1/NKX2-1 | RX025 | TP53 |
| 11 | DAPI | RX047 | Ki67 | RX045 | CD3e | RX028 | CD163 |
| 12 | DAPI | RX052 | FOXP3 | RX054 | CD279/PD-1 | RX031 | CD278/ICOS |
| 13 | DAPI | RX055 | CD56 | Empty | — | RX037 | TIGIT |
| 14 | DAPI | Blank | — | Blank | — | Blank | — |

**Supplementary Table S5 General information of PhenoCycler for FF samples of AIS/MIA cases**

**(a) List of antibodies, barcodes, and reporters**

| PhenoCycler inventoried | | | | | Run set* | |
|---|---|---|---|---|---|---|
| Molecule | Conjugated-antibody catalog number | Clone | Barcode | Florescence | Set #1 | Set #2 |
| CD19 | 4550099 | HIB19 | BX003 | AF647 | y | y |
| CD8 | 4150004 | SK1 | BX004 | AF488 | y | y |
| CD279/PD-1 | 4250010 | EH12.2H7 | BX014 | Atto 550 | y | y |
| CD68 | 4350019 | KP1 | BX015 | Cy5 | | y |
| CD45RO | 4250023 | UCHL1 | BX017 | Atto 550 | y | y |
| Pan-Cytokeratin | 4150020 | AE-1/AE-3 | BX019 | AF488 | y | y |
| CD4 | 4350010 | SK3 | BX021 | Cy5 | y | y |
| Podoplanin | 4250004 | NC-08 | BX023 | Atto 550 | y | y |
| CD11c | 4550107 | S-HCL-3 | BX027 | AF647 | y | y |
| CD31 | 4250009 | WM59 | BX032 | Atto 550 | y | y |
| Mac2/Galectin-3 | 4450034 | M3/38 | BX035 | Atto 550 | | y |
| Ki67 | 4250019 | B56 | BX047 | Atto 550 | y | y |
| Custom | | | | | Run set | |
| Molecule | Antibody catalog number | Clone | Barcode | Florescence | Set #1 | Set #2 |
| TP53 | 554169 (BD Pharmingen) | Pab 1801 | BX025 | AF488 | y | y |
| CD163 | ab213612 (Abcam) | EPR19518 | BX028 | AF488 | y | y |
| BCA1/CXCL13 | ab270408 (Abcam) | EPR23400-92 | BX030 | Cy5 | | y |
| CTLA4 | NB100-64849 (Novus Biologicals) | BNI3 | BX033 | Cy5 | | y |
| CD274/PD-L1 | ab228413 (Abcam) | 28-8 | BX041 | Atto 550 | | y |
| TTF-1/NKX2-1 | ab242428 (Abcam) | SP141 | BX050 | AF647 | y | y |
| FOXP3 | #14-4777-82 (Invitrogen) | 236A/E7 | BX052 | Atto 550 | y | y |
| α-SMA/ACTA2 | ab7817 (Abcam) | 1A4 | BX054 | Cy5 | y | y |
| CD56/NCAM1 | ab214435 (Abcam) | EP2567Y | BX055 | Atto 550 | | y |

Run set #1 was used for TSU-23, TSU-28, and TSU-31. Run set #2 was used for TSU-24, TSU-30, and TSU-35.

**(b) Run design for set #1**

| Cycle | DAPI | Atto 550 | | Cy5/AF647 | | AF488 | |
|---|---|---|---|---|---|---|---|
| | | Reporter No. | Molecule | Reporter No. | Molecule | Reporter No. | Molecule |
| 1 | DAPI | Blank | --- | Blank | --- | Blank | --- |
| 2 | DAPI | RX014 | CD279/PD-1 | RX003 | CD19 | RX004 | CD8 |
| 3 | DAPI | RX017 | CD45RO | RX021 | CD4 | RX019 | Pan-Cytokeratin |
| 4 | DAPI | RX023 | Podoplanin | RX027 | CD11c | RX025 | TP53 |
| 5 | DAPI | RX032 | CD31 | RX050 | TTF-1/NKX2-1 | BX028 | CD163 |
| 6 | DAPI | RX047 | Ki67 | RX054 | α-SMA/ACTA2 | Empty | --- |
| 7 | DAPI | RX052 | FOXP3 | Empty | --- | Empty | --- |
| 8 | DAPI | Blank | --- | Blank | --- | Blank | --- |

**(c) Run design for set #2**

| Cycle | DAPI | Atto 550 | | Cy5/AF647 | | AF488 | |
|---|---|---|---|---|---|---|---|
| | | Reporter No. | Molecule | Reporter No. | Molecule | Reporter No. | Molecule |
| 1 | DAPI | Blank | --- | Blank | --- | Blank | --- |
| 2 | DAPI | RX014 | CD279/PD-1 | RX003 | CD19 | RX004 | CD8 |
| 3 | DAPI | RX017 | CD45RO | BX015 | CD68 | RX019 | Pan-Cytokeratin |
| 4 | DAPI | RX023 | Podoplanin | RX021 | CD4 | RX025 | TP53 |
| 5 | DAPI | RX032 | CD31 | RX027 | CD11c | BX028 | CD163 |
| 6 | DAPI | BX035 | Mac2/Galectin-3 | BX030 | BCA1/CXCL13 | Empty | --- |
| 7 | DAPI | BX041 | CD274/PD-L1 | BX033 | CTLA4 | Empty | --- |
| 8 | DAPI | RX047 | Ki67 | RX050 | TTF-1/NKX2-1 | Empty | --- |
| 9 | DAPI | RX052 | FOXP3 | RX054 | α-SMA/ACTA2 | Empty | --- |
| 10 | DAPI | BX055 | CD56/NCAM1 | Empty | --- | Empty | --- |
| 11 | DAPI | Blank | --- | Blank | --- | Blank | --- |

## Supplementary Table S6 General statistics of Xenium in IA cases

| Case | Region area (μm²) | Total cell area (μm²) | Fraction of transcripts decoded q20 | Decoded transcripts per 100 μm² | Number of cells detected | Median genes per cell | Median transcripts per cell |
|---|---|---|---|---|---|---|---|
| LUAD No. 2 (FFPE) | 74,526,696 | 51,690,317 | 0.87 | 161.6 | 472,420 | 53 | 152 |
| LUAD No. 3 (FFPE) | 40,266,013 | 22,894,703 | 0.81 | 53.1 | 150,055 | 31 | 50 |
| LUAD No. 14 (FF) | 69,333,431 | 48,584,499 | 0.75 | 89.7 | 309,940 | 45 | 102 |
| LUAD No. 16 (FF) | 46,729,502 | 35,272,599 | 0.85 | 96 | 294,386 | 36 | 75 |
| LUAD No. 17 (FF) | 32,817,217 | 17,305,433 | 0.79 | 79.1 | 96,749 | 41 | 107 |

**Supplementary Table S7 General statistics of Xenium in AIS/MIA cases**

| | Region area (μm²) | Total cell area (μm²) | Fraction of transcripts decoded q20 | Decoded transcripts per 100 μm² | Number of cells detected | Median genes per cell | Median transcripts per cell |
|---|---|---|---|---|---|---|---|
| | | | AIS, Noguchi type A | | | | |
| TSU-23 | 27,551,678 | 5,836,253 | 0.80 | 92.9 | 38,219 | 45 | 112 |
| TSU-25 | 17,612,095 | 8,171,814 | 0.79 | 108.5 | 48,942 | 52 | 148 |
| | | | AIS, Noguchi type B | | | | |
| TSU-24 | 22,792,052 | 11,220,056 | 0.80 | 91.1 | 91,457 | 37 | 87 |
| TSU-35 | 20,302,622 | 9,640,210 | 0.82 | 108.7 | 67,699 | 50 | 136 |
| | | | MIA, Noguchi type C | | | | |
| TSU-27 | 33,728,086 | 23,518,171 | 0.79 | 159.3 | 173,219 | 61 | 180 |
| TSU-28 | 28,900,204 | 19,153,553 | 0.85 | 68.8 | 104,968 | 39 | 88 |
| TSU-30 | 23,686,133 | 14,794,324 | 0.83 | 89.3 | 84,611 | 46 | 132 |
| TSU-33 | 30,232,430 | 18,764,465 | 0.81 | 62.2 | 100,199 | 43 | 97 |
| TSU-36 | 13,673,489 | 5,616,139 | 0.83 | 48.0 | 26,817 | 33 | 82 |
| TSU-41 | 21,550,217 | 11,274,866 | 0.82 | 114.9 | 74,186 | 52 | 146 |

All datasets were obtained from FF samples.

Data from two sections, TSU-20 and TSU-21, were previously reported[6].

**Supplementary Table S8 List of genes and parameters for calculating TME scores and evaluation of their changes**

**(a) Signature genes for tumor cells**

| Cell type (epithelial/tumor) | Gene |
|---|---|
| Well-differentiated | *CAPS, SNTN, HOPX, SFTPC, SFTPB, SCGB3A2, NAPSA, SCGB1A1, SCGB3A1, SFTPA1, CLDN18* |
| Proliferative | *WEE1, FOS, FOSB* |
| Invasive | *SPINK1, MMP7, CXCL14, S100A6, TGFBI, TNC* |

**(b) Signature genes for the microenvironment**

| Cell type | Gene |
|---|---|
| Immune-rich (plasma cell) | *IGKC, IGHG1, IGHA1, JCHAIN, IGHG2, IGLC1, CRISPLD2, IGKV4-1, IGHM, IGHG3, IGHD, IGKV4-1, IGLC1, IGHG4* |
| B cell | *CD79A, CD79B, MS4A1, CXCR4* |
| T cell | *CD2, CD3D, CD3E, CD3G, CD8A, CD4* |
| Fibroblast/CAF | *DCN, COL1A1, COL1A2, ACTA2, MYH11* |
| Endothelial cell | *CLDN5, PECAM1, VWF, AQP1* |
| M1 Macrophage | *CD68, CXCL9, APOE* |
| M2 Macrophage (anti-inflammatory) | *CD163, C1QC, SPP1* |

**(c) Parameters for extracting the regions with TME changes**

| Trajectory path | Cluster path | Slope threshold | Regions with TME change | |
|---|---|---|---|---|
| | | | All | Overlap |
| LUAD No. 2, B (FFPE) | 8 -> 1 -> 2 -> 3 -> 0 -> 10 -> 4 | 0.02 | 10 | 5 |
| LUAD No. 2, C (FFPE) | 8 -> 1 -> 2 -> 3 -> 0 -> 10 -> 4 | 0.02 | 10 | 5 |
| LUAD No. 3, B (FFPE) P1 | 9 -> 6 -> 3 | 0.04 | 4 | 4 |
| LUAD No. 3, B (FFPE) P2 | 9 -> 10 | 0.03 | 3 | 3 |
| LUAD No. 14 P1 | 4 -> 5 -> 8 -> 10 -> 13 -> 0 -> 15 | 0.02 | 10 | 5 |
| LUAD No. 14 P2 | 4 -> 5 -> 0 -> 15 | 0.02 | 10 | 5 |
| LUAD No. 14 P3 | 14 -> 11 -> 2 -> 12 -> 6 | 0.045 | 5 | 5 |
| LUAD No. 16 | 9 -> 7 -> 10 | 0.035 | 5 | 5 |

## Supplementary References


1. Gu, Z., Eils, R. & Schlesner, M. Complex heatmaps reveal patterns and correlations in multidimensional genomic data. *Bioinformatics* **32**, 2847–2849 (2016).
2. Jin, S. *et al.* Inference and analysis of cell-cell communication using CellChat. *Nat. Commun.* **12**, 1088 (2021).
3. Dries, R. *et al.* Giotto: a toolbox for integrative analysis and visualization of spatial expression data. *Genome Biol.* **22**, 78 (2021).
4. Miller, B. F., Huang, F., Atta, L., Sahoo, A. & Fan, J. Reference-free cell type deconvolution of multi-cellular pixel-resolution spatially resolved transcriptomics data. *Nat. Commun.* **13**, 2339 (2022).
5. Zhou, Y. *et al.* Metascape provides a biologist-oriented resource for the analysis of systems-level datasets. *Nat. Commun.* **10**, 1523 (2019).
6. Haga, Y. *et al.* Whole-genome sequencing reveals the molecular implications of the stepwise progression of lung adenocarcinoma. *Nat. Commun.* **14**, 8375 (2023).
7. Zeng, Q. *et al.* Understanding tumour endothelial cell heterogeneity and function from single-cell omics. *Nat. Rev. Cancer* **23**, 544–564 (2023).
8. Korn, C. & Augustin, H. G. Mechanisms of Vessel Pruning and Regression. *Dev. Cell* **34**, 5–17 (2015).
9. Goveia, J. *et al.* An Integrated Gene Expression Landscape Profiling Approach to Identify Lung Tumor Endothelial Cell Heterogeneity and Angiogenic Candidates. *Cancer Cell* **37**, 21-36.e13 (2020).
