# Supplementary material for: Spatially resolved gene expression profiling of tumor microenvironment reveals key steps of lung adenocarcinoma development
Source: Nat Commun. 2024 Dec 6;15:10637. doi: 10.1038/s41467-024-54671-7 (PMC11621540; doi:10.1038/s41467-024-54671-7)
Supplement: Supplementary file 3 — Description of Additional Supplementary Files [file 41467_2024_54671_MOESM3_ESM.pdf]

<u>Description of Additional Supplementary Files</u>

Supplementary Data S1: The list of 302 target genes in the custom human lung cancer panel for Xenium
