# Supplementary material for: Spatially resolved gene expression profiling of tumor microenvironment reveals key steps of lung adenocarcinoma development
Source: Nat Commun. 2024 Dec 6;15:10637. doi: 10.1038/s41467-024-54671-7 (PMC11621540; doi:10.1038/s41467-024-54671-7)
Supplement: Supplementary file 5 — Reporting Summary [file 41467_2024_54671_MOESM5_ESM.pdf]

nature portfolio

Corresponding author(s): Yutaka Suzuki, Ayako Suzuki

Last updated by author(s): Nov 15, 2024

Reporting Summary

Nature Portfolio wishes to improve the reproducibility of the work that we publish. This form provides structure for consistency and transparency in reporting. For further information on Nature Portfolio policies, see our Editorial Policies and the Editorial Policy Checklist.

Statistics

For all statistical analyses, confirm that the following items are present in the figure legend, table legend, main text, or Methods section.

n/a	Confirmed	
☐	☒	The exact sample size (*n*) for each experimental group/condition, given as a discrete number and unit of measurement
☐	☒	A statement on whether measurements were taken from distinct samples or whether the same sample was measured repeatedly
☐	☒	The statistical test(s) used AND whether they are one- or two-sided<br>*Only common tests should be described solely by name; describe more complex techniques in the Methods section.*
☐	☒	A description of all covariates tested
☐	☒	A description of any assumptions or corrections, such as tests of normality and adjustment for multiple comparisons
☐	☒	A full description of the statistical parameters including central tendency (e.g. means) or other basic estimates (e.g. regression coefficient) AND variation (e.g. standard deviation) or associated estimates of uncertainty (e.g. confidence intervals)
☐	☒	For null hypothesis testing, the test statistic (e.g. *F*, *t*, *r*) with confidence intervals, effect sizes, degrees of freedom and *P* value noted<br>*Give P values as exact values whenever suitable.*
☒	☐	For Bayesian analysis, information on the choice of priors and Markov chain Monte Carlo settings
☒	☐	For hierarchical and complex designs, identification of the appropriate level for tests and full reporting of outcomes
☐	☒	Estimates of effect sizes (e.g. Cohen's *d*, Pearson's *r*), indicating how they were calculated

Our web collection on statistics for biologists contains articles on many of the points above.

Software and code

Policy information about availability of computer code

Data collection

The methods for the data collection are described in the Methods section. The methods are also described as below;

Spatial transcriptome sequencing by Visium
For fresh frozen (FF) tissues, cryosections (10-µm thickness) were placed on the Visium slide (10x Genomics). Fixation, H&E staining, and imaging were performed according to the manufacturer's instructions. Tissue permeabilization was conducted with a 12-min (invasive adenocarcinoma; IA) or 6-min (adenocarcinoma in situ/minimally invasive adenocarcinoma; AIS/MIA) incubation. After the tissues were permeabilized, cDNA synthesis, amplification, and sequencing library preparation were carried out using Visium Spatial Gene Expression Reagent Kits (10x Genomics).
For FFPE tissues, tissue sections at 5-µm thickness were prepared. Deparaffinization, H&E staining, and imaging were performed according to the manufacturer's instructions. Probe hybridization and library preparation were performed using Visium Spatial Gene Expression Reagent Kits for FFPE (10x Genomics) according to the user guide (CG000407, Rev A, 10x Genomics).
NovaSeq 6000 (Illumina) was used for sequencing the prepared libraries.

Multiplexed immunostaining by PhenoCycler
Multiplexed immunostaining was performed using the PhenoCycler system (Akoya Biosciences) according to the manufacturer's instructions. For FFPE tissues, tissue sections were prepared at 5-µm thickness and mounted on a coverslip. The section was deparaffinized, and antigen activation was performed using a pressure cooker for 20 min. Tissue sections were stained with 35 antibodies for 3 h. For FF tissues, tissue sections were prepared at 10-µm thickness and mounted on a coverslip. The sections were fixed and stained with antibodies for 3 h. The prepared sections were washed, and the antibodies were fixed. Imaging analysis was conducted using the PhenoCycler instrument (Akoya Biosciences) and BZ-X810 fluorescence microscope (Keyence).

In situ gene expression analysis by Xenium
Xenium in situ expression analysis was conducted using Xenium Slides & Sample Prep Reagents (PN-1000460, 10x Genomics). FFPE tissues were sectioned at 5-µm thickness and placed on the Xenium slide (10x Genomics) according to the manufacturer's instructions (CG000578, Rev A, 10x Genomics). Deparaffinization and decrosslinking of sectioned tissues were performed according to the manufacturer's instructions (CG000580, Rev A, 10x Genomics). FF tissues were sectioned at 10-µm thickness using a cryostat and placed onto the Xenium slide (10x Genomics) according to the manufacturer's instructions (CG000579, Rev A, 10x Genomics). Fixation and permeabilization were performed (CG000581, Rev A, 10x Genomics). Preparation of the Xenium slide was conducted according to the user guide (CG000582, Rev A, 10x Genomics). Briefly, pre-designed and custom probes were hybridized at 50 °C overnight, washed, ligated, and amplified. Autofluorescence quenching and nuclei staining were performed in the dark. The instrument run was performed using Xenium Analyzer (10x Genomics). A total of 302 target genes were previously reported (Haga Y et al. 2023 Nat Commun).

Data analysis

The softwares used for the data analysis are described in the Methods section.

Computational preprocessing of Visium data:
Space Ranger (versions 1.2.1, 1.3.0, and 1.3.1, 10x Genomics), Seurat (version 4.0.0 and 4.3.0), Monocle 3 (version 1.0.0), COMMOT (version 0.03), Giotto (version 1.1.2), SPATA2 (version 0.1.0), spacexr (version 2.2.1), STdeconvolve (version 1.3.1)

Computational processing of PhenoCycler data:
CODEX Processor (version 1.8), QuPath (version 0.3.2), StarDist (QuPath StarDist extension, version 0.3.2), Seurat (version 5.1.0)

Computational processing of Xenium data:
Xenium Explorer (version 1.1.0, 1.3.0 and 3.0.0, 10x Genomics), Seurat (version 4.3.0)

For manuscripts utilizing custom algorithms or software that are central to the research but not yet described in published literature, software must be made available to editors and reviewers. We strongly encourage code deposition in a community repository (e.g. GitHub). See the Nature Portfolio guidelines for submitting code & software for further information.

Data

Policy information about availability of data

All manuscripts must include a data availability statement. This statement should provide the following information, where applicable:

- Accession codes, unique identifiers, or web links for publicly available datasets
- A description of any restrictions on data availability
- For clinical datasets or third party data, please ensure that the statement adheres to our policy

Newly obtained sequencing and image data are available in the Japanese Genotype-Phenotype Archive (JGA, http://trace.ddbj.nig.ac.jp/jga), which is hosted by the National Bioscience Database Center (NBDC) and DDBJ with the identifiers JGAS000613 [https://ddbj.nig.ac.jp/resource/jga-study/JGAS000613] and JGAS000677 [https://ddbj.nig.ac.jp/resource/jga-study/JGAS000677]. Detailed information about the data is also available on the NBDC websites [https://humandbs.dbcls.jp/en/hum0394-v1] and [https://humandbs.dbcls.jp/en/hum0068-v9] for the IA and AIS/MIA projects, respectively. These accession numbers contain raw sequencing and image data. These raw data are available under controlled access due to ethical restriction because they are defined as personally identifiable information in Japan. Users require the approval to access the data from NBDC (https://humandbs.dbcls.jp/en/guidelines/data-sharing-guidelines) by applying for the data use (https://humandbs.dbcls.jp/en/data-use). The restrictions for granting data are described in the NBDC web page (https://humandbs.dbcls.jp/en/guidelines/security-guidelines-for-users). The processed data is stored in the database DBKERO (https://kero.hgc.jp/) and made freely available on the project's webpage (https://kero.hgc.jp/Ad-SpatialAnalysis_2024.html). Source data are provided with this paper.

Research involving human participants, their data, or biological material

Policy information about studies with human participants or human data. See also policy information about sex, gender (identity/presentation), and sexual orientation and race, ethnicity and racism.

Reporting on sex and gender

Samples from both female and male were used in this study. For IA cases, we used the samples from six females and two males. For AIS/MIA cases, we used the samples from 16 females and six males. Sex and gender information was not considered for the statistical analyses of this study because the information was not a focus in this study.

Reporting on race, ethnicity, or other socially relevant groupings

We analyzed Japanese lung cancer patients.

Population characteristics

Japanese lung cancer patients
We reported information of IA cases including smoking history and pathological stages in Table 1. We reported the information for AIS/MIA cases in our previous study (Haga Y et al. 2023 Nat Commun) and briefly summarized it in Supplementary Table S1 .

Recruitment

All clinical samples were obtained with the appropriate informed consent by the National Cancer Center and University of Tsukuba, Japan.

Ethics oversight

National Cancer Center, Japan; University of Tsukuba, Japan; The University of Tokyo, Japan

Note that full information on the approval of the study protocol must also be provided in the manuscript.

Field-specific reporting

Please select the one below that is the best fit for your research. If you are not sure, read the appropriate sections before making your selection.

☒ Life sciences ☐ Behavioural & social sciences ☐ Ecological, evolutionary & environmental sciences

For a reference copy of the document with all sections, see nature.com/documents/nr-reporting-summary-flat.pdf

Life sciences study design

All studies must disclose on these points even when the disclosure is negative.

Sample size	No sample-size calculation was performed. Sample size was determined by the availability of the specimens.
Data exclusions	No data were excluded from the analyses.
Replication	For some cases, the data cannot be reproduced because there is limitation for sample amounts of clinical specimens. For some representative cases, we validated the data by analyzing sequential sections.
Randomization	Randomization was not relevant to our study. Groups were determined by invasive or non-invasive phenotypes.
Blinding	Blinding was not relevant to our study. Associations between the results of spatial omics analyses and clinico-pathological information including histological characters are important in this study.

Reporting for specific materials, systems and methods

We require information from authors about some types of materials, experimental systems and methods used in many studies. Here, indicate whether each material, system or method listed is relevant to your study. If you are not sure if a list item applies to your research, read the appropriate section before selecting a response.

Materials & experimental systems

n/a	Involved in the study	
☐	☒	Antibodies
☒	☐	Eukaryotic cell lines
☒	☐	Palaeontology and archaeology
☒	☐	Animals and other organisms
☒	☐	Clinical data
☒	☐	Dual use research of concern
☒	☐	Plants

Methods

n/a	Involved in the study	
☒	☐	ChIP-seq
☒	☐	Flow cytometry
☒	☐	MRI-based neuroimaging

Antibodies

Antibodies used	We used antibodies for PhenoCycler analysis (Akoya Biosciences). All antibodies are available to purchase. Information of all antibodies including clone names and catalogue numbers is shown in Supplementary Table S4 and S5.
Validation	We reported information of manufacturers and catalogue numbers of antibodies in Supplementary Table S4 and S5.<br>For conjugated-antibodies, information is provided in the Akoya Biosciences web page [https://www.akoyabio.com/phenocycler/assays/]. For other antibodies, information of each antibody is provided by the manufacturers' web pages.

Plants

Seed stocks	*Report on the source of all seed stocks or other plant material used. If applicable, state the seed stock centre and catalogue number. If plant specimens were collected from the field, describe the collection location, date and sampling procedures.*
Novel plant genotypes	*Describe the methods by which all novel plant genotypes were produced. This includes those generated by transgenic approaches, gene editing, chemical/radiation-based mutagenesis and hybridization. For transgenic lines, describe the transformation method, the number of independent lines analyzed and the generation upon which experiments were performed. For gene-edited lines, describe the editor used, the endogenous sequence targeted for editing, the targeting guide RNA sequence (if applicable) and how the editor was applied.*
Authentication	*Describe any authentication procedures for each seed stock used or novel genotype generated. Describe any experiments used to assess the effect of a mutation and, where applicable, how potential secondary effects (e.g. second site T-DNA insertions, mosiacism, off-target gene editing) were examined.*
